# Indium(III)-Catalyzed Synthesis of Pyrroles and Benzo[*g*]indoles by Intramolecular Cyclization of Homopropargyl Azides

**DOI:** 10.1021/acs.joc.4c01768

**Published:** 2024-10-15

**Authors:** Ana Da Lama, José Pérez Sestelo, Luis A. Sarandeses, M. Montserrat Martínez

**Affiliations:** CICA, Centro Interdisciplinar de Química e Bioloxía and Departamento de Química, Universidade da Coruña, 15071 A Coruña, Spain

## Abstract

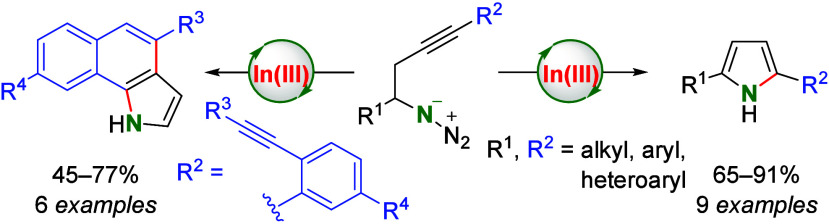

Pyrroles, privileged structural motifs in drug and material science, have been synthesized by indium(III)-catalyzed intramolecular cyclization of homopropargyl azides. This methodology exhibits a broad substrate scope, providing substituted pyrroles and bispyrroles in good yields. Furthermore, an atom-economical sequential method for the synthesis of benzo[*g*]indoles has been discovered from azido-diynes using InCl_3_ as catalyst. The method involves two successive intramolecular indium-catalyzed 5*-endo-dig* alkyne hydroamination and a hydroarylation reactions with 6*-endo-dig* regioselectivity.

Pyrroles and indoles are one of the most ubiquitous structural scaffolds, embedded in natural products,^1^ biologically active substances and pharmaceuticals,^2^ and materials science and industry.^3^ Additionally, these compounds are suitable building blocks for the preparation of bioactive compounds and other useful synthetic heterocyclic derivatives.^4^

Different approaches to the pyrrole nucleus have been described, ranging from the classical Knorr and Paal–Knorr reactions,^5^ Hantzsch reaction,^6^ and Barton–Zard reaction^7^ to modern methods of multicomponent reactions using transition-metal catalysis.^8^ One of the most valuable approaches in this area involves inter- or intramolecular hydroamination of alkynes.^9^ In these atom-economical processes, a nucleophilic nitrogen reacted with a metal-activated alkyne to produce the final pyrrole after transposition and rearrangement steps. Among the nucleophilic counterparts in this reaction, the use of azides was pioneered by Toste, under gold(I) catalysis, in a process named intramolecular Schmidt reaction.^10^ Other similar examples using platinum, zinc, or mercury salts as catalyst have been reported.^11^

Recently, indium catalysis has emerged as an alternative synthetic tool to precious transition metals such as platinum or gold for the activation of alkynes, thereby facilitating nucleophilic addition to the alkyne moiety to afford cyclization products such as indoles.^12^ As part of our current research on the indium(III) salts as π-Lewis acid catalysts in alkyne hydrofunctionalizations,^13^ we have reported hydroarylation and hydroalkoxylation reactions in the synthesis of polycyclic structures.^13,14^ Although the indium-catalyzed hydroamination reactions are difficult due to the coordination of the nitrogen atom with the catalyst,^15^ we have developed indium-catalyzed intramolecular hydroaminations using *N*-tosylamines in the synthesis of benzannulated spiroaminals, dihydroisoquinolines, and bicyclic structures.^16^ Following this research, we envisioned that the Lewis acid ability of indium(III) would be useful for the synthesis of pyrroles and related heterocycles as an economical and low-toxicity alternative. In this communication, we report the synthesis of pyrroles by indium-catalyzed intramolecular reaction of homopropargyl azides and the synthesis of benzo[*g*]indoles through indium-cascade cycloisomerization of azido-diynes.

At the outset of our research, we studied the reactivity of 4-azido-1-phenyl-1-butyne (**1a**) under indium(III) catalysis. After optimization, we found that 2-phenylpyrrole (**2a**) was obtained in good yield using InCl_3_ (5 mol %) in DCE at 80 °C after 18 h of reaction (90%, Table 1, entry 1). The cyclization proceeded with complete 5-*endo-dig* regioselectivity and loss of molecular nitrogen as the leaving group. When the reaction is carried out at rt, the yield decreases (62%, entry 2), and among other different indium(III) salts, the use of InBr_3_ in DCE at 80 °C or In(OTf)_3_ in toluene at 100 °C showed similar results to InCl_3_ (64–74%, entries 3 and 5). Surprisingly, InI_3_ and In(NTf_2_)_3_ gave a much lower yield (12–24%, entries 4 and 6). However, GaCl_3_ (5 mol %) provided **2a** in 78% yield (entry 7). These results illustrate the fundamental role of the indium(III) ligand or counterion, probably due to the different π interactions with the alkyne and the interaction with the basic azide moiety. Finally, the synthetic utility of this novel In(III)-catalyzed cyclization was demonstrated by scaling up the reaction (up to 3.0 mmol of **1a**), obtaining 80% isolated yield of **2a**.

**Table 1:**
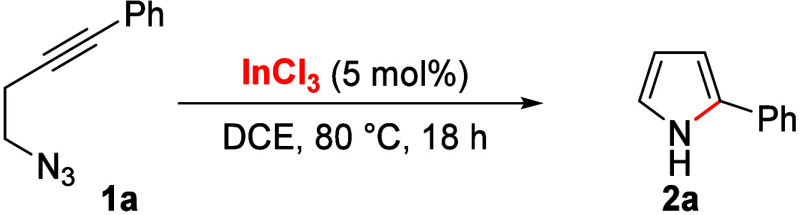
Optimization Studies of the Reaction Conditions^a^

entry	deviation from optimized conditions	yield (%)^b^^,^^c^
1	none	90 (7)
2	rt, 2 days	62 (36)
3	InBr_3_ (5 mol %)	64 (26)
4	InI_3_ (5 mol %)	12 (72)
5	In(OTf)_3_ (5 mol %), toluene, 100 °C, 18 h	74 (9)
6	In(NTf_2_)_3_ (5 mol %), toluene, 100 °C, 18 h	24 (51)
7	GaCl_3_ (5 mol %)	78 (5)

a Reactions performed on a 0.30 mmol scale (0.06 M solution).

b Isolated yields.

c In parentheses, yield of unreacted **1a** recovered.

Using the optimized conditions, we next sought to expand this method to differently substituted homopropargyl azides. In this research, we found that the presence of a methoxy substituent in the aryl moiety gave a good yield of **2b** (90% yield, Table 2, entry 2). In our next experiment, taking into account that pyrroles bearing a 4-trifluorophenyl moiety have biological applications,^2a^ we explored the reaction using azide **1c**, which also gave a good yield of the corresponding pyrrole **2c** (87% yield, entry 3). Analogously, using 4-azido-1-(2-thienyl)-1-butyne (**1d**) as the starting material afforded 2-(thien-2-yl)pyrrole satisfactorily (**2d**, 91% yield, entry 4). We also found that the use of a benzylic azide (**1e**) afforded 2,5-diphenylpyrrole in good yield (**2e**, 78% yield, entry 5) or from the 2-alkynyl cyclohexyl azide **1f** gave the bicyclic pyrrole **2f** in 84% yield (entry 6), although a loading to 10 mol % of InCl_3_ was necessary. The reactivity of secondary homopropargyl azides was also studied with azide **1g**, obtaining 2,5-dialkylpyrrole **2g** in 65% yield (entry 7). The suitability of indium catalysis to perform a double intramolecular cyclization from bis-homopropargyl azide **1h** was also demonstrated with the synthesis of 1,4-bis(pyrrol-2-yl)benzene (**2h**, 84% yield, entry 8). Analogously, the double cyclization of **1i** gave 2,5-di(pyrrol-2-yl)thiophene (**2i**) in 65% yield (entry 9). To the best of our knowledge, this reaction constitutes the sole example of metal-catalyzed double cyclization to access bispyrroles under metal catalysis. These donor–acceptor compounds (**2h**, **2i**) are particularly attractive for the preparation of electrically conducting and biodegradable π-conjugated polymers with biomedical applications.^17^

**Table 2:**
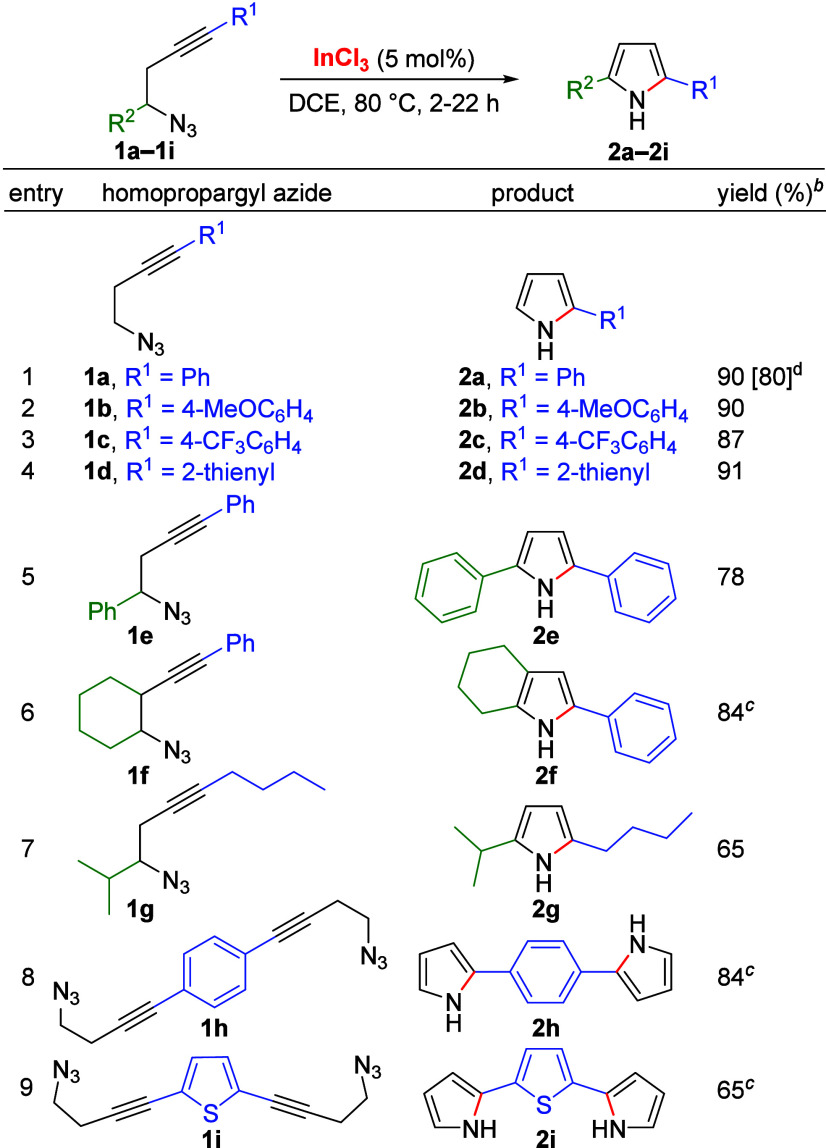
Scope of the Synthesis of Pyrroles^a^

a Reactions performed on a 0.40 mmol scale (0.08 M solution).

b Isolated yields.

c Reaction with 10 mol % of InCl_3_.

d Isolated yield of **2a** on 3 mmol scale.

To study further the ability of indium salts to promote intramolecular cyclization reactions by electrophilic activation of alkynes, we envisioned the possibility of a subsequent intramolecular hydroarylation of the pyrroles. The process should require, after initial pyrrole formation, an additional indium-catalyzed intramolecular hydroarylation reaction. In this sense, the intramolecular hydroarylation of alkynes with heterocycles was already established for alkynylaryl thiophenes^18^ and benzofurans^19^ under different metal catalysis and for alkynylaryl indoles under Au(I)^20^ or In(III) catalysis.^21^ For this purpose, we prepared the alkynylaryl homopropargyl azide **3a** (Scheme 1) bearing an additional triple bond in the ortho position with respect to the azide substituent. Under the previously optimized conditions, reaction of **3a** with InCl_3_ (10 mol %) in DCE at 80 °C for 48 h afforded the benzo[*g*]indole **4a** in 25% yield (Scheme 1), and the use of InI_3_ (10 mol %) did not improve the yield (24%). Nevertheless, a satisfactory 75% yield of **4a** was obtained using InCl_3_ (15 mol %) in toluene at 110 °C, while the use of InBr_3_ under the same conditions did not give better results (46%). The formation of **4a** can be explained by an intramolecular 5-*endo-dig* hydroamination followed by a regioselective 6-*endo-dig* hydroarylation, which is supported by the detection of pyrrole intermediate **5b** (Scheme 1) by ^1^H NMR during the conversion of **3b** into benzo[*g*]indole **4b** (Supporting Information, Figure S1).

**Scheme 1:**
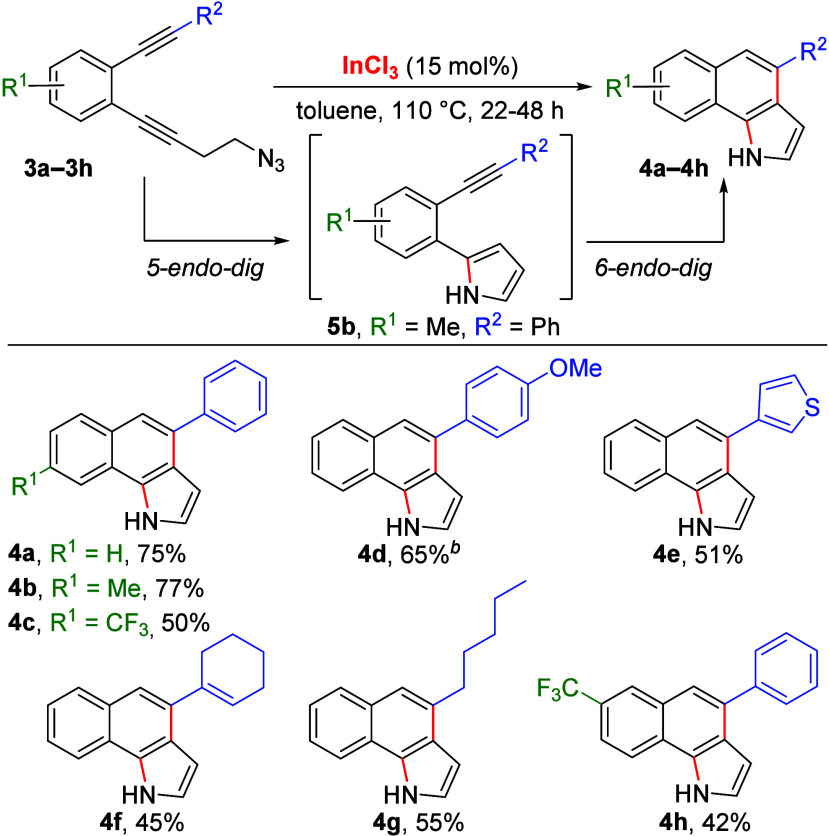
Synthesis of Benzo[*g*]indoles by In(III)-Catalyzed Double-Cyclization Reaction of Azido-Diynes Isolated yields. Reaction performed using 20 mol % of InCl_3_.

This cascade reaction constitutes a novel approach to the synthesis of benzo[*g*]indoles,^22^ a privileged family of building blocks of biologically active molecules, since some derivatives have interesting anti-inflammatory and antiproliferative properties,^23^ as well as in materials science by their interesting photophysical behavior.^24^

Upon establishing the optimal conditions, we explored the substrate scope with differently substituted azido-diynes. In this venture, we found that the substituents in the central aryl group have a limited influence on the yields, where the presence of a methyl group gave similar results (**4b**, 77% yield, Scheme 1), while a trifluoromethyl moiety afforded a slightly lower yield (**4c**, 50%). The synthetic utility of our strategy was expanded by the introduction of different substituents in the distal alkyne with respect to the azide. To our delight, reaction of dialkynyl azide containing electron-donating groups such as 4-methoxyphenyl and 3-thienyl groups provided satisfactory yields (**4d** and **4e**, 65% and 51%, respectively). Conversely, the presence of an alkenyl substituent afforded a slightly lower yield (**4f**, 45%), probably due to the low electrophilic character of the triple bond. However, the reaction with azide **3g** bearing an alkyl substituent yielded cyclized product **4g** (55% yield). Finally, we found that the reaction using azide **3h** afforded benzo[*g*]indole **4h** in 42% yield (Scheme 1).

Although the reaction mechanism was not studied, on the basis of previous reports for the synthesis of pyrroles from homopropargyl azides^10,11,25^ and the good alkynophilicity of indium(III) halides toward alkynes,^13^ we propose a η^2^ coordination of the indium(III) catalytic species with the alkyne that triggers a intramolecular 5-*endo-dig* anti nucleophilic addition of the internal nitrogen atom of the azide (Scheme 2). Further elimination of molecular nitrogen and a 1,2-H shift should regenerate the active catalyst and a 2*H*-pyrrole which tautomerizes into the 1*H*-pyrrole by a 1,5-H shift. However, an alternative pathway involving initial indium-promoted nitrene formation followed by indium-catalyzed hydroamination could not be excluded. For the cascade reaction of azido-diynes, after the pyrrole formation, a new activation of the distal alkyne by indium catalysis followed by regioselective intramolecular 6-*endo-dig* hydroarylation by the C-3 atom of the pyrrole ring is proposed.^18a,18b,21^

**Scheme 2:**
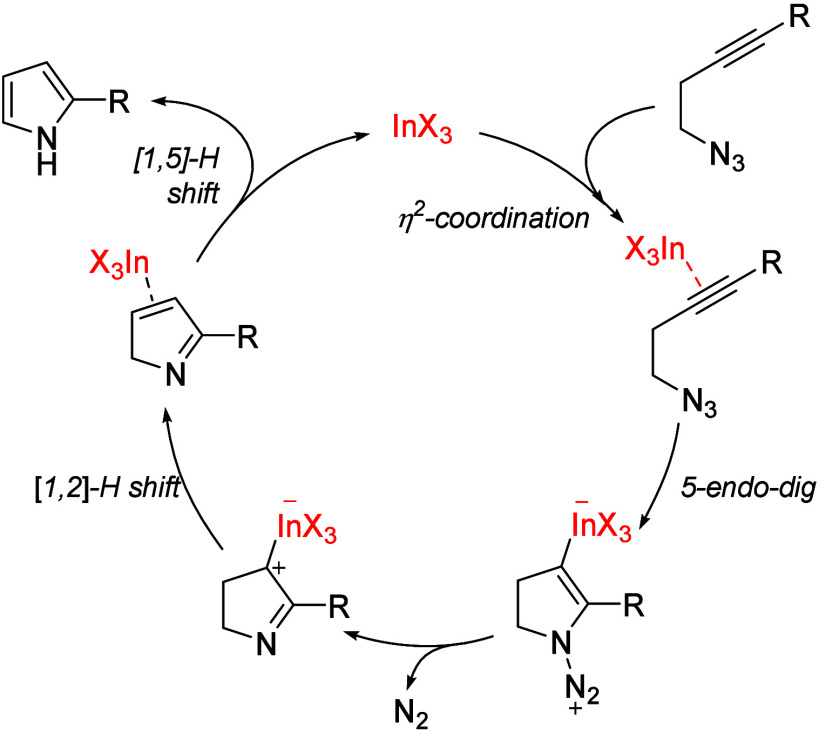
Plausible Mechanism

In conclusion, we have demonstrated the usefulness of indium(III) as an effective catalyst for the synthesis of a variety of substituted pyrroles incorporating aryl, heteroaryl, and alkyl groups by intramolecular cyclization of homopropargyl azides. This atom-economic method provides a better scope in good yields than previous metal-catalyzed cyclizations, providing a more environmentally friendly alternative. We also discovered a new indium(III)-catalyzed cascade reaction for the synthesis of a range of substituted benzo[*g*]indoles from azido-diynes. The reaction involves two sequential indium-catalyzed processes: a 5*-endo-dig* hydroamination followed by a selective 6*-endo-dig* hydroarylation. Further studies on these reactions, including elucidation of the mechanism, are in progress and will be published in due course.

## Experimental Section

### General Information and Materials

All reactions were carried out in dried glassware under an argon atmosphere using standard gastight syringes and septa. Dry DCE, toluene, and other commercially available reagents were used as received. Reaction temperatures refer to external bath temperatures. All indium(III) salts were used as received under argon in a glovebox system. Reactions were monitored by thin-layer chromatography in precoated silica gel foils using UV light as the visualizing agent and ethanolic phosphomolybdic acid as the developing agent. Flash column chromatography was performed using 230–400 mesh silica gel. ^1^H and ^13^C{^1^H} NMR spectra were recorded at room temperature by using a 300 MHz spectrometer and calibrated to the solvent peak. DEPT data were used to assign carbon types. Chemical shifts are reported in ppm (δ) relative to the solvent CDCl_3_ (δ_H_ 7.26 ppm and δ_C_ 77.1 ppm) and acetone-*d*_6_ (2.05 and 29.8 ppm). Mass spectra were recorded on a QSTAR Elite hybrid quadrupole time-of-flight (TOF) ESI mass spectrometer, operating in positive ionization mode. IR spectra were recorded with attenuated total reflectance (ATR). Melting points were recorded in an open capillary tube and were uncorrected.

#### Chemical Safety Caution!

Azides (organic and inorganic) are potentially explosive substances, in particular, with low molecular weight. Although no safety problems were encountered when dealing with sodium azide and synthesized azides, we encourage employing plastic spatulas, avoiding the use of acidic solutions, and taking precautions with the azide waste to prevent explosions.

All reactants and reagents were handled in a fume hood.

### General Procedure for the In(III)-Catalyzed Intramolecular Cyclization of Homopropargyl Azides 2

A snap cap vial (20 mL) equipped with a magnetic stir bar was charged with InCl_3_ (5 mol %) inside of a glovebox. Then, a solution of the appropriate azide (1 equiv) in DCE (5 mL) was added to the vial. The reaction mixture was heated in an oil bath at 80 °C and monitored by TLC until the starting material was consumed. The reaction mixture was cooled to ambient temperature, and the solvent was removed under reduced pressure. The resulting crude was purified by flash column chromatography on silica gel (EtOAc/hexanes) to afford, after concentration and high-vacuum drying, the corresponding pyrroles.

#### 2-Phenyl-1*H*-pyrrole (**2a**)^26^

Following the general procedure, compound **2a** was obtained from azide **1a** (67.5 mg, 0.395 mmol) and InCl_3_ (4.4 mg, 0.0197 mmol) in DCE (5 mL) at 80 °C for 20 h followed by purification (5–12% EtOAc/hexanes). White powder, 90% yield (51.0 mg). Mp = 130–131 °C (128–130 °C).^14^ IR (ATR): ν 3432, 3388, 2923, 1465 cm^–1^. ^1^H NMR (300 MHz, CDCl_3_): δ 8.37 (s, 1H), 7.46–7.43 (m, 2H), 7.37–7.32 (m, 2H), 7.23–7.18 (m, 1H), 6.83–6.81 (m, 1H), 6.54–6.53 (m, 1H), 6.31–6.29 (m, 1H). ^13^C{^1^H} NMR (75 MHz, CDCl_3_): δ 132.8 (C), 132.2 (C), 129.0 (2 × CH), 126.3 (CH), 123.9 (2 × CH), 118.9 (CH), 110.2 (CH), 106.0 (CH). HRMS (ESI) calcd for C_10_H_9_N [M]^+^ 143.0735, found 143.0724.

##### Scale-up Experiment

Following the general procedure, compound **2a** was obtained from azide **1a** (508 mg, 2.97 mmol) and InCl_3_ (32.8 mg, 0.148 mmol) in DCE (25 mL) at 80 °C for 22 h followed by purification (10% EtOAc/hexanes). White powder, 80% yield (343 mg).

#### 2-(4-Methoxyphenyl)-1*H*-pyrrole (**2b**)^27^

Following the general procedure, compound **2b** was obtained from azide **1b** (100.4 mg, 0.497 mmol) and InCl_3_ (5.5 mg, 0.025 mmol) in DCE (5 mL) at 80 °C for 2 h followed by purification (10–15% EtOAc/hexanes). White powder, 90% yield (77.9 mg). Mp = 143–145 °C (145–147 °C).^15^ IR (ATR): ν 3431, 2920, 2868, 1509, 1302 cm^–1^. ^1^H NMR (300 MHz, CDCl_3_): δ 8.33 (s, 1H), 7.40 (d, *J* = 8.6 Hz, 2H), 6.93 (d, *J* = 8.6 Hz, 2H), 6.80 (s, 1H), 6.44 (s, 1H), 6.31 (s, 1H), 3.84 (s, 3H). ^13^C{^1^H} NMR (75 MHz, CDCl_3_): δ 158.3 (C), 132.1 (C), 125.9 (C), 125.3 (2 × CH), 118.3 (CH), 114.4 (2 × CH), 109.9 (CH), 104.9 (CH), 55.4 (CH_3_). HRMS (ESI) calcd for C_11_H_11_NO [M]^+^ 173.0841, found 173.0835.

#### 2-(4-(Trifluoromethyl)phenyl)-1*H*-pyrrole (**2c**)^28^

Following the general procedure, compound **2c** was obtained from azide **1c** (95.7 mg, 0.40 mmol) and InCl_3_ (4.4 mg, 0.020 mmol) in DCE (5 mL) at 80 °C for 20 h followed by purification (5% EtOAc/hexanes). White powder, 87% yield (73 mg). Mp = 162–164 °C (158 °C).^16^ IR (ATR): ν 3401, 2920, 2851, 1738, 1343, 1112 cm^–1^. ^1^H NMR (300 MHz, CDCl_3_): δ 8.49 (s, 1H), 7.58 (q, *J* = 8.6, 11.7 Hz, 4H), 6.93 (s, 1H), 6.65 (s, 1H), 6.37–6.34 (m, 1H). ^13^C{^1^H} NMR (75 MHz, CDCl_3_): δ 135.9 (C), 130.6 (C), 127.7 (q, ^2^*J*_CF_ = 32.8 Hz, C), 125.9 (q, ^3^*J*_CF_ = 3.9 Hz, 2 × CH), 124.3 (q, ^1^*J*_CF_ = 271.6 Hz, CF_3_), 123.6 (2 × CH), 120.2 (CH), 110.7 (CH), 107.7 (CH). ^19^F NMR (282 MHz, CDCl_3_): δ – 62.37 (s, CF_3_). HRMS (ESI) calcd for C_11_H_8_F_3_N [M]^+^ 211.0609, found 211.0600.

#### 2-(Thiophen-2-yl)-1*H*-pyrrole (**2d**)^29^

Following the general procedure, compound **2d** was obtained from azide **1d** (102.9 mg, 0.581 mmol) and InCl_3_ (6.4 mg, 0.029 mmol) in DCE (5 mL) at 80 °C for 2 h followed by purification (5% EtOAc/hexanes). White powder, 91% yield (76.6 mg). Mp = 74–76 °C (78–80 °C).^17^ IR (ATR): ν 3365, 2923, 2852, 1461, 1123 cm^–1^. ^1^H NMR (300 MHz, CDCl_3_): δ 8.28 (s, 1H), 7.17 (t, *J* = 3.2 Hz, 1H), 7.04 (d, *J* = 3.1 Hz, 2H), 6.81 (s, 1H), 6.46 (s, 1H), 6.31–6.28 (m, 1H). ^13^C{^1^H} NMR (75 MHz, CDCl_3_): δ 136.3 (C), 127.7 (CH), 126.7 (C), 122.7 (CH), 120.9 (CH), 118.6 (CH), 110.1 (CH), 106.8 (CH). HRMS (ESI) calcd for C_8_H_7_NS [M]^+^ 149.0299, found 149.0292.

#### 2,5-Diphenyl-1*H*-pyrrole (**2e**)^30^

Following the general procedure, compound **2e** was obtained from azide **1e** (70.0 mg, 0.283 mmol) and InCl_3_ (3.1 mg, 0.014 mmol) in DCE (5 mL) at 80 °C for 23 h followed by purification (5% EtOAc/hexanes). White powder, 78% yield (48.4 mg). Mp = 143–144 °C (142–143 °C).^18^ IR (ATR): ν 3457, 2922, 2855, 1735, 1460 cm^–1^. ^1^H NMR (300 MHz, CDCl_3_): δ 8.47 (s, 1H), 7.43 (d, *J* = 7.5 Hz, 4H), 7.29 (t, *J* = 7.5 Hz, 4H), 7.13 (t, *J* = 7.3 Hz, 2H), 6.49 (d, *J* = 2.5 Hz, 2H). ^13^C{^1^H} NMR (75 MHz, CDCl_3_): δ 133.2 (2 × C), 132.5 (2 × C), 129.0 (4 × CH), 126.4 (2 × CH), 123.8 (4 × CH), 108.0 (2 × CH). HRMS (ESI) calcd for C_16_H_13_N [M]^+^ 219.1048, found 219.1041.

#### ((2-Azidocyclohexyl)ethynyl)benzene (**2f**)^10^

Following the general procedure, compound **2f** was obtained from azide **1f** (54.4 mg, 0.24 mmol) and InCl_3_ (5.3 mg, 0.024 mmol) in DCE (5 mL) at 80 °C for 3 h followed by purification (4% EtOAc/hexanes). White powder, 84% yield (40.0 mg). Mp = 110–112 °C (111–113 °C).^19^ IR (ATR): ν 3347, 2921, 2851, 1703, 1447 cm^–1^. ^1^H NMR (300 MHz, CDCl_3_): δ 7.92 (s, 1H), 7.44–7.30 (m, 4H), 7.15 (t, *J* = 7.3 Hz, 1H), 6.29 (d, *J* = 2.4 Hz, 1H), 2.65 (t, *J* = 5.7 Hz, 2H), 2.55 (t, *J* = 5.9 Hz, 2H), 1.90–1.74 (m, 4H). ^13^C{^1^H} NMR (75 MHz, CDCl_3_): δ 133.2 (C), 130.3 (C), 128.8 (2 × CH), 128.5 (C), 125.6 (CH), 123.4 (2 × CH), 119.0 (C), 105.2 (CH), 23.8 (CH_2_), 23.4 (CH_2_), 22.9 (CH_2_), 22.9 (CH_2_). HRMS (ESI) calcd for C_14_H_15_N [M]^+^ 197.1204, found 197.1198.

#### 2-Butyl-5-isopropyl-1*H*-pyrrole (**2g**)

Following the general procedure, compound **2g** was obtained from azide **1g** (57.4 mg, 0.297 mmol) and InCl_3_ (3.3 mg, 0.015 mmol) in DCE (5 mL) at 80 °C for 6 h followed by purification (-hexanes). Brown powder, 65% yield (31.8 mg). Mp = 92–94 °C. IR (ATR): ν 3301, 2921, 2851, 1462 cm^–1^. ^1^H NMR (300 MHz, CDCl_3_): δ 7.60 (s, 1H), 5.79 (d, *J* = 2.0 Hz, 2H), 2.91–2.84 (m, 1H), 2.56 (t, *J* = 7.6 Hz, 2H), 1.65–1.57 (m, 2H), 1.42–1.35 (m, 2H), 1.29–1.23 (m, 6H), 0.93 (t, *J* = 7.3 Hz, 3H). ^13^C{^1^H} NMR (75 MHz, CDCl_3_): δ 137.3 (C), 131.2 (C), 104.3 (CH), 102.6 (CH), 31.8 (CH_2_), 29.7 (CH_2_), 27.5 (CH_2_), 27.0 (CH), 22.7 (2 × CH_3_), 13.9 (CH_3_). HRMS (ESI) calcd for C_11_H_19_N [M]^+^ 165.1517, found 165.1510.

#### 1,4-Di(1*H*-pyrrol-2-yl)benzene (**2h**)^31^

Following the general procedure, compound **2h** was obtained from compound **1h** (49.6 mg, 0.188 mmol) and InCl_3_ (4.2 mg, 0.019 mmol) in DCE (5 mL) at 80 °C for 6 h followed by purification (2% MeOH/CH_2_Cl_2_). Green powder, 84% yield (34.6 mg). Mp > 300 °C. IR (ATR): ν 3365, 2927, 2854, 1980, 1217 cm^–1^. ^1^H NMR (300 MHz, acetone-*d*_6_): δ 10.39 (s, 2H), 7.55 (s, 4H), 6.80–6.77 (m, 2H), 6.46–6.44 (m, 2H), 6.11–6.09 (m, 2H). ^13^C{^1^H} NMR (75 MHz, acetone-*d*_6_): δ 132.6 (2 × C), 131.7 (2 × C), 124.8 (4 × CH), 119.8 (2 × CH), 110.3 (2 × CH), 106.2 (2 × CH). HRMS (ESI) calcd for C_14_H_12_N_2_ [M]^+^ 208.1000; found 208.0996.

#### 2,5-Di(1*H*-pyrrol-2-yl)thiophene (**2i**)^32^

Following the general procedure, compound **2i** was obtained from compound **1i** (103.0 mg, 0.381 mmol) and InCl_3_ (8.4 mg, 0.038 mmol) in DCE (5 mL) at 80 °C for 22 h followed by purification (5–20% EtOAc/hexanes). Green powder, 65% yield (52.9 mg). Mp = 198–199 °C (195 °C).^21^ IR (ATR): ν 3377, 2922, 2852, 1457 cm^–1^. ^1^H NMR (300 MHz, acetone-*d*_6_): δ 10.45 (s, 2H), 7.07 (s, 2H), 6.84–6.82 (m, 2H), 6.34–6.31 (m, 2H), 6.16–6.13 (m, 2H). ^13^C{^1^H} NMR (75 MHz, acetone-*d*_6_): δ 133.5 (2 × C), 126.4 (2 × C), 120.7 (2 × CH), 118.7 (2 × CH), 109.2 (2 × CH), 105.9 (2 × CH). HRMS (ESI): calcd for C_12_H_10_N_2_S [M]^+^ 214.0565, found 214.0560.

### General Procedure for the Cascade In(III)-Catalyzed Cycloisomerization of Azido-Diynes 4

A snap cap vial (20 mL) equipped with a magnetic stir bar was charged with InCl_3_ (15 mol %) inside of a glovebox. Then, a solution of the appropriate azide (1 equiv) in toluene (5 mL) was added to the vial. The reaction mixture was heated in an oil bath at 110 °C and monitored by TLC until the starting material was consumed. The reaction mixture was cooled to ambient temperature, and the solvent was removed under reduced pressure. The resulting crude was purified by flash column chromatography on silica gel (EtOAc/hexanes) to afford, after concentration and high-vacuum drying, the corresponding benzopyrroles.

#### 4-Phenyl-1*H*-benzo[*g*]indole (**4a**)

Following the general procedure, compound **4a** was obtained from azide **3a** (50.3 mg, 0.185 mmol) and InCl_3_ (6.2 mg, 0.028 mmol) in toluene (5 mL) at 110 °C for 48 h followed by purification (4–6% EtOAc/hexanes). Brown oil, 75% yield (34.0 mg). IR (ATR): ν 3500, 2924, 2853, 1717, 1495, 1378 cm^–1^. ^1^H NMR (300 MHz, CDCl_3_): δ 8.97 (s, 1H), 8.02 (d, *J* = 8.2 Hz, 2H), 7.85 (d, *J* = 7.7 Hz, 2H), 7.62–7.44 (m, 6H), 7.31 (t, *J* = 2.7 Hz, 1H), 6.87 (t, *J* = 2.7 Hz, 1H). ^13^C{^1^H} NMR (75 MHz, CDCl_3_): δ 141.1 (C) 134.5 (C), 130.9 (C), 130.8 (C), 129.1 (CH), 128.9 (2 × CH), 128.6 (2 × CH), 127.3 (CH), 125.5 (CH), 124.3 (CH), 122.9 (C), 122.4 (CH), 121.1 (C), 120.0 (CH), 119.3 (CH), 104.0 (CH). HRMS (ESI) calcd for C_18_H_13_N [M]^+^ 243.1048, found 243.1050.

#### 8-Methyl-4-phenyl-1*H*-benzo[*g*]indole (**4b**)

Following the general procedure, compound **4b** was obtained from azide **3b** (74.0 mg, 0.259 mmol) and InCl_3_ (8.6 mg, 0.039 mmol) in toluene (5 mL) at 110 °C for 22 h followed by purification (6% EtOAc/hexanes). Green oil, 77% yield (51.2 mg). IR (ATR): ν 3430, 2919, 2851, 2362, 1402 cm^–1^. ^1^H NMR (300 MHz, CDCl_3_): δ ^1^H NMR (300 MHz, CDCl_3_): δ 8.81 (s, 1H), 7.90 (d, *J* = 8.3 Hz, 1H), 7.84 (d, *J* = 7.2 Hz, 2H), 7.75 (s, 1H), 7.59–7.53 (m, 3H), 7.48–7.42 (m, 1H), 7.32 (dd, *J* = 8.3, 1.6 Hz, 1H), 7.22 (t, *J* = 2.8 Hz, 1H), 6.86 (t, *J* = 2.5 Hz, 1H), 2.60 (s, 3H). ^13^C{^1^H} NMR (75 MHz, CDCl_3_): δ 141.3 (C), 135.3 (2 × C), 133.6 (C), 130.6 (C), 128.9 (CH), 128.9 (2 × CH), 128.6 (2 × CH), 127.2 (CH), 126.4 (CH), 123.0 (C), 122.3 (CH), 121.2 (C), 119.9 (CH), 118.7 (CH), 104.0 (CH), 22.0 (CH_3_). HRMS (ESI) calcd for C_19_H_15_NNa [M + Na]^+^ 280.1102, found 280.1109.

#### 4-Phenyl-8-(trifluoromethyl)-1*H*-benzo[*g*]indole (**4c**)

Following the general procedure, compound **4c** was obtained from azide **3c** (77.1 mg, 0.227 mmol) and InCl_3_ (7.5 mg, 0.034 mmol) in toluene (5 mL) at 110 °C for 48 h followed by purification (5% EtOAc/hexanes). Brown oil, 50% yield (35.3 mg). IR (ATR): ν 3429, 2924, 2854, 1327, 1121 cm^–1^. ^1^H NMR (300 MHz, CDCl_3_): δ 9.09 (s, 1H), 8.32 (s, 1H), 8.06 (d, *J* = 8.4 Hz, 1H), 7.80 (d, *J* = 7.6 Hz, 2H), 7.65–7.44 (m, 5H), 7.35 (t, *J* = 2.7 Hz, 1H), 6.88 (t, *J* = 2.5 Hz, 1H). ^13^C{^1^H} NMR (75 MHz, CDCl_3_): δ 140.5 (C), 136.9 (C), 132.1 (C), 131.0 (C), 129.8 (CH), 128.9 (2 × CH), 128.7 (2 × CH), 127.7 (CH), 126.9 (q, ^2^*J*_CF_ = 32.1 Hz, C), 124.7 (q, ^1^*J*_CF_ = 272.4 Hz, CF_3_), 123.9 (C), 123.2 (CH), 120.0 (C), 119.9 (q, ^3^*J*_CF_ = 3.4 Hz, CH), 119.6 (CH), 117.1 (q, ^3^*J*_CF_ = 4.3 Hz, CH), 104.4 (CH). ^19^F NMR (282 MHz, CDCl_3_): δ – 62.95 (s, CF_3_). HRMS (ESI) calcd for C_19_H_12_F_3_NNa [M + Na]^+^ 334.2970, found 334.2960.

#### 4-(4-Methoxyphenyl)-1*H*-benzo[*g*]indole (**4d**)

Following the general procedure, compound **4d** was obtained from azide **3d** (102.0 mg, 0.338 mmol) and InCl_3_ (20 mol %, 15.0 mg, 0.067 mmol) in toluene (5 mL) at 110 °C for 48 h followed by purification (3–5% EtOAc/hexanes). Yellow oil, 65% yield (60.0 mg). IR (ATR): ν 3424, 2954, 2922, 1509, 1462 cm^–1^. ^1^H NMR (300 MHz, CDCl_3_): δ 8.98 (s, 1H), 7.98 (t, *J* = 6.1 Hz, 2H), 7.75 (d, *J* = 8.9 Hz, 2H), 7.56–7.43 (m, 3H), 7.30 (t, *J* = 2.7 Hz, 1H), 7.08 (d, *J* = 8.5 Hz, 2H), 6.85 (t, *J* = 2.8 Hz, 1H), 3.91 (s, 3H). ^13^C{^1^H} NMR (75 MHz, CDCl_3_): δ 159.1 (C), 134.2 (C), 133.7 (C), 130.9 (C), 129.9 (2 × CH), 128.9 (CH), 125.2 (CH), 124.3 (CH), 123.0 (C), 122.3 (CH), 121.0 (C), 120.9 (C), 119.5 (CH), 119.2 (CH), 114.0 (2 × CH), 104.1 (CH), 55.4 (CH_3_). HRMS (ESI) calcd for C_19_H_15_NNaO [M + Na]^+^ 296.1051, found 296.1045.

#### 4-(Thiophen-3-yl)-1*H*-benzo[*g*]indole (**4e**)

Following the general procedure, compound **4e** was obtained from azide **3e** (100.2 mg, 0.360 mmol) and InCl_3_ (12.0 mg, 0.054 mmol) in toluene (5 mL) at 110 °C for 48 h followed by purification (3–5% EtOAc/hexanes). Brown oil, 51% yield (45.7 mg). IR (ATR): ν 3430, 2954, 2922, 1462, 1377 cm^–1^. ^1^H NMR (300 MHz, CDCl_3_): δ 8.82 (s, 1H), 7.84 (d, *J* = 8.4 Hz, 2H), 7.56–7.51 (m, 2H), 7.50–7.47 (m, 1H), 7.43–7.31 (m, 3H), 7.17 (t, *J* = 2.3 Hz, 1H), 6.81 (t, *J* = 2.6 Hz, 1H). ^13^C{^1^H} NMR (75 MHz, CDCl_3_): δ 141.9 (C), 130.9 (C), 130.7 (C), 129.1 (C), 128.9 (CH), 128.2 (CH), 125.6 (CH), 125.5 (CH), 124.4 (CH), 122.7 (C), 122.4 (CH), 122.2 (CH), 121.1 (C), 119.5 (CH), 119.3 (CH), 104.0 (CH). HRMS (ESI) calcd for C_16_H_11_NS [M]^+^ 249.0612, found 249.0616.

#### 4-(Cyclohex-1-en-1-yl)-1*H*-benzo[*g*]indole (**4f**)

Following the general procedure, compound **4f** was obtained from azide **3f** (96.0 mg, 0.349 mmol) and InCl_3_ (11.6 mg, 0.052 mmol) in toluene (5 mL) at 110 °C for 48 h followed by purification (3% EtOAc/hexanes). Yellow oil, 45% yield (38.8 mg). IR (ATR): ν 3426, 2925, 2855, 1462, 1381 cm^–1^. ^1^H NMR (300 MHz, CDCl_3:_) δ 8.70 (s, 1H), 7.81–7.77 (m, 2H), 7.38–7.28 (m, 3H), 7.11 (t, *J* = 2.7 Hz, 1H), 6.72 (t, *J* = 2.5 Hz, 1H), 6.14–6.10 (m, 1H), 2.53–2.48 (m, 2H), 2.24–2.20 (m, 2H), 1.82–1.65 (m, 4H). ^13^C{^1^H} NMR (75 MHz, CDCl_3_): δ 137.3 (C), 137.0 (C), 130.7 (C), 130.7 (C), 128.8 (CH), 126.9 (CH), 124.9 (CH), 124.1 (CH), 122.6 (C), 121.8 (CH), 120.8 (C), 119.1 (CH), 117.5 (CH), 104.4 (CH), 29.2 (CH_2_), 25.8 (CH_2_), 23.3 (CH_2_), 22.5 (CH_2_). HRMS (ESI) calcd for C_18_H_18_N [M + H]^+^ 248.1395, found 248.1405.

#### 4-(Pentyl)-1*H*-benzo[*g*]indole (**4g**)

Following the general procedure, compound **4g** was obtained from azide **3g** (80.6 mg, 0.30 mmol) and InCl_3_ (10.1 mg, 0.0546 mmol) in toluene (5 mL) at 110 °C for 48 h followed by purification (3% EtOAc/hexanes). Brown oil, 55% yield (39.6 mg). ^1^H NMR (300 MHz, CDCl_3_): δ 8.88 (s, 1H), 7.97–7.90 (m, 2H), 7.52–7.41 (m, 2H), 7.35 (s, 1H), 7.29 (s, 1H), 6.77 (s, 1H), 3.03 (t, *J* = 7.0 Hz, 2H), 1.90–1.82 (m, 2H), 1.49–1.39 (m, 4H), 0.95 (t, *J* = 7.0 Hz, 3H). ^13^C{^1^H} NMR (75 MHz, CDCl_3_): δ 135.1 (C), 130.9 (C), 130.3 (C), 128.3 (CH), 124.6 (CH), 124.2 (C), 123.9 (CH), 121.8 (CH), 120.6 (C), 119.2 (CH), 118.7 (CH), 102.8 (CH), 33.8 (CH_2_), 32.0 (CH_2_), 29.9 (CH_2_), 22.7 (CH_2_), 14.1 (CH_3_). HRMS (ESI) calcd for C_17_H_20_N [M + H]^+^ 238.1596, found 238.1591.

#### 4-(Phenyl)-7-(trifluoromethyl)-1*H*-benzo[*g*]indole (**4h**)

Following the general procedure, compound **4h** was obtained from azide **3h** (83.0 mg, 0.340 mmol) and InCl_3_ (8.1 mg, 0.032 mmol) in toluene (5 mL) at 110 °C for 48 h followed by purification (3–5% EtOAc/hexanes). Brown oil, 42% yield (32.0 mg). ^1^H NMR (300 MHz, CDCl_3_): δ 9.03 (s, 1H), 8.26 (s, 1H), 8.07 (d, *J* = 8.0 Hz, 1H), 7.79 (d, *J* = 7.3 Hz, 2H), 7.70 (d, *J* = 7.8 Hz, 1H), 7.64 (s, 1H), 7.55 (t, *J* = 7.8 Hz, 2H), 7.47 (d, *J* = 7.4 Hz, 1H), 7.37 (t, *J* = 3.2 Hz, 1H), 6.89 (t, *J* = 3.1 Hz, 1H). ^13^C NMR (75 MHz, CDCl_3_) δ 140.4 (C), 136.0 (C), 129.6 (C), 128.8 (2 × CH), 128.7 (2 × CH), 127.7 (CH), 126.6 (q, ^3^*J*_CF_ = 4.6 Hz, CH), 126.0 (q, ^2^*J*_CF_ = 32.0 Hz, C),123.6 (CH), 123.3 (q, ^1^*J*_CF_ = 253.1 Hz, CF_3_), 122.3 (C), 121.2 (q, ^3^*J*_CF_ = 4.6 Hz, CH), 120.3 (CH), 120.1 (CH), 104.4 (CH). ^19^F NMR (282 MHz, CDCl_3_): δ – 61.60 (s, CF_3_). HRMS (ESI) calcd for C_19_H_13_F_3_N [M + H]^+^ 312.1000, found 312.0996.

## Data Availability

The data underlying this study are available in the published article and its Supporting Information.

## Supporting Information Available

Synthetic procedures, characterization data, and ^1^H and ^13^C NMR spectra for all compounds (PDF)
