# Supplementary material for: Indium(III)-Catalyzed Synthesis of Pyrroles and Benzo[g]indoles by Intramolecular Cyclization of Homopropargyl Azides
Source: J Org Chem. 2024 Oct 15;89(21):16015–21. doi: 10.1021/acs.joc.4c01768 (PMC11536385; doi:10.1021/acs.joc.4c01768)
Supplement: Supplementary file 1 — jo4c01768_si_001.pdf [file jo4c01768_si_001.pdf]

Supporting Information

Indium(III)-Catalyzed Synthesis of Pyrroles and Benzo[*g*]indoles by Intramolecular Cyclization of Homopropargyl Azides

Ana Da Lama, José Pérez Sestelo, Luis A. Sarandeses,* and M. Montserrat Martínez*

CICA – Centro Interdisciplinar de Química e Bioloxía and Departamento de Química, Universidade da Coruña, 15071 A Coruña, Spain

Table of Contents

	Page No.
1. Synthesis of homopropargyl azides and azido-diynes	S2
2. Detection of intermediate **5b** by ^{1}H NMR	S26
3. References	S27
4. Copies of ^{1}H NMR and ^{13}C NMR spectra	S28

1. Synthesis of homopropargyl azides and azido-diynes

General procedure for the synthesis of substituted but-3-yn-1-ols (S1-S6)

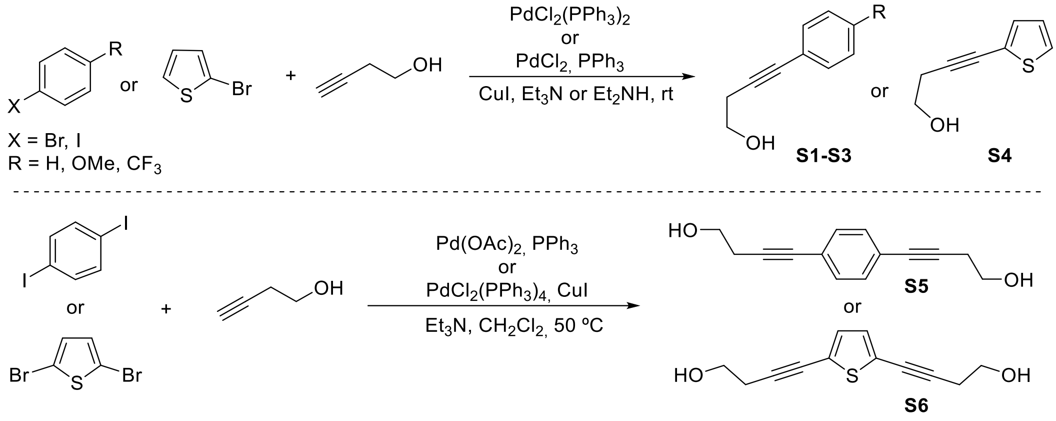

A mixture of but-3-yn-1-ol (1 equiv.) with the corresponding aromatic halide (1.1 equiv.), Pd catalyst (10-30 mol%), CuI (10-30 mol%) and a base (Et_3N or Et_2NH, 6-10 mL) was stirred at room temperature overnight. The reaction was then diluted with EtOAc (15 mL) and washed with water (2 x 15 mL). The organic phase was dried ($MgSO_4$ anhyd.), filtered and concentrated. The resulting crude was purified by flash column chromatography (EtOAc/hexanes) to afford, after concentration and high vacuum drying, the corresponding alcohol.

4-Phenylbut-3-yn-1-ol (S1)[1]

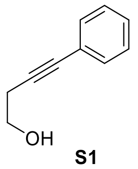

S1

Following the general procedure, the title compound **S1** was obtained from but-3-yn-1-ol (1.1 mL, 14.27 mmol) on reaction with iodobenzene (1.7 mL, 15.69 mmol), $PdCl_2(PPh_3)_2$ (100.3 mg, 0.143 mmol), CuI (27.5 mg, 0.143 mmol) and Et_3N (10 mL) at rt for 18 h, followed by purification (30-50% EtOAc/hexanes). Yellow oil, 97% yield (2.02 g). IR (ATR): ν 3430, 2880, 1683, 1449 cm^{-1}. ^1H NMR (300 MHz, $CDCl_3$): δ 7.47-7.43 (m, 2H), 7.33-7.29 (m, 3H), 3.82 (t, J = 6.5 Hz, 2H), 3.02 (s, 1H), 2.69 (t, J = 6.5 Hz, 2H). $^{13}C\{1H\}$ NMR (75 MHz, $CDCl_3$): δ 131.7 (2 x CH), 128.3 (2 x CH), 127.9 (CH), 123.5 (C), 86.7 (C), 82.3 (C), 61.1 (CH_2), 23.7 (CH_2). HRMS (ESI) calcd for $C_{10}H_{11}O$ $[M + H]^+$ 147.0765, found 147.0763.

4-(4-Methoxyphenyl)but-3-yn-1-ol (S2)[2]

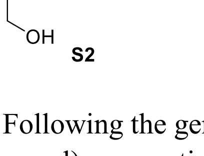

S2

Following the general procedure, compound **S2** was obtained from but-3-yn-1-ol (0.22 mL, 2.86 mmol) on reaction with 1-iodo-4-methoxybenzene (736.2 mg, 3.15 mmol), $PdCl_2(PPh_3)_2$ (60.3 mg, 0.086 mmol), CuI (16.5 mg, 0.086 mmol) and Et_3N (9 mL) at rt for 18 h, followed by purification (40% EtOAc/hexanes). Yellow oil, 89% yield (446.1 mg). IR (ATR): ν 3390, 2919,

1609, 1511 cm^{-1}. ^{1}H NMR (300 MHz, $CDCl_3$): δ 7.33 (d, *J* = 8.6 Hz, 2H), 6.80 (d, *J* = 7.3 Hz, 2H), 3.80-3.75 (m, 5H), 2.65 (t, *J* = 6.3 Hz, 2H), 2.40 (s, 1H). ^{13}C{1H} NMR (75 MHz, $CDCl_3$): δ 159.3 (C), 133.0 (2 x CH), 115.5 (C), 113.9 (2 x CH), 84.9 (C), 82.2 (C), 61.2 (CH_2), 55.3 (CH_3), 23.8 (CH_2). HRMS (ESI) calcd for $C_{11}H_{12}Na\ O_2$ $[M + Na]^+$ 199.0735, found 199.0730.

4-(4-(Trifluoromethyl)phenyl)but-3-yn-1-ol (S3)[3]

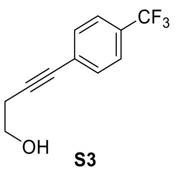

S3

Following the general procedure, compound **S3** was obtained from but-3-yn-1-ol (0.18 mL, 2.38 mmol) on reaction with 1-bromo-4-(trifluoromethyl)benzene (0.37 mL, 2.62 mmol), $PdCl_2$ (13.1 mg, 0.07 mmol), PPh_3 (37.6 mg, 0.14 mmol), CuI (27.7 mg, 0.14 mmol) and Et_2NH (6 mL) at rt for 17 h, followed by purification (25% EtOAc/hexanes). Yellow oil, 88% yield (447.9 mg). IR (ATR): ν 3370, 2932, 2234, 1324 cm^{-1}. ^{1}H NMR (300 MHz, $CDCl_3$): δ 7.54-7.46 (m, 4H), 3.82 (t, *J* = 6.1 Hz, 2H) 2.69 (t, *J* = 6.1 Hz, 2H), 2.51 (s, 1H). ^{13}C{1H} NMR (75 MHz, $CDCl_3$): δ 132.4 (2 x CH), 130.2 (q, $^{2}J_{CF}$ = 32.7 Hz, C), 127.8 (C), 125.7 (q, $^{3}J_{CF}$ = 3.8 Hz, 2 x CH), 124.4 (q, $^{1}J_{CF}$ = 271.6 Hz, CF_3), 89.9 (C), 81.6 (C), 61.5 (CH_2), 24.2 (CH_2). HRMS (ESI) calcd for $C_{11}H_9F_3O$ $[M]^+$ 214.0605, found 214.0610.

4-(Thiophen-2-yl)but-3-yn-1-ol (S4)[4]

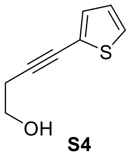

S4

Following the general procedure, the title compound **S4** was obtained from but-3-yn-1-ol (0.25 mL, 3.30 mmol) on reaction with 2-bromothiophene (0.35 mL, 3.63 mmol), $PdCl_2$ (17.6 mg, 0.10 mmol), PPh_3 (52.1 mg, 0.20 mmol), CuI (37.9 mg, 0.20 mmol) and Et_2NH (8 mL) at rt for 18 h, followed by purification (25% EtOAc/hexanes). Yellow oil, 83% yield (417.2 mg). IR (ATR): ν 3402, 2928, 1659, 1417 cm^{-1}. ^{1}H NMR (300 MHz, $CDCl_3$) δ 7.19 (d, *J* = 5.2 Hz, 1H), 7.15 (d, *J* = 3.4 Hz, 1H), 6.94 (t, *J* = 3.9 Hz, 1H), 3.80 (t, *J* = 6.2 Hz, 2H), 2.69 (t, *J* = 6.2 Hz, 2H), 2.20 (s, 1H). ^{13}C{1H} NMR (75 MHz, $CDCl_3$): δ 131.6 (CH), 126.9 (CH), 126.4 (CH), 123.4 (C), 90.6 (C), 75.5 (C), 61.0 (CH_2), 24.1 (CH_2). HRMS (ESI) calcd for C_8H_8NaOS $[M + Na]^+$ 175.0194, found 175.0188.

4,4'-(1,4-Phenylene)bis(but-3-yn-1-ol) (S5)[5]

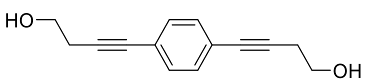

S5

Following the general procedure, compound **S5** was obtained from but-3-yn-1-ol (0.44 mL, 5.83 mmol) on reaction with 1,4-diiodobenzene (770.1 mg, 2.33 mmol), $Pd(OAc)_2$ (5.2 mg, 0.023 mmol), PPh_3 (12.2 mg, 0.47 mmol), CuI (8.9 mg, 0.047 mmol) and Et_3N/CH_2Cl_2 (1/3, 50 mL) at 50 ºC for 22 h, followed by purification (50-100% EtOAc/hexanes). White solid, 84% yield (420 mg). IR (ATR): ν 2921, 2852, 2365, 1359, 1179 cm^{-1}. (300 MHz, Methanol-d4): δ 7.32 (s, 4H), 3.73 (t, *J* = 6.7 Hz, 4H), 2.63 (t, *J* = 6.7 Hz, 4H). ^{13}C{1H} NMR (75 MHz, Methanol-d4): δ 131.0

(4 x CH), 123.2 (2 x C), 88.2 (2 x C), 80.6 (2 x C), 60.3 (2 x CH_2), 22.9 (2 x CH_2). HRMS (ESI) calcd for $C_{14}H_{14}O_2$ $[M]^+$ 214.0994, found 214.1003.

4,4'-(thiophene-2,5-diyl)bis(but-3-yn-1-ol) (S6)[5]

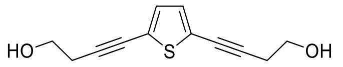

S6

Following the general procedure, compound **S6** was obtained from but-3-yn-1-ol (0.55 mL, 7.23 mmol) on reaction with 2,5-dibromothiophene (0.33 mL, 2.89 mmol), $PdCl_2(PPh_3)_2$ (20.3 mg, 0.03 mmol), CuI (11.0 mg, 0.06 mmol) and Et_3N/CH_2Cl_2 (1/3, 30 mL) at 50 ºC for 21 h, followed by purification (30-50% EtOAc/hexanes). White solid, 95% yield (670.6 mg). IR (ATR): ν 3313, 2920, 1454, 1042 cm^{-1}. ^1H NMR (300 MHz, $CDCl_3$): δ 6.96 (s, 2H), 3.79 (t, J = 6.3 Hz, 4H), 2.69 (t, J = 6.3 Hz, 4H), 2.58 (s, 2H). $^{13}C\{1H\}$ NMR (75 MHz, $CDCl_3$): δ 131.3 (2 x CH), 124.0 (2 x C), 91.5 (2 x C), 75.1 (2 x C), 60.8 (2 x CH_2), 24.0 (2 x CH_2). HRMS (ESI): calcd for $C_{12}H_{12}NaO_2S$ $[M + Na]^+$ 243.0451, found 243.0456.

Synthesis of 2-methyldec-5-yn-3-ol (S7)

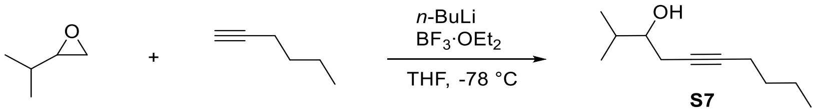

S7

Butyllithium (3.9 mL, 8.8 mmol) was added dropwise to a solution of hexyne (1.1 mL, 9.3 mmol) in dry THF (30 mL) at −78 °C under argon atmosphere. After stirring for 30 minutes at −78 °C, a solution of 2-isopropyloxirane (0.49 mL, 4.64 mmol) in THF (5 mL) and $BF_3{\cdot}Et_2O$ (1.2 mL, 9.3 mmol) were successively added and the resulting mixture was stirred at −78°C for 2 hours. The reaction was quenched with saturated aqueous $NaHCO_3$ solution (15 mL) and extracted with EtOAc (2 x 15 mL). The combined organic phase was dried ($MgSO_4$ anhyd.), filtered and concentrated. The resulting crude was purified by column chromatography (3-10% EtOAc/hexanes) to afford, after concentration and high vacuum drying, compound **S7** as a colorless oil in 88% yield (690.1 mg). IR (ATR): ν 3423, 2958, 2928, 1740, 1466 cm^{-1}. ^1H NMR (300 MHz, $CDCl_3$): δ 3.41-3.34 (m, 1H), 2.40-2.20 (m, 2H), 2.16-2.08 (m, 3H), 1.77-1.66 (m, 1H), 1.49-1.29 (m, 4H), 0.92-0.84 (m, 9H). $^{13}C\{1H\}$ NMR (75 MHz, $CDCl_3$): δ 83.0 (C), 76.3 (C), 75.0 (CH), 32.7 (CH), 31.1 (CH_2), 25.0 (CH_2), 21.9 (CH_2), 18.7 (CH_3), 18.4 (CH_2), 17.7 (CH_3), 13.5 (CH_3). HRMS (ESI) calcd for $C_{11}H_{20}NaO$ $[M + Na]^+$ 191.1412, found 191.1419.

General procedure for the synthesis of tosylates (S8-S13)

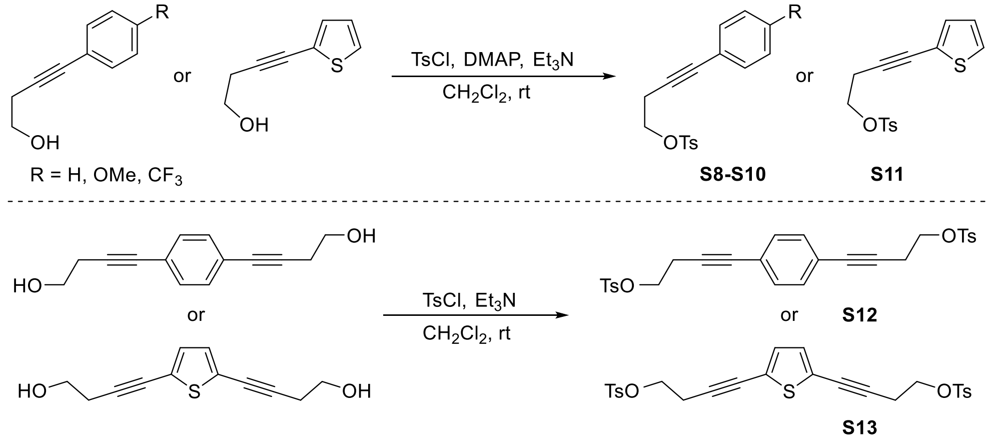

The alcohol was dissolved in CH_2Cl_2 (10-70 mL) and then Et_3N (1.2-1.6 equiv.) and DMAP (0.02-0.12 equiv.) were added. Then, TsCl was added in portions (1.2-1.5 equiv.) and the reaction was stirred at room temperature overnight. The reaction was quenched with saturated aqueous $NaHCO_3$ solution (15 mL) and extracted with EtOAc (2 x 15 mL). The combined organic phase was washed with brine (15 mL), dried ($MgSO_4$ anhyd.), filtered and concentrated. The resulting crude was purified by flash column chromatography (EtOAc/hexanes) to afford, after concentration and high vacuum drying, the corresponding tosylate.

4-Phenylbut-3-yn-1-yl 4-methylbenzenesulfonate (S8)[3]

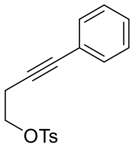

S8

Following the general procedure, compound **S8** was obtained from alcohol **S1** (2.02 g, 13.81 mmol) on reaction with TsCl (3.16 g, 16.58 mmol), Et_3N (2.31 mL, 16.58 mmol) and DMAP (33.8 mg, 0.276 mmol) in CH_2Cl_2 (70 mL) at rt for 23 h, followed by purification (20% EtOAc/hexanes). Yellow oil, 98% yield (4.06 g). IR (ATR): ν 3036, 2922, 1361, 1176 cm^{-1}. ^1H NMR (300 MHz, $CDCl_3$): δ 7.85-7.83 (m, 2H), 7.37-7.28 (m, 7H), 4.21 (t, J = 7.0 Hz, 2H), 2.80 (t, J = 7.0 Hz, 2H), 2.44 (s, 3H). $^{13}C\{1H\}$ NMR (75 MHz, $CDCl_3$): δ 144.9 (C), 132.9 (C), 131.6 (2 x CH), 129.9 (2 x CH), 128.2 (2 x CH), 128.2 (CH), 128.0 (2 x CH), 123.0 (C), 83.8 (C), 82.7 (C), 67.8 (CH_2), 21.6 (CH_3), 20.4 (CH_2). HRMS (ESI) calcd for $C_{17}H_{16}NaO_3S$ $[M + Na]^+$ 323.0718, found 323.0700.

4-(4-Methoxyphenyl)but-3-yn-1-yl 4-methylbenzenesulfonate (S9)[2]

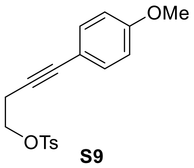

S9

Following the general procedure, comppund **S9** was obtained from alcohol **S2** (218 mg, 1.24 mmol) on reaction with TsCl (344.5 mg, 1.81 mmol), Et_3N (0.29 mL, 2.06 mmol) and DMAP (7.9 mg, 0.065 mmol) in CH_2Cl_2 (10 mL) at rt for 16 h, followed by purification (20% EtOAc/hexanes). Yellow oil, 96% yield (392 mg). IR (ATR): ν 2928, 1716, 1600, 1212, 1116 cm^{-1}. ^1H NMR (300 MHz, $CDCl_3$): δ 7.81 (d, J = 8.5 Hz, 2H), 7.29 (t, J = 8.8 Hz, 4H), 6.80 (d, J = 8.7 Hz, 2H), 4.17 (t, J = 7.0 Hz, 2H), 3.78 (s, 3H), 2.75 (t, J = 7.1 Hz, 2H), 2.41 (s, 3H). $^{13}C\{1H\}$ NMR (75 MHz, $CDCl_3$): δ 159.5 (C), 145.0 (C), 133.0 (2 x CH), 132.9 (C), 129.9 (2 x CH), 128.0 (2 x CH), 115.1 (C), 113.9 (2 x CH), 82.5 (C), 82.3 (C), 68.0 (CH_2), 55.3 (CH_3), 21.6 (CH_3), 20.4 (CH_2). HRMS (ESI) calcd for $C_{18}H_{18}NaO_4S$ $[M + Na]^+$ 353.0823, found 353.0820.

4-(4-(Trifluoromethyl)phenyl)but-3-yn-1-yl 4-methylbenzenesulfonate (S10)

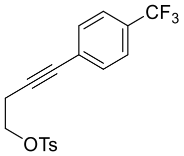

S10

Following the general procedure, compound **S10** was obtained from alcohol **S3** (447 mg, 2.09 mmol) on reaction with TsCl (596.8 mg, 3.13 mmol), Et_3N (0.44 mL, 3.13 mmol) and DMAP (30.8 mg, 0.25 mmol) in CH_2Cl_2 (20 mL) at rt for 16 h, followed by purification (20% EtOAc/hexanes). Yellow oil, 95% yield (729.5 mg). IR (ATR): ν 2920, 1324, 1176 cm^{-1}. ^{19}F NMR (282 MHz, $CDCl_3$): δ –62.84 (s, CF_3). ^1H NMR (300 MHz, $CDCl_3$): δ 7.81 (d, J = 8.2 Hz, 2H), 7.52 (d, J = 8.2 Hz, 2H), 7.42 (d, J = 8.2 Hz, 2H), 7.30 (d, J = 8.2 Hz, 2H), 4.19 (t, J = 6.7 Hz, 2H), 2.79 (t, J = 6.7 Hz, 2H), 2.39 (s, 3H). $^{13}C\{1H\}$ NMR (75 MHz, $CDCl_3$): δ 145.1 (C), 132.8 (2 x C), 131.9 (2 x CH), 129.9 (2 x CH), 127.9 (2 x CH), 126.9 (q, $^2J_{CF}$ = 36.6 Hz, C), 125.1 (q, $^3J_{CF}$ = 3.8 Hz, 2 x CH), 123.9 (q, $^1J_{CF}$ = 272.5 Hz, CF_3), 86.8 (C), 81.4 (C), 67.6 (CH_2), 21.6 (CH_3), 20.4 (CH_2). HRMS (ESI) calcd for $C_{18}H_{15}F_3NaO_3S$ $[M + Na]^+$ 391.0592, found 391.0587.

4-(Thiophen-2-yl)but-3-yn-1-yl 4-methylbenzenesulfonate (S11)[6]

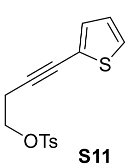

S11

Following the general procedure, compound **S11** was obtained from alcohol **S4** (417 mg, 2.74 mmol) on reaction with TsCl (783.5 mg, 4.11 mmol), Et_3N (0.57 mL, 4.11 mmol) and DMAP (40.3 mg, 0.329 mmol) in CH_2Cl_2 (20 mL) at rt for 16 h, followed by purification (20% EtOAc/hexanes). Yellow oil, 92% yield (772 mg). IR (ATR): ν 2926, 1600, 1362, 1178 cm^{-1}. ^1H NMR (300 MHz, $CDCl_3$): δ 7.79 (d, J = 8.1 Hz, 2H), 7.28 (d, J = 8.1 Hz, 2H), 7.17 (d, J = 5.2 Hz, 1H), 7.08 (d, J = 3.6 Hz, 1H), 6.92-6.89 (m, 1H), 4.14 (t, J = 6.9 Hz, 2H), 2.76 (t, J = 6.9 Hz, 2H), 2.38 (s, 3H). $^{13}C\{1H\}$ NMR (75 MHz, $CDCl_3$): δ 145.4 (C), 133.0 (C), 132.2 (CH), 130.3 (2 x CH), 128.2 (2 x CH), 127.2 (CH), 127.1 (CH), 123.3 (C), 88.5 (C), 76.2 (C), 67.9 (CH_2), 21.9 (CH_3), 20.9 (CH_2). HRMS (ESI) calcd for $C_{15}H_{14}Na\ O_3S_2$ $[M + Na]^+$ 329.0228, found 329.0229.

1,4-Phenylenebis(but-3-yne-4,1-diyl) bis(4-methylbenzenesulfonate) (S12)

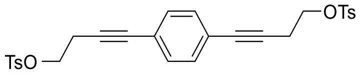

S12

Following the general procedure, compound **S12** was obtained from diol **S5** (223.2 mg, 1.04 mmol) on reaction with TsCl (595.9 mg, 3.12 mmol), Et_3N (0.43 mL, 3.12 mmol) in CH_2Cl_2 (10 mL) at rt for 48 h, followed by purification (10-100% EtOAc/hexanes). Yellow solid, 76% yield (413.6 mg). IR (ATR): ν 3035, 2923, 1492, 1137 cm^{-1}. ^1H NMR (300 MHz, $CDCl_3$): δ 7.81 (d, J = 8.3 Hz, 4H), 7.31 (d, J = 8.2 Hz, 4H), 7.24 (s, 4H), 4.18 (t, J = 7.1 Hz, 4H), 2.78 (t, J = 7.1 Hz, 4H), 2.42 (s, 6H). $^{13}C\{1H\}$ NMR (75 MHz, $CDCl_3$): δ 145.0 (2 x C), 132.9 (2 x C), 131.5 (4 x CH), 129.9 (4 x CH), 128.0 (4 x CH), 122.7 (2 x C), 85.7 (2 x C), 82.3 (2 x C), 67.7 (2 x CH_2), 21.7 (2 x CH_3), 20.5 (2 x CH_2). HRMS (ESI) calcd for $C_{28}H_{26}NaO_6S_2$ $[M + Na]^+$ 545.1068, found 545.1063.

Thiophene-2,5-diylbis(but-3-yne-4,1-diyl) bis(4-methylbenzenesulfonate) (S13)

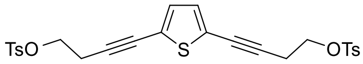

S13

Following the general procedure, compound **S13** was obtained from diol **S6** (615 mg, 2.79 mmol) on reaction with TsCl (1.6 g, 8.37 mmol), Et_3N (1.2 mL, 8.37 mmol) in CH_2Cl_2 (25 mL) at rt for 16 h, followed by purification (10-100% EtOAc/hexanes). Yellow solid, 88% yield (1.26 g). IR

(ATR): ν 3065, 2961, 1597, 1358, 1174 cm^{-1}. ^1H NMR (300 MHz, $CDCl_3$): δ 7.81 (d, *J* = 8.2 Hz, 4H), 7.33 (d, *J* = 8.2 Hz, 4H), 6.92 (s, 2H), 4.17 (t, *J* = 6.8 Hz, 4H), 2.79 (t, *J* = 6.8 Hz, 4H), 2.43 (s, 6H). $^{13}C\{1H\}$ NMR (75 MHz, $CDCl_3$): δ 145.1 (2 x C), 132.8 (2 x C), 131.5 (2 x CH), 130.0 (4 x CH), 127.9 (4 x CH), 123.8 (2 x C), 88.9 (2 x C), 75.4 (2 x C), 67.4 (2 x CH_2), 21.6 (2 x CH_3), 20.7 (2 x CH_2); HRMS (ESI): calcd for $C_{26}H_{24}NaO_6S_3$ $[M + Na]^+$ 551.0366, found 551.0365.

2-Methyldec-5-yn-3-yl 4-methylbenzenesulfonate (S14)

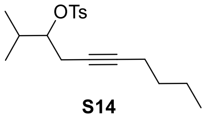

S14

Following the general procedure, compound **S14** was obtained from diol **S7** (605 mg, 3.60 mmol) on reaction with TsCl (190.6 mg, 7.19 mmol), Et_3N (0.75 mL, 15.4 mmol) and DMAP (263.6 mg, 2.16 mmol) in CH_2Cl_2 (25 mL) at rt for 48 h, followed by purification (3-5% EtOAc/hexanes). Yellow oil, 77% yield (892.3 mg). IR (ATR): ν 2958, 2926, 1739, 1368, 1178 cm^{-1}. ^1H NMR (300 MHz, $CDCl_3$): δ 7.80 (d, *J* = 8.2 Hz, 2H), 7.32 (d, *J* = 8.2 Hz, 2H), 4.40 (q, *J* = 5.6 Hz, 1H), 2.51-2.47 (m, 2H), 2.43 (s, 3H), 2.19-2.04 (m, 3H), 1.47-1.25 (m, 4H), 0.91-0.82 (m, 9H). $^{13}C\{1H\}$ NMR (75 MHz, $CDCl_3$): δ 144.5 (C), 134.4 (C), 129.7 (2 x CH), 127.8 (2 x CH), 85.7 (CH), 83.0 (C), 74.2 (C), 30.9 (CH_2), 30.4 (CH), 22.6 (CH_2), 21.9 (CH_2), 21.6 (CH_3), 18.5 (CH_3), 18.4 (CH_2), 16.6 (CH_3), 13.6 (CH_3). HRMS (ESI) calcd for $C_{18}H_{26}NaO_3S$ $[M + Na]^+$ 345.1500, found 345.1496.

General procedure for the synthesis of homopropargyl azides from tosylates (1a-1d, 1g-1i)

Substrates employed in the reaction:

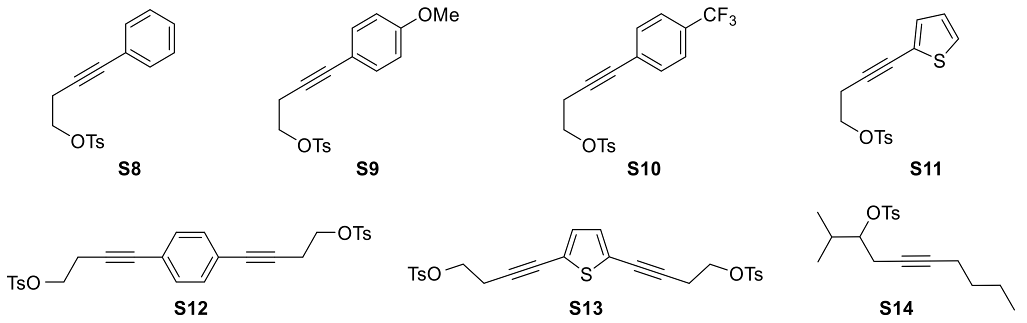

Sodium azide (1.1 equiv.) was added to a solution of tosylate compound (1 equiv.) in DMF (8-50 mL) and the mixture was stirred at room temperature until TLC showed that the reaction was completed. The reaction was quenched with H_2O (15 mL) and extracted with EtOAc (2 x 15 mL). The combined organic phase was washed with brine (15 mL), dried ($MgSO_4$ anhyd.), filtered and concentrated. The resulting crude was purified by flash column chromatography (EtOAc/hexanes) to afford, after concentration and high vacuum drying, the corresponding azide.

(4-Azidobut-1-yn-1-yl)benzene (1a)[7]

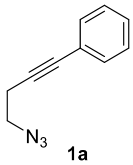

1a

Following the general procedure, compound **1a** was obtained from tosylate **S8** (2.3 g, 7.66 mmol) on reaction with NaN_3 (548.6 mg, 8.42 mmol) in DMF (50 mL) at rt for 19 h, followed by purification (5% EtOAc/hexanes). Colorless oil, 86% yield (1.1 g). IR (ATR): ν 2936, 2086, 1489, 1263 cm^{-1}. ^1H NMR (300 MHz, $CDCl_3$): δ 7.54-7.50 (m, 2H), 7.36-7.34 (m, 3H), 3.44 (t, J = 6.7 Hz, 2H), 2.71 (t, J = 6.7 Hz, 2H). $^{13}C\{1H\}$ NMR (75 MHz, $CDCl_3$): δ 131.5 (2 x CH), 128.2 (2 x CH), 127.9 (CH), 123.1 (C), 85.9 (C), 82.4 (C), 49.7 (CH_2), 20.4 (CH_2). HRMS (ESI) calcd for $C_{10}H_9N_3$ $[M]^+$ 171.0796, found 171.0790.

1-(4-Azidobut-1-yn-1-yl)-4-methoxybenzene (1b)[2]

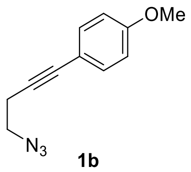

1b

Following the general procedure, compound **1b** was obtained from tosylate **S9** (334 mg, 1.01 mmol) on reaction with NaN_3 (72.4 mg, 1.11 mmol) in DMF (8 mL) at rt for 19 h, followed by purification (5-10% EtOAc/hexanes). Colorless oil, 92% yield (187.6 mg). IR (ATR): ν 2935, 2089, 1509, 1245 cm^{-1}. ^1H NMR (300 MHz, $CDCl_3$): δ 7.35 (d, J = 8.5 Hz, 2H), 6.82 (d, J = 8.5 Hz, 2H), 3.79 (s, 3H), 3.45 (t, J = 6.7 Hz, 2H), 2.69 (t, J = 6.7 Hz, 2H). $^{13}C\{1H\}$ NMR (75 MHz, $CDCl_3$): δ 159.4 (C), 133.0 (2 x CH), 115.3 (C), 113.9 (2 x CH), 84.3 (C), 82.4 (C), 55.3 (CH_3), 50.1 (CH_2), 20.6 (CH_2). HRMS (ESI) calcd for $C_{11}H_{11}N_3O$ $[M]^+$ 201.0902, found 201.0910.

1-(4-Azidobut-1-yn-1-yl)-4-(trifluoromethyl)benzene (1c)

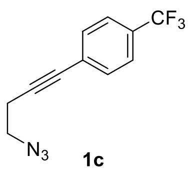

1c

Following the general procedure, compound **1c** was obtained from tosylate **S10** (579.5 mg, 1.57 mmol) on reaction with NaN_3 (112.7 mg, 1.73 mmol) in DMF (12 mL) at rt for 17 h, followed by purification (5% EtOAc/hexanes). Colorless oil, 87% yield (327 mg). IR (ATR): ν 2938, 2092, 1322, 1125 cm^{-1}. ^1H NMR (300 MHz, $CDCl_3$): δ 7.57-7.46 (m, 4H), 3.49 (t, J = 6.7 Hz, 2H), 2.73 (t, J = 6.7 Hz, 2H). ^{13}C NMR (75 MHz, $CDCl_3$): δ 131.9 (2 x CH), 129.8 (q, $^2J_{CF}$ = 31.9 Hz, C), 127.0 (C), 125.2 (q, $^3J_{CF}$ = 3.9 Hz, 2 x CH), 123.9 (q, $^1J_{CF}$ = 271.7 Hz, CF_3), 88.6 (C), 81.4 (C), 49.7 (CH_2), 20.7 (CH_2). ^{19}F NMR (282 MHz, $CDCl_3$): δ –62.83 (s, CF_3). HRMS (ESI) calcd for $C_{11}H_8F_3N_3$ $[M]^+$ 239.0670, found 239.0670.

2-(4-Azidobut-1-yn-1-yl)thiophene (1d)

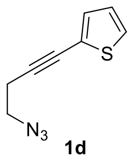

1d

Following the general procedure, compound **1d** was obtained from tosylate **S11** (770 mg, 2.51 mmol) on reaction with NaN_3 (199.5 mg, 3.07 mmol) in DMF (20 mL) at rt for 19 h, followed by purification (5% EtOAc/hexanes). Colorless oil, 89% yield (396 mg). IR (ATR): ν 3106, 2935, 2104, 1274 cm^{-1}. ^1H NMR (300 MHz, $CDCl_3$): δ 7.22- 7.17 (m, 2H), 6.97-6.94 (m, 1H), 3.47 (t, J = 6.8 Hz, 2H), 2.73 (t, J = 6.8 Hz, 2H). ^{13}C NMR (75 MHz, $CDCl_3$): δ 131.7 (CH), 126.9 (CH), 126.7 (CH), 123.2 (C), 89.9 (C), 75.9 (C), 49.8 (CH_2), 20.8 (CH_2). HRMS (ESI) calcd for $C_8H_7N_3S$ $[M]^+$ 177.0361, found 177.0370.

3-Azido-2-methyldec-5-yne (1g)

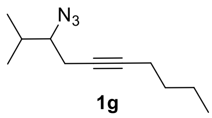

1g

Following the general procedure, compound **1g** was obtained from tosylate **S14** (351.5 mg, 1.09 mmol) on reaction with NaN_3 (141.7 mg, 2.18 mmol) in DMF (10 mL) at rt for 2 h, followed by purification (100% hexanes). Yellow oil, 76% yield (160 mg). IR (ATR): ν 2923, 2870, 2360, 2099, 1463 cm^{-1}. ^1H NMR (300 MHz, $CDCl_3$): δ 3.25 (q, J = 6.4 Hz, 1H), 2.44-2.40 (m, 2H), 2.19-2.13 (m, 2H), 1.96-1.85 (m, 1H), 1.53-1.33 (m, 4H), 0.98-0.85 (m, 9H). $^{13}C\{1H\}$ NMR (75 MHz, $CDCl_3$): δ 83.0 (C), 75.7 (C), 67.8 (CH), 31.6 (CH), 30.9 (CH_2), 22.9 (CH_2), 21.9 (CH_2), 19.6 (CH_3), 18.4 (CH_2), 17.7 (CH_3), 13.6 (CH_3). HRMS (ESI) calcd for $C_{11}H_{19}N_3Na$ $[M + Na]^+$ 216.1477, found 216.1488.

1,4-Bis(4-azidobut-1-yn-1-yl)benzene (1h)

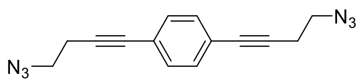

1h

Following the general procedure, compound **1h** was obtained from tosylate **S12** (302.7 mg, 0.58 mmol) on reaction with NaN_3 (113.1 mg, 1.74 mmol) in DMF (12 mL) at rt for 18 h, followed by purification (5% EtOAc/hexanes). Colorless solid, 88% yield (134.3 mg). IR (ATR): ν 2927, 2096, 1288 cm^{-1}. ^1H NMR (300 MHz, $CDCl_3$): δ 7.33 (s, 4H), 3.46 (t, J = 6.9 Hz, 4H), 2.71 (t, J = 6.9 Hz, 4H). $^{13}C\{1H\}$ NMR (75 MHz, $CDCl_3$): δ 131.5 (4 x CH), 122.9 (2 x C), 87.6 (2 x C), 82.2 (2 x C), 49.9 (2 x CH_2), 20.7 (2 x CH_2); HRMS (ESI) calcd for $C_{14}H_{12}N_6$ $[M + Na]^+$ 287.1021, found 287.1017.

2,5-Bis(4-azidobut-1-yn-1-yl)thiophene (1i)

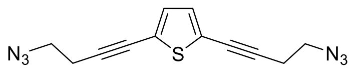

1i

Following the general procedure, compound **1i** was obtained from tosylate **S13** (1.3 g, 2.49 mmol) on reaction with NaN_3 (485.1 mg, 7.46 mmol) in DMF (25 mL) at rt for 22 h, followed by purification (5-10% EtOAc/hexanes). Yellow oil, 95% yield (638.3 mg). IR (ATR): ν 2924, 2104,

1523, 1271 cm^{-1}. ^{1}H NMR (300 MHz, $CDCl_3$): δ 7.00 (s, 2H), 3.47 (t, J = 6.8 Hz, 4H), 2.72 (t, J = 6.8 Hz, 4H). $^{13}C\{1H\}$ NMR (75 MHz, $CDCl_3$): δ 131.5 (2 x CH), 124.0 (2 x C), 90.7 (2 x C), 75.5 (2 x C), 49.6 (2 x CH_2), 20.8 (2 x CH_2). HRMS (ESI): calcd for $C_{12}H_{10}N_6NaS$ $[M + Na]^+$ 293.0585, found 293.0580.

1-Phenylbut-3-yn-1-ol (S15)[8]

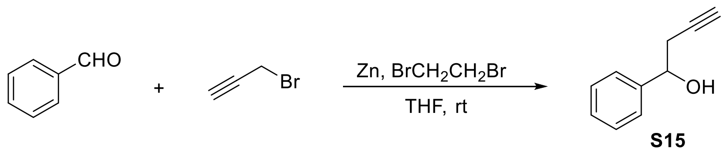

To a solution of benzaldehyde (1.44 mL, 14.13 mmol) in THF (70 mL), propargyl bromide (3.1 mL, 21.20 mmol), Zn dust (4.6 mg, 70.65 mmol) and dibromomethane (1.2 mL, 14.13 mmol) were added successively. The resulting suspension was stirred at room temperature for 21 hours. The reaction was passed through a celite pad with Et_2O. The filtrate was concentrated under reduced pressure. The resulting crude was purified by column chromatography (10% EtOAc/hexanes) to afford, after concentration and high vacuum drying, compound **S15** as a colorless oil in 69% yield (1.03 g). ^{1}H NMR (300 MHz, $CDCl_3$): δ 7.41-7.30 (m, 5H), 4.90-4.85 (m, 1H), 2.72-2.71 (m, 1H), 2.67-2.64 (m, 2H), 2.10 (s, 1H). $^{13}C\{1H\}$ NMR (75 MHz, $CDCl_3$): δ 142.5 (C), 128.5 (2 x CH), 128.0 (CH), 125.8 (2 x CH), 80.8 (C), 72.4 (CH), 71.0 (CH), 29.4 (CH_2). HRMS (ESI) calcd for $C_{10}H_{10}NaO$ $[M + Na]^+$ 169.0629, found 169.0625.

1,4-Diphenylbut-3-yn-1-ol (S16)[9]

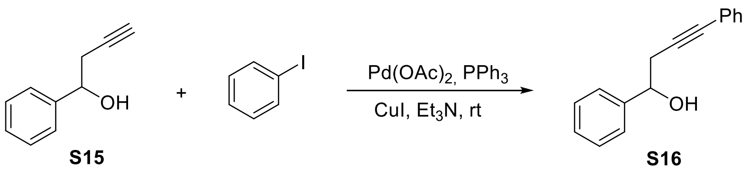

A mixture of compound **S15** (342.6 mg, 2.34 mmol) with iodobenzene (0.29 mL, 2.58 mmol), $Pd(OAc)_2$ (105.5 mg, 0.0.47 mmol), PPh_3 (245.5 mg, 0.94 mmol), CuI (89.2 mg, 0.47 mmol) and Et_3N (15 mL) was stirred at room temperature for 16 h. The reaction was then diluted with EtOAc (15 mL) and washed with brine (2 x 15 mL). The organic phase was dried ($MgSO_4$ anhyd.), filtered and concentrated. The resulting crude was purified by column chromatography (10-20% EtOAc/hexanes) to afford, after concentration and high vacuum drying, compound **S16** as a yellow paste in 88% yield (460.6 mg). IR (ATR): ν 3412, 2923, 2852, 2361, 1681, 1449 cm^{-1}. ^{1}H NMR (300 MHz, $CDCl_3$): δ 7.47-7.28 (m, 10H), 4.99-4.94 (m, 1H), 2.88 (d, J = 6.4 Hz, 2H), 2.55 (s, 1H). $^{13}C\{1H\}$ NMR (75 MHz, $CDCl_3$): δ 142.7 (C), 131.7 (2 x CH), 128.5 (2 x CH), 128.3 (2 x CH), 128.0 (CH), 128.0 (CH), 125.9 (2 x CH), 123.3 (C), 86.0 (C), 83.3 (C), 72.6 (CH), 30.6 (CH_2). HRMS (ESI) calcd for $C_{16}H_{14}NaO$ $[M + Na]^+$ 245.0942, found 245.0937.

(4-Azidobut-1-yne-1,4-diyl)dibenzene (1e)[7]

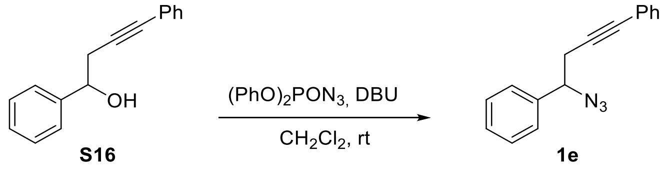

To a solution of **S16** (138.8 mg, 0.62 mmol) in CH_2Cl_2 (3 mL), 1,8-diazabiciclo[5.4.0]undec-7-eno (0.11 mL, 0.81 mmol) and diphenylphosphoryl azide (0.16 mL, 0.75 mmol) were added

successively. The resulting mixture was stirred at rt for 22 hours. The reaction was quenched with saturated aqueous NH_4Cl solution (15 mL) and extracted with EtOAc (2 x 15 mL). The organic phase was dried ($MgSO_4$ anhyd.), filtered and concentrated. The resulting crude was purified by column chromatography (2% EtOAc/hexanes) to afford, after concentration and high vacuum drying, compound **1e** as a colorless oil in 66% yield (101.5 mg). IR (ATR): ν 3051, 2923, 2329, 2102, 1493, 1249 cm^{-1}. ^1H NMR (300 MHz, $CDCl_3$): δ 7.41-7.35 (m, 7H), 7.30-7.27 (m, 3H), 4.75 (t, J = 6.9 Hz, 1H), 2.89 (t, J = 7.0 Hz, 1H), 2.97-2.81 (m, 1H). $^{13}C\{1H\}$ NMR (75 MHz, $CDCl_3$): δ 138.6 (C), 131.7 (2 x CH), 128.8 (2 x CH), 128.6 (CH), 128.3 (2 x CH), 128.1 (CH), 126.9 (2 x CH), 123.2 (C), 85.5 (C), 83.3 (C), 64.8 (CH), 28.1 (CH_2). HRMS (ESI) calcd for $C_{16}H_{13}N_3$ $[M]^+$ 247.1109, found 247.1114.

2-(Phenylethynyl)cyclohexan-1-ol (S17)[7]

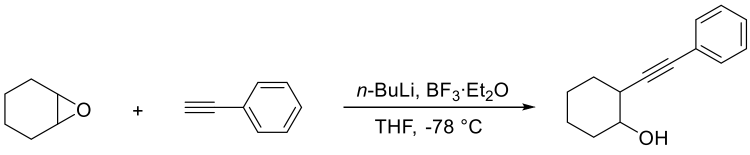

Butyllithium (2 mL, 4.5 mmol) was added dropwise to a solution of phenylacetylene (0.5 mL, 4.5 mmol) in dry THF (30 mL) at −78 °C under argon atmosphere and the mixture was stirred for 30 minutes. Then 1,2-epoxycyclohexane (0.3 mL, 3.0 mmol) and $BF_3\cdot Et_2O$ (0.57 mL, 4.5 mmol) were successively added and the resulting mixture was stirred for 4h at −78 °C. The reaction was quenched with saturated aqueous NH_4Cl solution (15 mL) and extracted with EtOAc (2 x 15 mL). The combined organic phase was washed with brine (15 mL), dried ($MgSO_4$ anhyd.), filtered and concentrated. The resulting crude was purified by column chromatography (20% EtOAc/hexanes) to afford, after concentration and high vacuum drying, compound **S17** as a colorless oil in 95% yield (570.3 mg). IR (ATR): ν 3360, 2930, 2856, 1490, 1444 cm^{-1}. ^1H NMR (300 MHz, $CDCl_3$): δ 7.32-7.30 (m, 2H), 7.18-7.16 (m, 3H), 3.48-3.41 (m, 1H), 2.57 (s, 1H), 2.37-2.31 (m, 1H), 1.99-1.91 (m, 2H), 1.68-1.53 (m, 2H), 1.42-1.25 (m, 1H), 1.23-1.07 (m, 3H). $^{13}C\{1H\}$ NMR (75 MHz, $CDCl_3$): δ 131.7 (2 x CH), 128.2 (2 x CH), 127.9 (CH), 123.4 (C), 91.1 (C), 82.6 (C), 73.4 (CH), 39.5 (CH), 33.2 (CH_2), 31.1 (CH_2), 24.8 (CH_2), 24.2 (CH_2). HRMS (ESI) calcd for $C_{14}H_{16}NaO$ $[M + Na]^+$ 223.1099, found 223.1093.

((A-azidocyclohexyl)ethynyl)benzene (1f)[7]

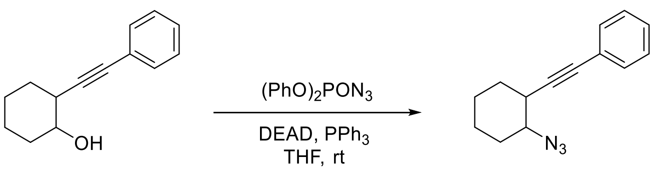

Diethyl azodicarboxylate (1.9 mL, 4.28 mmol, 40% solution in toluene) was added to a solution of PPh_3 (1.1 g, 4.28 mmol) in dry THF (15 mL) at 0 °C. The mixture was stirred 30 minutes and then it was added to a solution of **S17** in THF (8 mL) at 0°C. After being stirred for another 30 minutes at 0°C, diphenylphosphoryl azide (0.9 mL, 4.28 mmol) was added and the resulting mixture was stirred at rt for 20 hours. The solvent was removed under reduced pressure and the resulting crude was purified by column chromatography (1% EtOAc/hexanes) to afford, after concentration and high vacuum drying, compound **1f** as a yellow oil in 77% yield (493.7 mg). IR (ATR): ν 2935, 2168, 2091, 1488, 1299 cm^{-1}. ^1H NMR (300 MHz, $CDCl_3$): δ 7.48-7.45 (m, 2H), 7.32-7.27 (m, 3H), 3.54-3.49 (m, 1H), 3.11-3.07 (m, 1H), 2.00-1.97 (m, 2H), 1.80-1.59 (m, 4H), 1.51-1.41 (m, 2H). $^{13}C\{1H\}$ NMR (75 MHz, $CDCl_3$): δ 131.7 (2 x CH), 128.2 (2 x CH), 127.9

(CH), 123.5 (C), 89.1 (C), 84.0 (C), 61.1 (CH), 34.7 (CH), 29.7 (CH_2), 28.0 (CH_2), 23.2 (CH_2), 22.1 (CH_2). HRMS (ESI) calcd for $C_{14}H_{15}N_3Na$ $[M]^+$ 248.1164, found 248.1159.

General procedure I for the Sonogashira cross-coupling reaction on the iodine atom (S18-S25)

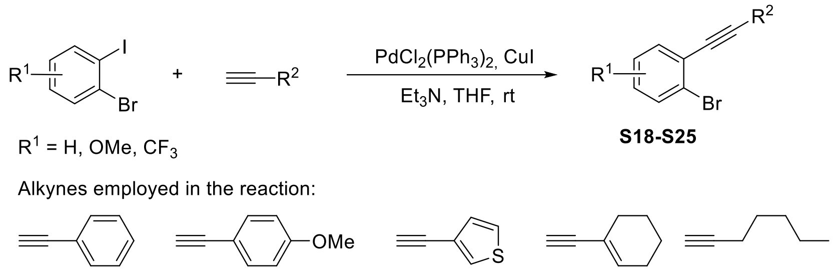

A mixture of 2-bromo-1-iodo-4-substituted-benzene (1 equiv.) with the corresponding alkyne (1.1 equiv.), $PdCl_2(PPh_3)_2$ (2.5-5 mol%), CuI (3-5 mol%), Et_3N (4-10 mL) and THF (4-7 mL) was stirred at room temperature until the starting material was consumed. The reaction was quenched with saturated aqueous NH_4Cl solution (15 mL) and extracted with EtOAc (2 x 15 mL). The combined organic phase was washed with water (15 mL) and brine (15 mL), dried ($MgSO_4$ anhyd.), filtered and concentrated. The resulting crude was purified by column chromatography (EtOAc/hexanes) to afford, after concentration and high vacuum drying, the corresponding compound.

1-Bromo-2-(phenylethynyl)benzene (S18)[10]

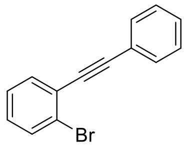

S18

Following the general procedure I, compound **S18** was obtained from 1-bromo-2-iodobenzene (0.45 mL, 3.53 mmol) on reaction with phenyl acetylene (0.43 mL, 3.88 mmol), $PdCl_2(PPh_3)_2$ (123.9 mg, 0.176 mmol), CuI (33.6 mg, 0.176 mmol), Et_3N (7 mL) and THF (7 mL) at rt for 18 h, followed by purification (4% EtOAc/hexanes). Yellow oil, 98% yield (890.7 mg). IR (ATR): ν 2920, 2852, 1736, 1492, 1465 cm^{-1}. ^1H NMR (300 MHz, $CDCl_3$): δ 7.68-7.59 (m, 4H), 7.43-7.39 (s, 3H), 7.36-7.30 (m, 1H), 7.24-7.19 (m, 1H). $^{13}C\{1H\}$ NMR (75 MHz, $CDCl_3$): δ 133.3 (CH), 132.5 (CH), 131.8 (2 x CH), 129.4 (CH), 128.7 (CH), 128.5 (2 x CH), 127.1 (CH), 125.7 (C), 125.5 (C), 123.0 (C), 94.0 (C), 88.1 (C).

2-Bromo-4-methyl-1-(phenylethynyl)benzene (S19)[11]

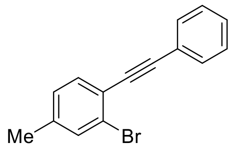

S19

Following the general procedure I, compound **S19** was obtained from 2-bromo-1-iodo-4-methylbenzene (0.48 mL, 3.37 mmol) on reaction with phenyl acetylene (0.41 mL, 3.71 mmol), $PdCl_2(PPh_3)_2$ (59.9 mg, 0.084 mmol), CuI (32.2 mg, 0.168 mmol) and Et_3N (10 mL) at rt for 3 h, followed by purification (3% EtOAc/hexanes). Colorless oil, 99% yield (904.0 mg). IR (ATR): ν

3063, 2921, 1571, 1499 cm^{-1}. ^{1}H NMR (300 MHz, $CDCl_3$): δ 7.64-7.59 (m, 2H), 7.50-7.47 (m, 2H), 7.42-7.38 (m, 3H), 7.13 (d, J = 7.9 Hz, 1H), 2.38 (s, 3H). ^{13}C{1H} NMR (75 MHz, $CDCl_3$): δ 140.1 (C), 133.0 (CH), 133.0 (CH), 131.7 (2 x CH), 128.5 (CH), 128.4 (2 x CH), 128.0 (CH), 125.4 (C), 123.2 (C), 122.4 (C), 93.2 (C), 88.2 (C), 21.2 (CH_3).

2-Bromo-1-(phenylethynyl)-4-(trifluoromethyl)benzene (S20)[11]

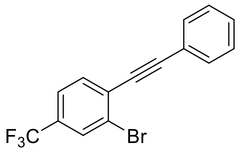

S20

Following the general procedure I, compound **S20** was obtained from 2-bromo-1-iodo-4-(trifluoromethyl)benzene (1 g, 2.85 mmol) on reaction with phenyl acetylene (0.34 mL, 3.13 mmol), $PdCl_2(PPh_3)_2$ (50.0 mg, 0.071 mmol), CuI (27.1 mg, 0.14 mmol) and Et_3N (10 mL) at rt for 3 h, followed by purification (1% EtOAc/hexanes). Colorless oil, 97% yield (898.5 mg). IR (ATR): ν 2927, 2853, 2197, 1609, 1323 cm^{-1}. ^{1}H NMR (300 MHz, $CDCl_3$): δ 7.89 (s, 1H), 7.67-7.53 (m, 4H), 7.42-7.37 (m, 3H). ^{13}C{1H} NMR (75 MHz, $CDCl_3$): δ 133.3 (CH), 132.5 (C), 131.9 (2 x CH), 131.0 (q, $^{2}J_{CF}$ = 33.5 Hz, C), 129.4 (q, $^{3}J_{CF}$ = 3.9 Hz, CH), 129.3 (CH), 128.5 (2 x CH), 125.8 (C), 123.9 (q, $^{3}J_{CF}$= 3.7 Hz, CH), 123.0 (q, $^{1}J_{CF}$ = 272.9 Hz, CF_3), 122.3 (C), 96.5 (C), 87.0 (C).

1-Bromo-2-((4-methoxyphenyl)ethynyl)benzene (S21)[11]

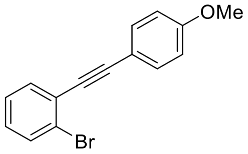

S21

Following the general procedure I, compound **S21** was obtained from 1-bromo-2-iodobenzene (0.31 mL, 2.44 mmol) on reaction with 1-ethynyl-4-methoxybenzene (354.7 mg, 2.68 mmol), $PdCl_2(PPh_3)_2$ (42.8 mg, 0.061 mmol), CuI (23.2 mg, 0.122 mmol), Et_3N (4 mL) and THF (4 ml) at rt for 18 h, followed by purification (7% EtOAc/hexanes). Colorless oil, 95% yield (664.3 mg). IR (ATR): ν 2921, 2852, 2193, 1513, 1251 cm^{-1}. ^{1}H NMR (300 MHz, $CDCl_3$): δ 7.51 (d, J = 8.0 Hz, 1H), 7.45-7.41 (m, 3H), 7.18 (t, J = 7.8 Hz, 1H), 7.08-7.02 (m, 1H), 6.79 (d, J = 8.4 Hz, 2H), 3.73 (s, 3H). ^{13}C{1H} NMR (75 MHz, $CDCl_3$): δ 160.0 (C), 133.2 (2 x CH), 133.1 (CH), 132.4 (CH), 129.0 (CH), 127.0 (CH), 125.8 (C), 125.5 (C), 115.1 (C), 114.1 (2 x CH), 94.2 (C), 86.9 (C), 55.3 (CH_3).

3-((2-Bromophenyl)ethynyl)thiophene (S22)[12]

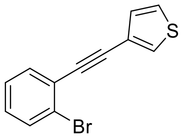

S22

Following the general procedure I, compound **S22** was obtained from 1-bromo-2-iodobenzene (0.36 mL, 2.83 mmol) on reaction with 3-ethynylthiophene (0.31 mL, 3.11 mmol), $PdCl_2(PPh_3)_2$ (59.5 mg, 0.085 mmol), CuI (16.2 mg, 0.085 mmol) and Et_3N (10 mL) at rt for 16 h, followed by

purification (1-2% EtOAc/hexanes). Colorless oil, 96% yield (706.8 mg). IR (ATR): ν 2919, 1850, 1736, 1434 cm^{-1}. ^1H NMR (300 MHz, $CDCl_3$): δ 7.66-7.56 (m, 3H), 7.38-7.27 (m, 3H), 7.23-7.17 (m, 1H). $^{13}C\{1H\}$ NMR (75 MHz, $CDCl_3$): δ 133.2 (CH), 132.5 (CH), 131.3 (C), 130.2 (C), 129.9 (CH), 129.4 (CH), 129.3 (CH), 127.1 (CH), 125.5 (CH), 122.0 (C), 89.2 (C), 87.6 (C).

1-Bromo-2-(cyclohex-1-en-1-ylethynyl)benzene (S23)[13]

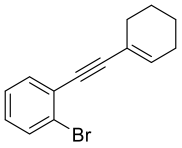

S23

Following the general procedure I, compound **S23** was obtained from 1-bromo-2-iodobenzene (0.36 mL, 2.83 mmol) on reaction with 1-ethynylcyclohex-1-ene (0.37 mL, 3.11 mmol), $PdCl_2(PPh_3)_2$ (59.9 mg, 0.085 mmol), CuI (16.2 mg, 0.085 mmol) and Et_3N (10 mL) at rt for 16 h, followed by purification (1% EtOAc/hexanes). Colorless oil, 95% yield (701.1 mg). IR (ATR): ν 2953, 2924, 1434, 1377 cm^{-1}. ^1H NMR (300 MHz, $CDCl_3$): δ 7.59 (d, *J* = 8.0 Hz, 1H), 7.47 (d, *J* = 7.7 Hz, 1H), 7.26 (t, *J* = 7.6 Hz, 1H), 7.14 (t, *J* = 7.7 Hz, 1H), 6.34-6.30 (m, 1H), 2.33-2.27 (m, 2H), 2.21-2.14 (m, 2H), 1.76-1.64 (m, 4H). $^{13}C\{1H\}$ NMR (75 MHz, $CDCl_3$): δ 136.1 (CH), 133.0 (CH), 132.3 (CH), 128.8 (CH), 126.9 (CH), 125.9 (C), 125.4 (C), 120.7 (C), 96.0 (C), 85.6 (C), 29.0 (CH_2), 25.8 (CH_2), 22.3 (CH_2), 21.5 (CH_2).

1-Bromo-2-(Hept-1-yn-1-yl)benzene (S24)[14]

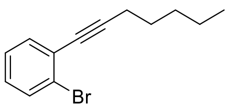

S24

Following the general procedure I, compound **S24** was obtained from 1-bromo-2-iodobenzene (0.45 mL, 3.53 mmol) on reaction with 1-heptyne (0.52 mL, 3.90 mmol), $PdCl_2(PPh_3)_2$ (123.9 mg, 0.176 mmol), CuI (33.6 mg, 0.176 mmol), Et_3N (7 mL) and THF (7 mL) at rt for 19 h, followed by purification (hexanes). Yellow oil, 95% yield (846 mg). ^1H NMR (300 MHz, $CDCl_3$): δ 7.56 (d, *J* = 8.1 Hz, 1H), 7.43 (d, *J* = 7.9 Hz, 1H), 7.22 (t, *J* = 7.7 Hz, 1H), 7.11 (t, *J* = 7.8 Hz, 1H), 2.46 (t, *J* = 7.2 Hz, 2H), 1.68-1.60 (m, 2H), 1.54-1.46 (m, 2H), 1.41-1.34 (m, 2H), 0.93 (t, *J* = 7.2 Hz, 3H). $^{13}C\{1H\}$ NMR (75 MHz, $CDCl_3$): δ 133.3 (CH), 132.3 (CH), 128.6 (CH), 126.9 (CH), 126.1 (C), 125.5 (C), 95.7 (C), 79.4 (C), 31.1 (CH_2), 28.3 (CH_2), 22.2 (CH_2), 19.6 (CH_2), 14.0 (CH_3).

1-Bromo-2-(phenylethynyl)-4-(trifluoromethyl)benzene (S25)[15]

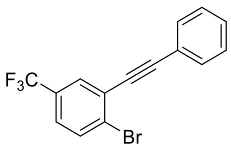

S25

Following the general procedure I, compound **S25** was obtained from 1-bromo-2-iodo-4-(trifluoromethyl)benzene (0.45 mL, 2.85 mmol) on reaction with phenyl acetylene (0.34 mL, 3.13 mmol), $PdCl_2(PPh_3)_2$ (100.0 mg, 0.14 mmol), CuI (26.7 mg, 0.14 mmol), Et_3N (7 mL) and TFH (7 mL) at rt for 19 h, followed by purification (hexanes). Yellow oil, 95% yield (769.5 g). ^1H NMR (300 MHz, $CDCl_3$): δ 7.82 (s, 1H), 7.74 (d, *J* = 8.8 Hz, 1H), 7.64-7.61 (m, 2H), 7.42-7.39

(m, 4H). $^{13}C\{1H\}$ NMR (75 MHz, $CDCl_3$): δ 133.1 (CH), 131.9 (2 x CH), 129.9 (q, $^3J_{CF}$ = 4.3 Hz, CH), 129.9 (q, $^2J_{CF}$ = 33.3 Hz, C), 129.4 (C), 129.2 (CH), 128.5 (2 x CH), 126.5 (C), 125.6 (q, $^3J_{CF}$ = 4.3 Hz, CH), 123.5 (q, $^1J_{CF}$ = 276.4 Hz, CF_3), 122.3 (C), 95.6 (C), 86.8 (C). ^{19}F NMR (282 MHz, CDCl3): δ –62.88 (s, CF_3).

General procedure II for the Sonogashira cross-coupling reaction on the bromine atom (S26-S33)

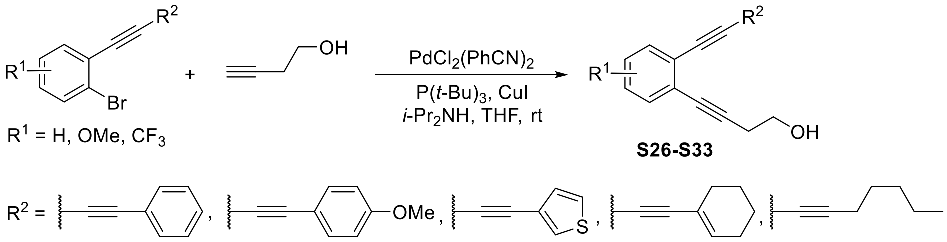

A mixture of 2-bromo-1,4-disubstituted-benzene (1 equiv.) with but-3-yn-1-ol (1.2 equiv.), $PdCl_2(PhCN)_2$ (6 mol%), CuI (6 mol%), P(*t*-Bu)$_3$ (12 mol%), *i*-Pr_2NH (5 equiv.) and THF (5-7 mL) was stirred at room temperature until TLC showed that the reaction was completed. The reaction was quenched with saturated aqueous NH_4Cl solution (15 mL) and extracted with EtOAc (2 x 15 mL). The combined organic phase was washed with brine (15 mL), dried ($MgSO_4$ anhyd.), filtered and concentrated. The resulting crude was purified by flash column chromatography (EtOAc/hexanes) to afford, after concentration and high vacuum drying, the corresponding compound.

4-(2-(Phenylethynyl)phenyl)but-3-yn-1-ol (S26)[16]

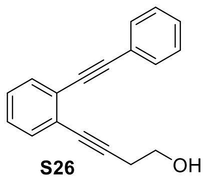

Following the general procedure II, compound **S26** was obtained from 1-bromo-2-(phenylethynyl)benzene (1.0 g, 3.89 mmol) on reaction with but-3-yn-1-ol (0.35 mL, 4.67 mmol), $PdCl_2(PhCN)_2$ (89.5 mg, 0.23 mmol), CuI (44.5 mg, 0.23 mmol), P(*t*-Bu)$_3$ (94.4 mg, 0.47 mmol), *i*-Pr_2NH (2.7 mL, 19.5 mmol) and THF (7 mL) at rt for 17 h, followed by purification (15-30% EtOAc/hexanes). Yellow oil, 78% yield (742.8 mg). IR (ATR): ν 3397, 2923, 2852, 1475 cm^{-1}. ^1H NMR (300 MHz, $CDCl_3$) δ 7.62-7.55 (m, 3H), 7.50-7.47 (m, 1H), 7.40-7.36 (m, 3H), 7.33-7.28 (m, 2H), 3.85 (t, *J* = 5.8 Hz, 2H), 2.79 (t, *J* = 6.1 Hz, 2H), 2.23 (s, 1H). $^{13}C\{1H\}$ NMR (75 MHz, $CDCl_3$): δ 131.9 (CH), 131.8 (CH), 131.7 (2 x CH), 128.6 (CH), 128.5 (2 x CH), 128.1 (CH), 127.8 (CH), 125.9 (C), 125.7 (C), 123.1 (C), 93.2 (C), 90.9 (C), 88.4 (C), 81.6 (C), 61.1 (CH_2), 24.2 (CH_2); HRMS (ESI) calcd for $C_{18}H_{14}NaO$ $[M + Na]^+$ 269.0942, found 269.0937.

4-(5-Methyl-2-(phenylethynyl)phenyl)but-3-yn-1-ol (S27)

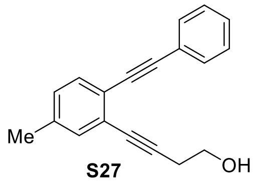

Following the general procedure II, compound **S27** was obtained from reaction of 2-bromo-4-methyl-1-(phenylethynyl)benzene (904 mg, 3.33 mmol) on reaction with but-3-yn-1-ol (0.30 mL, 4.00 mmol), $PdCl_2(PhCN)_2$ (76.6 mg, 0.20 mmol), CuI (38.1 mg, 0.20 mmol), P(*t*-Bu)$_3$ (80.8 mg, 0.40 mmol), *i*-Pr_2NH (2.33 mL, 16.65 mmol) and THF (7 mL) at rt for 22 h, followed by purification (15-40% EtOAc/hexanes). Yellow oil, 91% yield (788.7 mg). IR (ATR): ν 3382, 3059, 2921, 2232, 1596, 1497 cm^{-1}. ^1H NMR (300 MHz, $CDCl_3$): δ 7.49-7.41 (m, 2H), 7.32-7.14 (m, 5H), 6.96 (d, *J* = 7.8 Hz, 1H), 3.70 (t, *J* = 6.1 Hz, 2H), 2.64 (t, *J* = 6.2 Hz, 2H), 2.21 (s, 3H). $^{13}C\{1H\}$ NMR (75 MHz, $CDCl_3$): δ 138.3 (C), 132.4 (CH), 131.8 (CH), 131.7 (2 x CH), 128.8 (CH), 128.4 (2 x CH), 128.4 (CH), 125.6 (C), 123.3 (C), 122.9 (C), 92.4 (C), 90.4 (C), 88.6 (C), 81.7 (C), 61.1 (CH_2), 24.1 (CH_2), 21.3 (CH_3). HRMS (ESI) calcd for $C_{19}H_{16}NaO$ $[M + Na]^+$ 283.1099, found 283.1094.

4-(2-(Phenylethynyl)-5-(trifluoromethyl)phenyl)ut-3-yn-1-ol (S28)

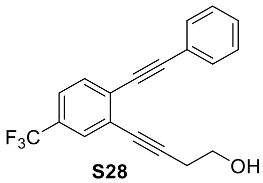

S28

Following the general procedure II, compound **S28** was obtained from 2-bromo-1-(phenylethynyl)-4-(trifluoromethyl)benzene (827 mg, 2.54 mmol) on reaction with but-3-yn-1-ol (0.23 mL, 3.05 mmol), $PdCl_2(PhCN)_2$ (59.1 mg, 0.15 mmol), CuI (29.5 mg, 0.15 mmol), P(*t*-Bu)$_3$ (61.7 mg, 0.31 mmol), *i*-Pr_2NH (1.8 mL, 12.7 mmol) and THF (5 mL) at rt for 22 h, followed by purification (20-40% EtOAc/hexanes). Yellow oil, 81% yield (650.6 mg). IR (ATR): ν 3368, 2925, 2218, 1332, 1124 cm^{-1}. ^{19}F NMR (282 MHz, $CDCl_3$): δ −63.00 (s, CF_3). ^1H NMR (300 MHz, $CDCl_3$): δ 7.71 (s, 1H), 7.62-7.56 (m, 3H), 7.49 (d, *J* = 7.9 Hz, 1H), 7.39-7.35 (m, 3H), 3.84 (t, *J* = 6.2 Hz, 2H), 2.77 (t, *J* = 6.2 Hz, 2H), 2.29 (s, 1H). $^{13}C\{1H\}$ NMR (75 MHz, $CDCl_3$): δ 132.2 (CH), 131.8 (2 x CH), 129.9 (q, $^2J_{CF}$ = 33.0 Hz, C), 129.3 (C), 129.1 (CH), 128.7 (q, $^3J_{CF}$ = 3.9 Hz, CH), 128.5 (2 x CH), 126.5 (C), 124.2 (q, $^3J_{CF}$ = 3.8 Hz, CH), 123.6 (q, $^1J_{CF}$ = 273.3 Hz, CF_3), 122.5 (C), 95.5 (C), 92.7 (C), 87.2 (C), 80.3 (C), 61.0 (CH_2), 24.0 (CH_2). HRMS (ESI) calcd for $C_{19}H_{13}F_3NaO$ $[M + Na]^+$ 337.0816, found 337.0812.

4-(2-((4-Methoxyphenyl)ethynyl)phenyl)but-3-yn-1-ol (S29)

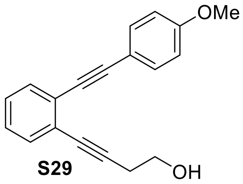

S29

Following the general procedure II, compound **S29** was obtained from 1-bromo-2-((4-methoxyphenyl)ethynyl)benzene (660 mg, 2.30 mmol) on reaction with but-3-yn-1-ol (0.21 mL, 2.76 mmol), $PdCl_2(PhCN)_2$ (52.9 mg, 0.14 mmol), CuI (26.3 mg, 0.14 mmol), P(*t*-Bu)$_3$ (55.8 mg, 0.28 mmol), *i*-Pr_2NH (1.6 mL, 11.5 mmol) and THF (5 mL) at rt for 17 h, followed by purification (25-40% EtOAc/hexanes). Yellow oil, 90% yield (571.4 mg). IR (ATR): ν 3386, 2957, 2872, 1513, 1251 cm^{-1}. ^1H NMR (300 MHz, $CDCl_3$): δ 7.53 (d, *J* = 8.3 Hz, 3H), 7.48-7.45 (m, 1H), 7.32-7.23 (m, 2H), 6.91 (d, *J* = 8.3 Hz, 2H), 3.85 (s, 3H), 3.85 (t, *J* = 7.8 Hz, 2H), 2.79 (t, *J* = 6.4 Hz, 2H), 2.21 (s, 1H). $^{13}C\{1H\}$ NMR (75 MHz, $CDCl_3$): δ 159.9 (C), 133.2 (2 x CH), 131.8 (CH), 131.7 (CH), 127.7 (CH), 127.7 (CH), 126.2 (C), 125.5 (C), 115.2 (C), 114.1 (2 x CH), 93.3 (C),

90.6 (C), 87.1 (C), 81.7 (C), 61.1 (CH_2), 55.3 (CH_2), 24.2 (CH_3). HRMS (ESI) calcd for $C_{19}H_{16}NaO_2$ [M + Na]$^+$ 299.1048, found 299.1054.

4-(2-(Thiophen-3-ylethynyl)phenyl)but-3-yn-1-ol (S30)

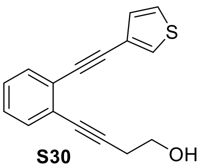

S30

Following the general procedure II, compound **S30** was obtained from 3-((2-bromophenyl)ethynyl)thiophene (705 mg, 2.68 mmol) on reaction with but-3-yn-1-ol (0.24 mL, 3.22 mmol), $PdCl_2(PhCN)_2$ (61.9 mg, 0.16 mmol), CuI (30.5 mg, 0.16 mmol), P(*t*-Bu)$_3$ (65.5 mg, 0.32 mmol), *i*-Pr_2NH (1.9 mL, 13.4 mmol) and THF (5 mL) at rt for 16 h, followed by purification (20-30% EtOAc/hexanes). Yellow oil, 83% yield (561.1 mg). IR (ATR): ν 3381, 3107, 2918, 2211, 1477 cm^{-1}. ^{1}H NMR (300 MHz, $CDCl_3$): δ 7.62 (d, *J* = 2.9 Hz, 1H), 7.55-7.52 (m, 1H), 7.48-7.45 (m, 1H), 7.34-7.31 (m, 1H), 7.29-7.24 (m, 3H), 3.83 (t, *J* = 6.1 Hz, 2H), 2.77 (t, *J* = 6.1 Hz, 2H), 2.44 (s, 1H). ^{13}C{1H} NMR (75 MHz, $CDCl_3$): δ 131.8 (CH), 131.8 (CH), 129.9 (CH), 129.2 (CH), 128.0 (CH), 127.8 (CH), 125.9 (C), 125.6 (C), 125.6 (CH), 122.1 (C), 91.0 (C), 88.3 (C), 88.0 (C), 81.6 (C), 61.0 (CH_2), 24.1 (CH_2). HRMS (ESI) calcd for $C_{16}H_{12}NaOS$ [M + Na]$^+$ 275.0507, found 275.0502.

4-(2-(Cyclohex-1-en-1-ylethynyl)phenyl)but-3-yn-1-ol (S31)

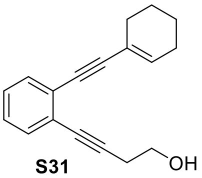

S31

Following the general procedure II, compound **S31** was obtained from reaction of 1-bromo-2-(cyclohex-1-en-1-ylethynyl)benzene (700 mg, 2.68 mmol) on reaction with but-3-yn-1-ol (0.24 mL, 3.22 mmol), $PdCl_2(PhCN)_2$ (61.8 mg, 0.16 mmol), CuI (30.4 mg, 0.16 mmol), P(*t*-Bu)$_3$ (65.1 mg, 0.32 mmol), *i*-Pr_2NH (1.9 mL, 13.4 mmol) and THF (5 mL) at rt for 22 h, followed by purification (20-30% EtOAc/hexanes). Yellow oil, 90% yield (603.6 mg). IR (ATR): ν 3360, 2927, 2858, 1478, 1443 cm^{-1}. ^{1}H NMR (300 MHz, $CDCl_3$): δ 7.41-7.36 (m, 2H), 7.21-7.17 (m, 2H), 6.26-6.22 (m, 1H), 3.80 (t, *J* = 6.2 Hz, 2H), 2.72 (t, *J* = 6.3 Hz, 2H), 2.35 (s, 1H), 2.25-2.21 (m, 2H), 2.18-2.13 (m, 2H), 1.68-1.62 (m, 4H). ^{13}C{1H} NMR (75 MHz, $CDCl_3$): δ ^{13}C NMR (75 MHz, $CDCl_3$): δ 135.8 (CH), 131.7 (CH), 131.7 (CH), 127.6 (CH), 127.5 (CH), 126.3 (C), 125.4 (C), 120.7 (C), 95.2 (C), 90.5 (C), 85.9 (C), 81.6 (C), 61.1 (CH_2), 29.2 (CH_2), 25.9 (CH_2), 24.1 (CH_2), 22.4 (CH_2), 21.5 (CH_2). HRMS (ESI) calcd for $C_{18}H_{18}NaO$ [M + Na]$^+$ 273.1255, found 273.1250.

4-(2-(Hept-1-yn-1-yl)phenyl)but-3-yn-1-ol (S32)

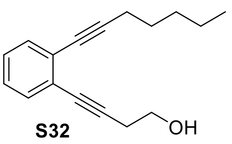

S32

Following the general procedure II, compound **S32** was obtained from reaction of 4-(2-bromophenyl)but-3-yn-1-ol (846 mg, 3.37 mmol) on reaction with but-3-yn-1-ol (0.31 mL, 4.04 mmol), $PdCl_2(PhCN)_2$ (77.5 mg, 0.20 mmol), CuI (38.5 mg, 0.20 mmol), P(*t*-Bu)$_3$ (81.8 mg, 0.40 mmol), *i*-Pr_2NH (2.4 mL, 16.8 mmol) and THF (6 mL) at rt for 22 h, followed by purification (15-25% EtOAc/hexanes). Yellow oil, 90% yield (615.3 mg). ^{1}H NMR (300 MHz, $CDCl_3$): δ 7.41-7.35 (m, 2H), 7.23-7.16 (m, 2H), 3.84-3.78 (m, 2H), 2.73 (t, J = 7.2 Hz, 2H), 2.46 (t, J = 7.3 Hz, 2H), 2.26-2.22 (m, 1H), 1.66-1.61 (m, 2H), 1.49-1.32 (m, 4H), 0.92 (t, J = 7.3 Hz, 3H). ^{13}C{1H} NMR (75 MHz, $CDCl_3$): δ 132.0 (CH), 131.5 (CH), 127.6 (CH), 127.3 (CH), 126.7 (C), 125.4 (C), 94.6 (C), 90.0 (C), 82.1 (C), 79.7 (C), 61.0 (CH_2), 31.1 (CH_2), 28.5 (CH_2), 24.2 (CH_2), 22.3 (CH_2), 19.6 (CH_2), 14.0 (CH_3). HRMS (ESI) calcd for $C_{17}H_{20}ONa$ [M + Na]$^+$ 263.1412, found 264.1407.

4-(2-(Phenylethynyl)-4-(trifluoromethyl)phenyl)ut-3-yn-1-ol (S33)

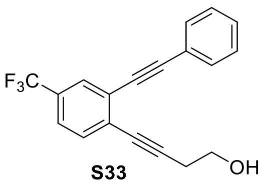

S33

Following the general procedure II, compound **S33** was obtained from 1-bromo-2-(phenylethynyl)-4-(trifluoromethyl)benzene (994 mg, 3.06 mmol) on reaction with but-3-yn-1-ol (0.28 mL, 3.67 mmol), $PdCl_2(PhCN)_2$ (82.9 mg, 0.22 mmol), CuI (41.9 mg, 0.22mmol), P(*t*-Bu)$_3$ (74.2 mg, 0.37 mmol), *i*-Pr_2NH (2.14 mL, 15.3 mmol) and THF (5 mL) at rt for 22 h, followed by purification (25-50% EtOAc/hexanes). Yellow oil, 93% yield (891.7 mg). ^{1}H NMR (300 MHz, $CDCl_3$): δ 7.78 (s, 1H), 7.61-7.46 (m, 4H), 7.39-7.37 (m, 3H), 3.84 (t, J = 6.1 Hz, 2H), 2.78 (t, J = 6.1 Hz, 2H), 2.24 (s, 1H). ^{13}C{1H} NMR (75 MHz, $CDCl_3$): δ 132.2 (CH), 131.8 (2 x CH), 129.8 (q, $^2J_{CF}$ = 33.3 Hz, C), 129.2 (C), 129.0 (CH), 128.7 (q, $^3J_{CF}$ = 4.3 Hz, CH), 128.5 (2 x CH), 126.6 (C), 124.4 (q, $^3J_{CF}$ = 4.3 Hz, CH), 123.6 (q, $^1J_{CF}$ = 276.4 Hz, CF_3), 122.5 (C), 94.6 (C), 93.8 (C), 87.1 (C), 80.5 (C), 60.9 (CH_2), 24.1 (CH_2). ^{19}F NMR (282 MHz, $CDCl_3$): δ –62.91 (s, CF_3). HRMS (ESI) calcd for $C_{19}H_{13}F_3ONa$ [M + Na]$^+$ 337.0816, found 337.0811.

General procedure for the synthesis of mesylates (S34-S41)

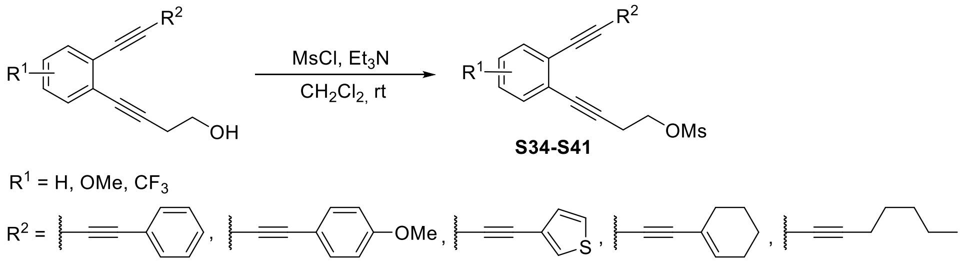

The alcohol was dissolved in CH_2Cl_2 (6-15 mL) and then Et_3N (1.2 equiv.) and MsCl in portions (1.2 equiv.) were added at 0°C. The reaction was stirred at room temperature until TLC showed that the reaction was completed. The reaction was quenched with saturated aqueous NH_4Cl solution (15 mL) and extracted with EtOAc (2 x 15 mL). The combined organic phase was washed with brine (15 mL), dried ($MgSO_4$ anhyd.), filtered and concentrated. The resulting crude was purified by flash column chromatography (EtOAc/hexanes) to afford, after concentration and high vacuum drying, the corresponding mesylate.

4-(2-(Phenylethynyl)phenyl)but-3-yn-1-yl methanesulfonate (S34)

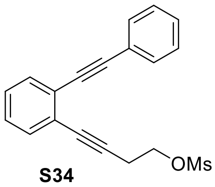

S34

Following the general procedure, compound **S34** was obtained from alcohol **S26** (660 mg, 2.68 mmol) on reaction with MsCl (0.25 mL, 3.22 mmol) and Et_3N (0.45 mL, 3.22 mmol) in CH_2Cl_2 (15 mL) at rt for 7 h, followed by purification (20-30% EtOAc/hexanes). Yellow oil, 95% yield (825.7 mg). IR (ATR): ν 3029, 2937, 1475, 1357, 1174 cm^{-1}. ^{1}H NMR (300 MHz, $CDCl_3$): δ 7.60-7.54 (m, 3H), 7.49-7.37 (m, 4H), 7.33-7.28 (m, 2H), 4.44 (t, J = 6.7 Hz, 2H), 3.01 (s, 3H), 3.00 (t, J = 6.2 Hz, 2H). ^{13}C{1H} NMR (75 MHz, $CDCl_3$): δ 132.0 (CH), 131.9 (CH), 131.7 (2 x CH), 128.6 (CH), 128.5 (2 x CH), 128.1 (2 x CH), 125.8 (C), 125.3 (C), 123.1 (C), 93.2 (C), 88.1 (C), 81.7 (C), 67.4 (CH_2), 37.7 (CH_3), 21.0 (CH_2). HRMS (ESI) calcd for $C_{19}H_{16}NaO_3S$ $[M + Na]^+$ 347.0718, found 347.0714.

4-(5-Methyl-2-(phenylethynyl)phenyl)but-3-yn-1-yl methanesulfonate (S35)

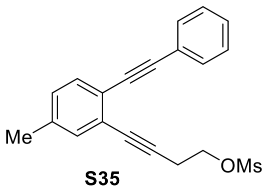

S35

Following the general procedure, compound **S35** was obtained from alcohol **S27** (785 mg, 3.02 mmol) on reaction with MsCl (0.28 mL, 3.62 mmol) and Et_3N (0.50 mL, 3.62 mmol) in CH_2Cl_2 (15 mL) at rt for 17 h, followed by purification (20-40% EtOAc/hexanes). Yellow oil, 90% yield (918.1 mg). IR (ATR): ν 3029, 2920, 1498, 1355, 1172 cm^{-1}. ^{1}H NMR (300 MHz, $CDCl_3$): δ ^{1}H NMR (300 MHz, $CDCl_3$): δ 7.46-7.41 (m, 2H), 7.32-7.16 (m, 5H), 6.99 (d, J = 7.9 Hz, 1H), 4.28 (t, J = 6.9 Hz, 2H), 2.86 (s, 3H), 2.86 (t, J = 7.1 Hz, 2H), 2.22 (s, 3H). ^{13}C{1H} NMR (75 MHz, $CDCl_3$): δ 138.3 (C), 132.6 (CH), 131.8 (CH), 131.6 (2 x CH), 129.1 (CH), 128.5 (2 x CH), 128.5 (CH), 125.1 (C), 123.3 (C), 122.9 (C), 92.5 (C), 88.3 (C), 87.8 (C), 81.8 (C), 67.6 (CH_2), 37.6 (CH_3), 21.3 (CH_3), 21.0 (CH_2). HRMS (ESI) calcd for $C_{20}H_{18}NaO_3S$ $[M + Na]^+$ 361.0874, found 361.0871.

4-(2-(Phenylethynyl)-5-(trifluoromethyl)phenyl)but-3-yn-1-yl methanesulfonate (S36)

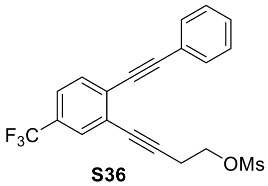

S36

Following the general procedure, compound **S36** was obtained from alcohol **S28** (600 mg, 1.91 mmol) on reaction with MsCl (0.18 mL, 2.29 mmol) and Et_3N (0.32 mL, 2.29 mmol) in CH_2Cl_2 (15 mL) at rt for 17 h, followed by purification (25-40% EtOAc/hexanes). Yellow oil, 89% yield (664.2 mg). IR (ATR): ν 2938, 2364, 2330, 1572, 1171 cm^{-1}. ^{1}H NMR (300 MHz, $CDCl_3$): δ 7.70 (s, 1H), 7.63-7.50 (m, 4H), 7.41-7.37 (m, 3H), 4.41 (t, J = 6.8 Hz, 2H), 2.99 (t, J = 6.4 Hz, 2H), 2.98 (s, 3H). ^{13}C{1H} NMR (75 MHz, $CDCl_3$): δ 132.2 (CH), 131.8 (2 x CH), 129.9 (q, $^2J_{CF}$ =

33.0 Hz, C), 129.4 (C), 129.2 (CH), 128.9 (q, $^3J_{CF}$ = 3.9 Hz, CH), 128.6 (2 x CH), 126.0 (C), 124.6 (q, $^3J_{CF}$ = 3.7 Hz, CH), 123.5 (q, $^1J_{CF}$ = 272.6 Hz, CF_3), 122.5 (C), 95.7 (C), 89.9 (C), 87.0 (C), 80.6 (C), 67.0 (CH_2), 37.6 (CH_3), 21.0 (CH_2). ^{19}F NMR (282 MHz, $CDCl_3$): δ −63.01 (s, CF_3). HRMS (ESI) calcd for $C_{20}H_{15}F_3NaO_3S$ [M + Na]$^+$ 415.0592, found 415.0589.

4-(2-((4-Methoxyphenyl)ethynyl)phenyl)but-3-yn-1-yl methanesulfonate (S37)

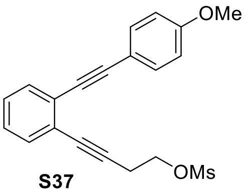

S37

Following the general procedure, compound **S37** was obtained from alcohol **S29** (420 mg, 1.52 mmol) on reaction with MsCl (0.14 mL, 1.82 mmol) and Et_3N (0.25 mL, 1.82 mmol) in CH_2Cl_2 (6 mL) at rt for 48 h, followed by purification (20-40% EtOAc/hexanes). Yellow oil, 93% yield (500.9 mg). IR (ATR): ν 2958, 2922, 2216, 1512, 1250, 1176 cm^{-1}. ^{1}H NMR (300 MHz, $CDCl_3$): δ 7.73-7.50 (m, 3H), 7.46-7.44 (m, 1H), 7.32-7.25 (m, 2H), 6.93 (d, *J* = 8.9 Hz, 2H), 4.43 (t, *J* = 6.6 Hz, 2H), 3.85 (s, 3H), 3.02 (s, 3H), 2.99 (t, *J* = 7.1 Hz, 2H). ^{13}C{1H} NMR (75 MHz, $CDCl_3$): δ 159.9 (C), 133.2 (2 x CH), 132.0 (CH), 131.7 (CH), 128.0 (CH), 127.7 (CH), 126.2 (C), 125.0 (C), 115.2 (C), 114.2 (2 x CH), 93.4 (C), 88.0 (C), 86.9 (C), 81.8 (C), 67.5 (CH_2), 55.4 (CH_3), 37.7 (CH_3), 21.0 (CH_2). HRMS (ESI) calcd for $C_{20}H_{18}NaO_4S$ [M + Na]$^+$ 377.0823, found 377.0833.

4-(2-(Thiophen-3-ylethynyl)phenyl)but-3-yn-1-yl methanesulfonate (S38)

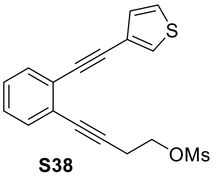

S38

Following the general procedure, compound **S38** was obtained from alcohol **S30** (560 mg, 2.22 mmol) on reaction with MsCl (0.21 mL, 2.66 mmol) and Et_3N (0.37 mL, 2.66 mmol) in CH_2Cl_2 (15 mL) at rt for 48 h, followed by purification (25% EtOAc/hexanes). Yellow oil, 95% yield (696.7 mg). IR (ATR): ν 2959, 2920, 1358, 1176 cm^{-1}. ^{1}H NMR (300 MHz, $CDCl_3$): δ 7.60 (d, *J* = 2.9 Hz, 2H), 7.54-7.50 (m, 1H), 7.49-7.44 (m, 1H), 7.36-7.33 (m, 1H), 7.31-7.23 (m, 3H), 4.42 (t, *J* = 6.8 Hz, 2H), 3.01 (s, 2H), 2.98 (t, *J* = 6.8 Hz, 2H). ^{13}C{1H} NMR (75 MHz, $CDCl_3$): δ 132.0 (CH), 131.9 (CH), 129.9 (CH), 129.1 (CH), 128.1 (CH), 128.0 (CH), 125.8 (C), 125.7 (CH), 125.2 (C), 122.1 (C), 88.4 (C), 88.3 (C), 87.7 (C), 81.7 (C), 67.5 (CH_2), 37.7 (CH_3), 21.0 (CH_2). HRMS (ESI) calcd for $C_{17}H_{14}NaO_3S_2$ [M + Na]$^+$ 353.0282, found 353.0277.

4-(2-(Cyclohex-1-en-1-ylethynyl)phenyl)but-3-yn-1-yl methanesulfonate (S39)

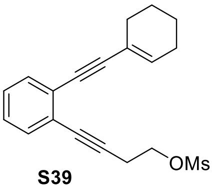

S39

Following the general procedure, compound **S39** was obtained from alcohol **S31** (600 mg, 2.40 mmol) on reaction with MsCl (0.22 mL, 2.88 mmol) and Et_3N (0.40 mL, 2.88 mmol) in CH_2Cl_2 (10 mL) at rt for 7 h, followed by purification (25% EtOAc/hexanes). Yellow oil, 90% (708.4 mg). IR (ATR): ν 2928, 2857, 2200, 1355, 1172 cm^{-1}. ^1H NMR (300 MHz, $CDCl_3$): δ 7.43-7.39 (m, 2H), 7.28-7.22 (m, 2H), 6.29-6.25 (m, 1H), 4.41 (t, J = 7.2 Hz, 2H), 3.07 (s, 3H), 2.95 (t, J = 7.0 Hz, 2H), 2.29-2.25 (m, 2H), 2.22-2.15 (m, 2H), 1.75-1.60 (m, 4H). $^{13}C\{1H\}$ NMR (75 MHz, $CDCl_3$): δ 136.0 (CH), 131.9 (CH), 131.7 (CH), 128.0 (CH), 127.5 (CH), 126.4 (C), 125.0 (C), 120.7 (C), 95.4 (C), 87.8 (C), 85.6 (C), 81.8 (C), 67.6 (CH_2), 37.7 (CH_3), 29.3 (CH_2), 25.8 (CH_2), 22.3 (CH_2), 21.5 (CH_2), 20.9 (CH_2). HRMS (ESI) calcd for $C_{19}H_{20}NaO_3S$ $[M + Na]^+$ 351.1031, found 351.1027.

4-(2-(Hept-1-yn-1-yl)phenyl)but-3-yn-1-yl methanesulfonate (S40)

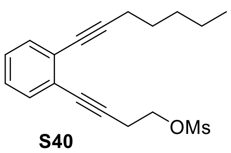

S40

Following the general procedure, compound **S40** was obtained from alcohol **S32** (373.2 mg, 1.55 mmol) on reaction with MsCl (0.14 mL, 1.86 mmol) and Et_3N (0.26 mL, 1.86 mmol) in CH_2Cl_2 (7 mL) at rt for 4 h, followed by purification (15% EtOAc/hexanes). Yellow oil, 89% (442.2 mg). ^1H NMR (300 MHz, $CDCl_3$): δ 7.42-7.38 (m, 2H), 7.29-7.18 (m, 2H), 4.43 (t, J = 7.1 Hz, 2H), 3.09 (s, 3H), 2.95 (t, J = 7.1 Hz, 2H), 2.49 (t, J = 7.1 Hz, 2H), 1.66-1.61 (m, 2H), 1.52-1.35 (m, 4H), 0.95 (t, J = 7.1 Hz, 3H). $^{13}C\{1H\}$ NMR (75 MHz, $CDCl_3$): δ 132.0 (CH), 131.9 (CH), 127.9 (CH), 127.3 (CH), 126.6 (C), 125.0 (C), 94.8 (C), 87.4 (C), 82.0 (C), 79.3 (C), 67.6 (CH_2), 37.8 (CH_3), 31.1 (CH_2), 28.5 (CH_2), 22.2 (CH_2), 20.9 (CH_2), 19.6 (CH_2), 14.0 (CH_3). HRMS (ESI) calcd for $C_{18}H_{22}O_3NaS$ $[M + Na]^+$ 341.1187, found 341.1181.

4-(2-(Phenylethynyl)-4-(trifluoromethyl)phenyl)but-3-yn-1-yl methanesulfonate (S41)

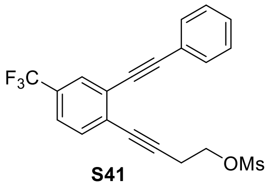

S41

Following the general procedure, compound **S41** was obtained from alcohol **S33** (891.7 mg, 2.84 mmol) on reaction with MsCl (0.26 mL, 3.40 mmol) and Et_3N (0.47 mL, 3.40 mmol) in CH_2Cl_2 (10 mL) at rt for 22 h, followed by purification (20-40% EtOAc/hexanes). Yellow oil, 92% yield (1.02 g). ^1H NMR (300 MHz, $CDCl_3$): δ 7.77 (s, 1H), 7.60-7.47 (m, 4H), 7.41-7.38 (m, 3H), 4.40 (t, J = 6.8 Hz, 2H), 2.99 (t, J = 6.9 Hz, 2H), 2.97 (s, 3H). $^{13}C\{1H\}$ NMR (75 MHz, $CDCl_3$): δ 132.5 (CH), 131.8 (2 x CH), 130.0 (q, $^2J_{CF}$ = 33.3 Hz, C), 129.1 (CH), 128.8 (C), 128.7 (q, $^3J_{CF}$ = 4.3 Hz, CH), 128.6 (2 x CH), 126.6 (C), 124.5 (q, $^3J_{CF}$ = 4.3 Hz, CH), 123.5 6 (q, $^1J_{CF}$ = 276.4 Hz, CF_3), 122.5 (C), 94.7 (C), 91.0 (C), 86.9 (C), 80.7 (C), 67.1 (CH_2), 37.6 (CH_3), 21.0 (CH_2). ^{19}F NMR (282 MHz, $CDCl_3$): δ –62.95 (s, CF_3). HRMS (ESI) calcd for $C_{20}H_{15}F_3O_3NaS$ $[M + Na]^+$ 415.0592, found 415.0587.

General procedure for the synthesis of azido-diynes from mesylates (3a-3h)

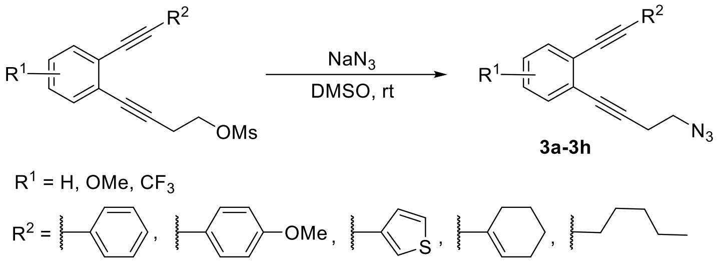

Sodium azide (1.5 equiv.) was added to a solution of mesylate compound (1 equiv.) in DMSO (6-10 mL) and the mixture was stirred at room temperature. The reaction was quenched with saturated aqueous $NaHCO_3$ solution (15 mL) and extracted with EtOAc (2 x 15 mL). The combined organic phase was washed with brine (15 mL), dried ($MgSO_4$ anhyd.), filtered and concentrated. The resulting crude was purified by flash column chromatography (EtOAc/hexanes) to afford, after concentration and high vacuum drying, the corresponding azide.

1-(4-Azidobut-1-yn-1-yl)-2-(phenylethynyl)benzene (3a)[16]

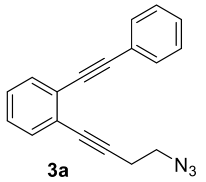

Following the general procedure, compound **3a** was obtained from mesylate **S34** (820 mg, 2.53 mmol) on reaction with NaN_3 (246.7 mg, 3.80 mmol) in DMSO (7 mL) at rt for 21 h, followed by purification (3% EtOAc/hexanes). Yellow oil, 75% yield (514.3 mg). IR (ATR): ν 3348, 2948, 2836, 1651, 1016 cm^{-1}. ^1H NMR (300 MHz, $CDCl_3$): δ 7.63-7.55 (m, 3H), 7.53-7.47 (m, 1H), 7.45-7.38 (m, 3H), 7.37-7.26 (m, 2H), 3.54 (t, J = 6.9 Hz, 2H), 2.83 (t, J = 6.9 Hz, 2H). $^{13}C\{1H\}$ NMR (75 MHz, $CDCl_3$): δ 132.0 (CH), 131.8 (CH), 131.7 (2 x CH), 128.5 (CH), 128.5 (2 x CH), 128.0 (CH), 127.9 (CH), 125.8 (C), 125.7 (C), 123.3 (C), 93.2 (C), 90.1 (C), 88.3 (C), 81.3 (C), 50.1 (CH_2), 20.8 (CH_2). HRMS (ESI) calcd for $C_{18}H_{13}N_3Na$ $[M + Na]^+$ 294.1007, found 294.1002.

2-(4-Azidobut-1-yn-1-yl)-4-methyl-1-(phenylethynyl)benzene (3b)

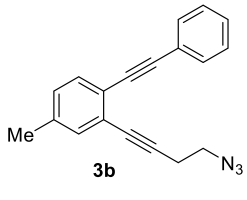

Following the general procedure, compound **3b**was obtained from mesylate **S35** (821 mg, 2.43 mmol) on reaction with NaN_3 (236.9 mg, 3.64 mmol) in DMSO (7 mL) at rt for 48 h, followed by purification (2% EtOAc/hexanes). Colorless oil, 75% yield (518.0 mg). IR (ATR): ν 2953, 2923, 2362, 2097, 1460 cm^{-1}. ^1H NMR (300 MHz, $CDCl_3$): δ ^1H NMR (300 MHz, $CDCl_3$) δ 7.58-7.54 (m, 2H), 7.43-7.33 (m, 4H), 7.29 (s, 1H), 7.09 (d, J = 8.0 Hz, 1H), 3.51 (t, J = 7.1 Hz, 2H), 2.80 (t, J = 7.1 Hz, 2H), 2.34 (s, 3H). $^{13}C\{1H\}$ NMR (75 MHz, $CDCl_3$): δ 138.2 (C), 132.5 (CH), 131.7 (CH), 131.6 (2 x CH), 128.9 (CH), 128.4 (2 x CH), 128.3 (CH), 125.5 (C), 123.5 (C), 122.9 (C), 92.4 (C), 89.6 (C), 88.4 (C), 81.4 (C), 50.1 (CH_2), 21.3 (CH_3), 20.7 (CH_2). HRMS (ESI) calcd for $C_{19}H_{15}N_3Na$ $[M + Na]^+$ 308.1164, found 308.1159.

2-(4-Azidobut-1-yn-1-yl)-1-(phenylethynyl)-4-(trifluoromethyl)benzene (3c)

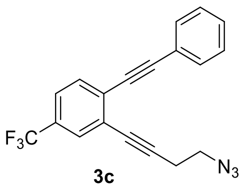

3c

Following the general procedure, compound **3c** was obtained from mesylate **S36** (577 mg, 1.47 mmol) on reaction with NaN_3 (143.3 mg, 2.21 mmol) in DMSO (6 mL) at rt for 48 h, followed by purification (2% EtOAc/hexanes). Colorless oil, 76% yield (379.8 mg). IR (ATR): ν 2925, 2218, 2102, 1331, 1126 cm^{-1}. ^{1}H NMR (300 MHz, $CDCl_3$): δ 7.72 (s, 1H), 7.63-7.56 (m, 3H), 7.51 (d, J = 8.5 Hz, 1H), 7.41-7.39 (m, 3H), 3.53 (t, J = 6.9 Hz, 2H), 2.81 (t, J = 6.9 Hz, 2H). $^{13}C\{1H\}$ NMR (75 MHz, $CDCl_3$): δ 132.1 (CH), 131.8 (2 x CH), 129.9 (q, $^{2}J_{CF}$ = 32.8 Hz, C), 129.4 (C), 129.0 (CH), 128.8 (q, $^{3}J_{CF}$ = 3.9 Hz, CH), 128.5 (2 x CH), 126.3 (C), 124.3 (q, $^{1}J_{CF}$ = 273.2 Hz, CF_3), 123.6 (q, $^{3}J_{CF}$ = 3.7 Hz, CH), 122.7 (C), 95.5 (C), 91.8 (C), 87.1 (C), 80.2 (C), 49.9 (CH_2), 20.7 (CH_2). ^{19}F NMR (282 MHz, $CDCl_3$): δ –63.00 (s, CF_3). HRMS (ESI) calcd for $C_{19}H_{12}F_3N_3Na$ $[M + Na]^+$ 362.0881, found 362.0881.

1-(4-Azidobut-1-yn-1-yl)-2-((4-methoxyphenyl)ethynyl)benzene (3d)

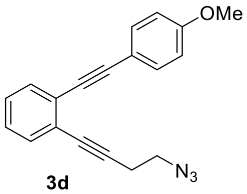

3d

Following the general procedure, compound **3d** was obtained from mesylate **S37** (444 mg, 1.25 mmol) on reaction with NaN_3 (163.0 mg, 2.51 mmol) in DMSO (10 mL) at rt for 20 h, followed by purification (15% EtOAc/hexanes). Yellow oil, 79% yield (299.8 mg). IR (ATR): ν 2955, 2923, 2051, 1511, 1463 cm^{-1}. ^{1}H NMR (300 MHz, $CDCl_3$): δ 7.41 (d, J = 8.9 Hz, 3H), 7.37-7.34 (m, 1H), 7.21-7.12 (m, 2H), 6.81 (d, J = 8.7 Hz, 2H), 3.75 (s, 3H), 3.42 (t, J = 6.9 Hz, 2H), 2.71 (t, J = 6.9 Hz, 2H). $^{13}C\{1H\}$ NMR (75 MHz, $CDCl_3$): δ 159.8 (C), 133.1 (2 x CH), 131.9 (CH), 131.6 (CH), 127.8 (CH), 127.6 (CH), 126.2 (C), 125.4 (C), 115.4 (C), 114.1 (2 x CH), 93.3 (C), 89.8 (C), 87.0 (C), 81.4 (C), 55.3 (CH_3), 50.1 (CH_2), 20.7 (CH_2). HRMS (ESI) calcd for $C_{19}H_{15}N_3NaO$ $[M + Na]^+$ 324.1113, found 324.1108.

3-((2-(4-Azidobut-1-yn-1-yl)phenyl)ethynyl)thiophene (3e)

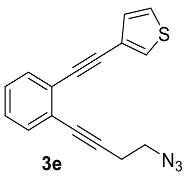

3e

Following the general procedure, compound **3e** was obtained from mesylate **S38** (695 mg, 2.10 mmol) on reaction with NaN_3 (204.8 mg, 3.15 mmol) in DMSO (10 mL) at rt for 21 h, followed by purification (3% EtOAc/hexanes). Yellow oil, 89% yield (518.9 mg). IR (ATR): ν 2919, 2869, 2104, 1735, 1459 cm^{-1}. ^{1}H NMR (300 MHz, $CDCl_3$): δ 7.59 (d, J = 3.1 Hz, 1H), 7.56-7.47 (m, 2H), 7.36-7.25 (m, 4H), 3.52 (t, J = 6.8 Hz, 2H), 2.81 (t, J = 6.8 Hz, 2H). $^{13}C\{1H\}$ NMR (75 MHz, $CDCl_3$): δ 132.0 (CH), 131.8 (CH), 129.9 (CH), 128.9 (CH), 128.0 (CH), 127.9 (CH), 125.8

(C), 125.6 (C), 125.5 (CH), 122.4 (C), 90.1 (C), 88.3 (C), 87.8 (C), 81.3 (C), 50.0 (CH_2), 20.8 (CH_2). HRMS (ESI) calcd for $C_{16}H_{11}N_3NaS$ [M + Na]$^+$ 300.0567, found 300.0567.

1-(4-Azidobut-1-yn-1-yl)-2-(cyclohex-1-en-1-ylethynyl)benzene (3f)

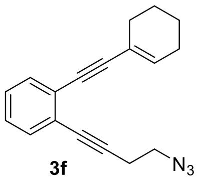

3f

Following the general procedure, compound **3f** was obtained from mesylate **S39** (653 mg, 1.99 mmol) on reaction with NaN_3 (258.5 mg, 3.98 mmol) in DMSO (10 mL) at rt for 20 h, followed by purification (3% EtOAc/hexanes). Colorless oil, 84% yield (462.5 mg). IR (ATR): ν 2926, 2200, 2086, 1478, 1263 cm^{-1}. ^{1}H NMR (300 MHz, $CDCl_3$): δ 7.44-7.41 (m, 2H), 7.27-7.21 (m, 2H), 6.30-6.26 (m, 1H), 3.53 (t, *J* = 7.1 Hz, 2H), 2.79 (t, *J* = 7.1 Hz, 2H), 2.31-2.27 (m, 2H), 2.23-2.16 (m, 2H), 1.77-1.66 (m, 4H). ^{13}C{1H} NMR (75 MHz, $CDCl_3$): δ 135.6 (CH), 131.8 (CH), 131.6 (CH), 127.7 (CH), 127.4 (CH), 126.4 (C), 125.3 (C), 120.9 (C), 95.2 (C), 89.6 (C), 85.7 (C), 81.4 (C), 50.1 (CH_2), 29.3 (CH_2), 25.9 (CH_2), 22.4 (CH_2), 21.5 (CH_2), 20.7 (CH_2). HRMS (ESI) calcd for $C_{18}H_{17}N_3Na$ [M +Na]$^+$ 298.1320, found 298.1315.

1-(4-Azidobut-1-yn-1-yl)-2-(hept-1-yn-1-yl)benzene (3g)

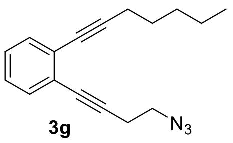

3g

Following the general procedure, compound **3g** was obtained from mesylate **S40** (442 mg, 1.39 mmol) on reaction with NaN_3 (135.4 mg, 2.08 mmol) in DMSO (5 mL) at rt for 23 h, followed by purification (10% EtOAc/hexanes). Colorless oil, 77% yield (283.6 mg). ^{1}H NMR (300 MHz, $CDCl_3$): δ 7.41-7.36 (m, 2H), 7.23-7.15 (m, 2H), 3.50 (t, *J* = 7.1 Hz, 2H), 2.76 (t, *J* = 7.1 Hz, 2H), 2.47 (t, *J* = 7.1 Hz, 2H), 1.69-1.60 (m, 2H), 1.53-1.32 (m, 4H), 0.94 (t, *J* = 7.0 Hz, 3H). ^{13}C{1H} NMR (75 MHz, $CDCl_3$): δ 131.9 (2 x CH), 127.7 (CH), 127.2 (CH), 126.6 (C), 125.4 (C), 94.5 (C), 89.2 (C), 81.5 (C), 79.4 (C), 50.0 (CH_2), 31.1 (CH_2), 28.5 (CH_2), 22.3 (CH_2), 20.7 (CH_2), 19.6 (CH_2), 14.0 (CH_3). HRMS (ESI) calcd for $C_{17}H_{19}N_3Na$ [M + Na]$^+$ 288.1477, found 288.1471.

1-(4-Azidobut-1-yn-1-yl)-2-(phenylethynyl)-4-(trifluoromethyl)benzene (3h)

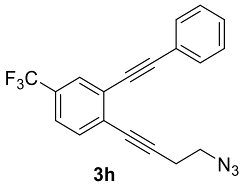

3h

Following the general procedure, compound **3h** was obtained from mesylate **S41** (940 mg, 2.39 mmol) on reaction with NaN_3 (233.6 mg, 3.59 mmol) in DMSO (7mL) at rt for 22 h, followed by purification (10% EtOAc/hexanes). Colorless oil, 74% yield (601.3 mg). ^{1}H NMR (300 MHz, $CDCl_3$): δ 7.79 (s, 1H), 7.60-7.48 (m, 4H), 7.41-7.37 (m, 3H), 3.52 (t, *J* = 6.8 Hz, 2H), 2.82 (t, *J* = 6.9 Hz, 2H). ^{13}C{1H} NMR (75 MHz, $CDCl_3$): δ 132.4 (CH), 131.8 (2 x CH), 129.9 (q, $^2J_{CF}$ = 33.3 Hz, C), 129.1 (C), 128.9 (CH), 128.6 (q, $^3J_{CF}$ = 4.3 Hz, CH), 128.5 (2 x CH), 126.6 (C), 124.4

(q, $^3J_{CF}$ = 4.3 Hz, CH), 123.6 (q, $^1J_{CF}$ = 276.4 Hz, CF_3), 122.7 (C), 94.6 (C), 92.8 (C), 86.9 (C), 80.4 (C), 49.8 (CH_2), 20.8 (CH_2). ^{19}F NMR (282 MHz, $CDCl_3$): δ –62.95 (s, CF_3). HRMS (ESI) calcd for $C_{19}H_{13}F_3N_3$ $[M + H]^+$ 340.0983, found 340.0979.

2. Detection of intermediate 5b by ^{1}H NMR.

The reaction of **3b** and $InCl_3$ (5 mol%) in DCE at 110 °C was monitored by ^{1}H NMR. During the reaction, the formation of pyrrole intermediate **5b** by *5-endo-dig* hydroamination from **3b** was identified, that was consumed during the reaction to afford benzo[*g*]indole **4b**.

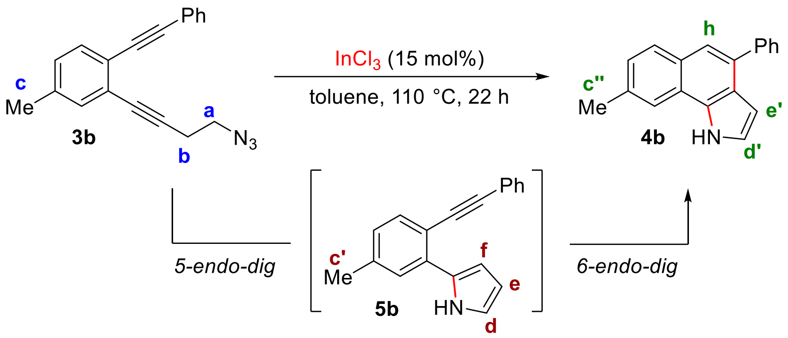

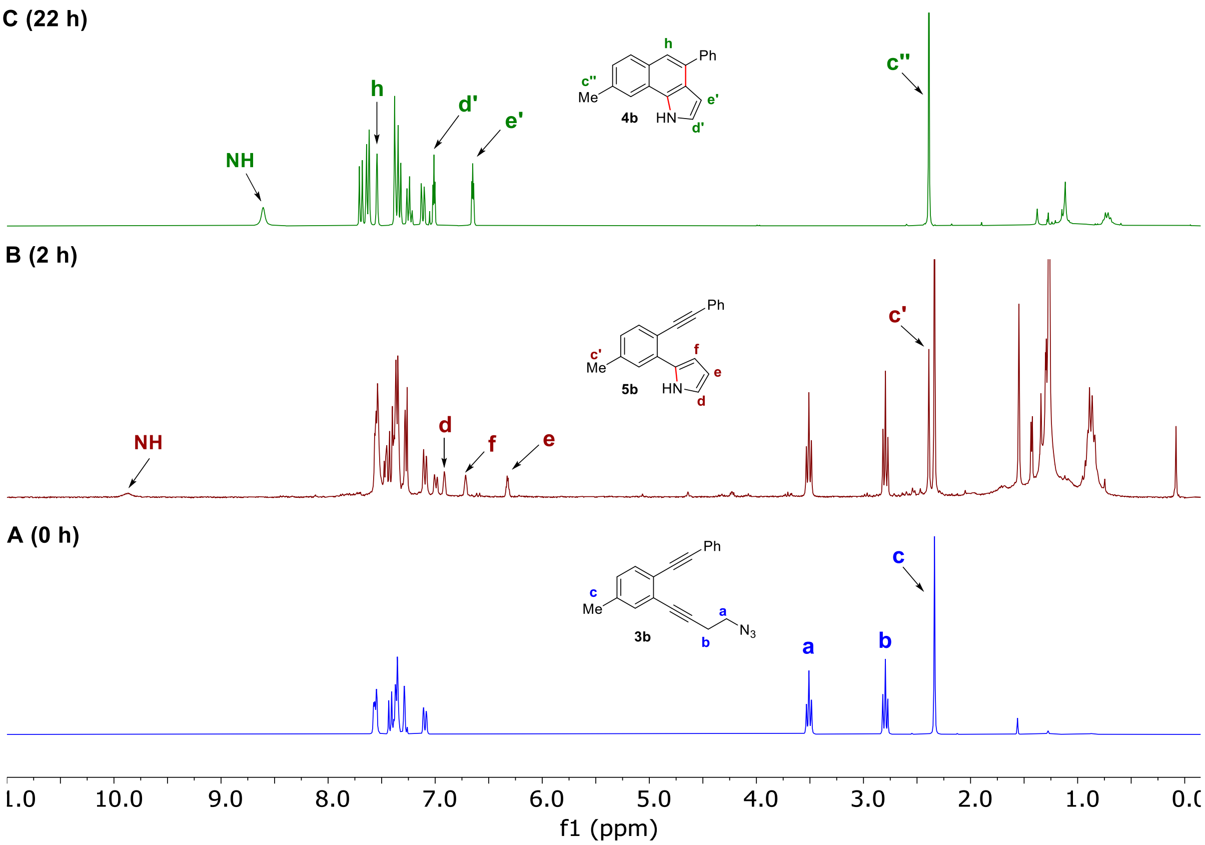

Figure S1. ^{1}H-NMR spectra of the cascade indium(III)-catalyzed cycloisomerization of azido-diyne **3b** recorded in CD_3Cl: 0 min (**A**); 2 h (**B**) and 22 h (**C**).

3. References

1 Lu, Z.; Hu, X-D.; Zhang, H.; Zhang, X-W.; Cai, J.; Usman, M.; Cong, H.; Liu, W. B. Enantioselective Assembly of Cycloenones with a Nitrile-Containing All-Carbon Quaternary Center from Malononitriles Enabled by Ni Catalysis. *J. Am. Chem. Soc*. **2020**, *142*, 7328–7333.

2 Shi, F.; Waldo, P. W.; Chen, Y.; Larock, R. C. Benzyne Click Chemistry: Synthesis of Benzotriazoles from Benzynes and Azides. *Org. Lett.* **2008**, *10,* 2409–2412.

3 Galéa, R.; Blond, G. Gold(I)-Catalyzed Domino Reaction: An Access to Furooxepines. *Adv. Synth. Catal*. **2022**, *364*, 1532–1536.

4 Points, G.L.; Stout, K. T.; Beaudry, C. M. Regioselective Formation of Substituted Indoles: Formal Synthesis of Lysergic Acid. *Chem. Eur. J.* **2020**, *26*, 16655–16658.

5 Caldarelli, S. A.; El Fangour, S.; Wein, S.; Tran van Ba, C.; Perigaud, C.; Pellet, A.; Vial, H. J.; Peyrottes, S. New Bis-thiazolium Analogues as Potential Antimalarial Agents: Design, Synthesis, and Biological Evaluation. *J. Med. Chem*. **2013**, *56*, 496–509.

6 M.; Schepmann, D.; Ametamey, S.M.; Wünsch, B. Modification of the 4-phenylbutyl side chain of potent 3-benzazepine-based GluN2B receptor antagonists. *Bio. Med. Chem*. **2019**, *27*, 3559–3567.

7 Hiroya, K.; Matsumoto, S.; Ashikawa, M.; Ogiwara, K.; Sakamoto, T. Cyclization Reactions of Homopropargyl Azide Derivatives Catalyzed by $PtCl_4$ in Ethanol Solution: Synthesis of Functionalized Pyrrole Derivatives. *Org. Lett.* **2006**, *8*, 5349–5352.

8 Yasukawa, N.; Yamada, Y., Furugen, C.; Miki, Y.; Sajiki, H.; Sawama, Y. Gold-Catalyzed Tandem Oxidative Coupling Reaction between β-Ketoallenes and Electron-Rich Arenes to 2-Furylmethylarenes. *Org. Lett.* **2021**, *23,* 5891–5895.

9 Wang, T.; Jiang, Y.; Wang, Y.; Yan, R. Fe-Catalyzed tandem cyclization for the synthesis of 3-nitrofurans from homopropargylic alcohols and $Al(NO_3)_3·9H_2O$. *Org. Biomol. Chem*. **2018**, *16*, 5232–5235.

10 Arndt, S.; Hansmann, M. M.; Motloch, P.; Rudolph, M.; Rominger, F.; Hashmi, A. S. K. Intramolecular *anti*-Phosphinoauration of Alkynes: An FLP-Motivated Approach to Stable Aurated Phosphindolium Complexes. *Chem. Eur. J.* **2017**, *23,* 2542–2547.

11 Chen, M.; Su, N.; Deng, T.; Wink, D. J.; Zhao, Y.; Driver, T. G. Controlling the Selectivity Patterns of Au-Catalyzed Cyclization–Migration Reactions. *Org. Lett*. **2019**, *21,* 1555–1558.

12 Naveen, K.; Perumal, P. T.; Cho, D-H. Domino Palladium-Catalyzed Double Norbornene Insertion/Annulation Reaction: Expeditious Synthesis of Overcrowded Tetrasubstituted Olefins. *Org. Lett*. **2019**, *21*, 4350–4354.

13. Morishita, T.; Yoshida, H.; Ohshita, J. Copper-Catalysed Bromoalkynylation of Arynes. *Chem. Comm*. **2010**, *46*, 640–642.

14. Shaikh, A. C.; Ranade, D. S.; Rajamohanan, P. R.; Kulkarni, P. P.; Patil, N. T. Oxidative Intramolecular 1,2-Amino-Oxygenation of Alkynes under Au^{I}/Au^{III} Catalysis: Discovery of a Pyridinium-Oxazole Dyad as an Ionic Fluorophore. *Angew. Chem. Int. Ed*. **2017**, *56*, 757–761.

15. Watanabe, T.; Abe, H.; Mutoh, Y.; Saito, S. Ruthenium-Catalyzed Cycloisomerization of 2-Alkynylstyrenes via 1,2-Carbon Migration That Leads to Substituted Naphthalenes. *Chem. Eur. J.* **2018**, *24*, 11545–11549.

16 Hirano, K.; Inaba, Y.; Takahashi, N.; Shimano, M.; Oishi, S.; Fujii, N.; Ohno, H. Direct Synthesis of Fused Indoles by Gold-Catalyzed Cascade Cyclization of Diynes. *J. Org. Chem*. **2011**, *76*, 1212–1227.

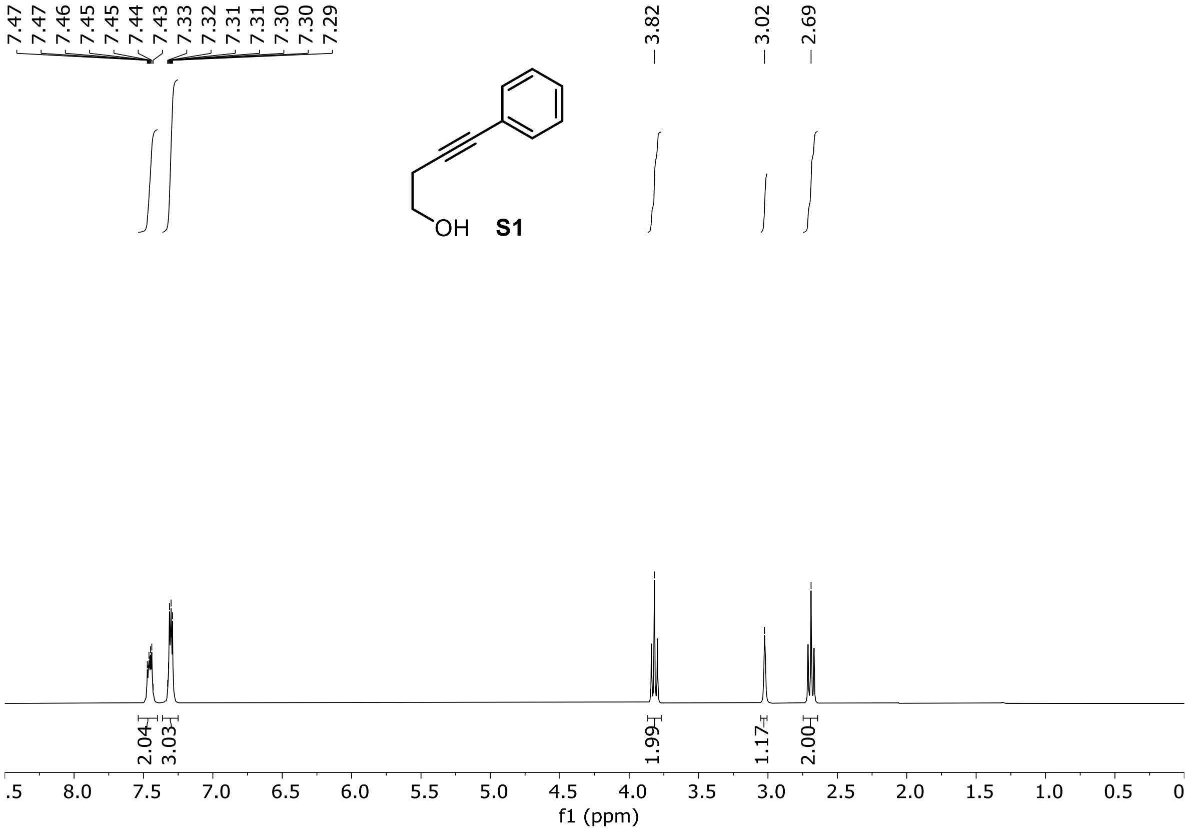

Figure S2. ^{1}H NMR spectrum of **S1** ($CDCl_3$, 300 MHz, 298 K)

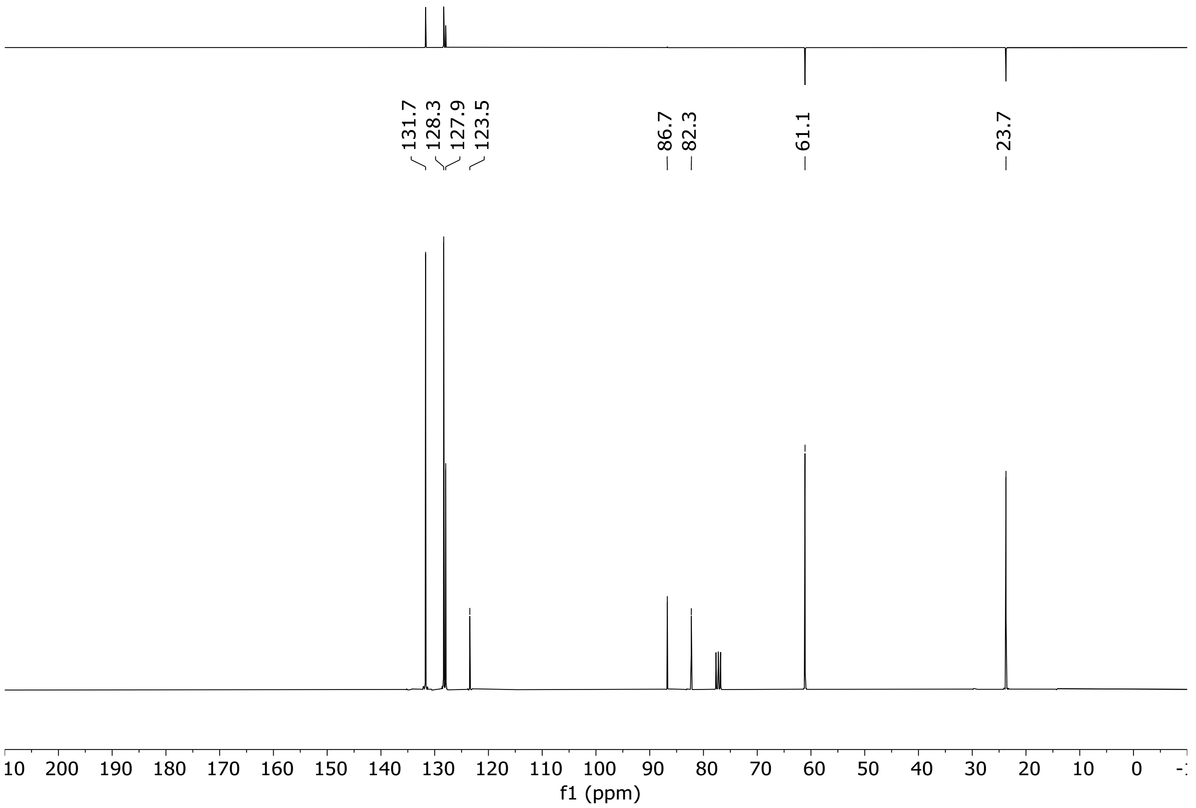

Figure S3. $^{13}C\{1H\}$ NMR spectrum of **S1** ($CDCl_3$, 75 MHz, 298 K)

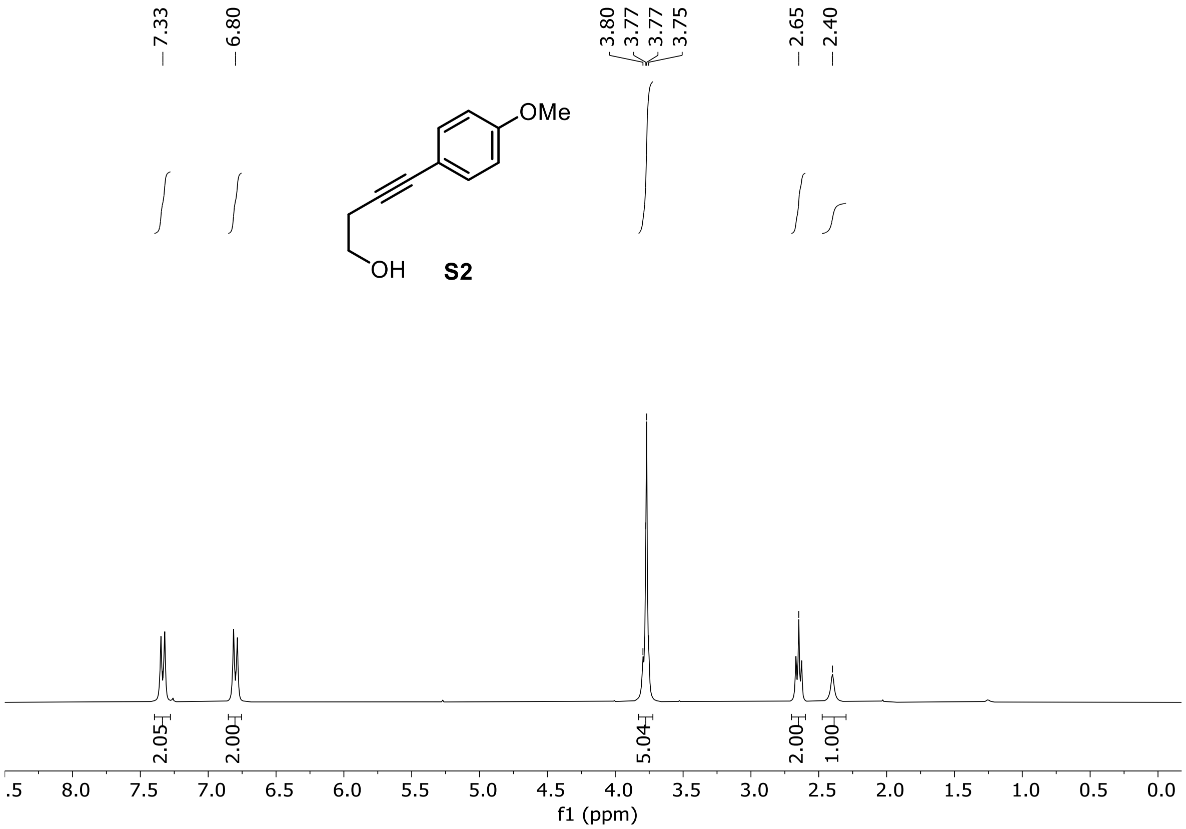

Figure S4. ^{1}H NMR spectrum of **S2** ($CDCl_3$, 300 MHz, 298 K)

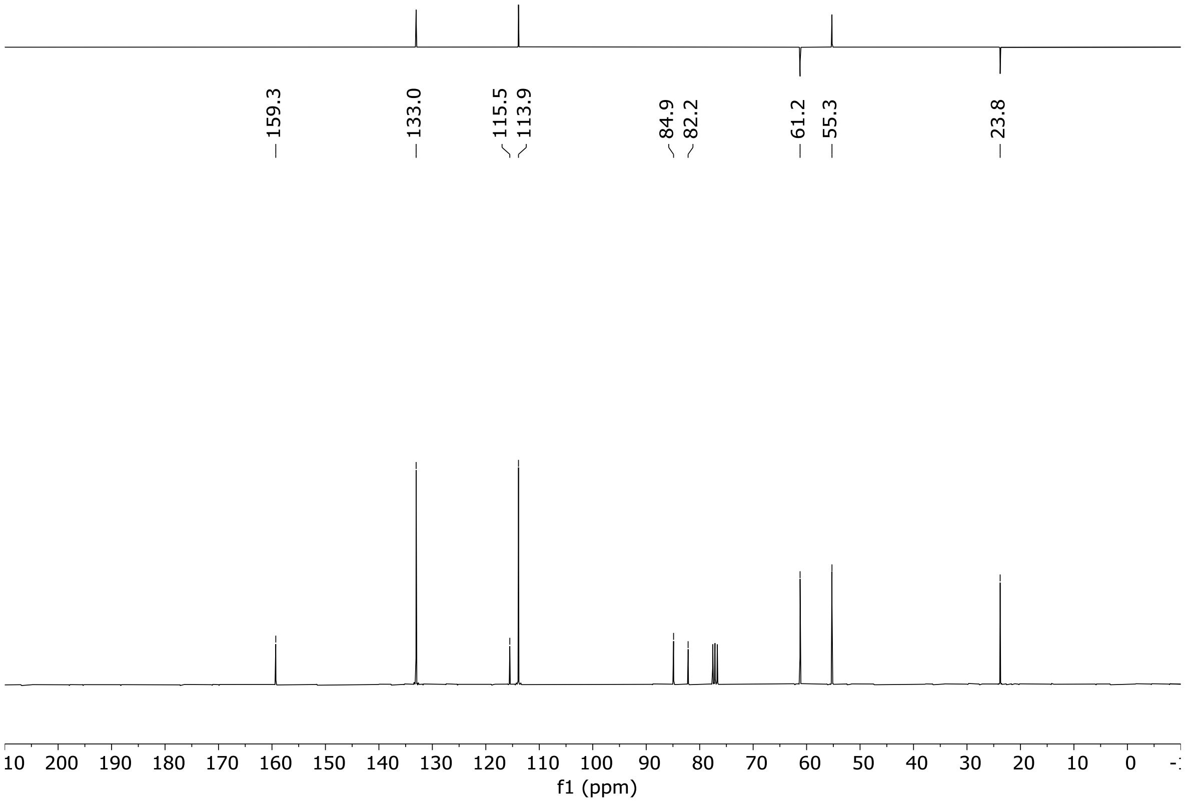

Figure S5. $^{13}C\{1H\}$ NMR spectrum of **S2** ($CDCl_3$, 75 MHz, 298 K)

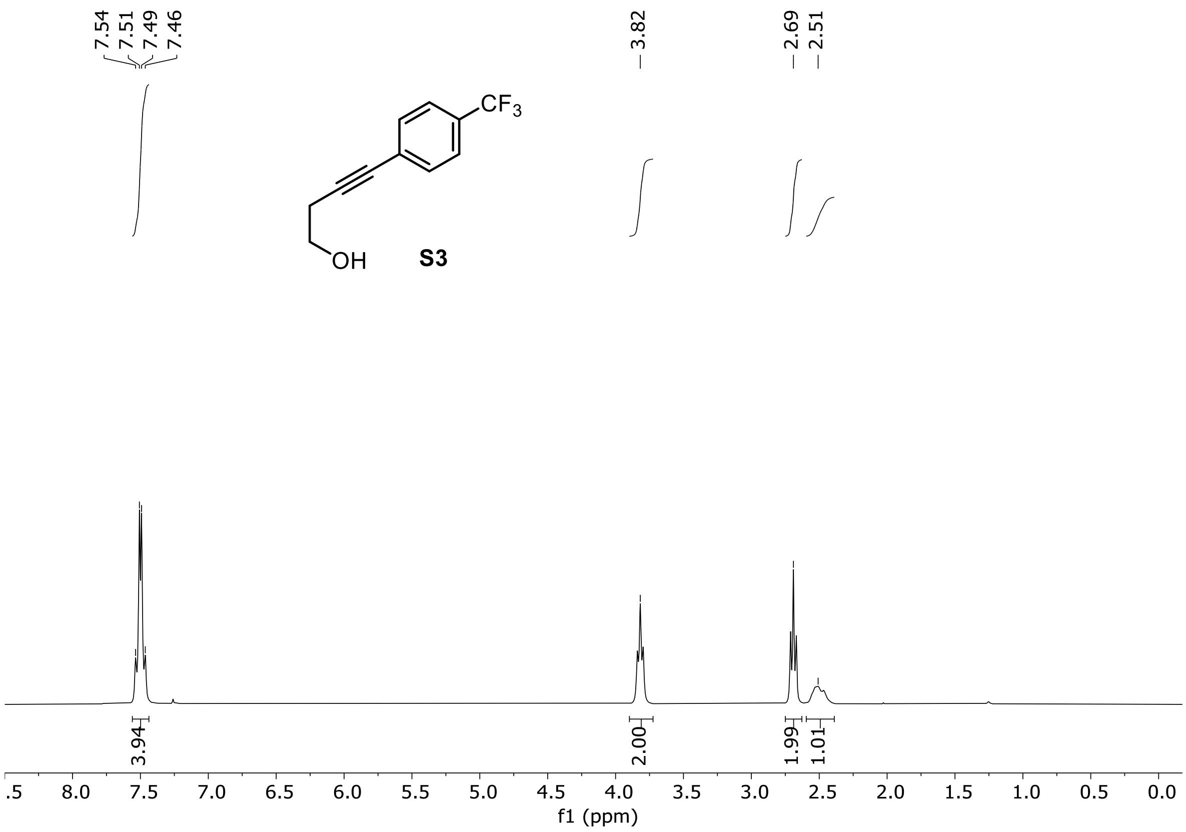

Figure S6. ^{1}H NMR spectrum of **S3** ($CDCl_3$, 300 MHz, 298 K)

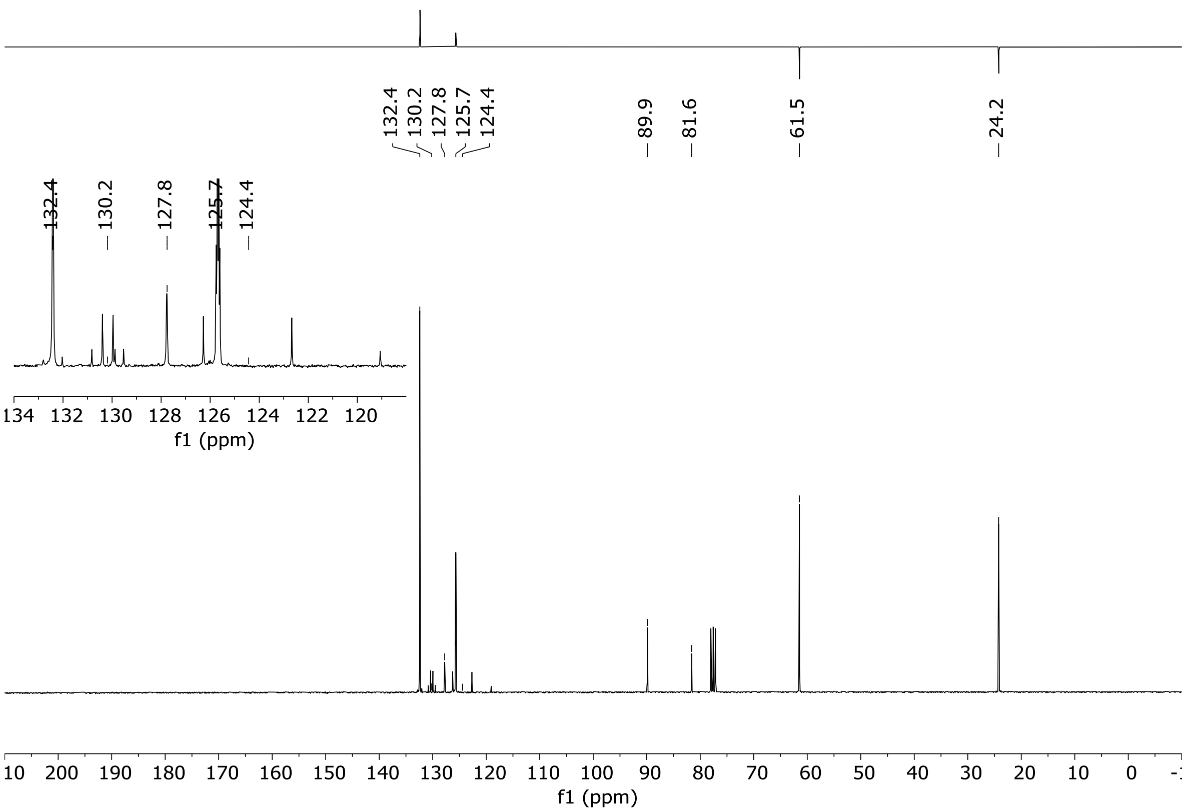

Figure S7. $^{13}C\{1H\}$ NMR spectrum of **S3** ($CDCl_3$, 75 MHz, 298 K)

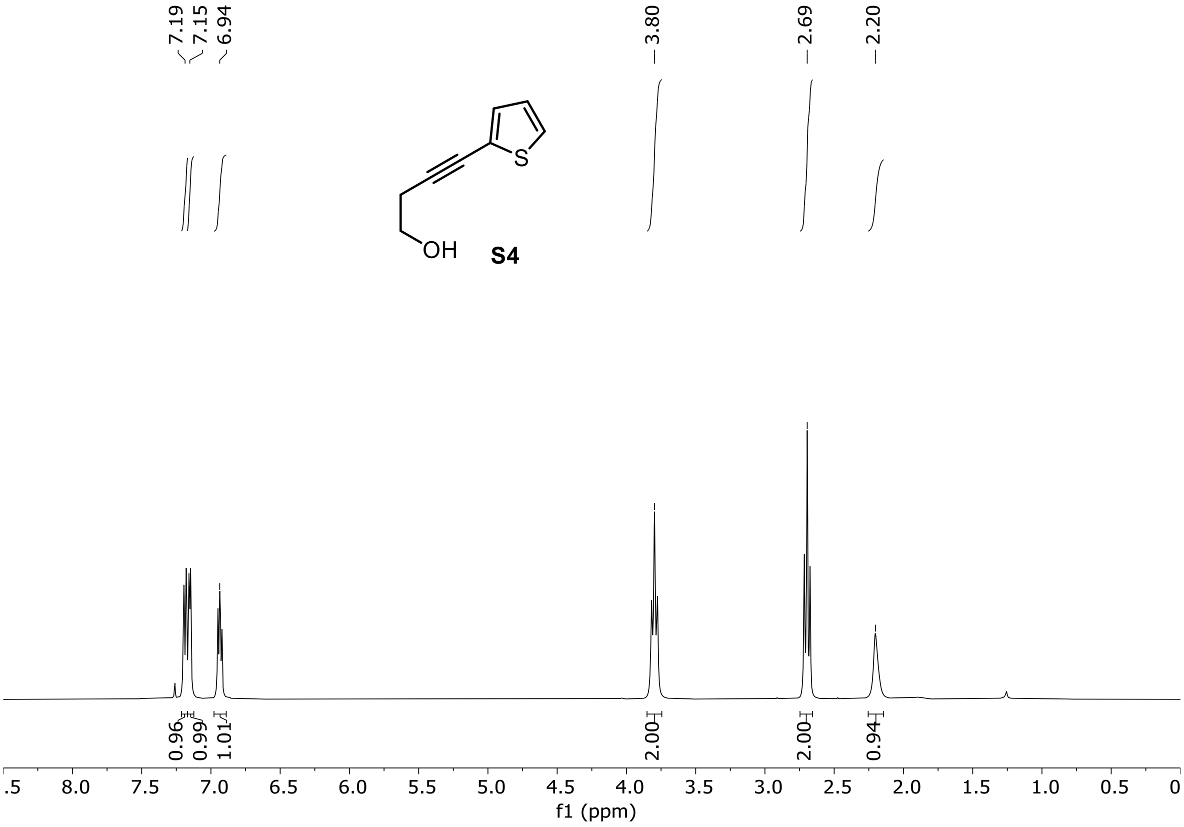

Figure S8. ^{1}H NMR spectrum of **S4** ($CDCl_3$, 300 MHz, 298 K)

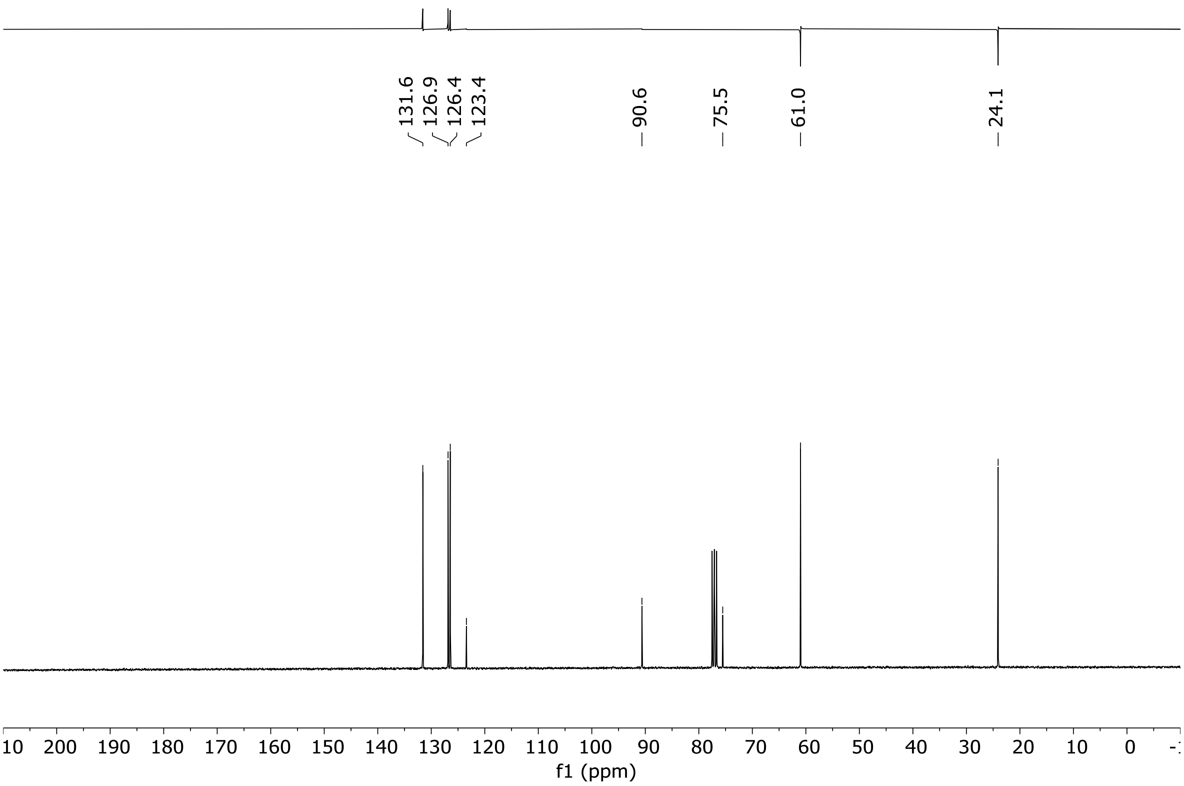

Figure S9. ^{13}C{1H} NMR spectrum of **S4** ($CDCl_3$, 75 MHz, 298 K)

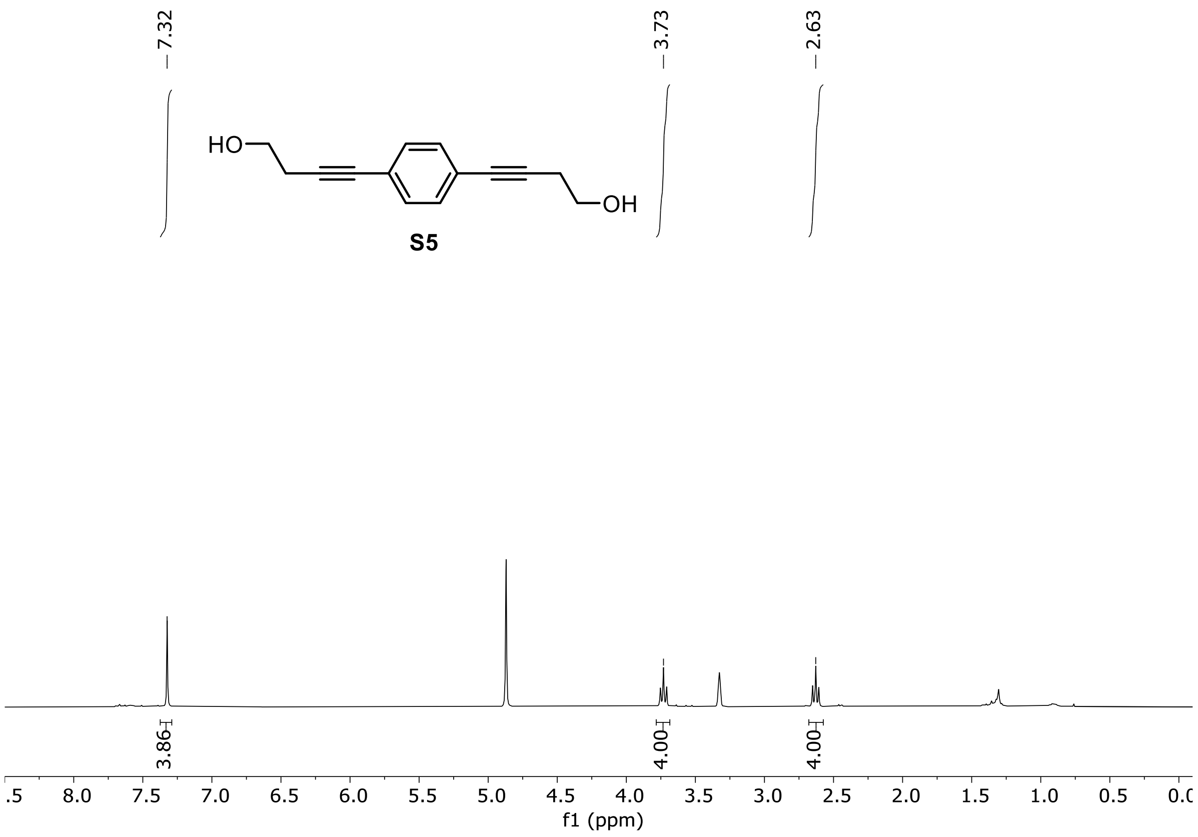

Figure S10. ^{1}H NMR spectrum of **S5** (Methanol-d_4, 300 MHz, 298 K)

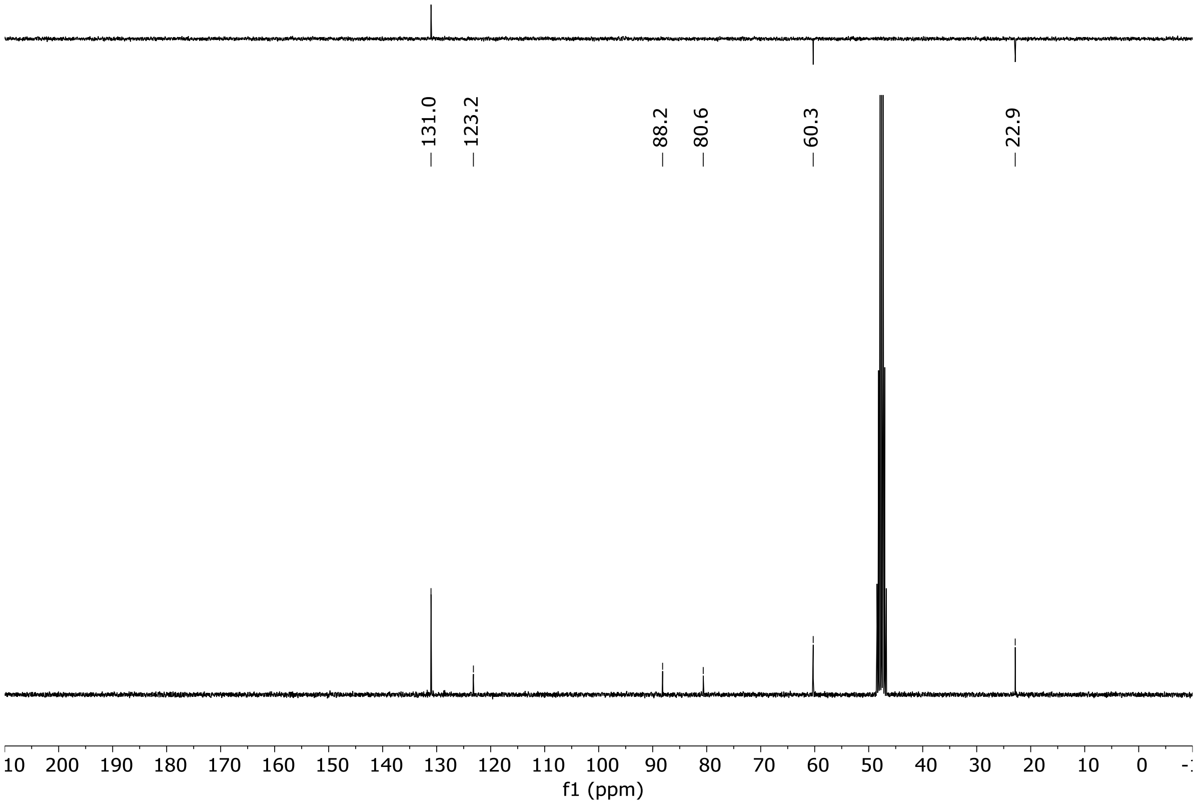

Figure S11. ^{13}C{1H} NMR spectrum of **S5** (Methanol-d_4, 75 MHz, 298 K)

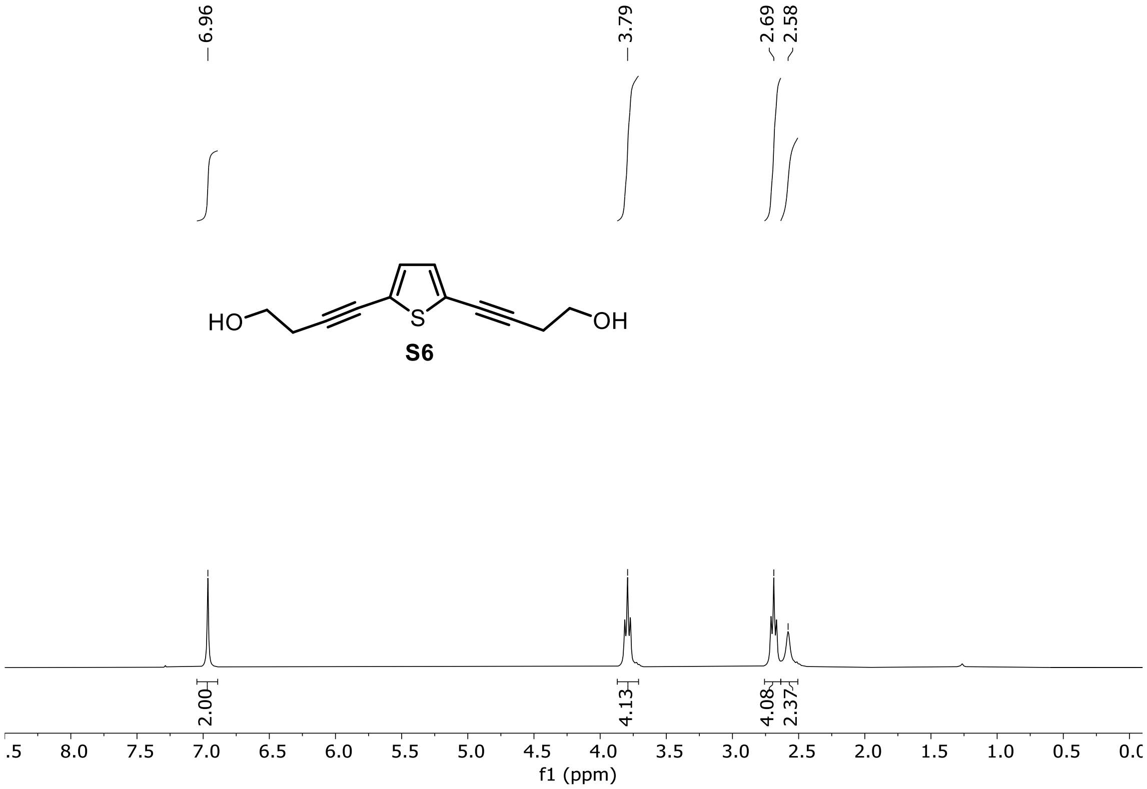

Figure S12. ^{1}H NMR spectrum of **S6** ($CDCl_3$, 300 MHz, 298 K)

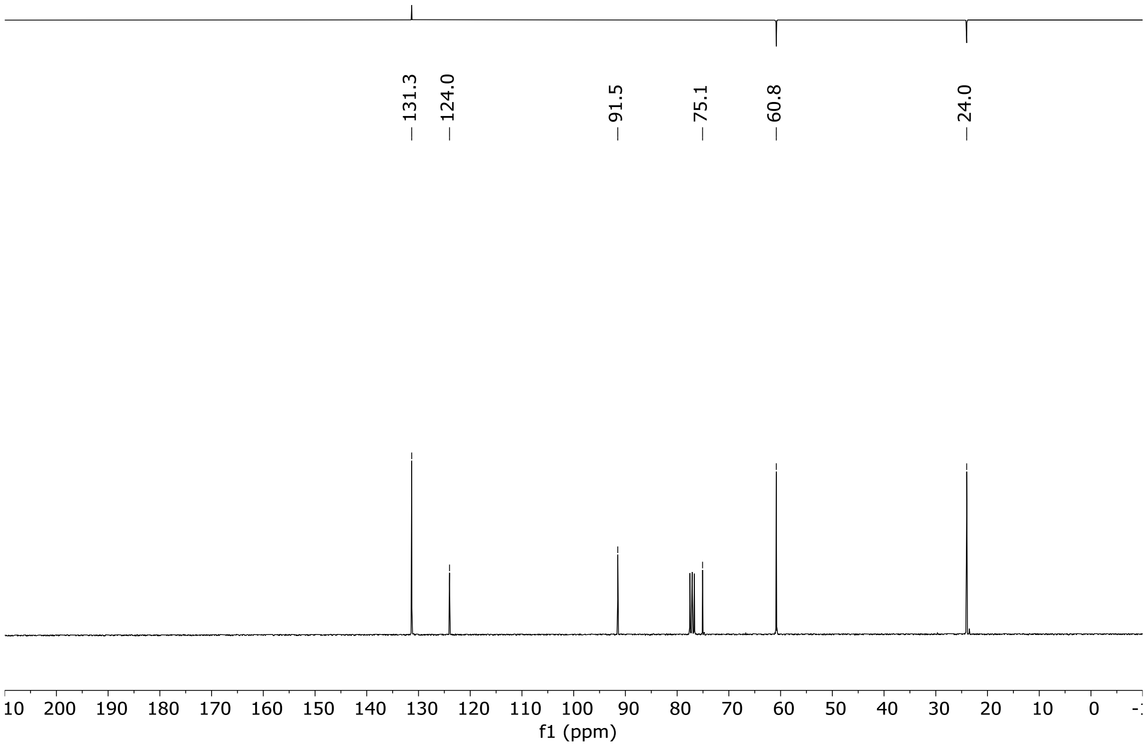

Figure S13. $^{13}C\{1H\}$ NMR spectrum of **S6** ($CDCl_3$, 75 MHz, 298 K)

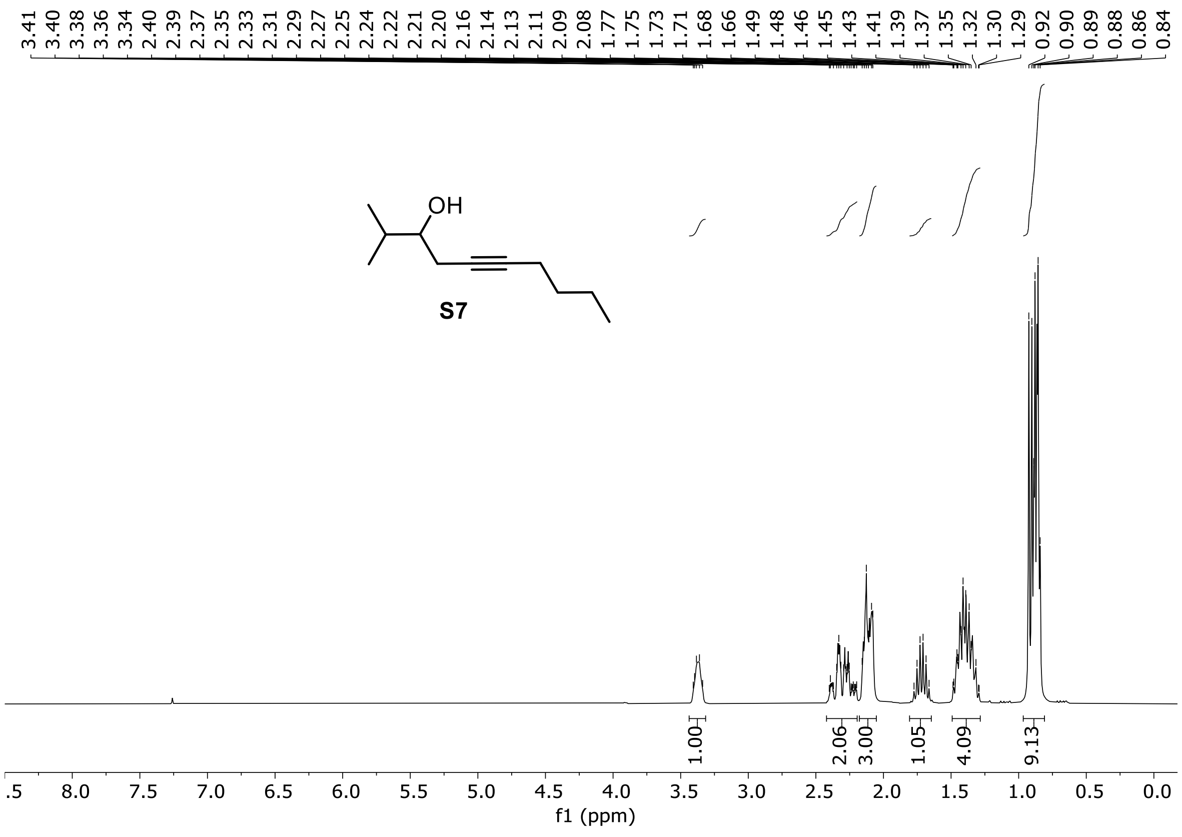

Figure S14. ^{1}H NMR spectrum of **S7** ($CDCl_3$, 300 MHz, 298 K)

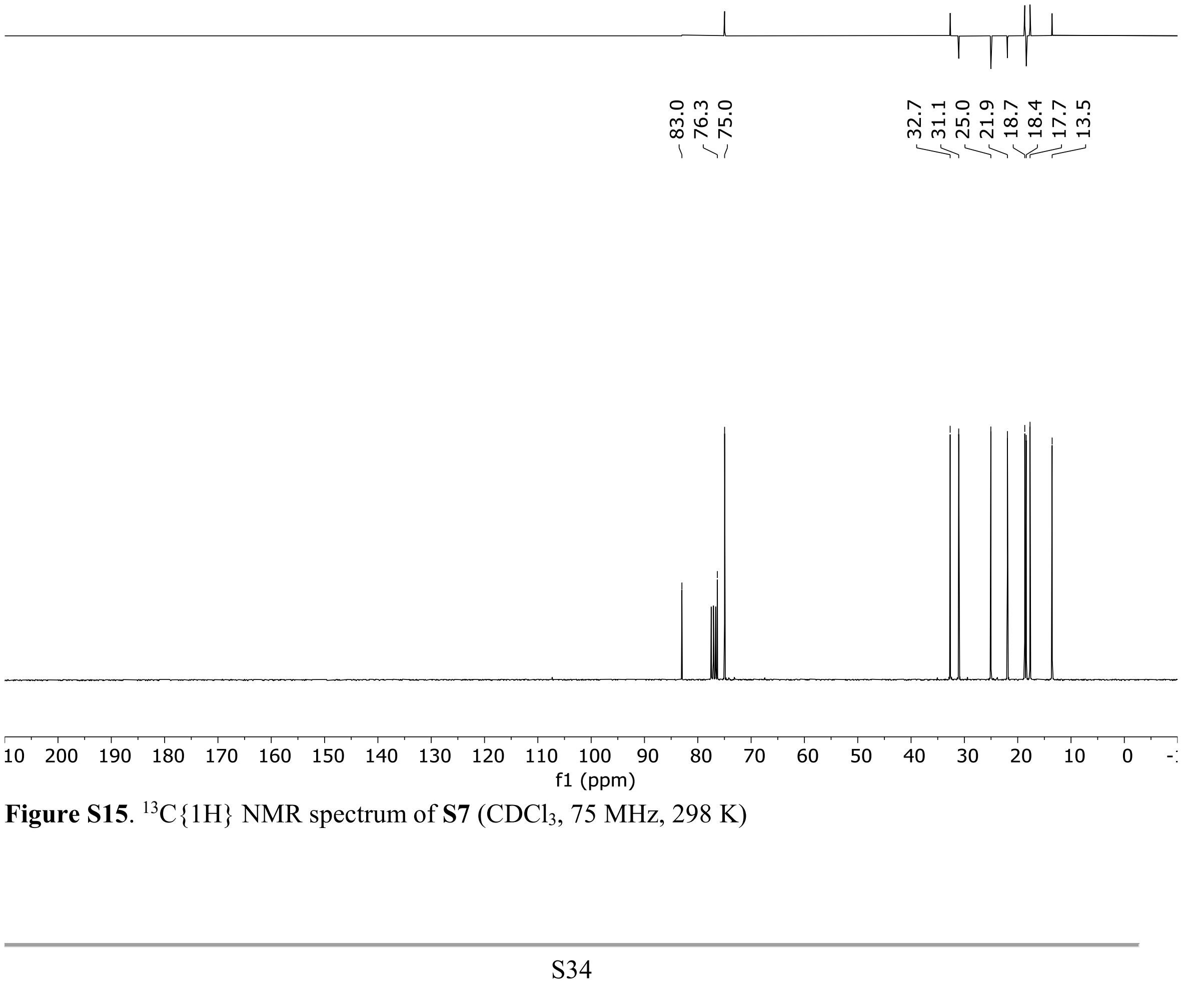

Figure S15. $^{13}C\{1H\}$ NMR spectrum of **S7** ($CDCl_3$, 75 MHz, 298 K)

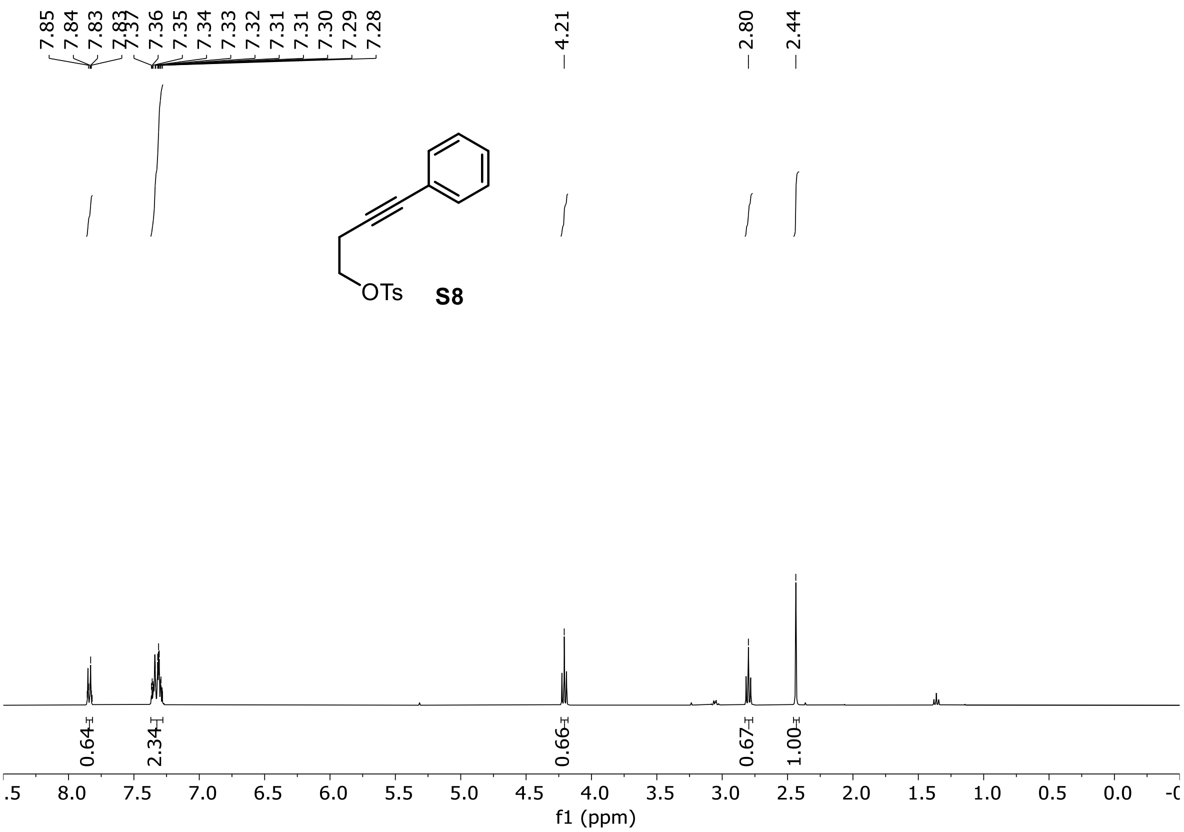

Figure S16. ^{1}H NMR spectrum of **S8** ($CDCl_3$, 300 MHz, 298 K)

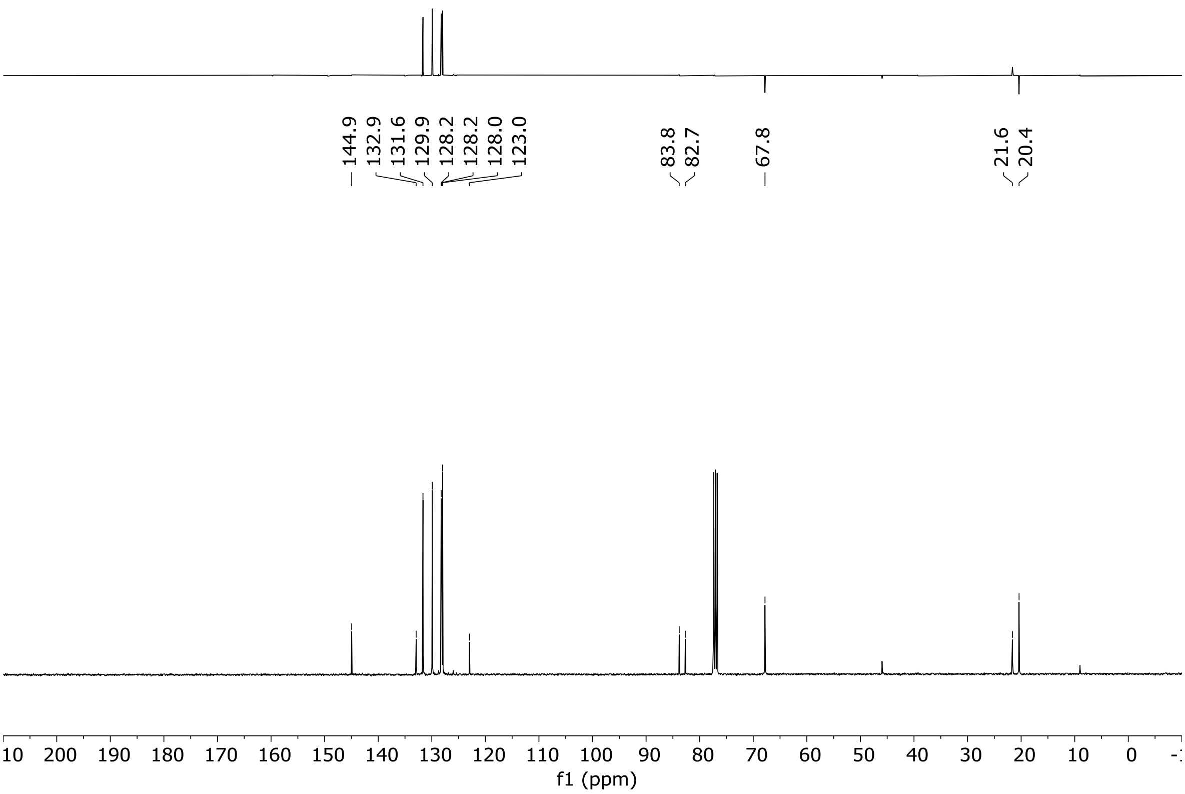

Figure S17. ^{13}C{1H} NMR spectrum of **S8** ($CDCl_3$, 75 MHz, 298 K)

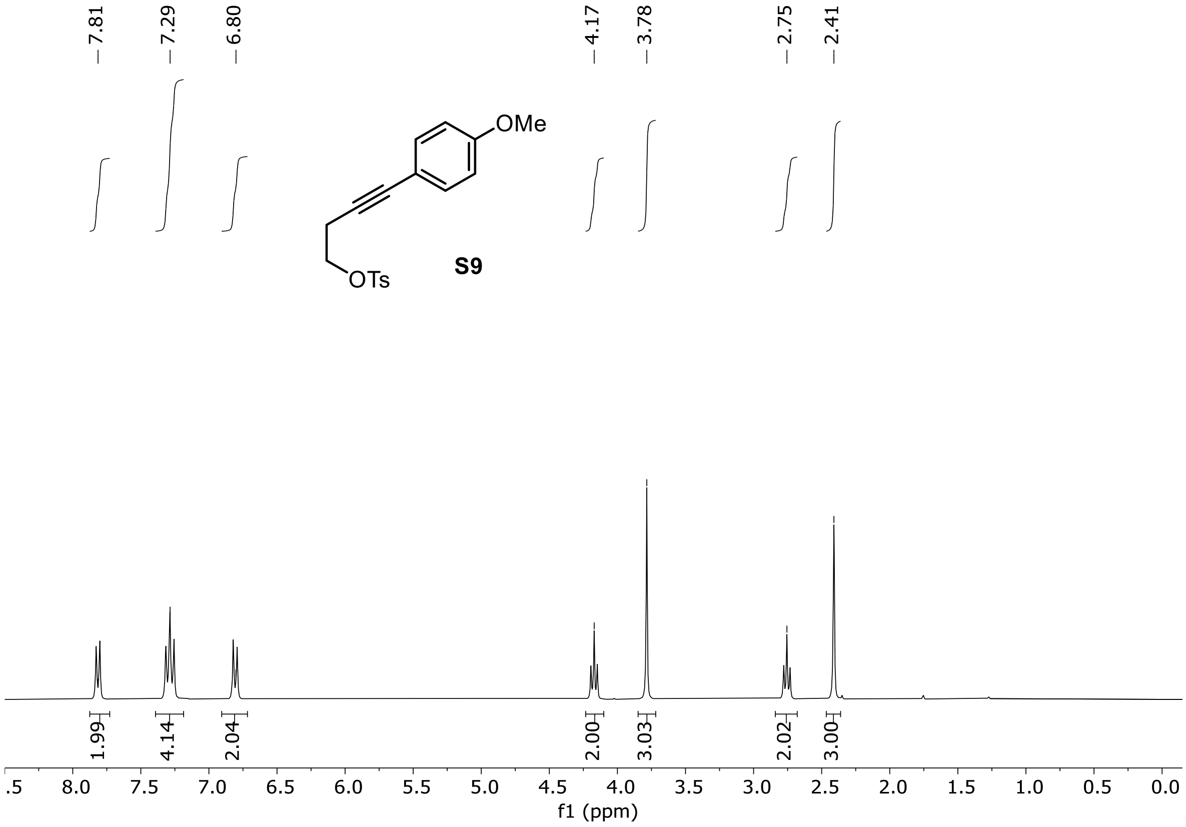

Figure S18. ^{1}H NMR spectrum of **S9** ($CDCl_3$, 300 MHz, 298 K)

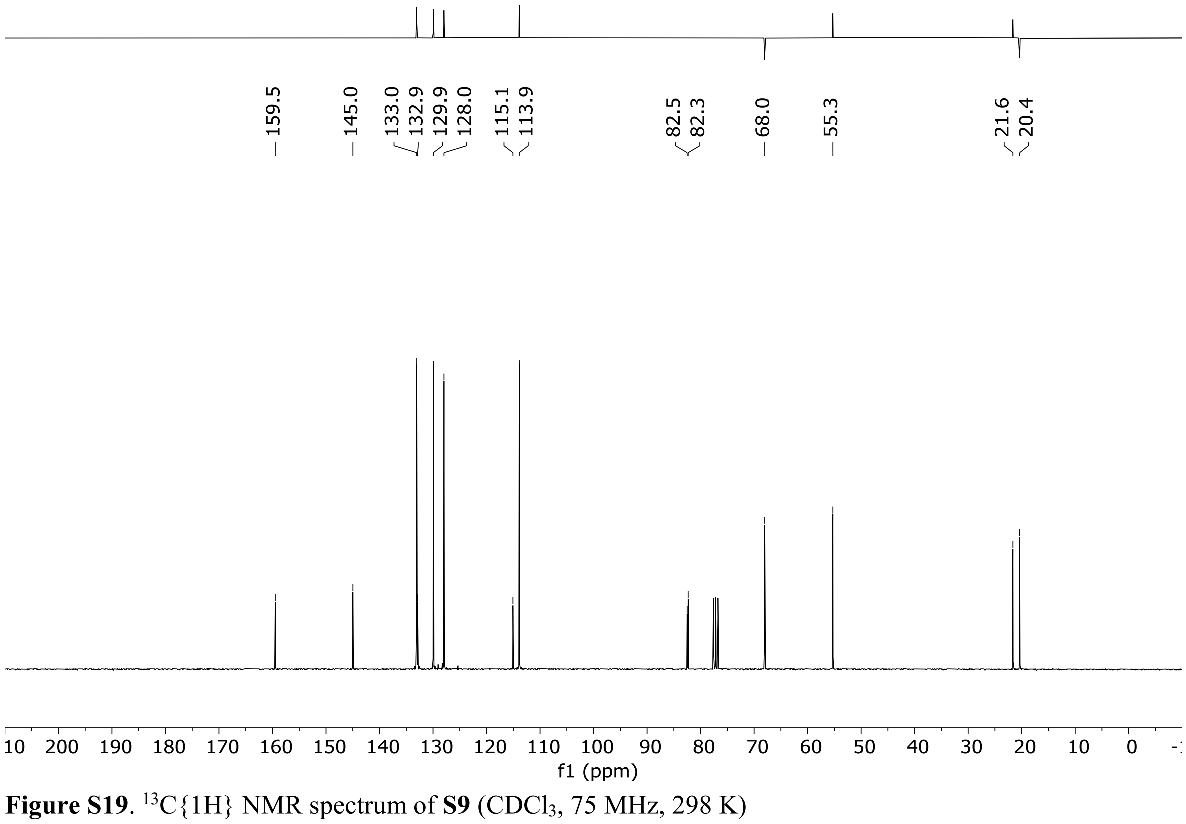

Figure S19. $^{13}C\{1H\}$ NMR spectrum of **S9** ($CDCl_3$, 75 MHz, 298 K)

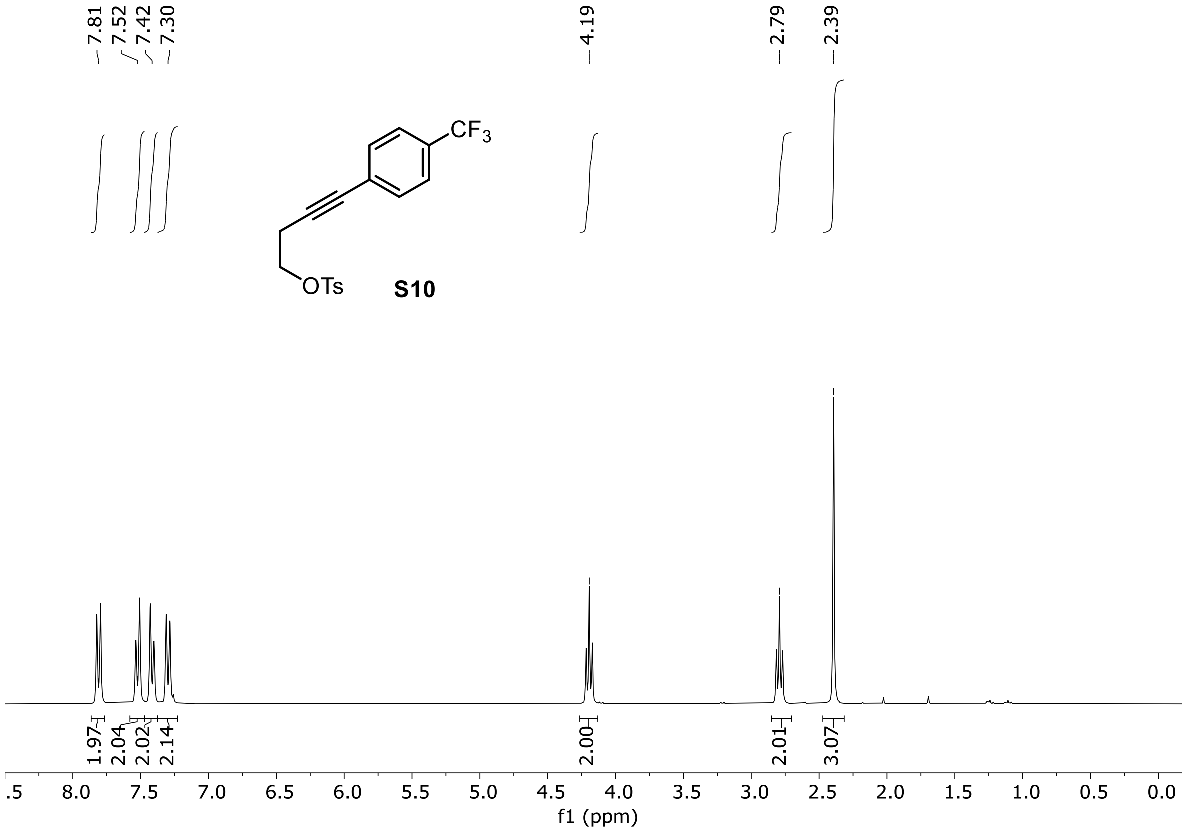

Figure S20. ^{1}H NMR spectrum of **S10** ($CDCl_3$, 300 MHz, 298 K)

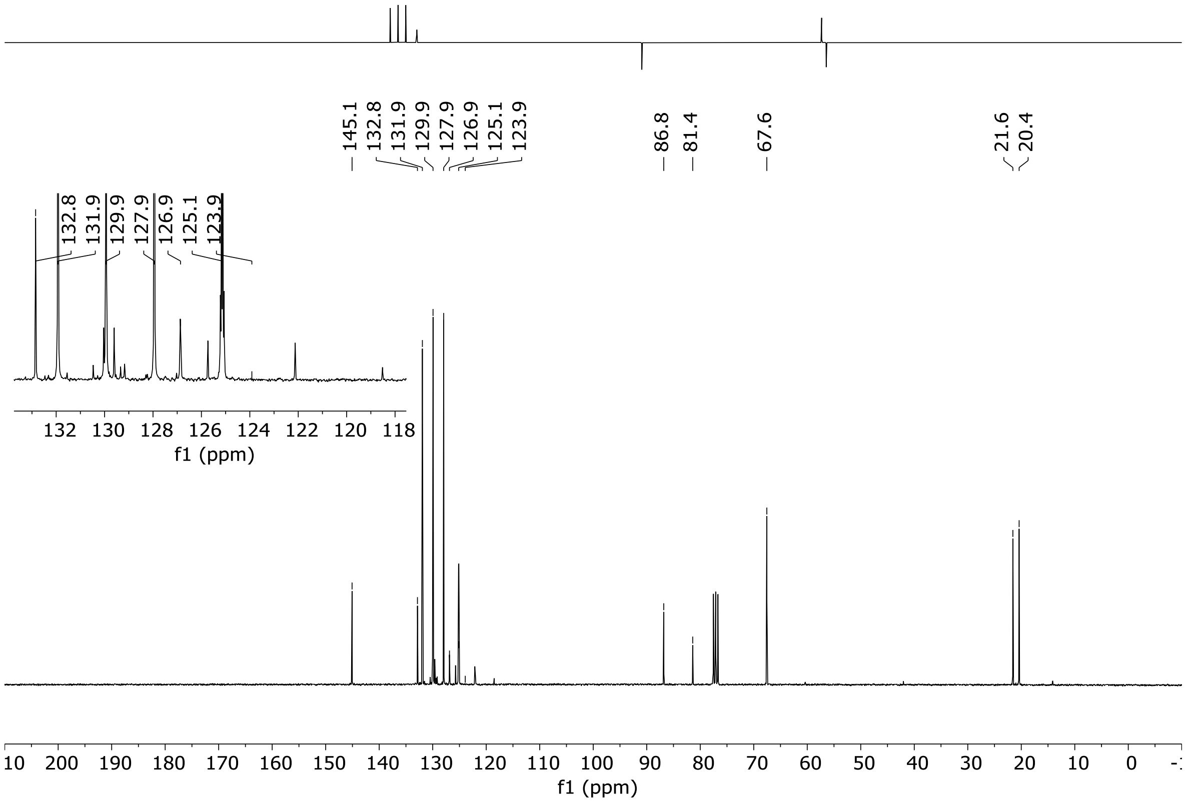

Figure S21. $^{13}C\{1H\}$ NMR spectrum of **S10** ($CDCl_3$, 75 MHz, 298 K)

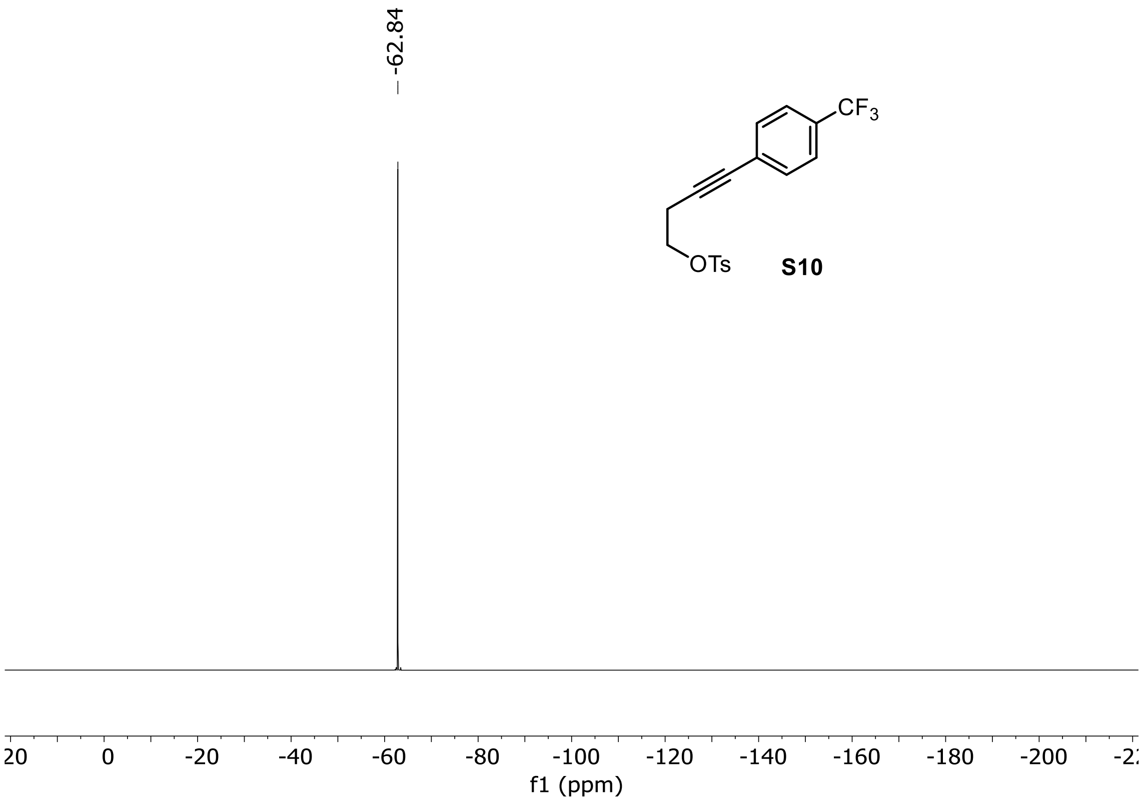

Figure S22. ^{19}F NMR spectrum of **S10** ($CDCl_3$, 285 MHz, 298 K)

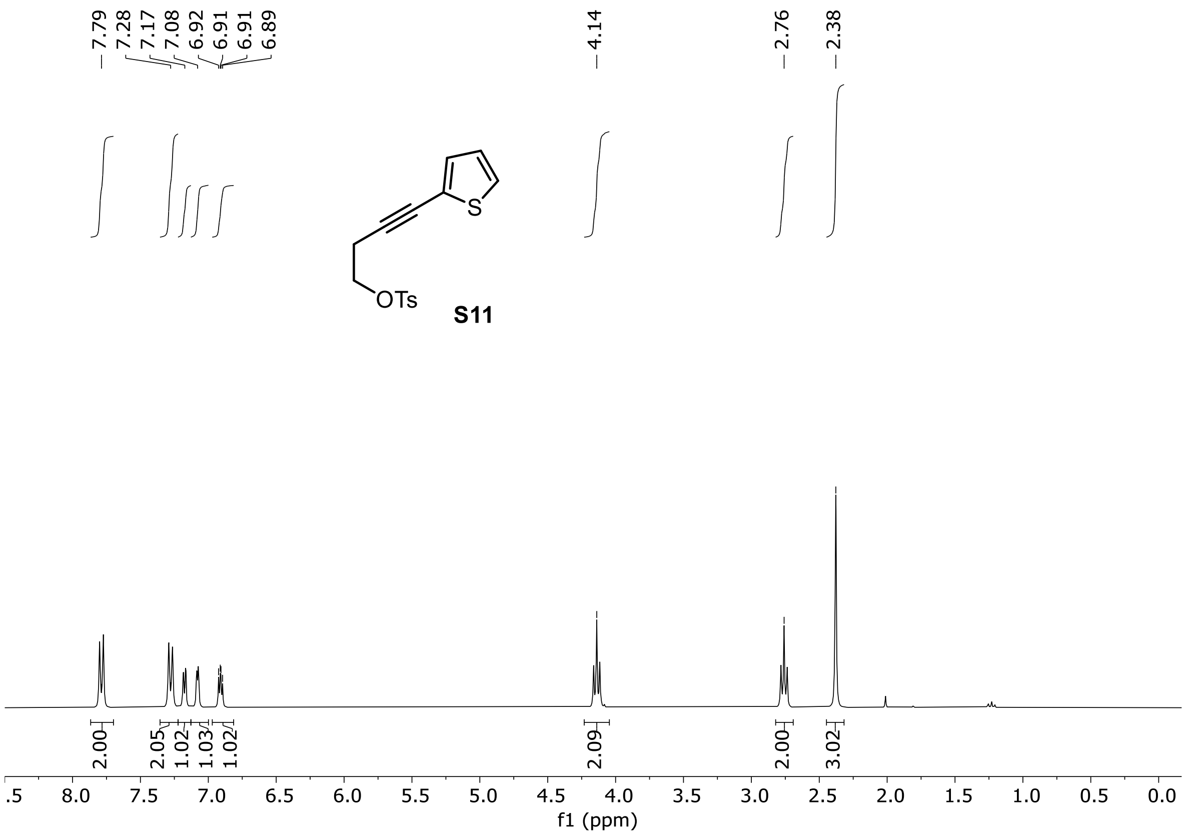

Figure S23. ^{1}H NMR spectrum of **S11** ($CDCl_3$, 300 MHz, 298 K)

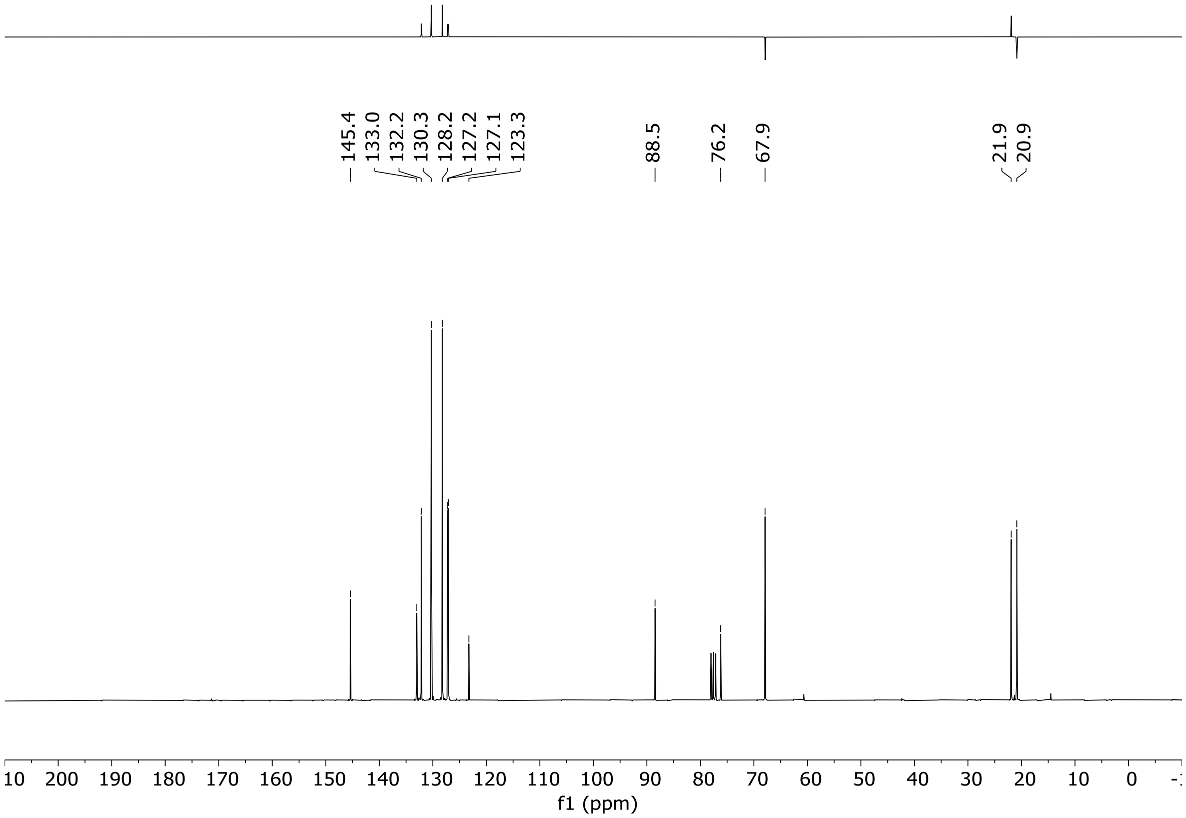

Figure S24. ^{13}C{1H} NMR spectrum of **S11** ($CDCl_3$, 75 MHz, 298 K)

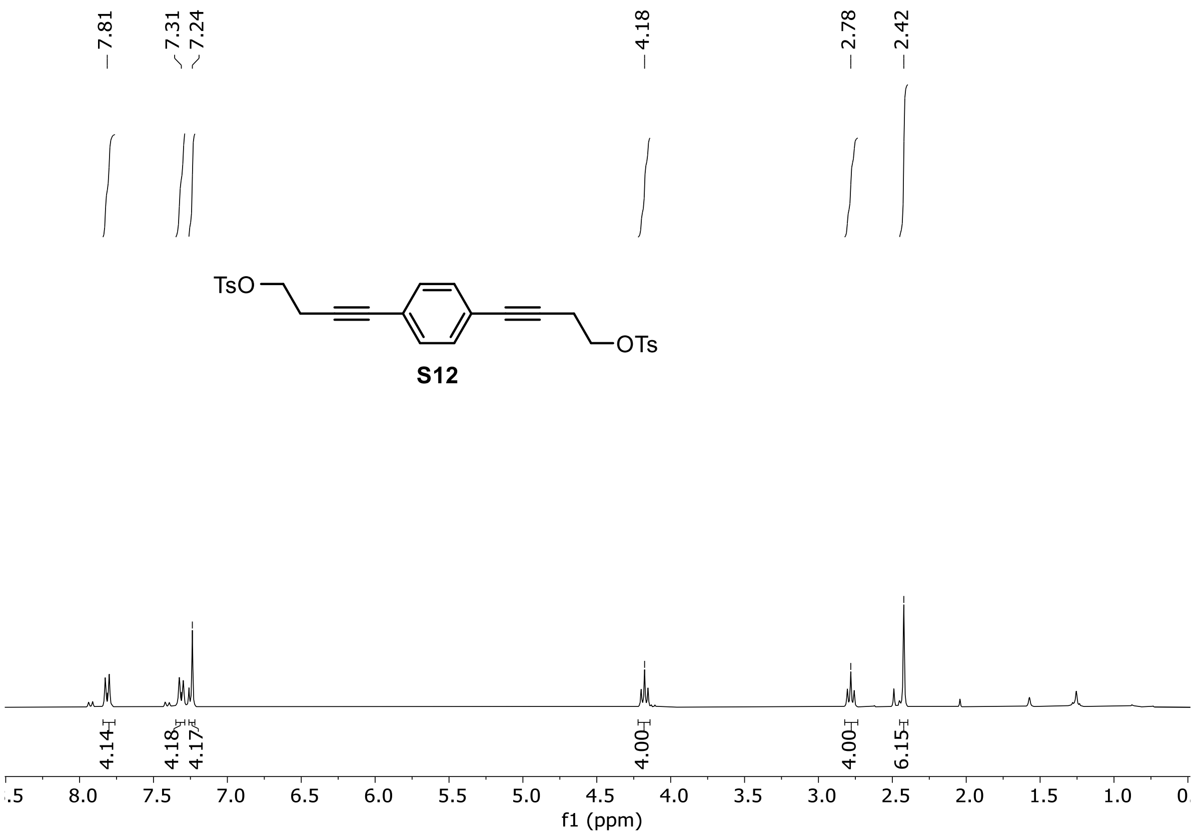

Figure S25. ^{1}H NMR spectrum of **S12** ($CDCl_3$, 300 MHz, 298 K)

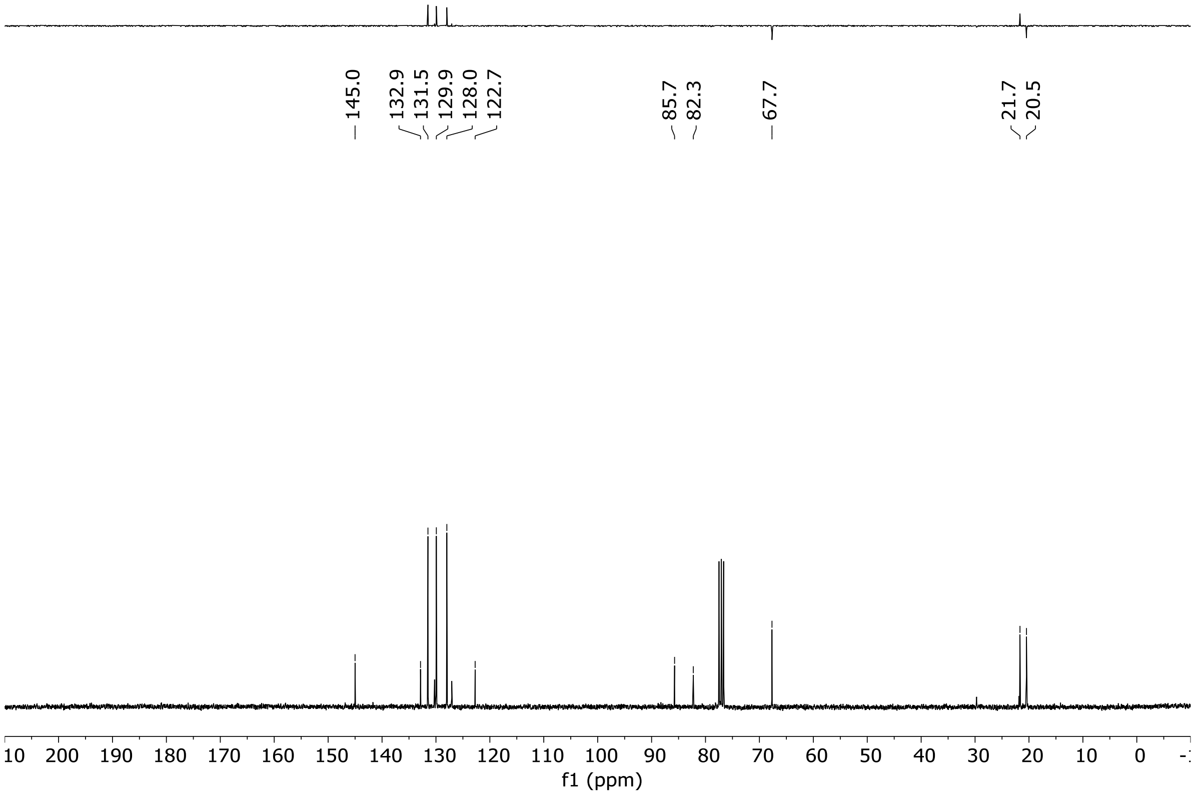

Figure S26. $^{13}C\{1H\}$ NMR spectrum of **S12** ($CDCl_3$, 75 MHz, 298 K)

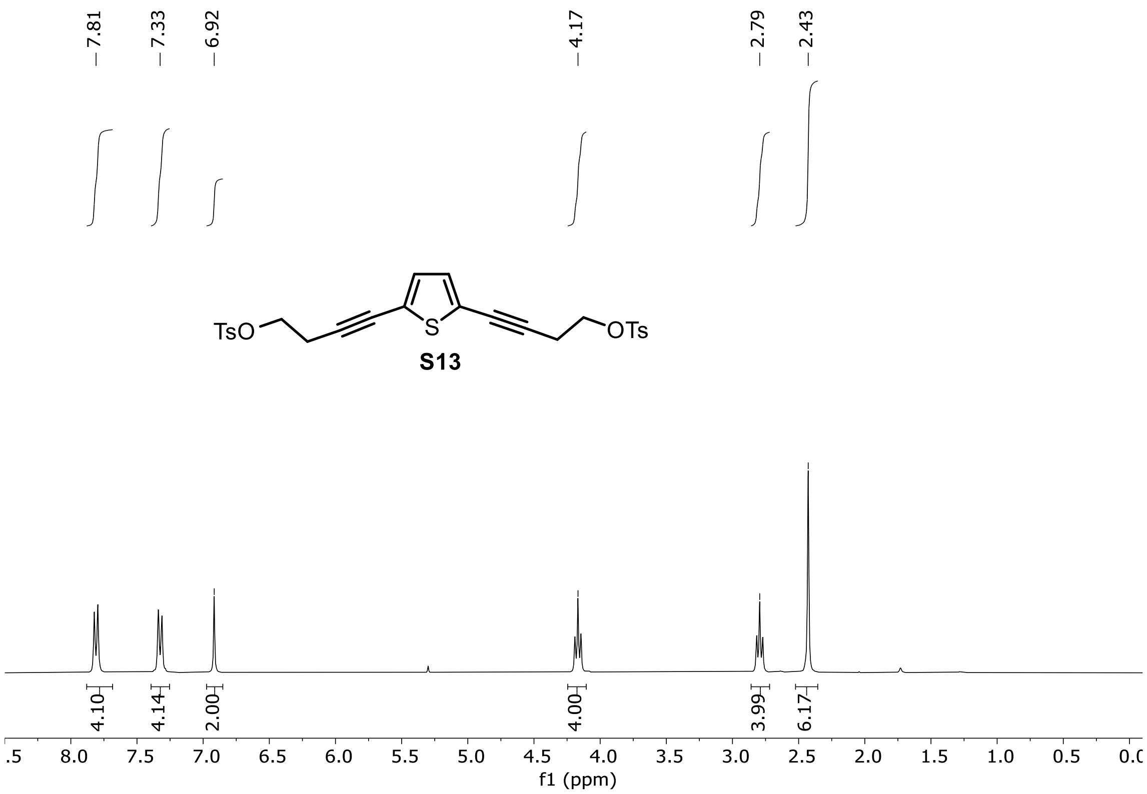

Figure S27. ^{1}H NMR spectrum of **S13** ($CDCl_3$, 300 MHz, 298 K)

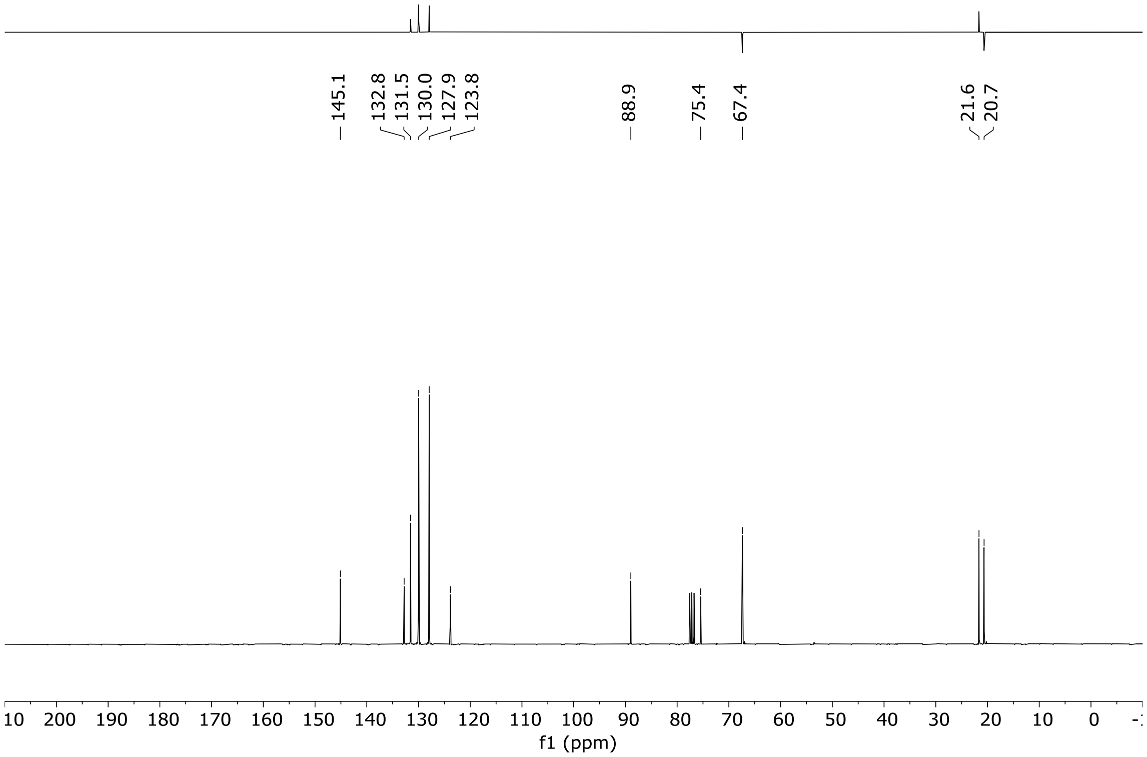

Figure S28. ^{13}C{1H} NMR spectrum of **S13** ($CDCl_3$, 75 MHz, 298 K)

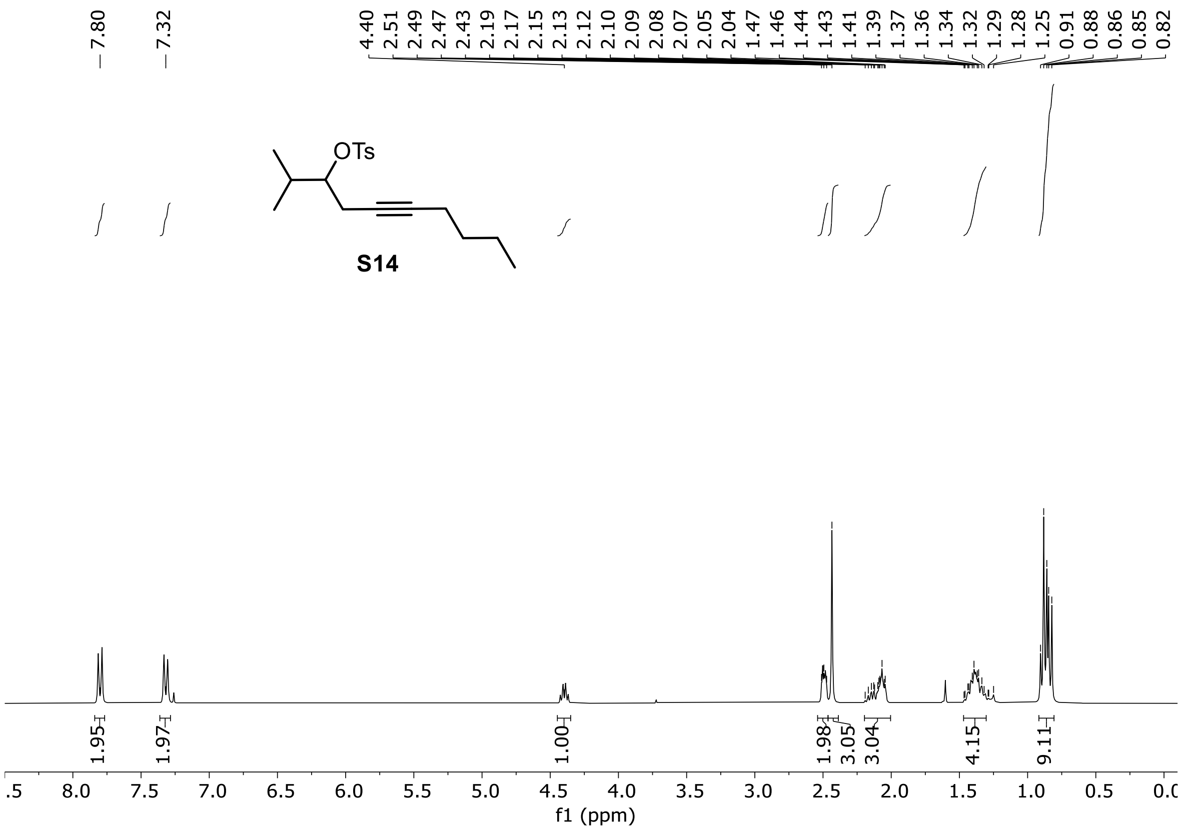

Figure S29. ^{1}H NMR spectrum of **S14** ($CDCl_3$, 300 MHz, 298 K)

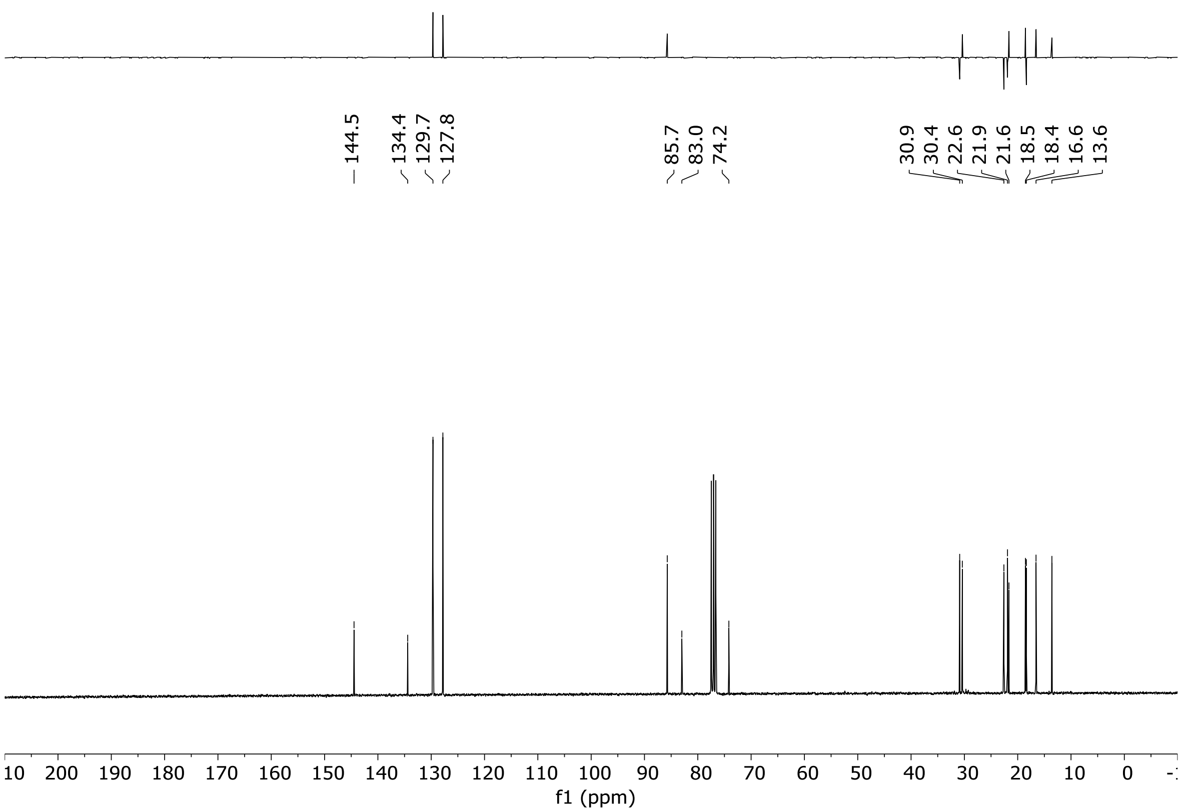

Figure S30. $^{13}C\{1H\}$ NMR spectrum of **S14** ($CDCl_3$, 75 MHz, 298 K)

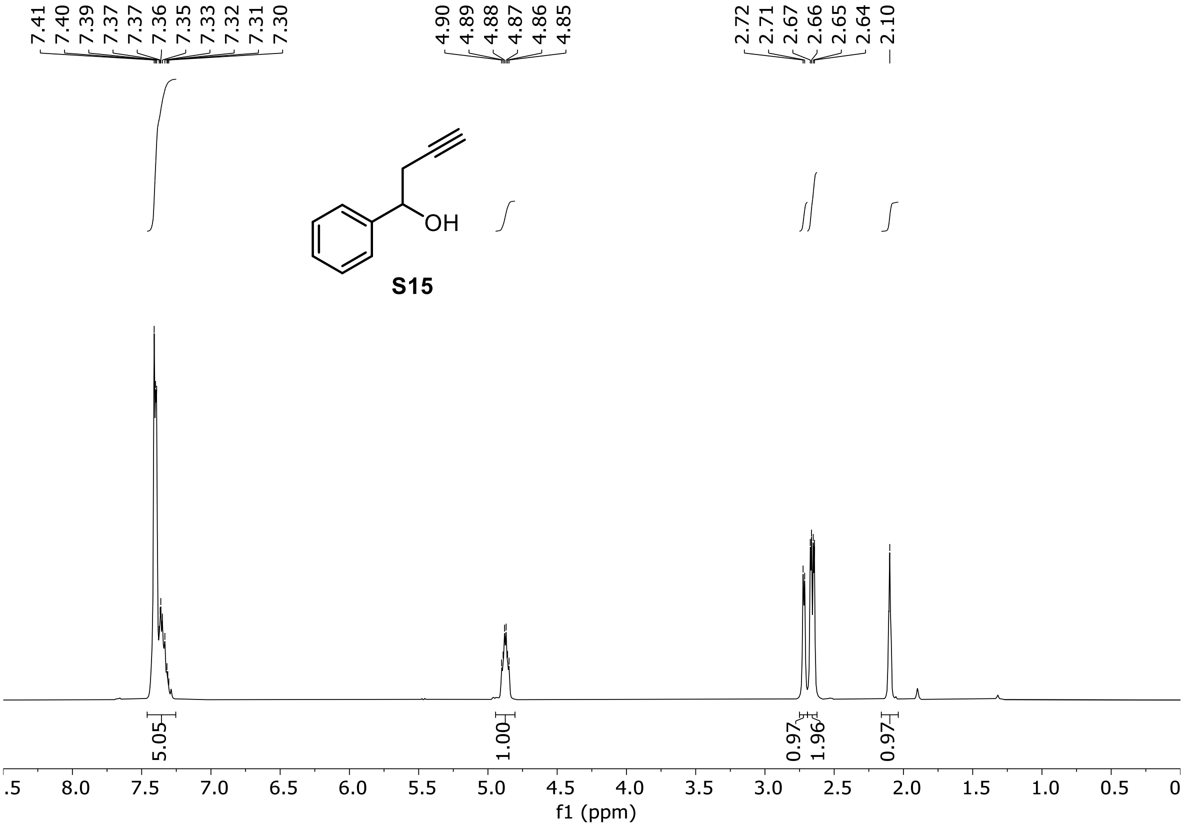

Figure S31. ^{1}H NMR spectrum of **S15** ($CDCl_3$, 300 MHz, 298 K)

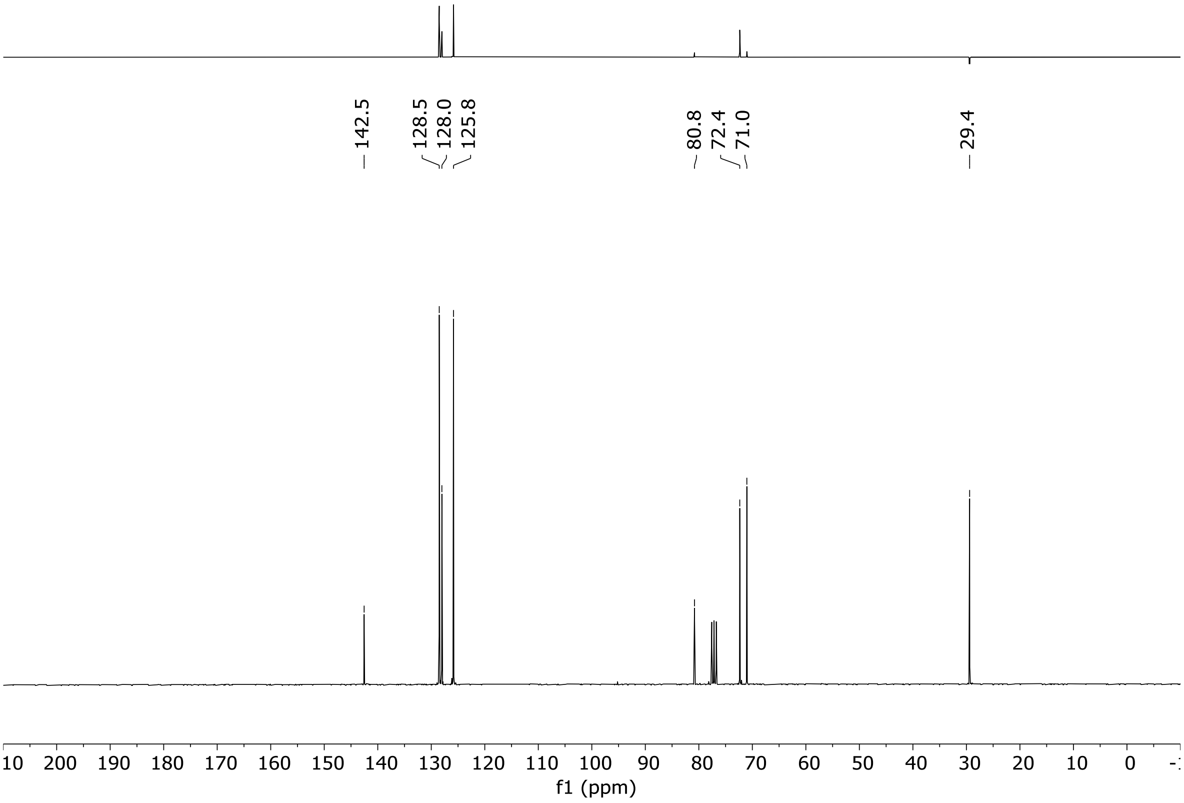

Figure S32. ^{13}C{1H} NMR spectrum of **S15** ($CDCl_3$, 75 MHz, 298 K)

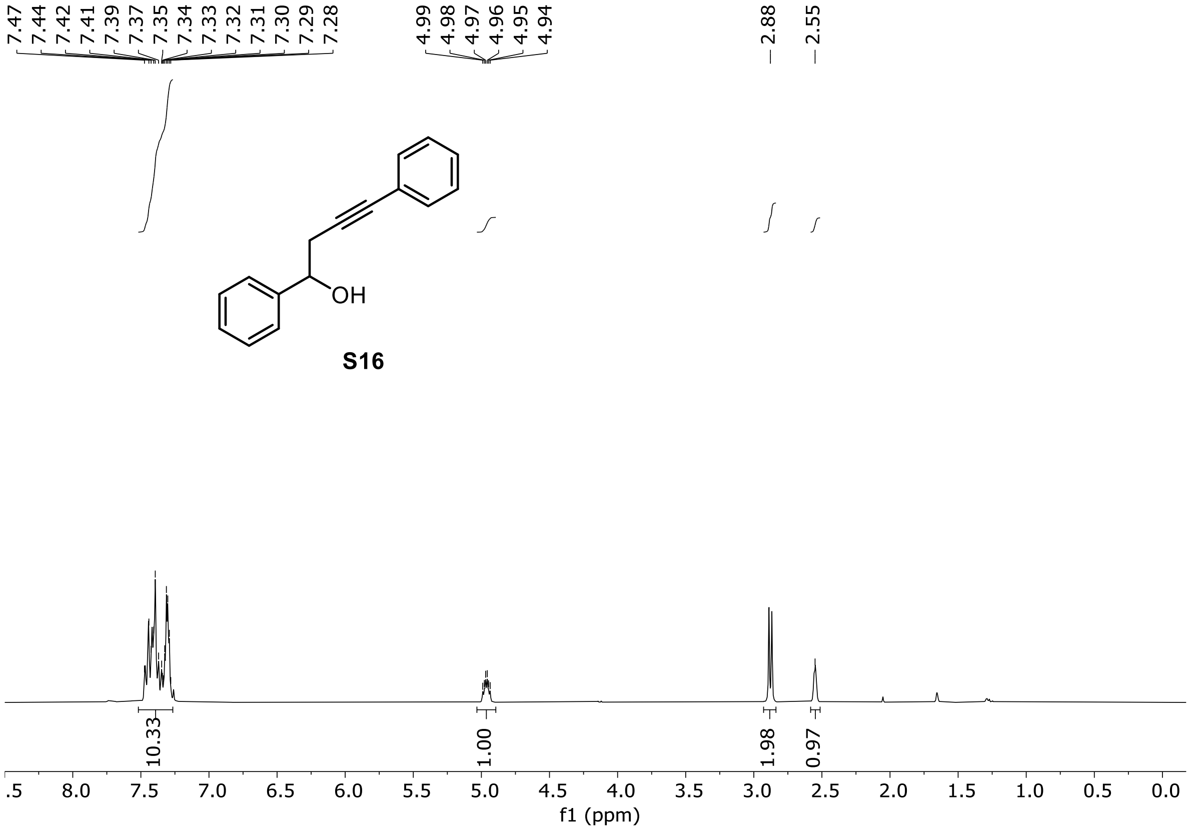

Figure S33. ^{1}H NMR spectrum of **S16** ($CDCl_3$, 300 MHz, 298 K)

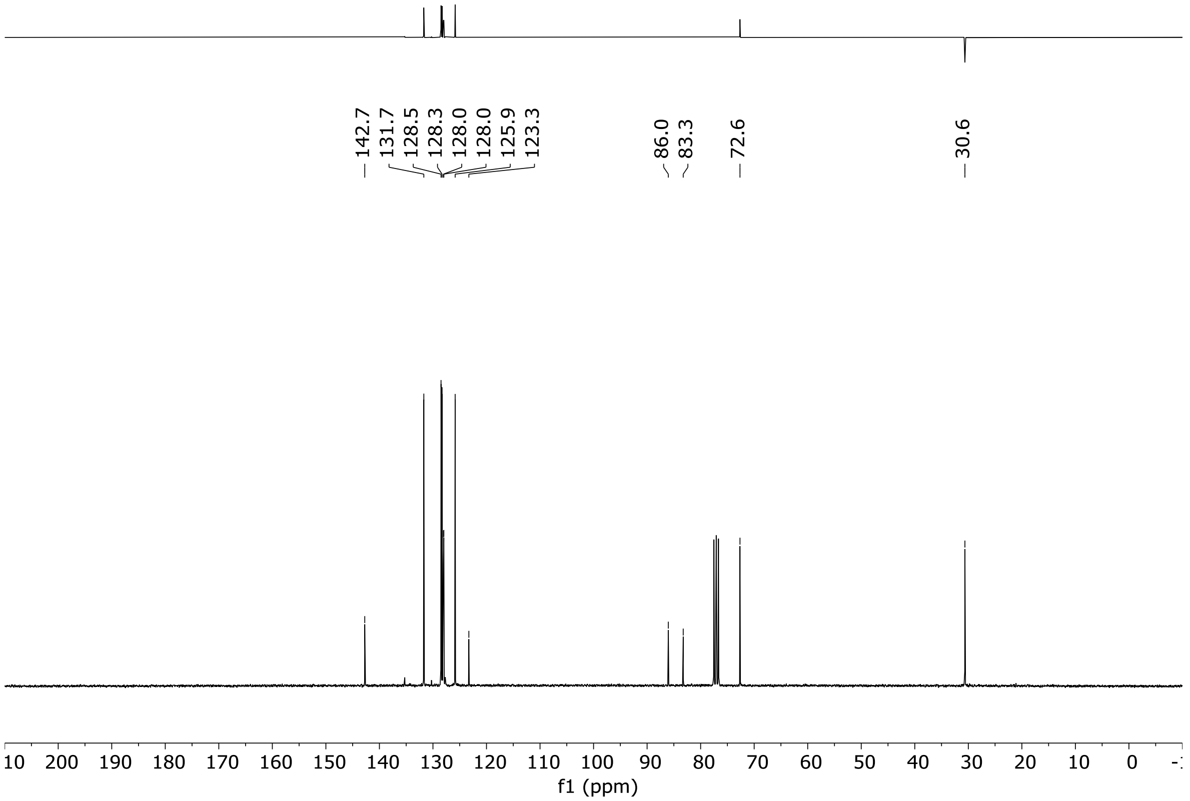

Figure S34. $^{13}C\{1H\}$ NMR spectrum of **S16** ($CDCl_3$, 75 MHz, 298 K)

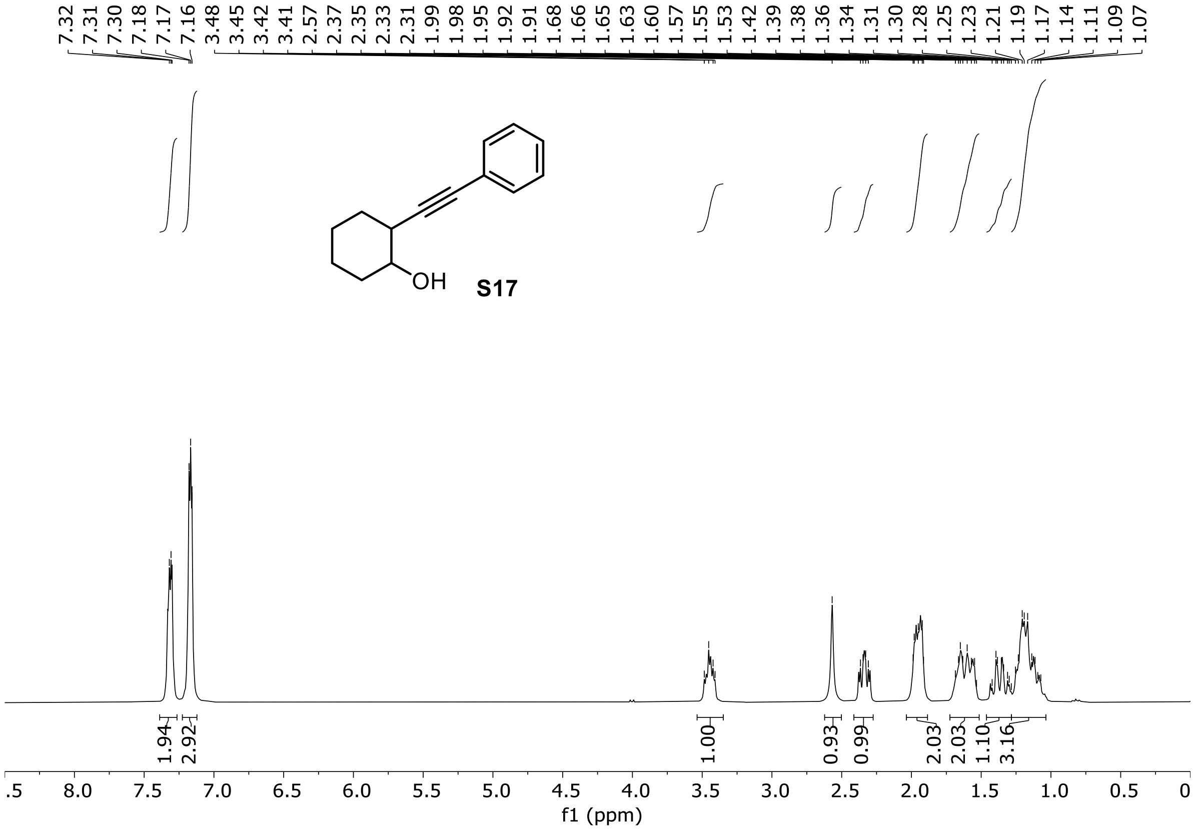

Figure S35. ^{1}H NMR spectrum of **S17** ($CDCl_3$, 300 MHz, 298 K)

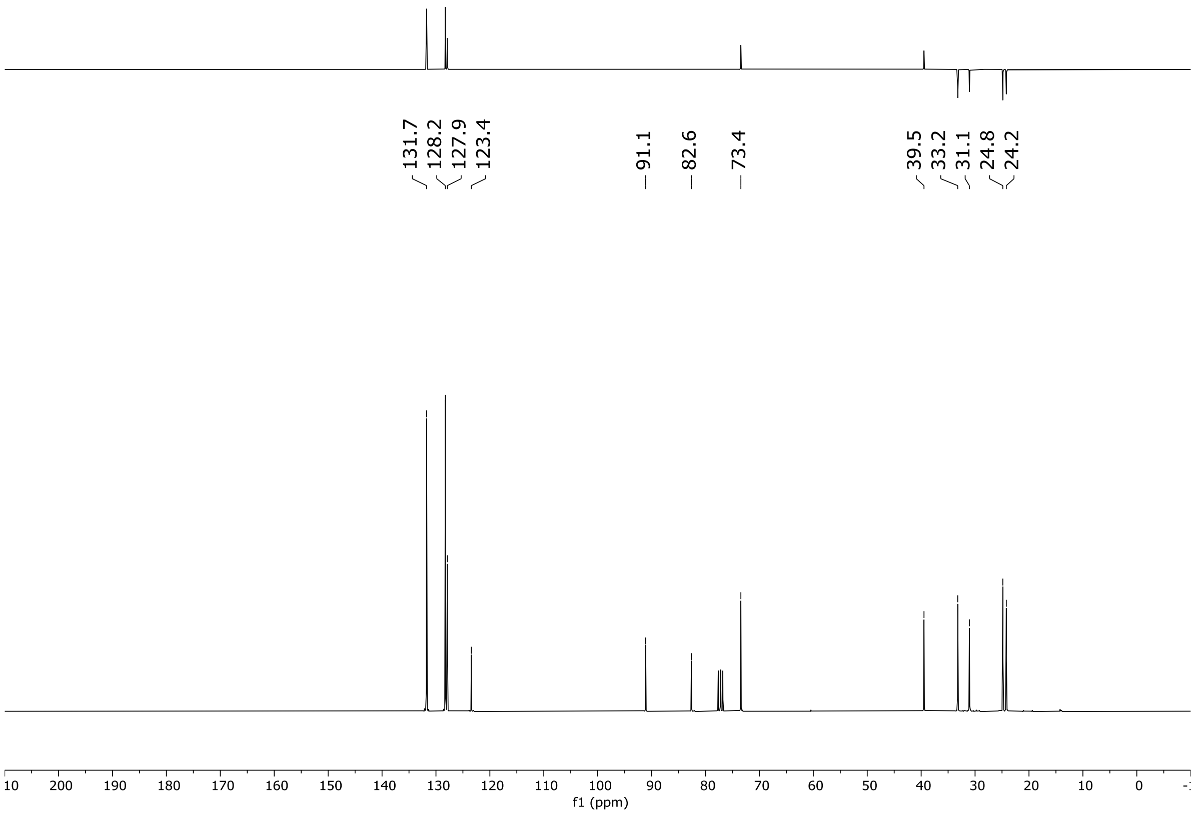

Figure S36. ^{13}C{1H} NMR spectrum of **S17** ($CDCl_3$, 75 MHz, 298 K)

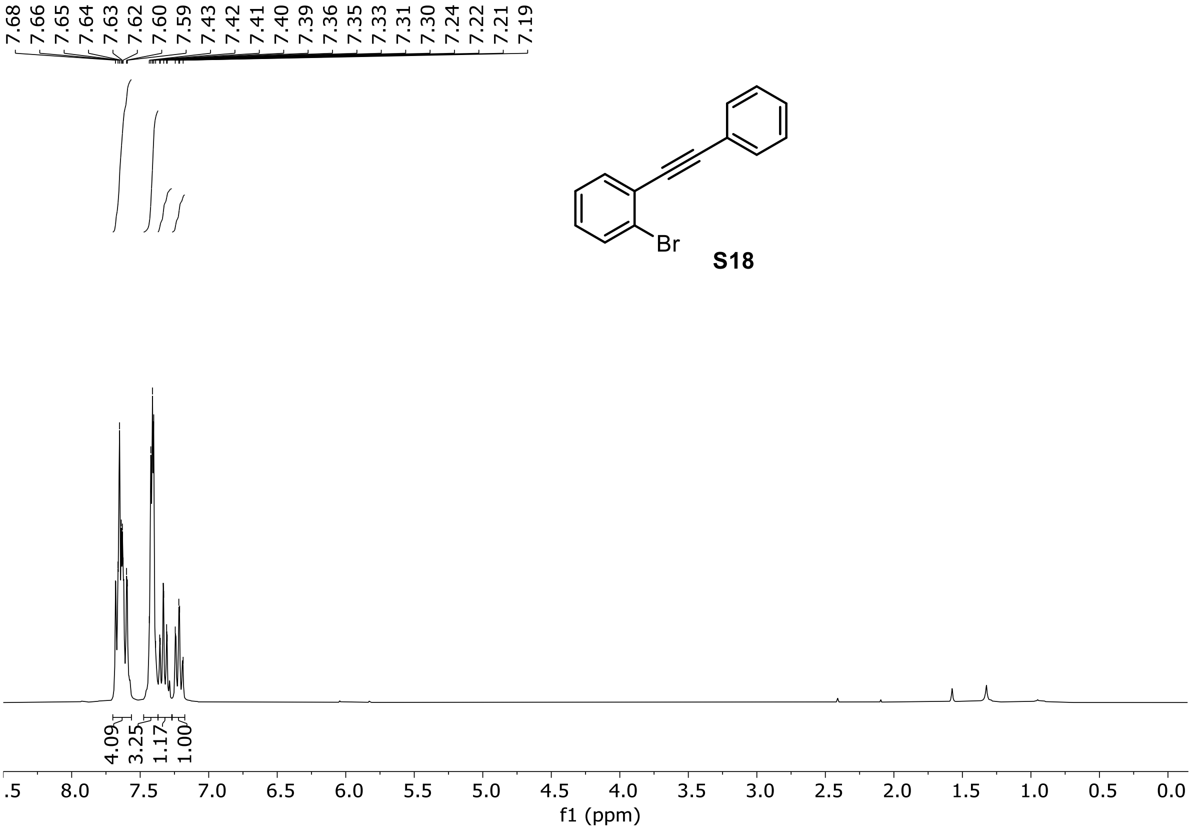

Figure S37. ^{1}H NMR spectrum of **S18** ($CDCl_3$, 300 MHz, 298 K)

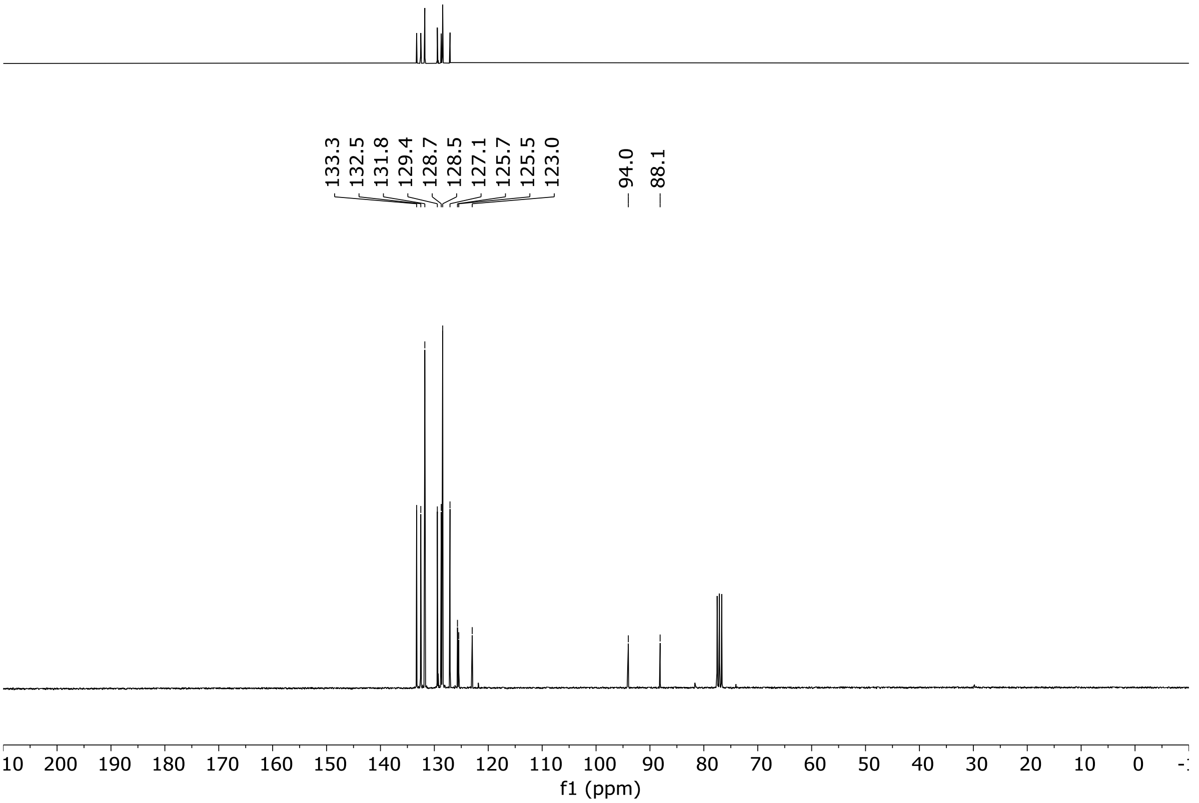

Figure S38. $^{13}C\{1H\}$ NMR spectrum of **S18** ($CDCl_3$, 75 MHz, 298 K)

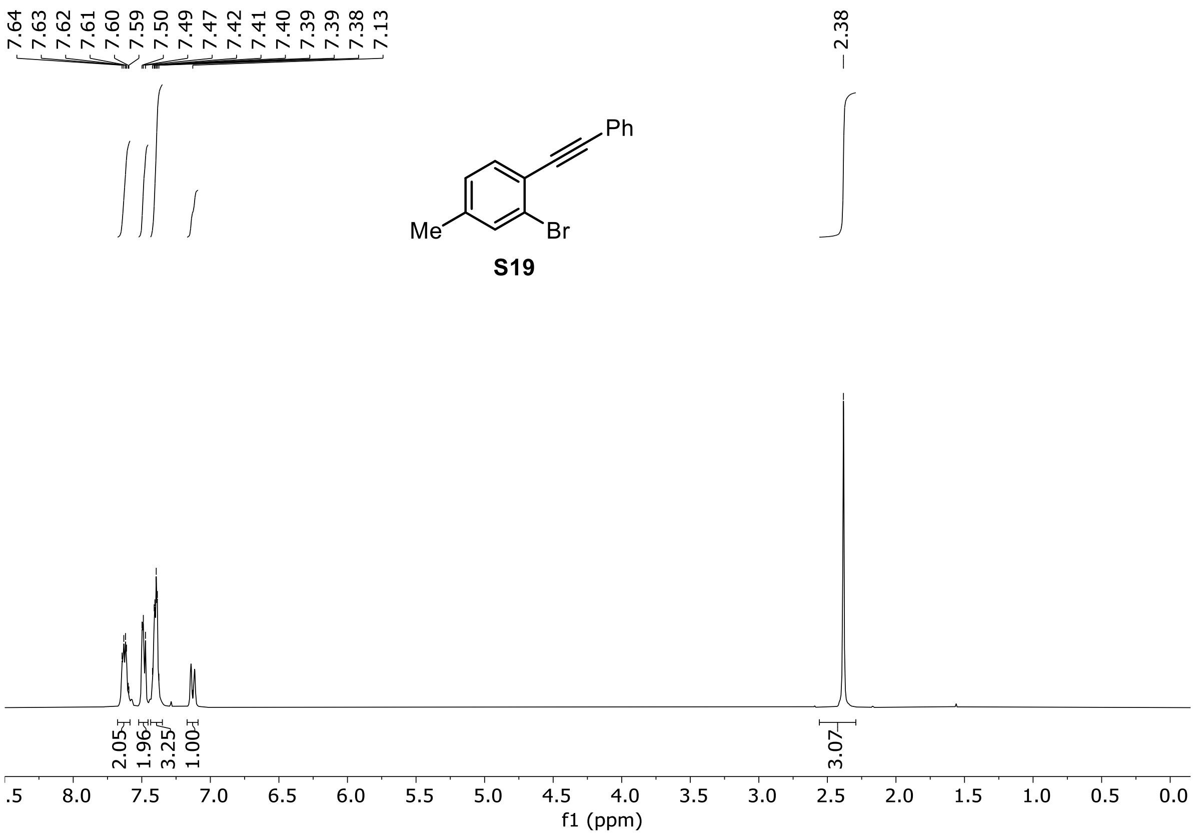

Figure S39. ^{1}H NMR spectrum of **S19** ($CDCl_3$, 300 MHz, 298 K)

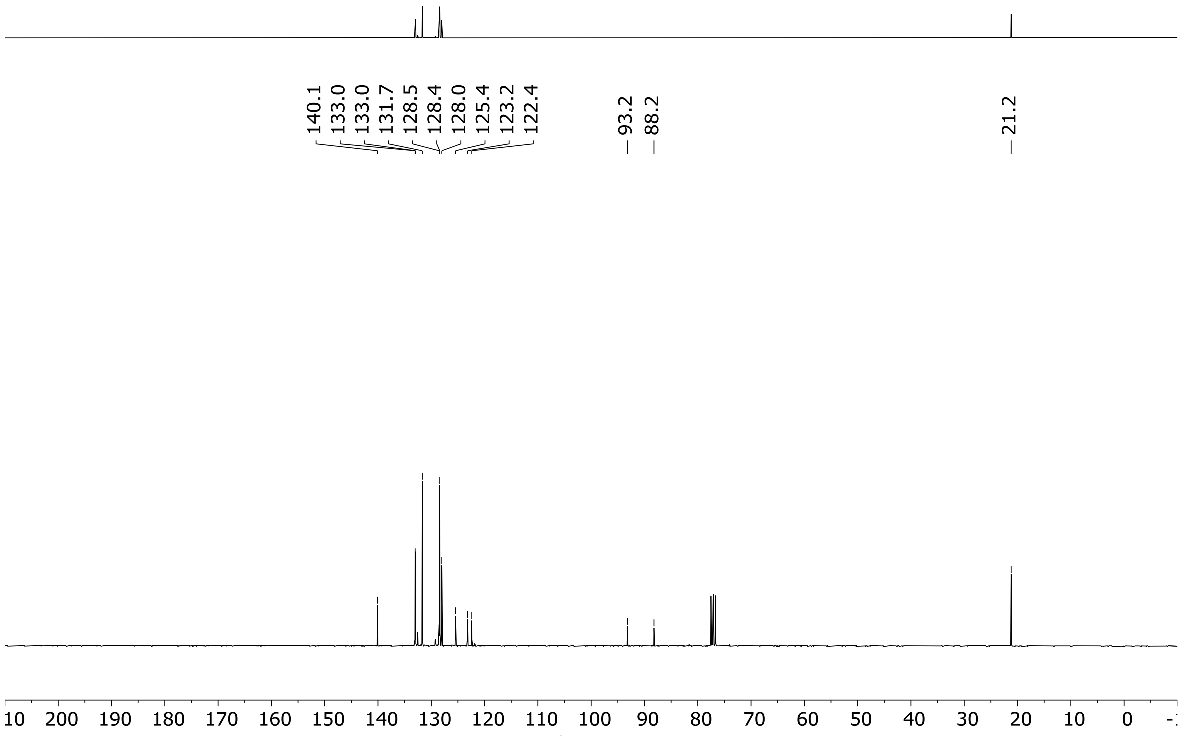

Figure S40. $^{13}C\{1H\}$ NMR spectrum of **S19** ($CDCl_3$, 75 MHz, 298 K)

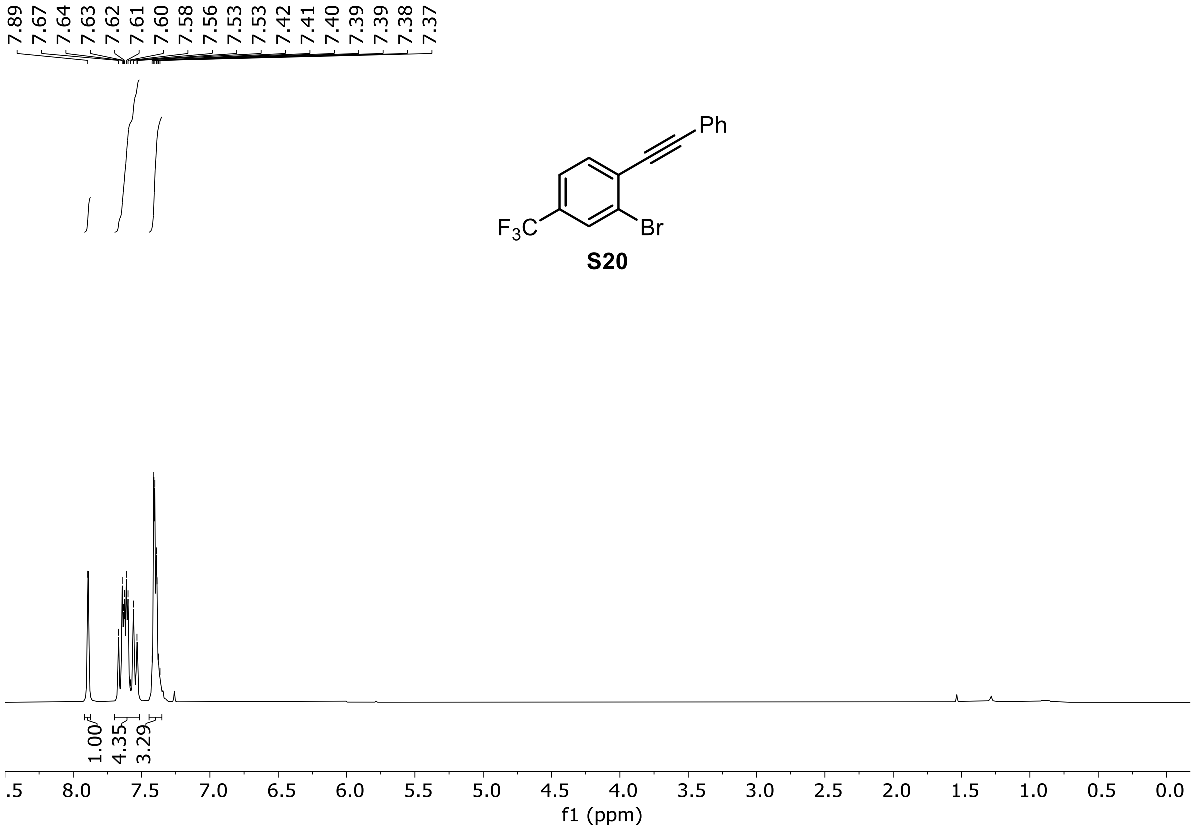

Figure S41. ^{1}H NMR spectrum of **S20** ($CDCl_3$, 300 MHz, 298 K)

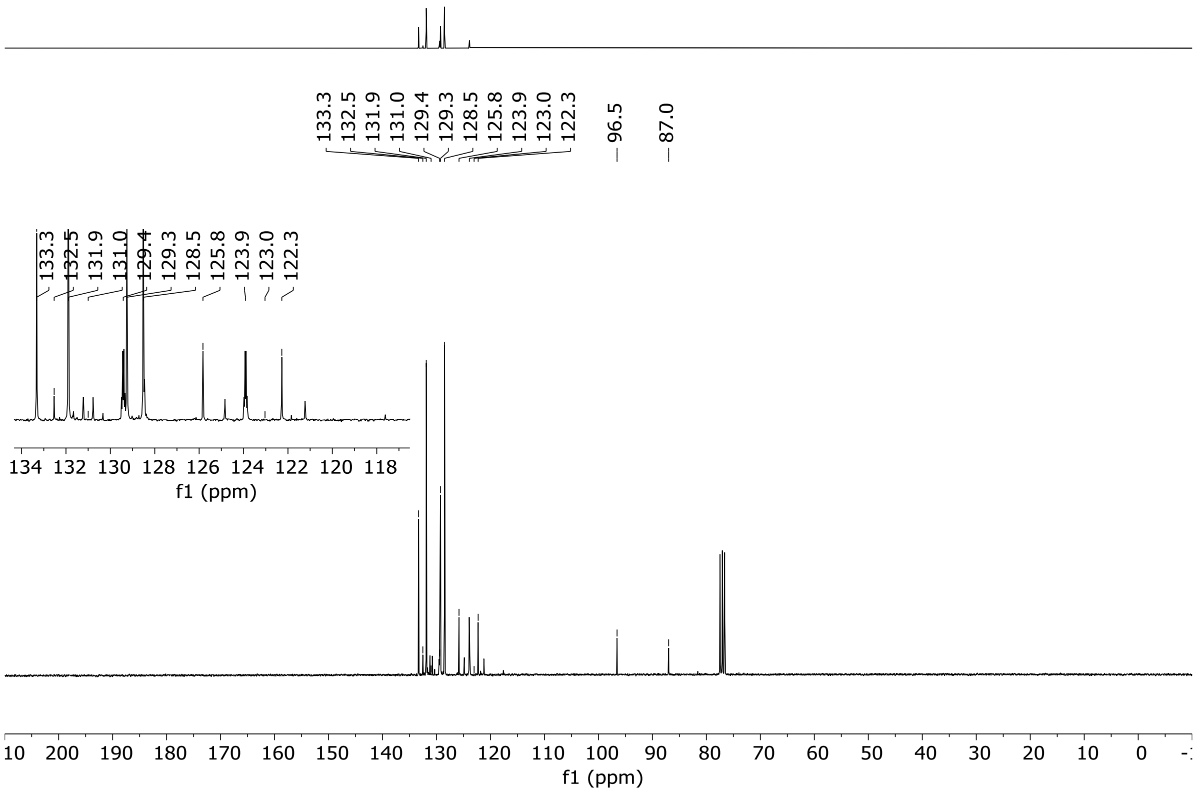

Figure S42. ^{13}C{1H} NMR spectrum of **S20** ($CDCl_3$, 75 MHz, 298 K)

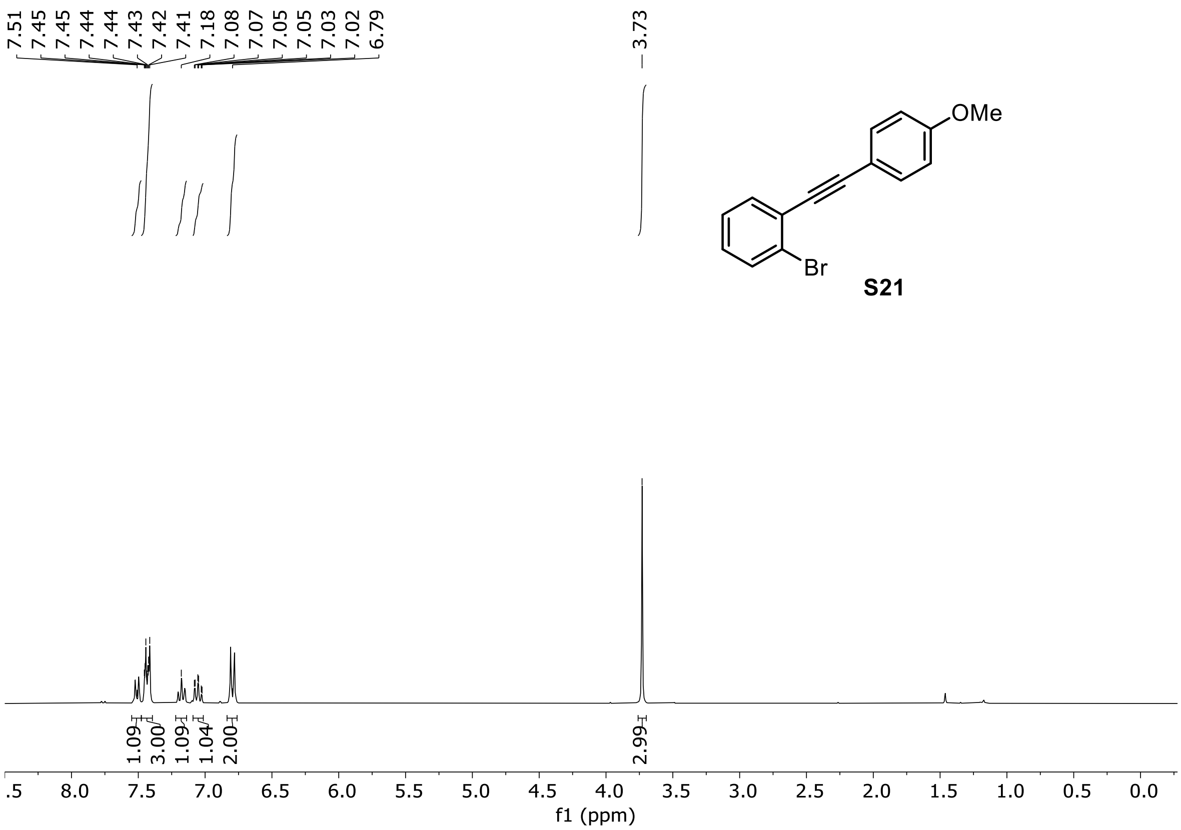

Figure S43. ^{1}H NMR spectrum of **S21** ($CDCl_3$, 300 MHz, 298 K)

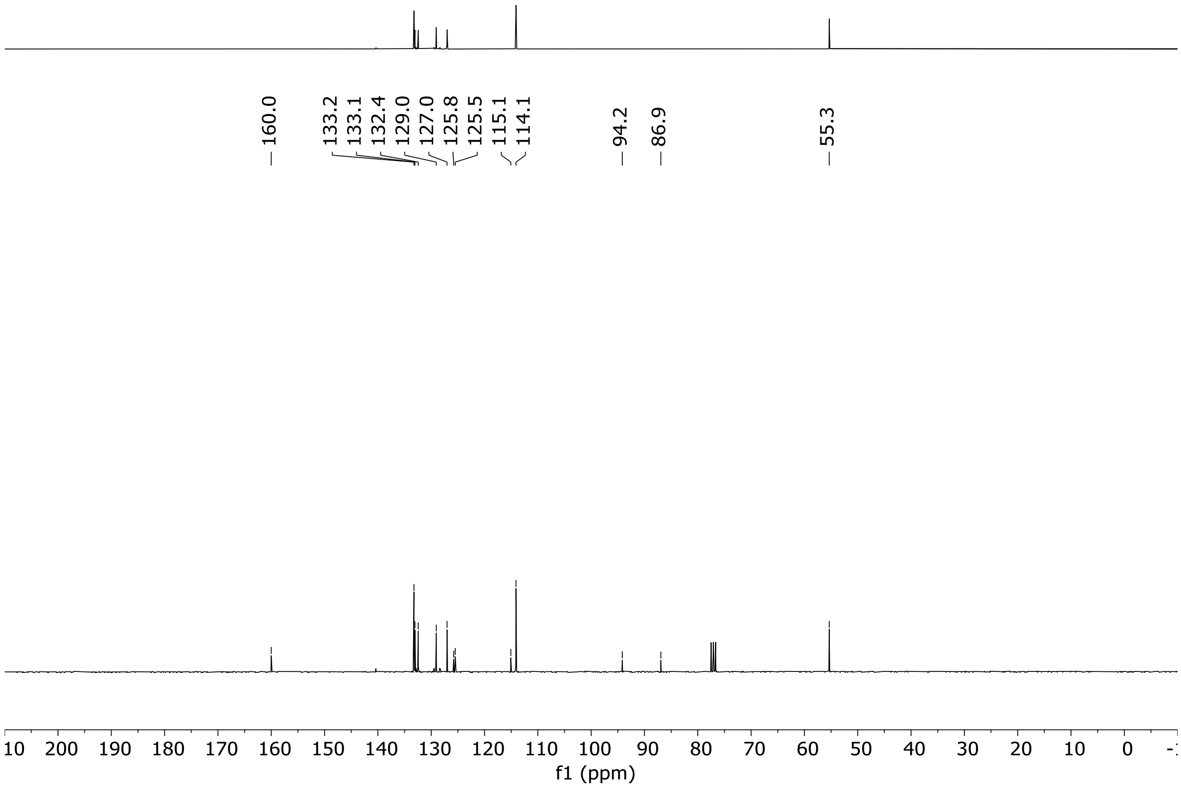

Figure S44. $^{13}C\{1H\}$ NMR spectrum of **S21** ($CDCl_3$, 75 MHz, 298 K)

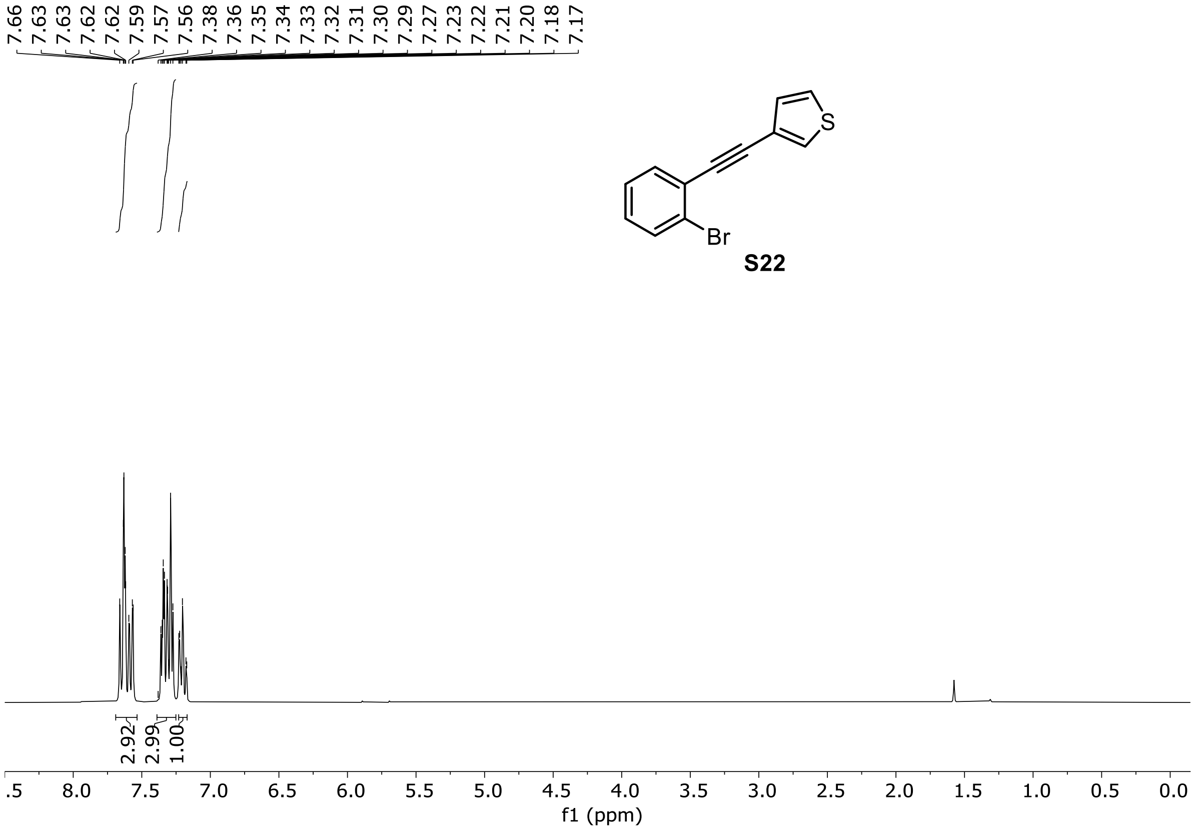

Figure S45. ^{1}H NMR spectrum of **S22** ($CDCl_3$, 300 MHz, 298 K)

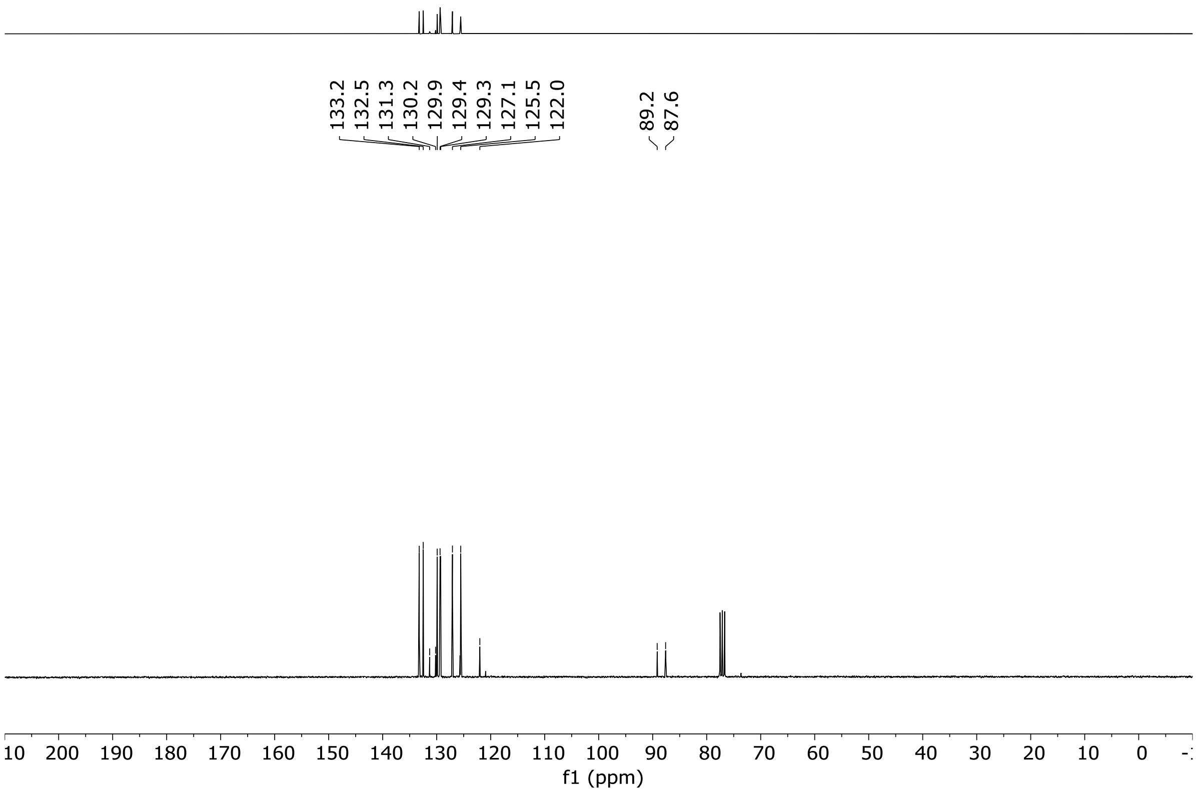

Figure S46. ^{13}C{1H} NMR spectrum of **S22** ($CDCl_3$, 75 MHz, 298 K)

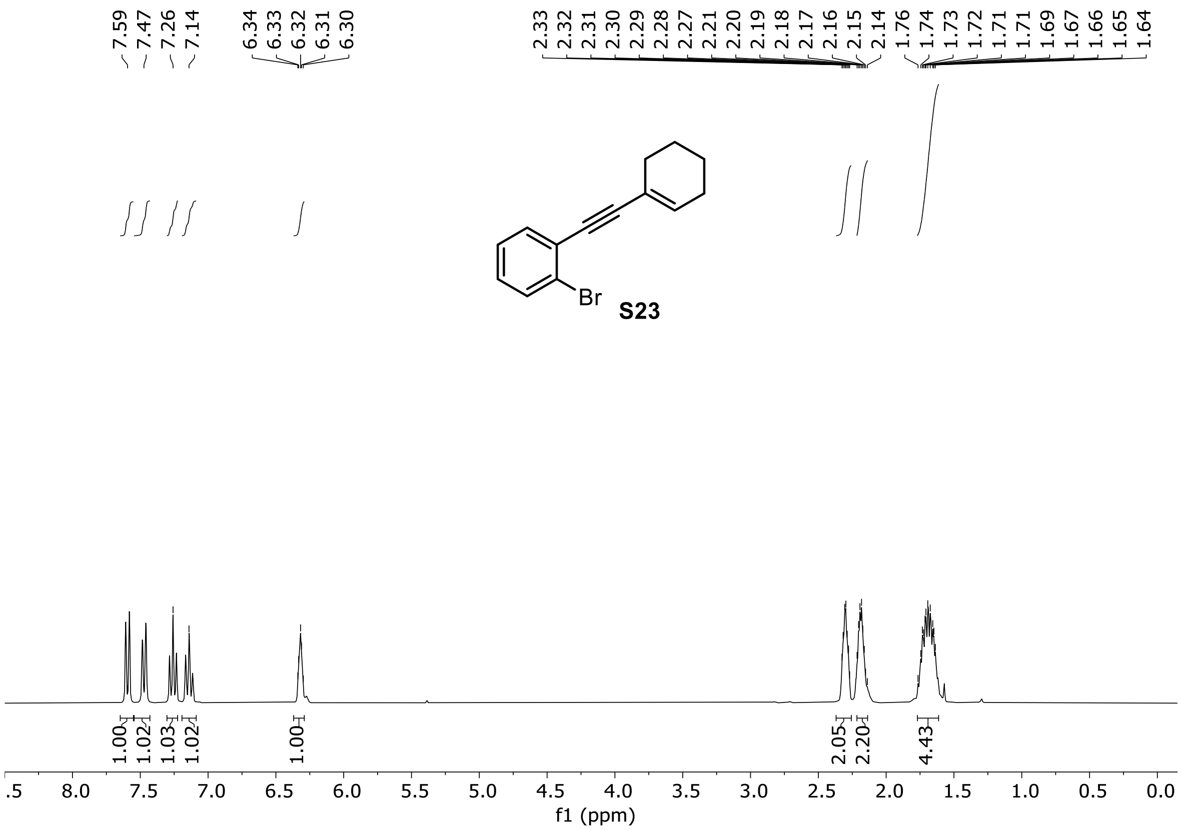

Figure S47. ^{1}H NMR spectrum of **S23** ($CDCl_3$, 300 MHz, 298 K)

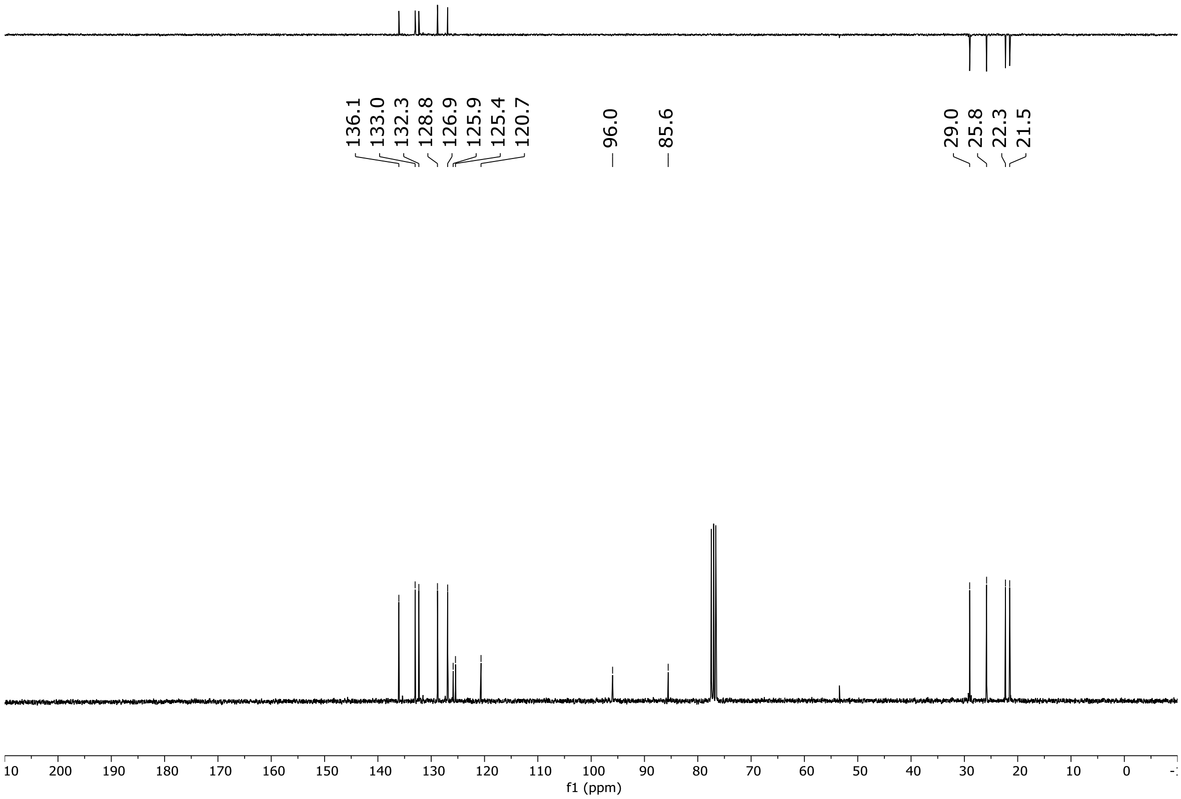

Figure S48. $^{13}C\{1H\}$ NMR spectrum of **S23** ($CDCl_3$, 75 MHz, 298 K)

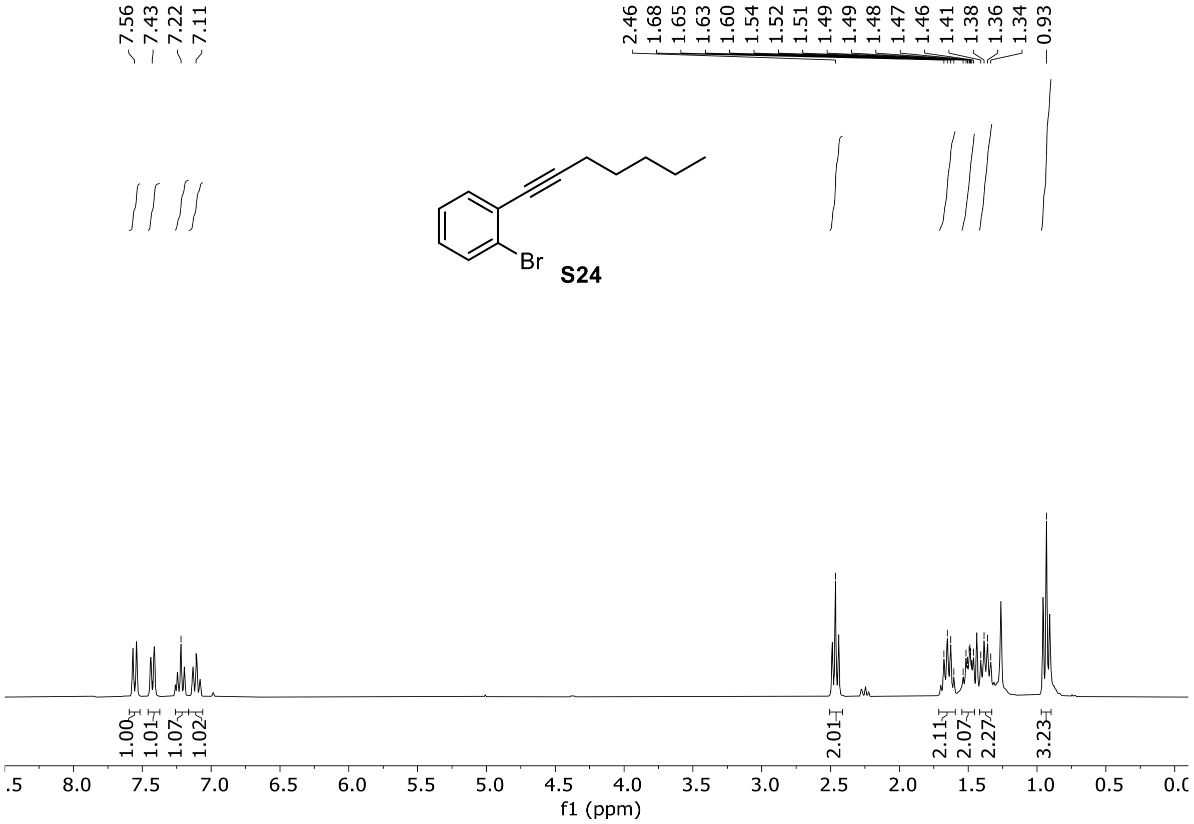

Figure S49. ^{1}H NMR spectrum of **S24** ($CDCl_3$, 300 MHz, 298 K)

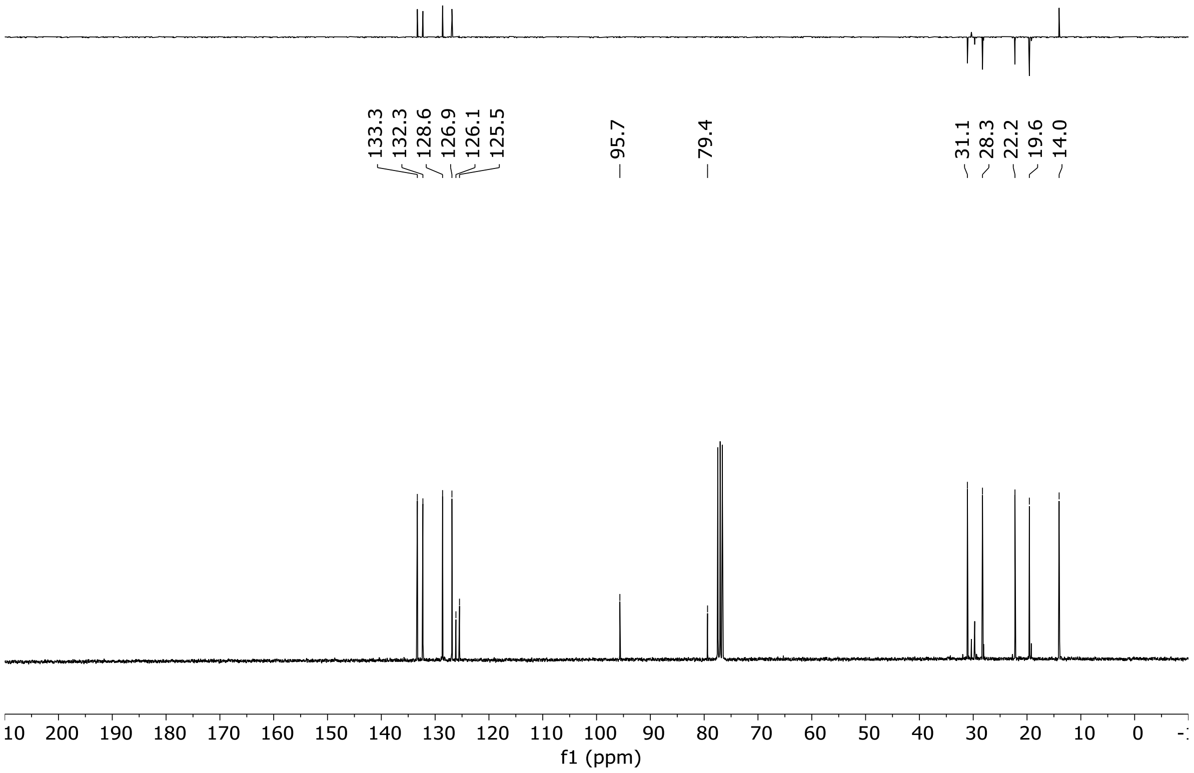

Figure S50. $^{13}C\{1H\}$ NMR spectrum of **S23** ($CDCl_3$, 75 MHz, 298 K)

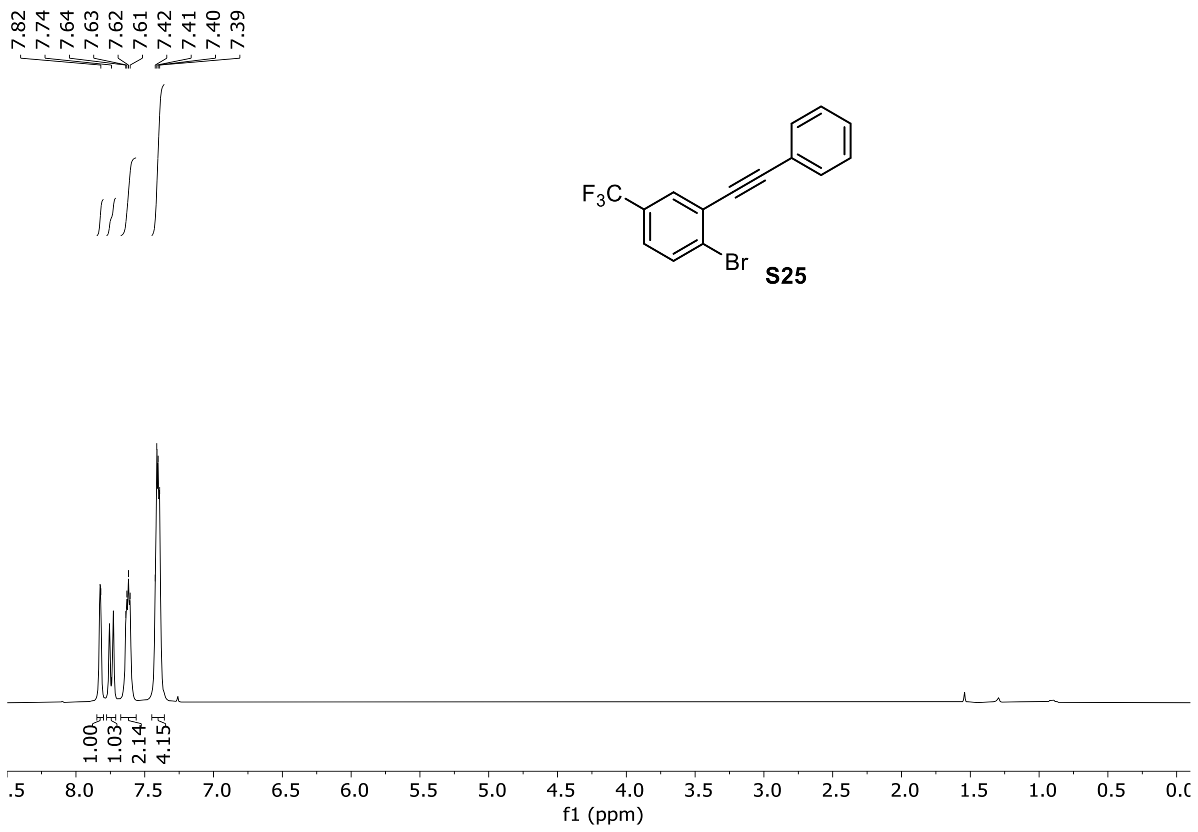

Figure S51. ^{1}H NMR spectrum of **S25** ($CDCl_3$, 300 MHz, 298 K)

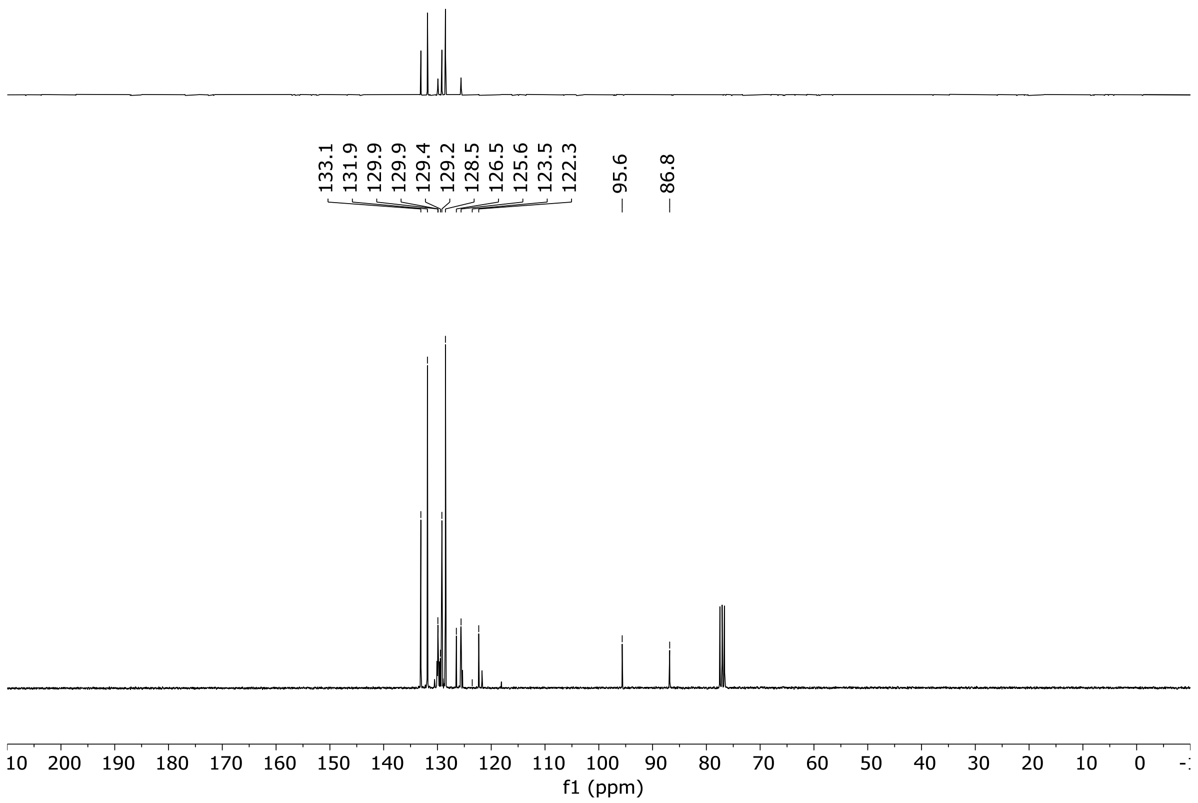

Figure S52. ^{13}C{1H} NMR spectrum of **S25** ($CDCl_3$, 75 MHz, 298 K)

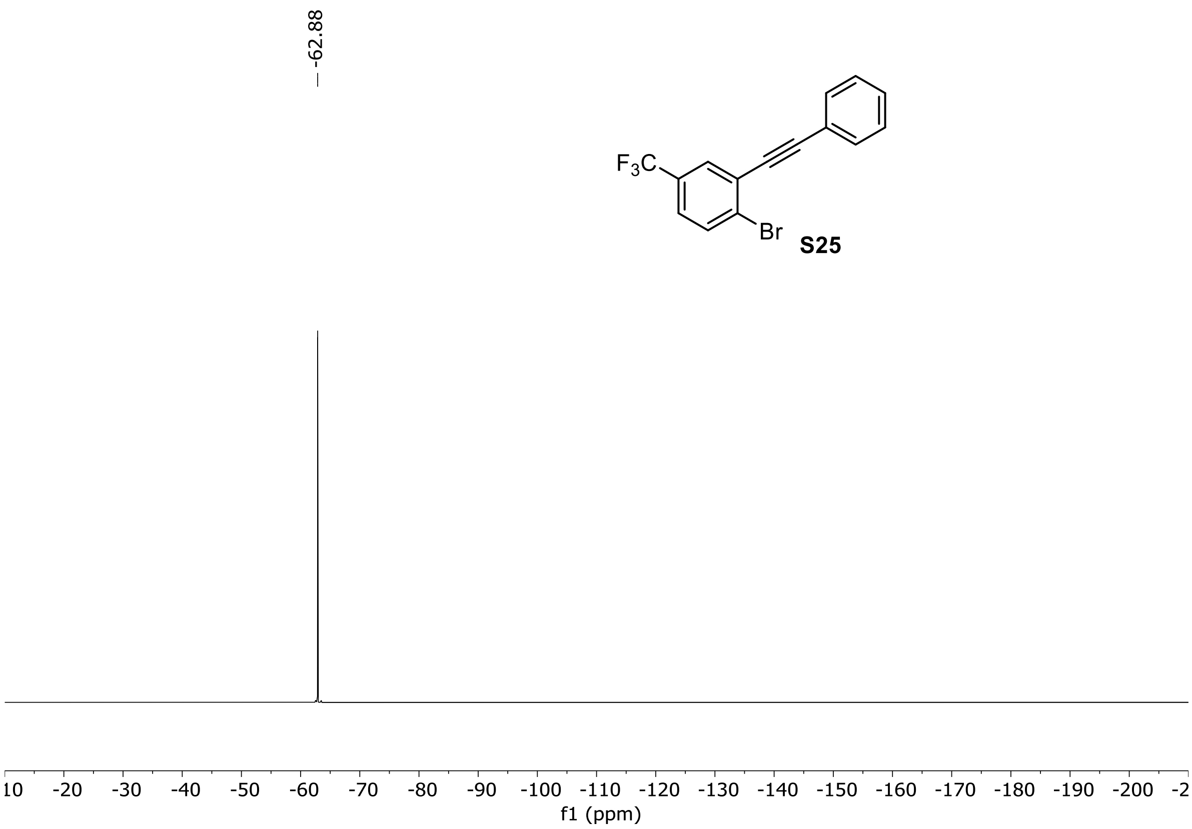

Figure S53. ^{19}F NMR spectrum of **S25** ($CDCl_3$, 282 MHz, 298 K)

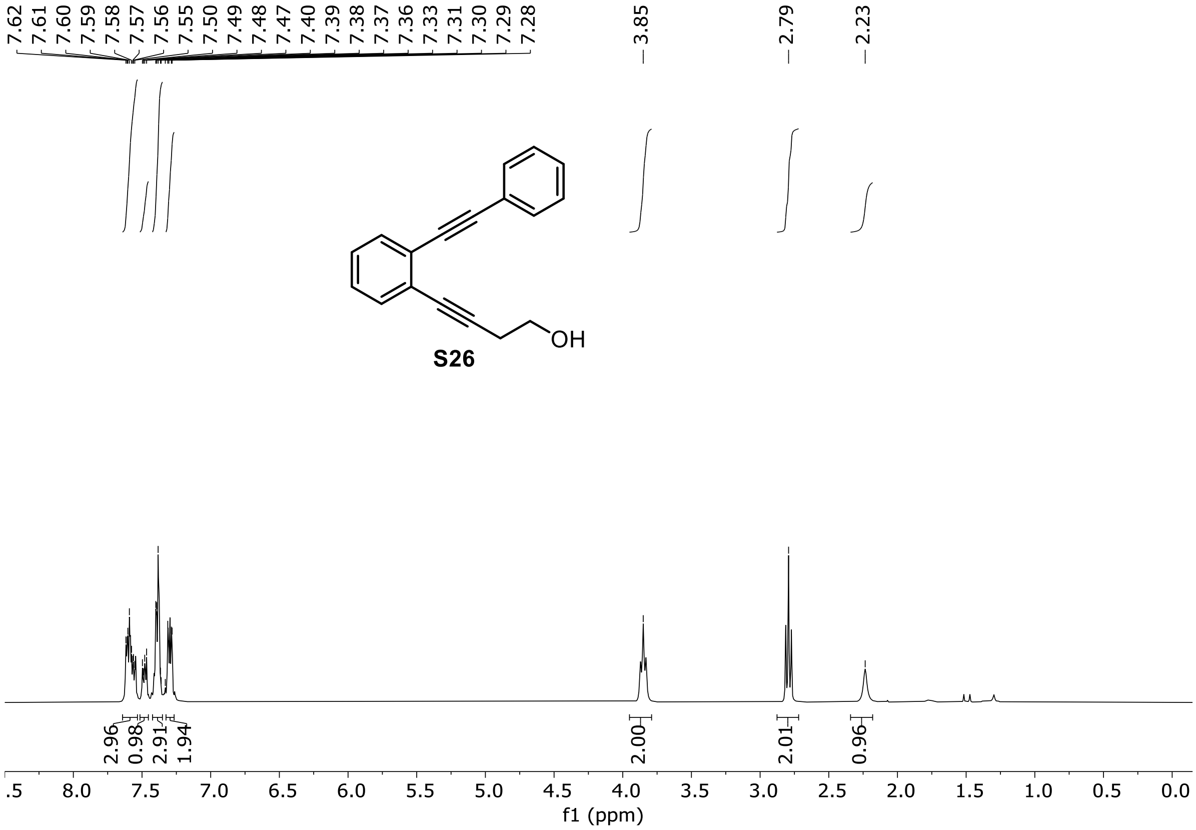

Figure S54. ^{1}H NMR spectrum of **S26** ($CDCl_3$, 300 MHz, 298 K)

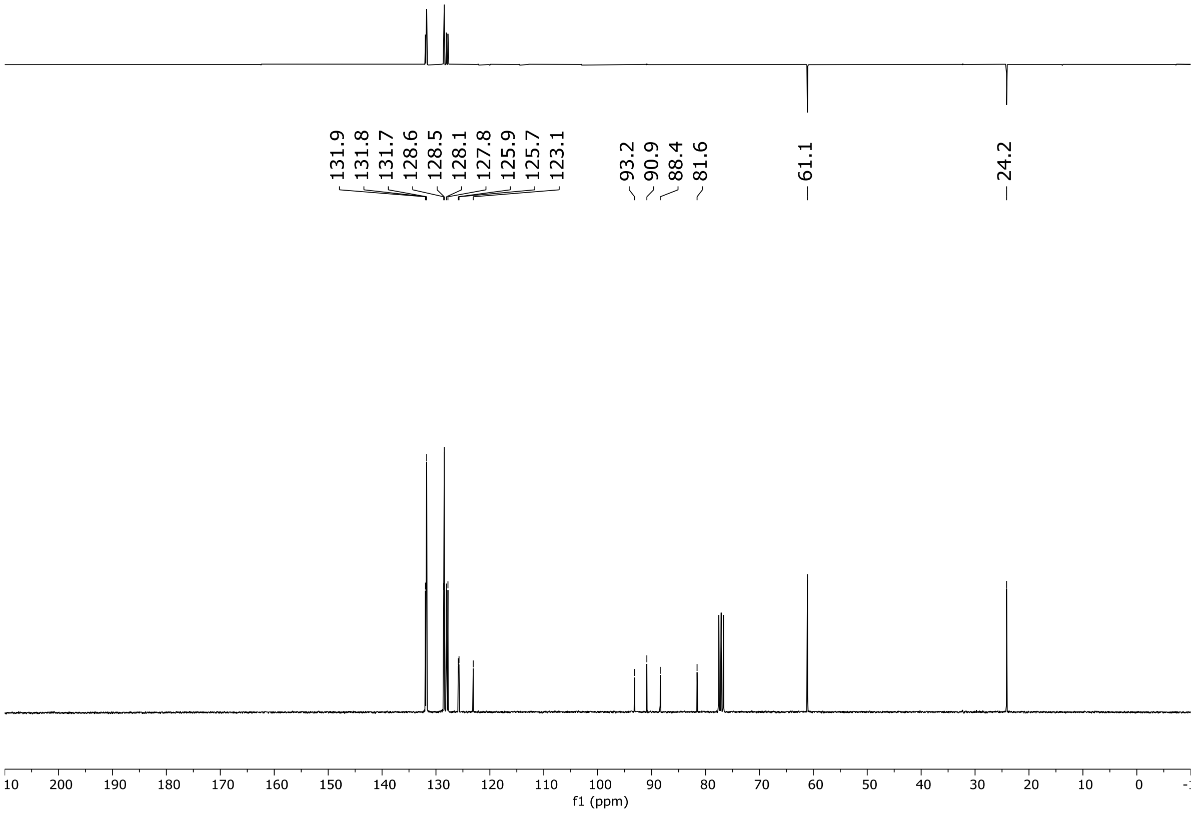

Figure S55. ^{13}C{1H} NMR spectrum of **S26** ($CDCl_3$, 75 MHz, 298 K)

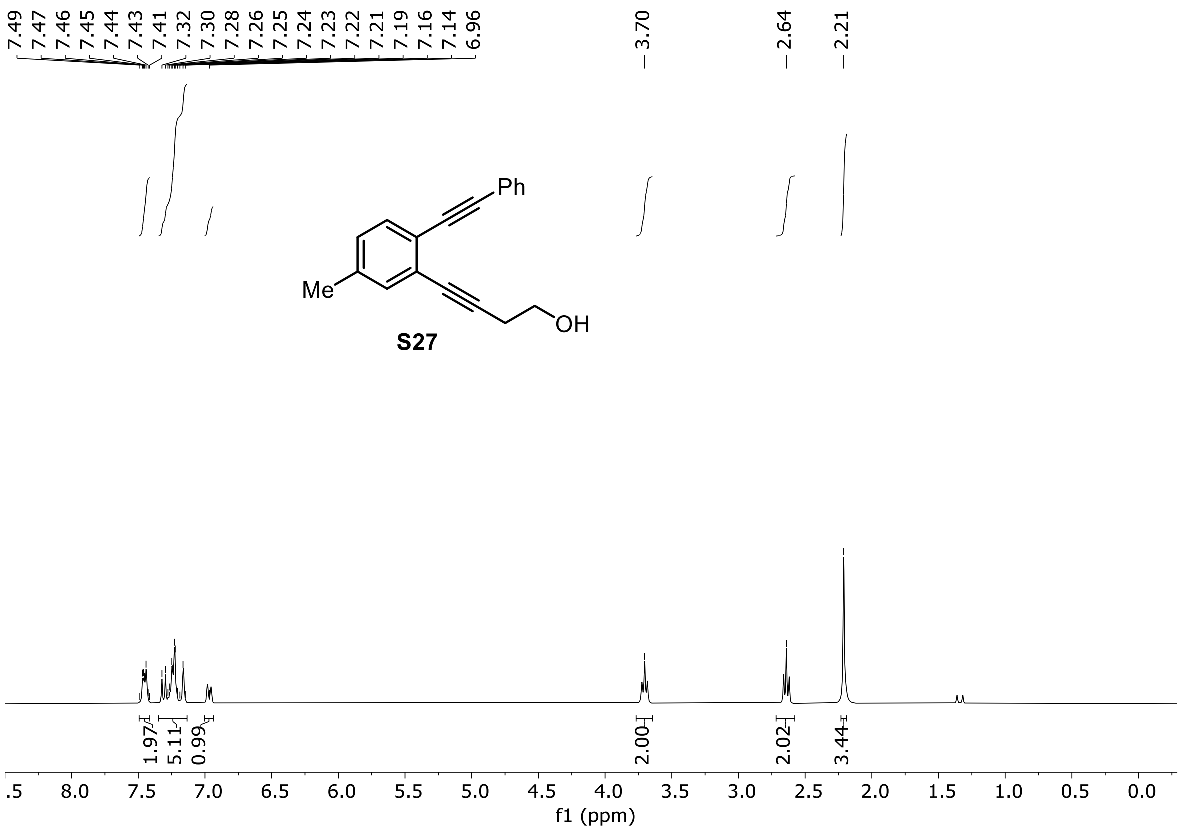

Figure S56. ^{1}H NMR spectrum of **S27** ($CDCl_3$, 300 MHz, 298 K)

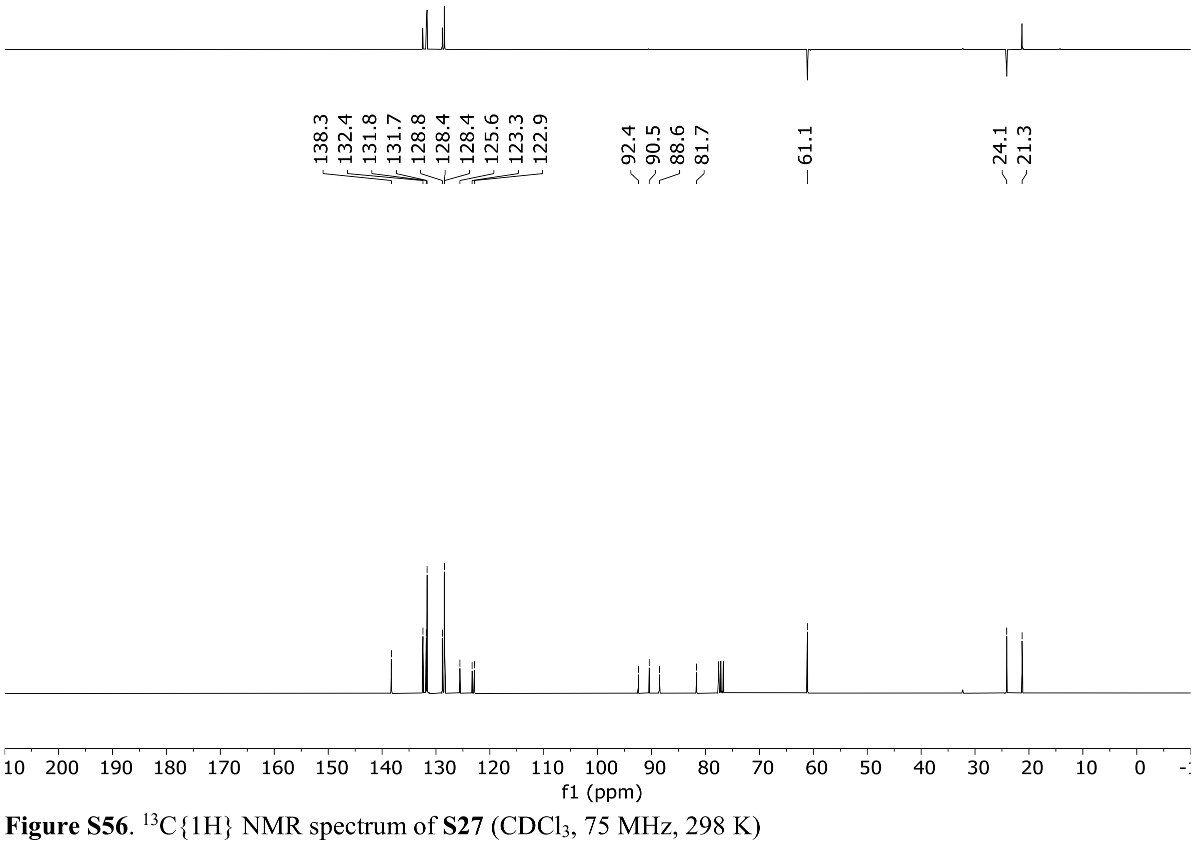

Figure S56. ^{13}C{1H} NMR spectrum of **S27** ($CDCl_3$, 75 MHz, 298 K)

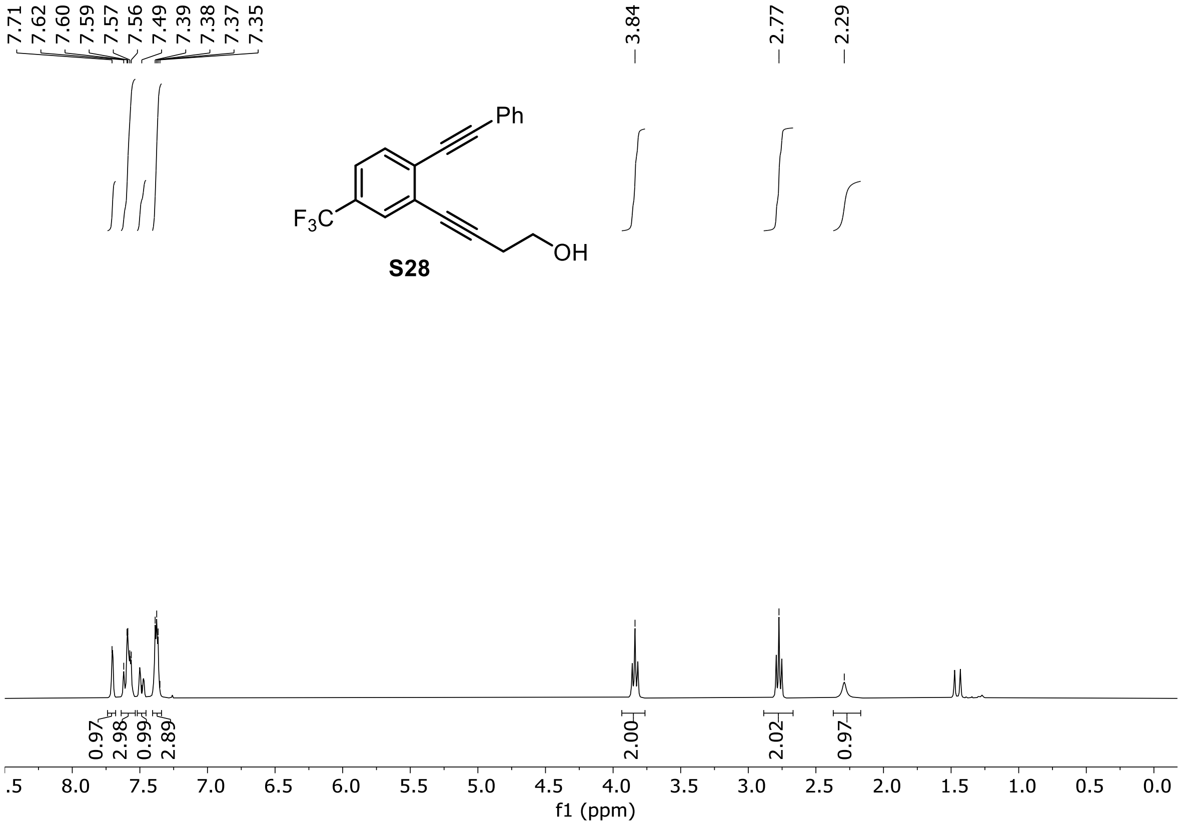

Figure S57. ^{1}H NMR spectrum of **S28** ($CDCl_3$, 300 MHz, 298 K)

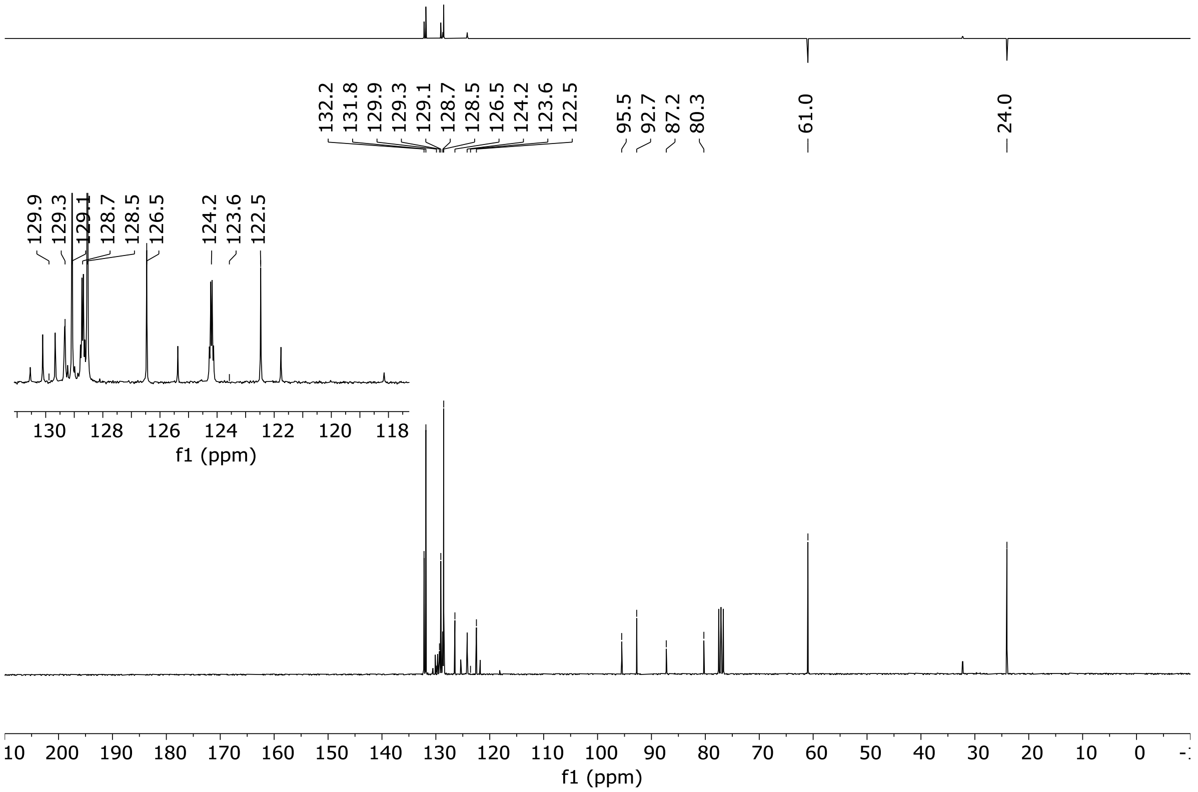

Figure S57. $^{13}C\{1H\}$ NMR spectrum of **S28** ($CDCl_3$, 75 MHz, 298 K)

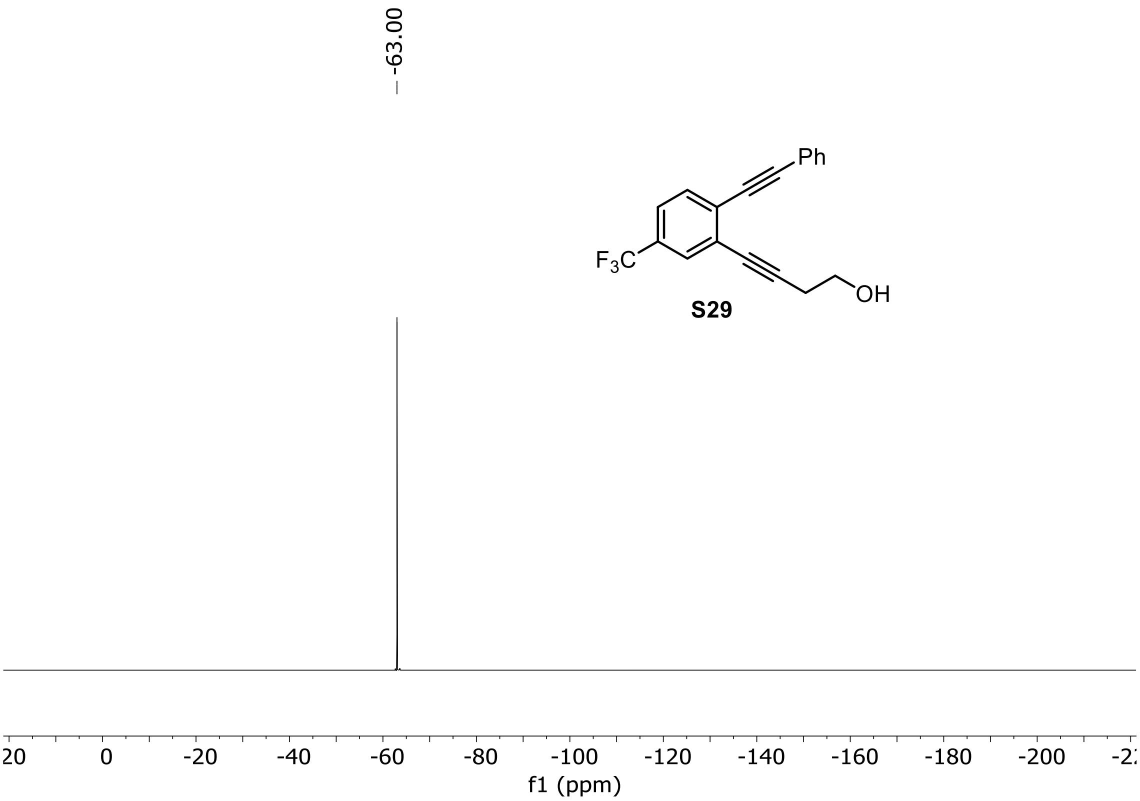

Figure S58. ^{19}F NMR spectrum of **S29** ($CDCl_3$, 285 MHz, 298 K)

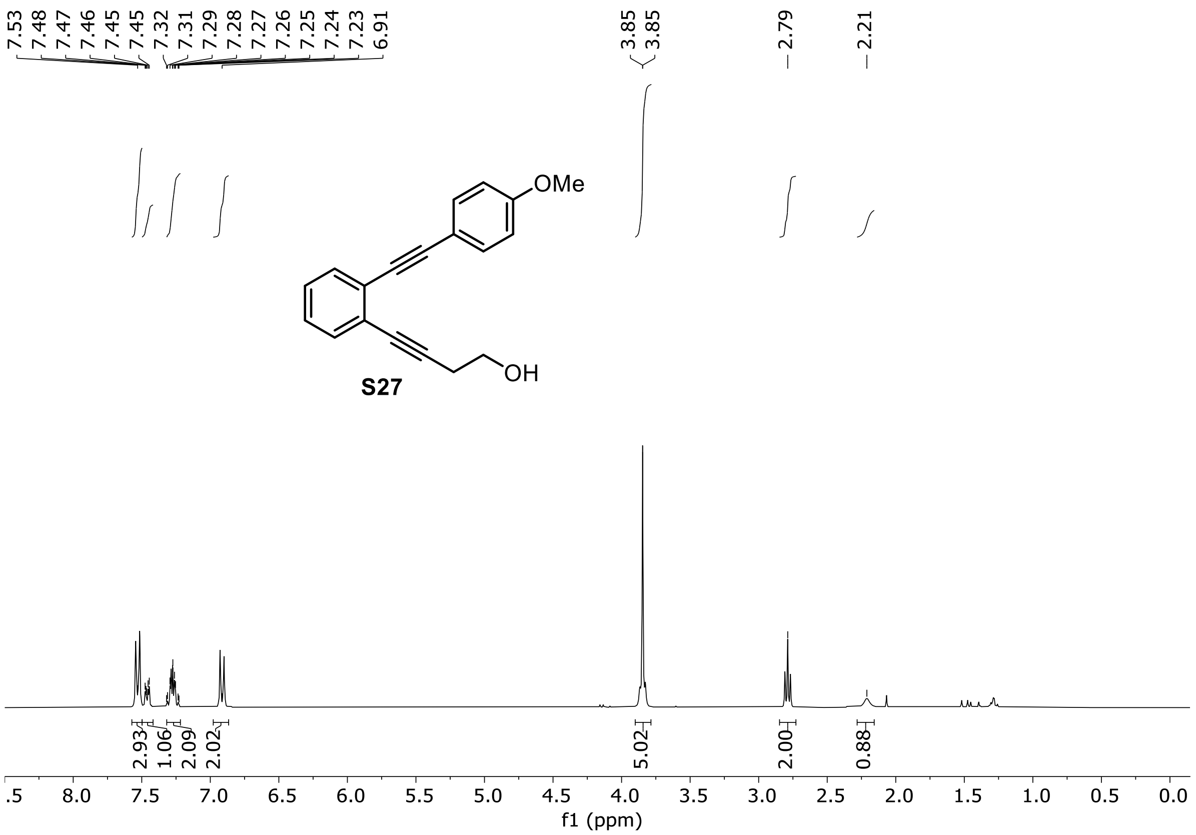

Figure S59. ^{1}H NMR spectrum of **S29** ($CDCl_3$, 300 MHz, 298 K)

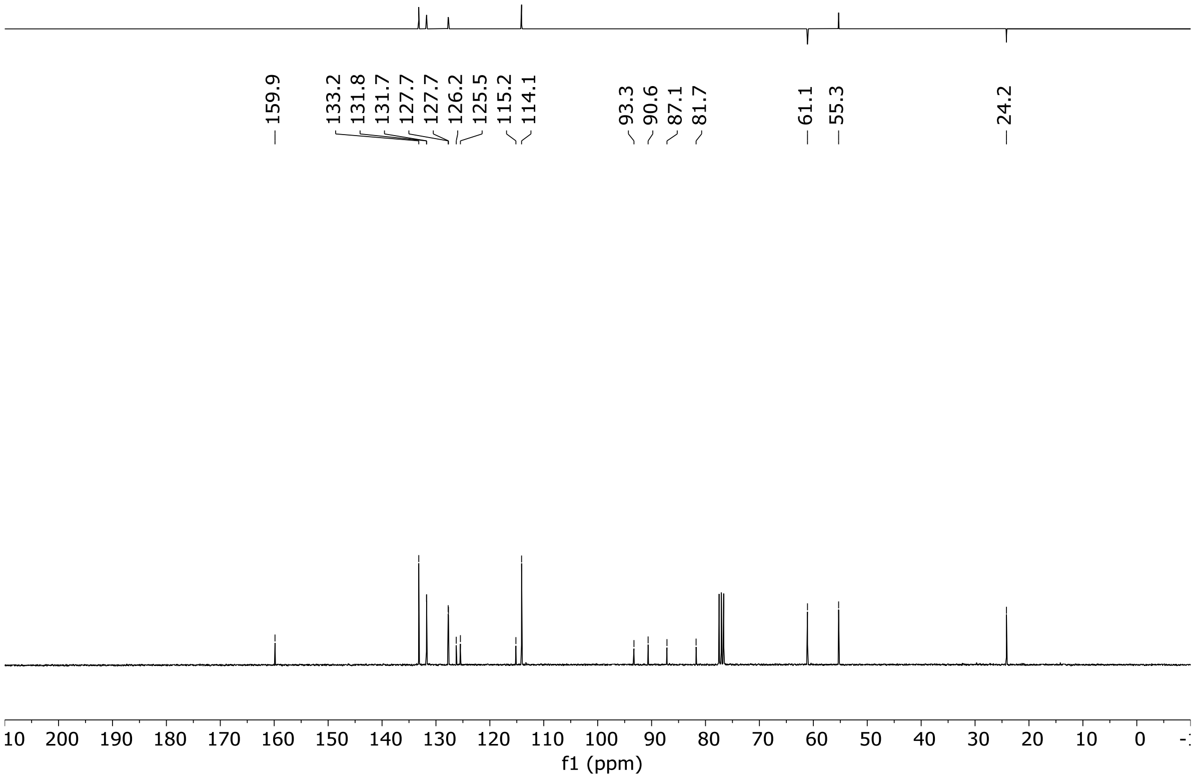

Figure S60. $^{13}C\{1H\}$ NMR spectrum of **S29** ($CDCl_3$, 75 MHz, 298 K)

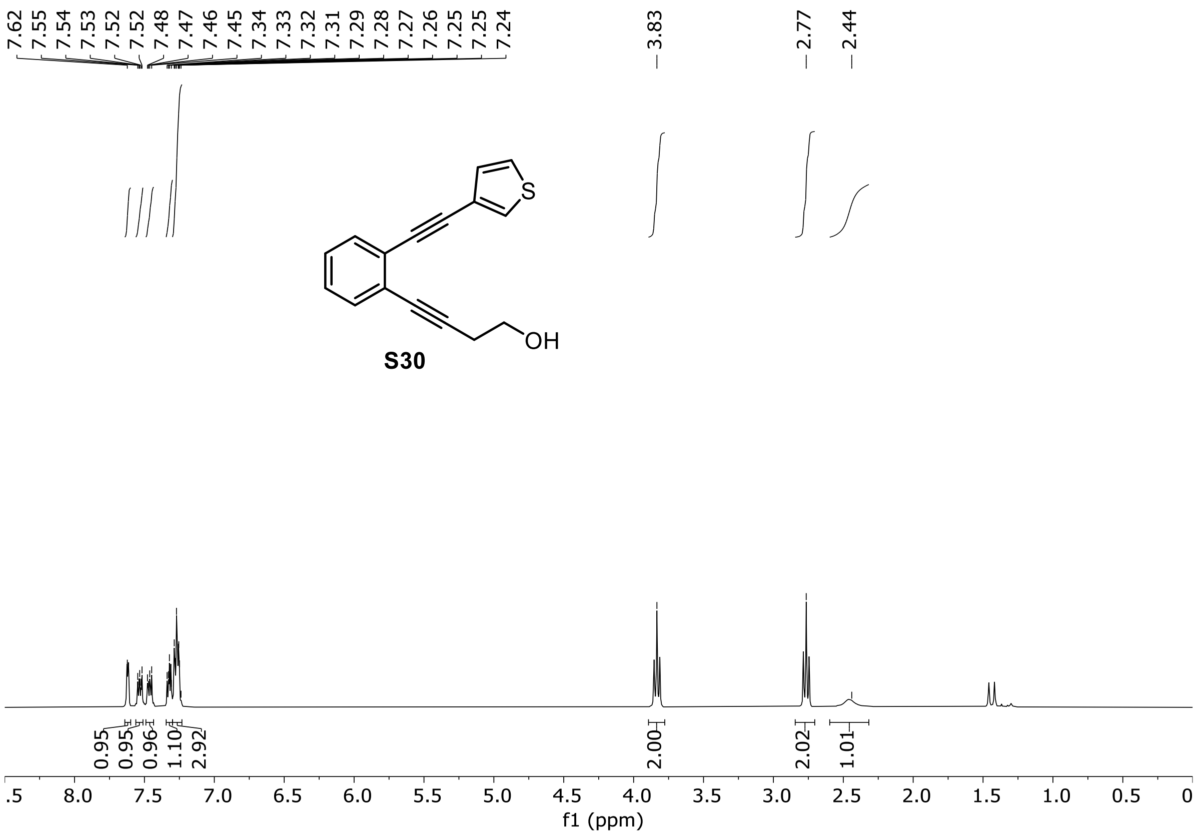

Figure S61. ^{1}H NMR spectrum of **S30** ($CDCl_3$, 300 MHz, 298 K)

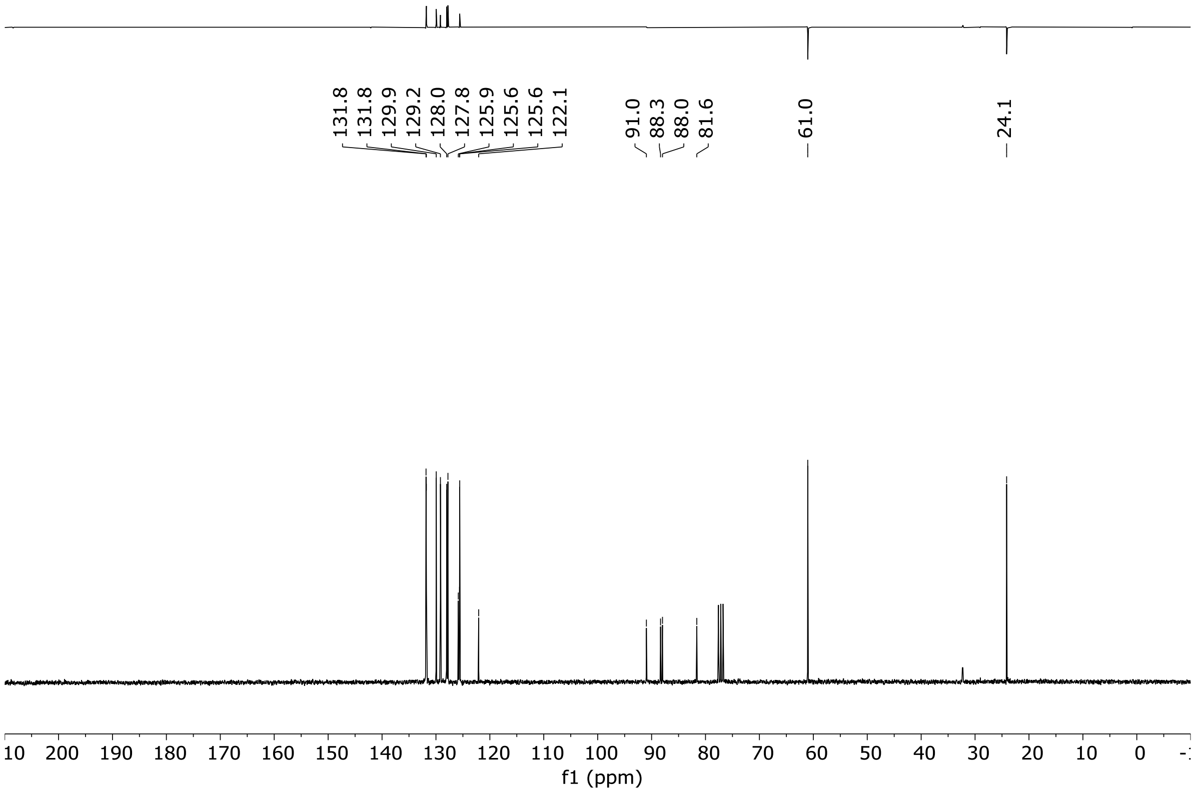

Figure S62. $^{13}C\{1H\}$ NMR spectrum of **S30** ($CDCl_3$, 75 MHz, 298 K)

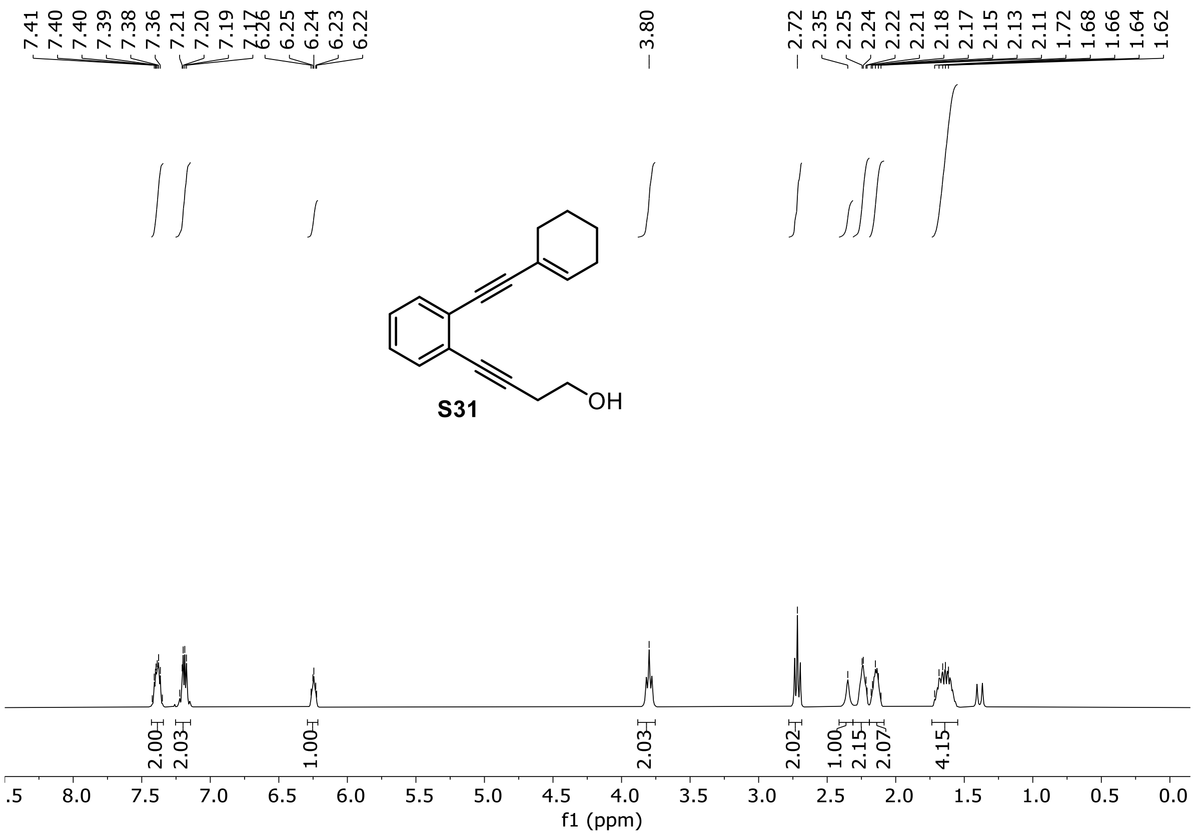

Figure S63. ^{1}H NMR spectrum of **S31** ($CDCl_3$, 300 MHz, 298 K)

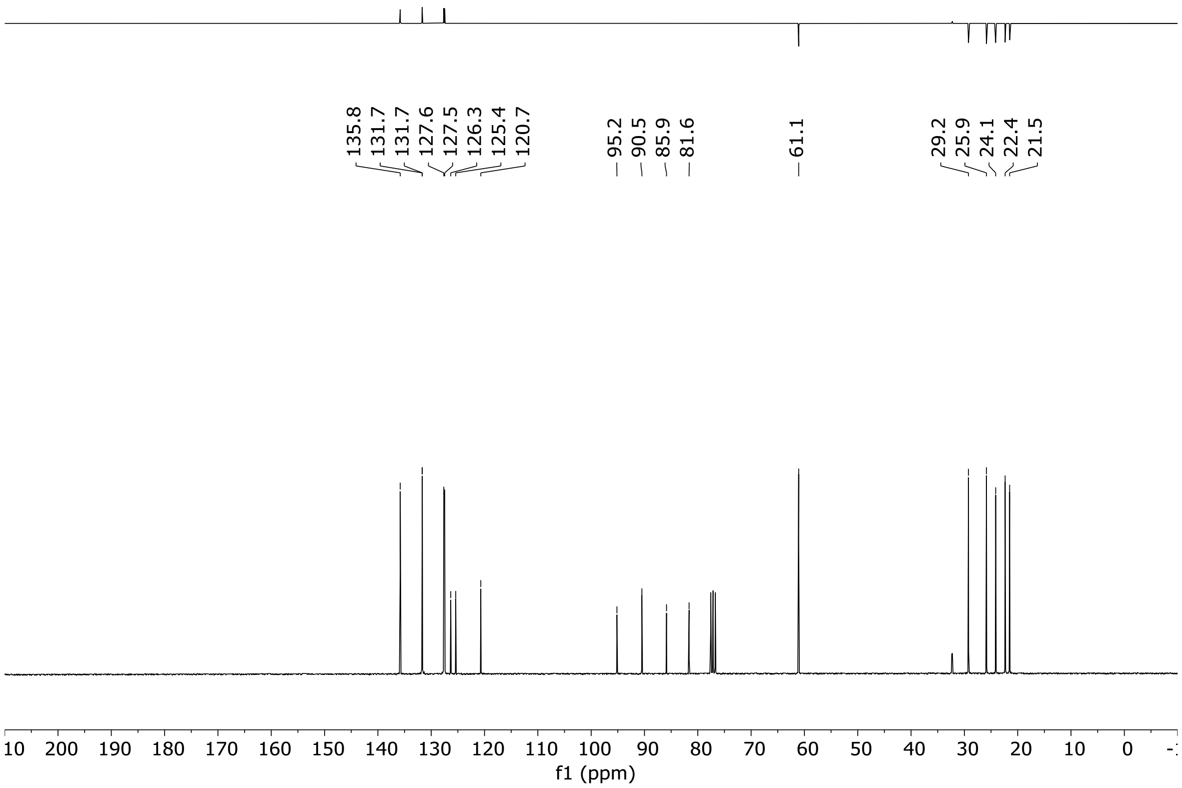

Figure S64. ^{13}C{1H} NMR spectrum of **S31** ($CDCl_3$, 75 MHz, 298 K)

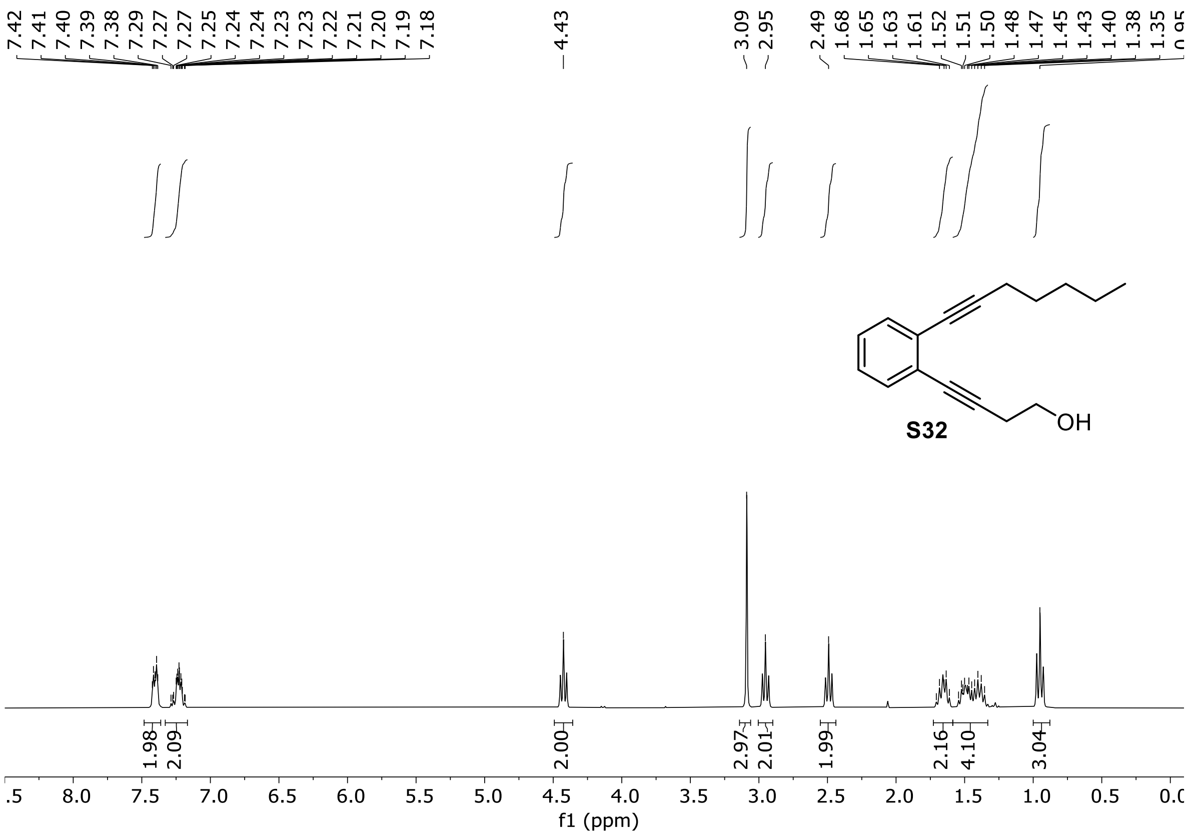

Figure S65. ^{1}H NMR spectrum of **S32** ($CDCl_3$, 300 MHz, 298 K)

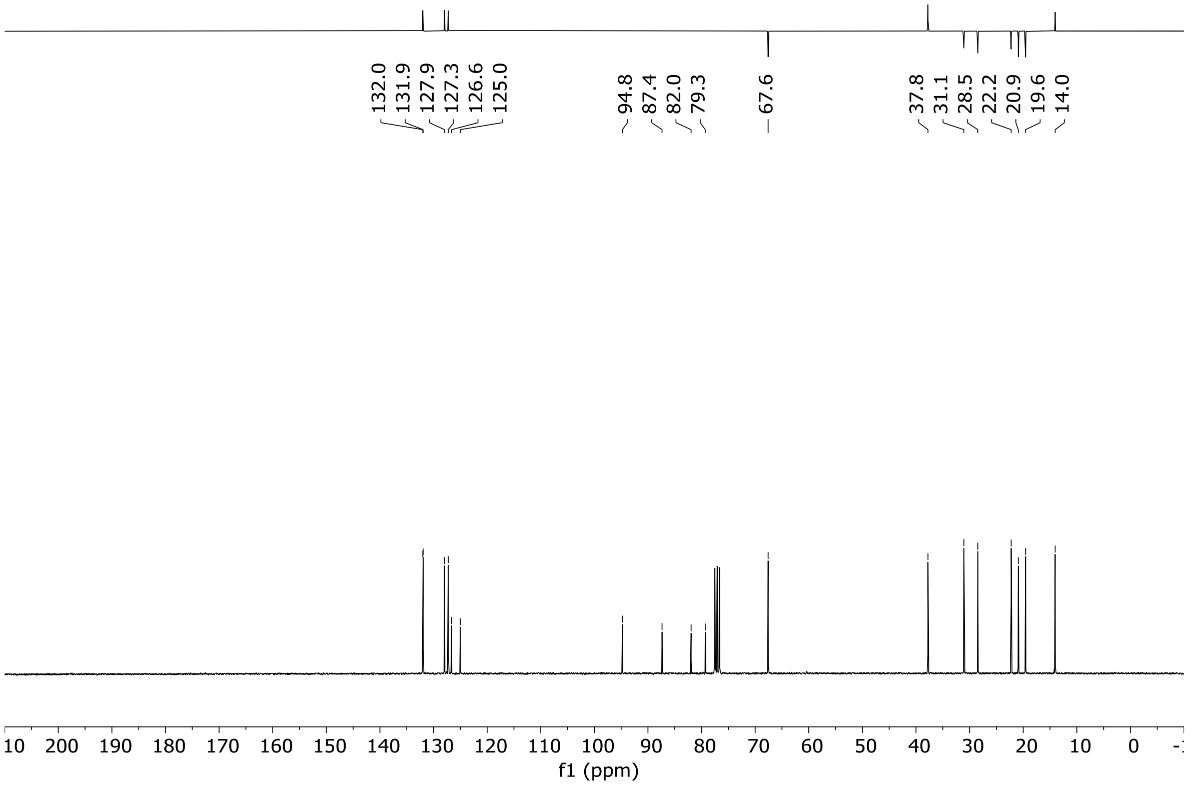

Figure S66. $^{13}C\{1H\}$ NMR spectrum of **S32** ($CDCl_3$, 75 MHz, 298 K)

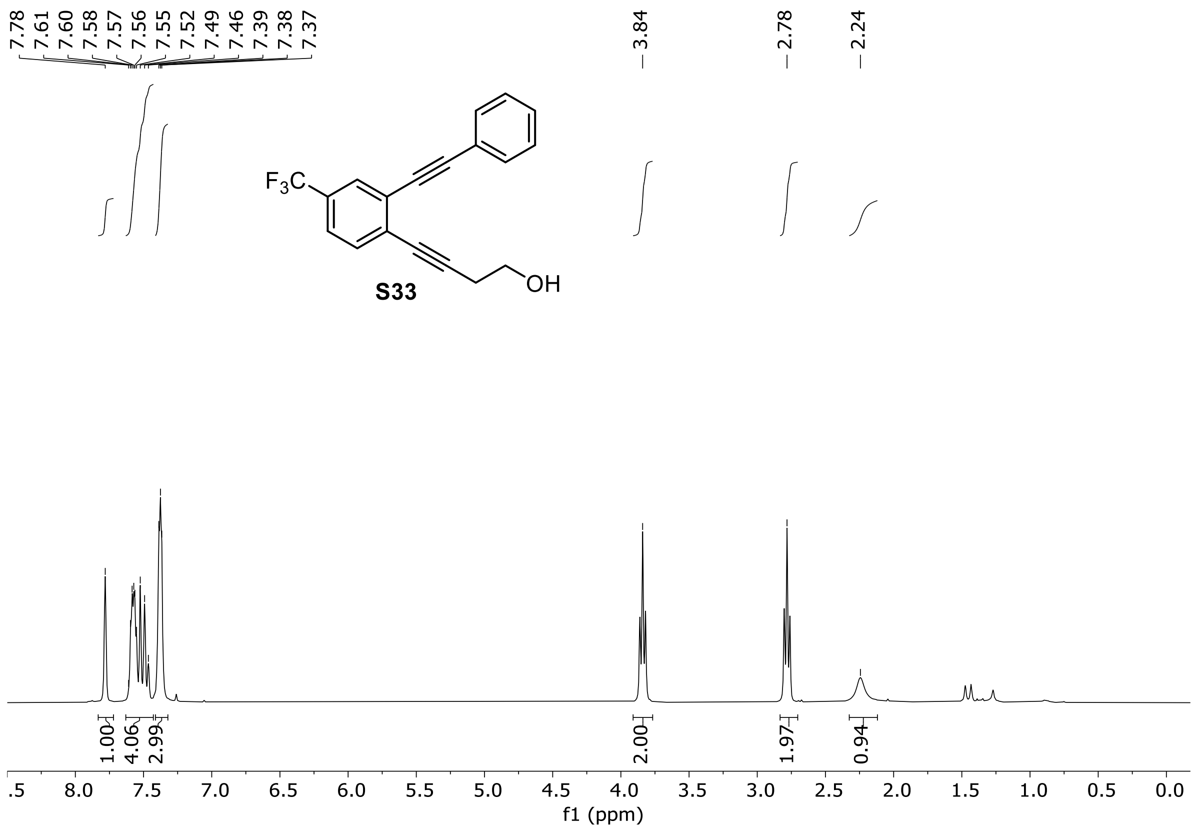

Figure S67. ^{1}H NMR spectrum of **S33** ($CDCl_3$, 300 MHz, 298 K)

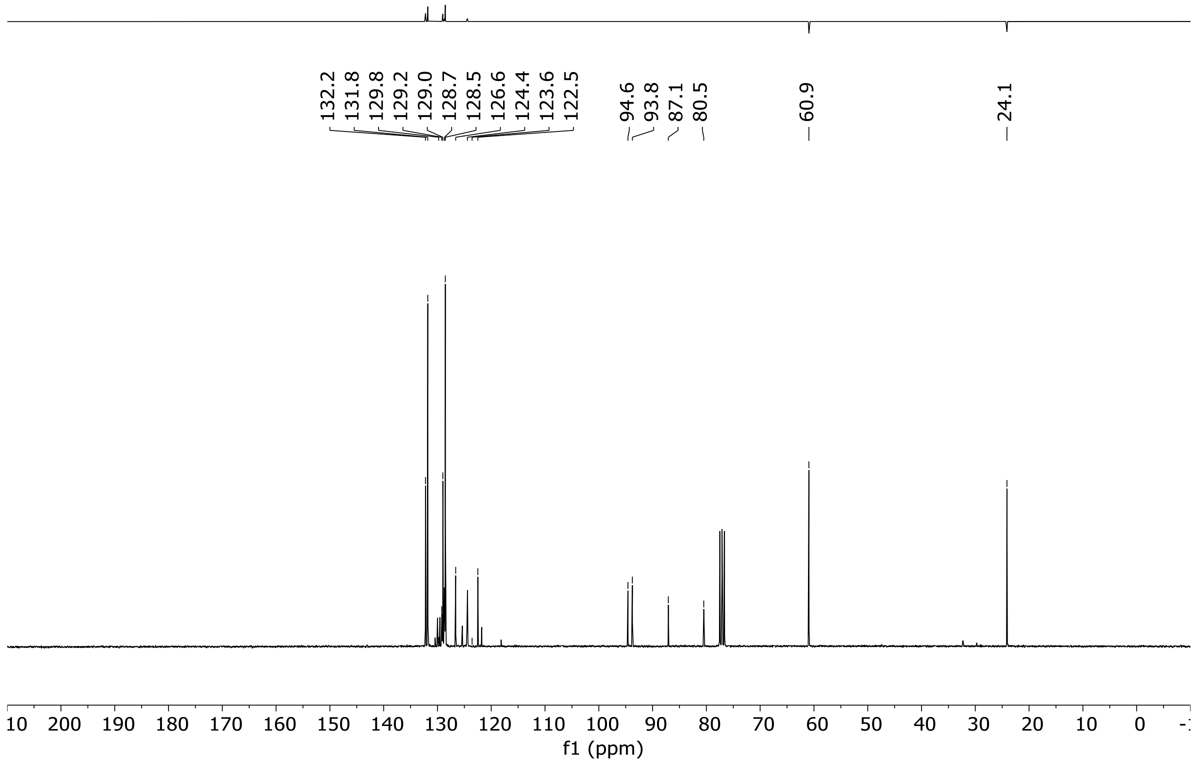

Figure S68. $^{13}C\{1H\}$ NMR spectrum of **S33** ($CDCl_3$, 75 MHz, 298 K)

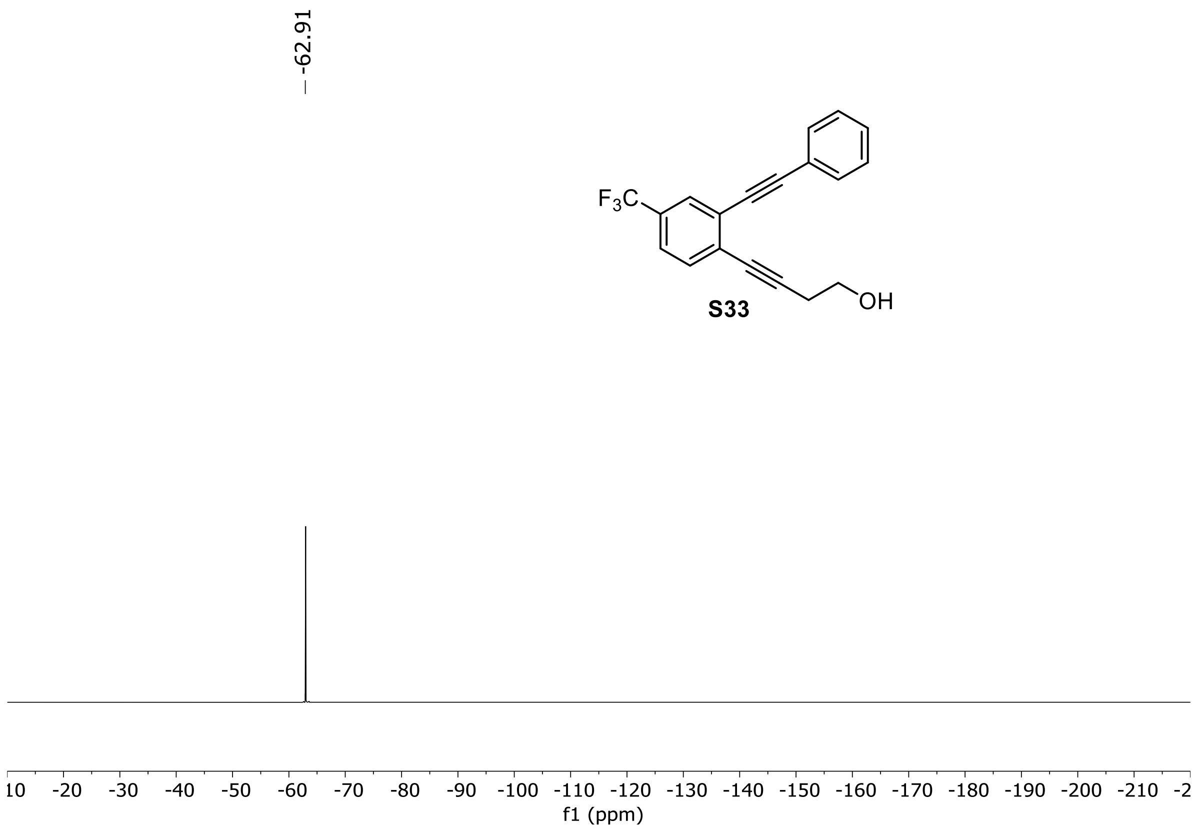

Figure S69. ^{19}F NMR spectrum of **S33** ($CDCl_3$, 282 MHz, 298 K)

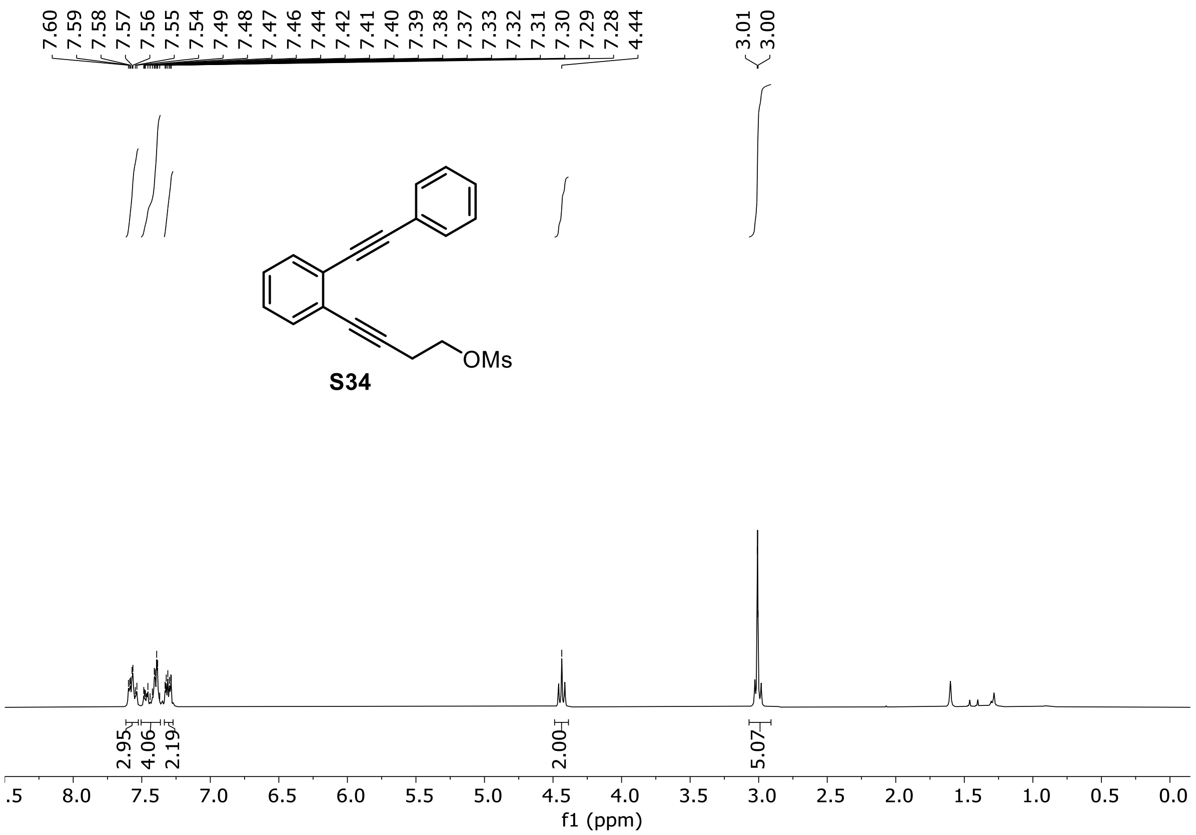

Figure S70. ^{1}H NMR spectrum of **S34** ($CDCl_3$, 300 MHz, 298 K)

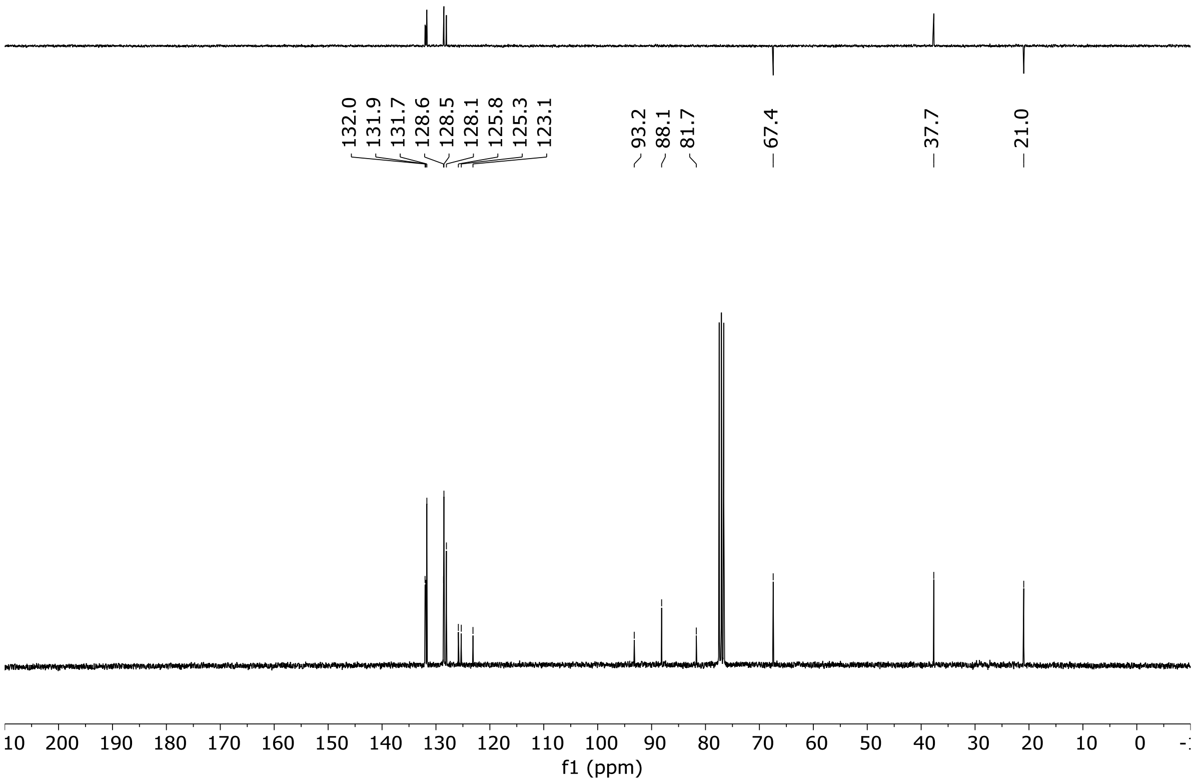

Figure S71. $^{13}C\{1H\}$ NMR spectrum of **S34** ($CDCl_3$, 75 MHz, 298 K)

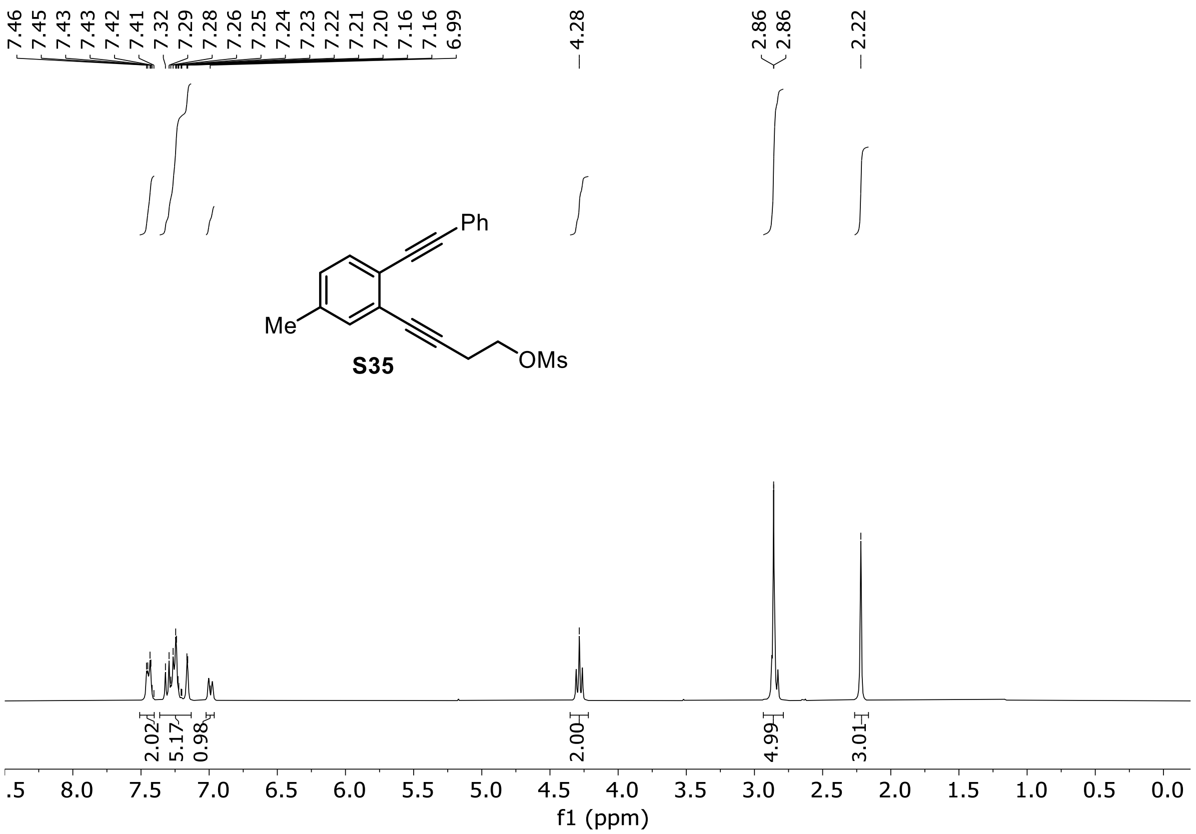

Figure S72. ^{1}H NMR spectrum of **S35** ($CDCl_3$, 300 MHz, 298 K)

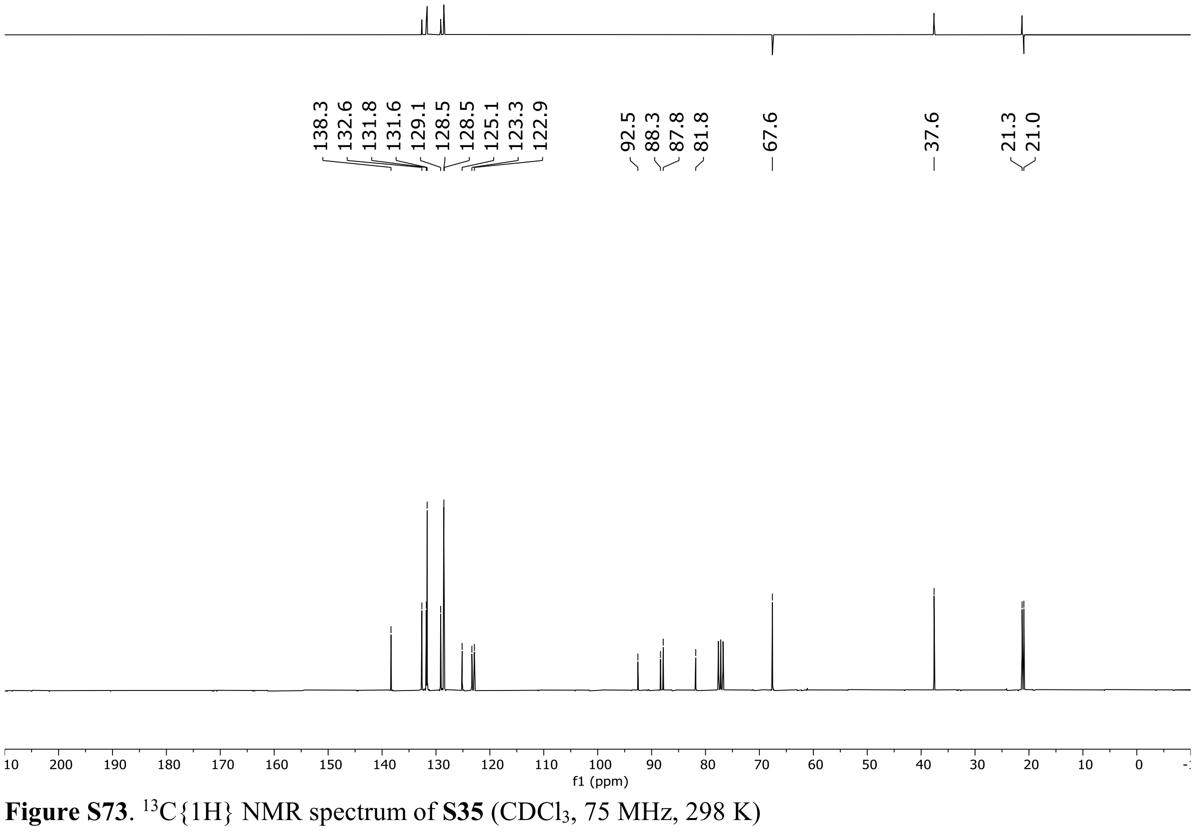

Figure S73. $^{13}C\{1H\}$ NMR spectrum of **S35** ($CDCl_3$, 75 MHz, 298 K)

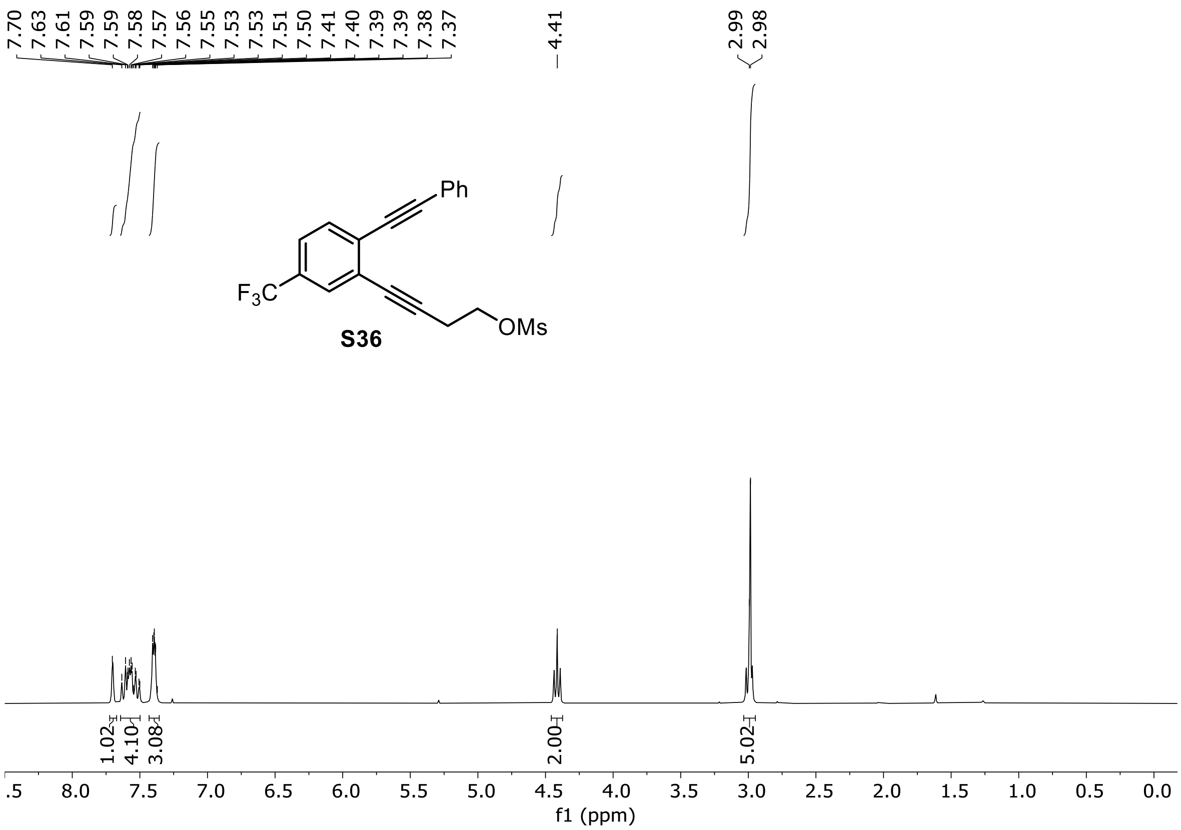

Figure S74. ^{1}H NMR spectrum of **S36** ($CDCl_3$, 300 MHz, 298 K)

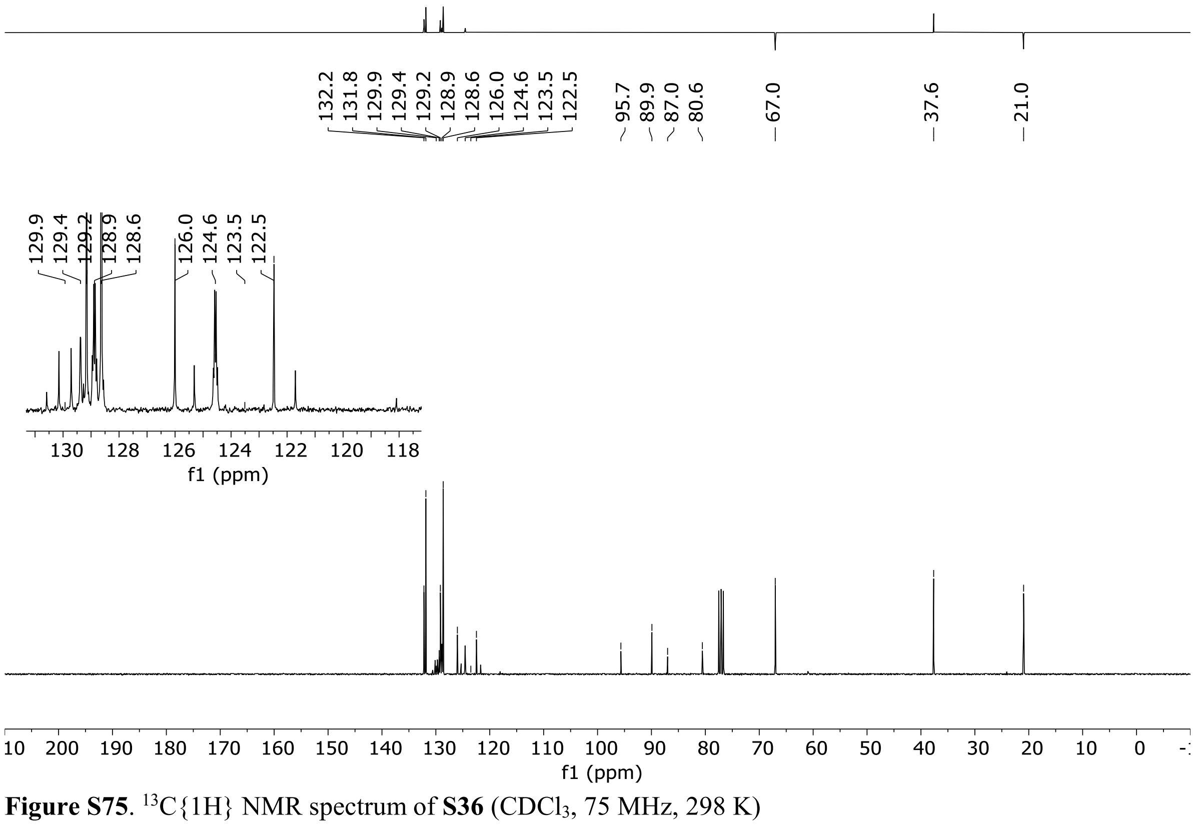

Figure S75. ^{13}C{1H} NMR spectrum of **S36** ($CDCl_3$, 75 MHz, 298 K)

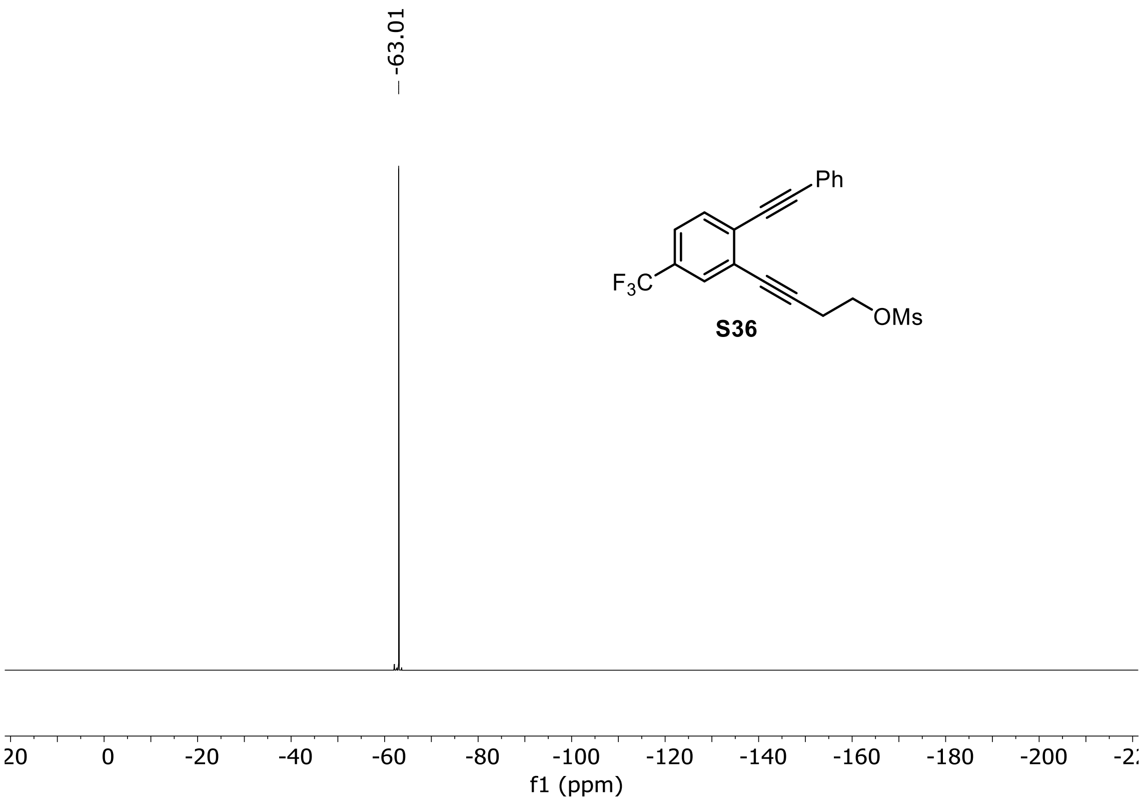

Figure S76. ^{19}F NMR spectrum of **S36** ($CDCl_3$, 282 MHz, 298 K)

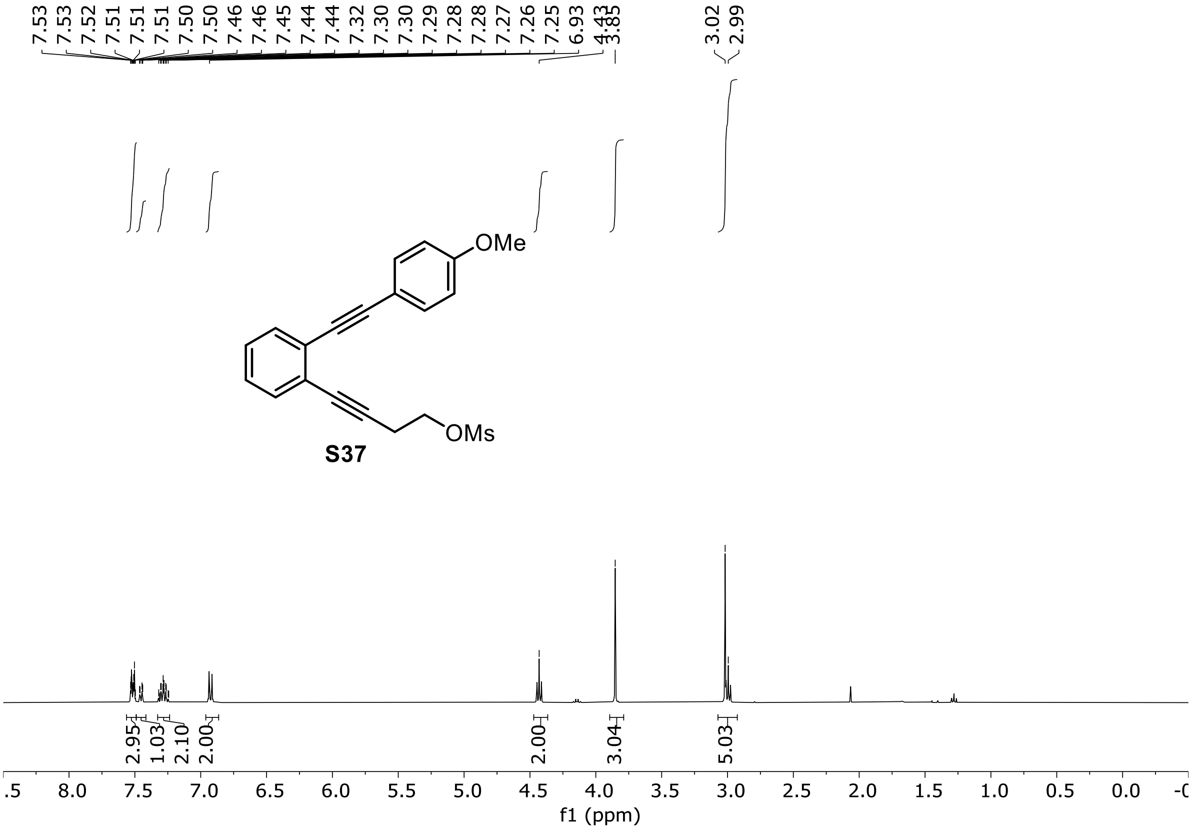

Figure S77. ^{1}H NMR spectrum of **S37** ($CDCl_3$, 300 MHz, 298 K)

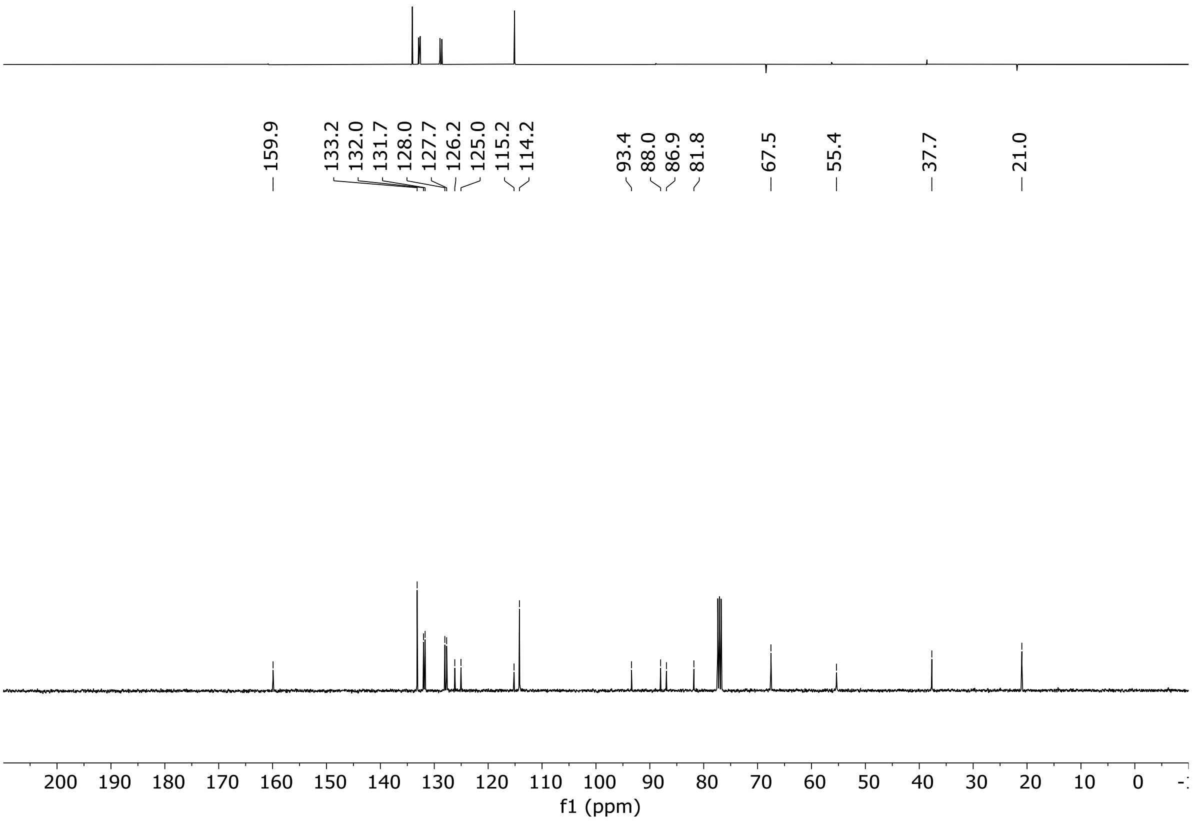

Figure S78. ^{13}C{1H} NMR spectrum of **S37** ($CDCl_3$, 75 MHz, 298 K)

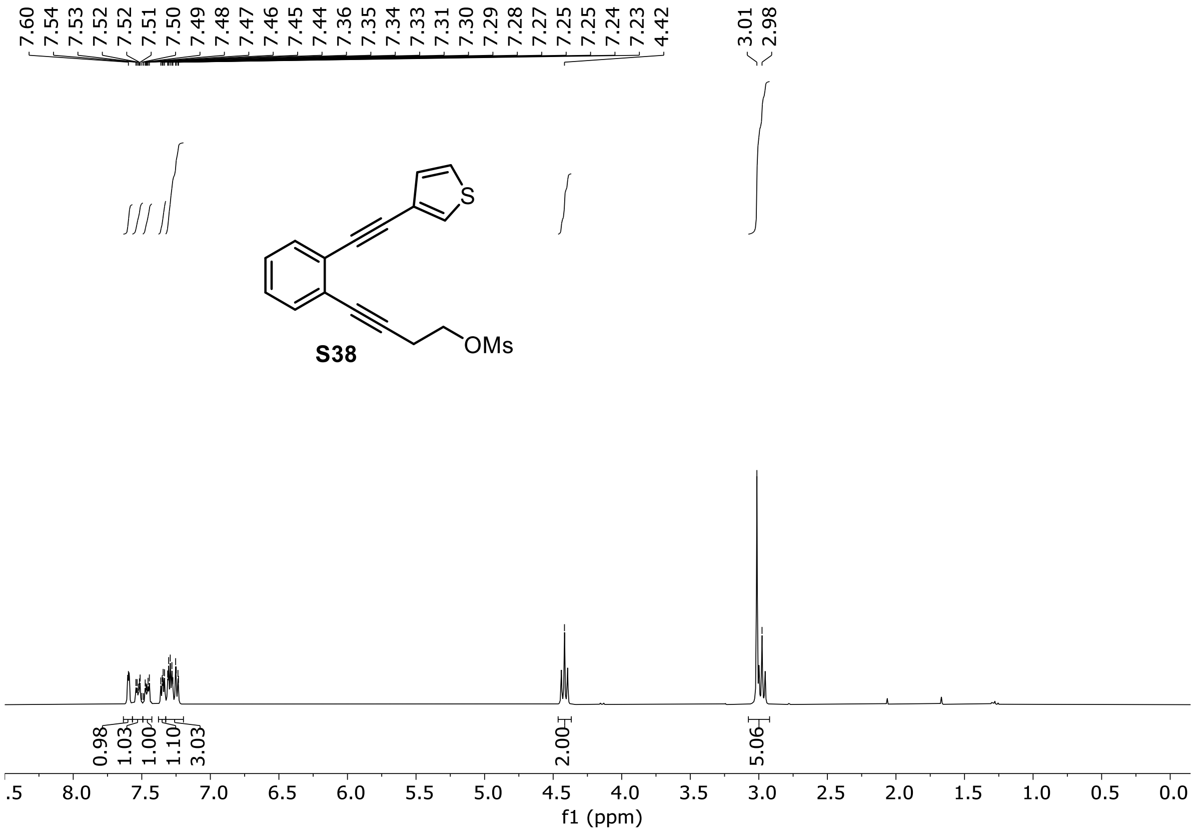

Figure S79. ^{1}H NMR spectrum of **S38** ($CDCl_3$, 300 MHz, 298 K)

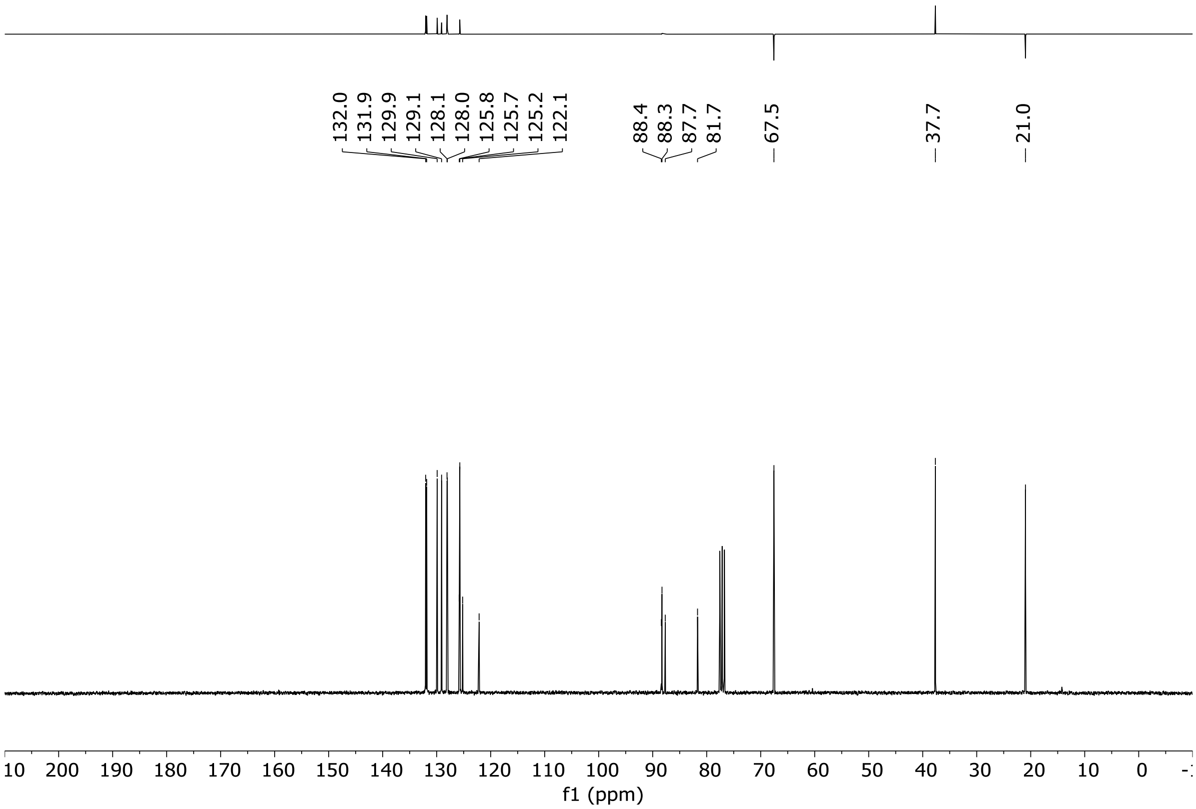

Figure S80. ^{13}C{1H} NMR spectrum of **S38** ($CDCl_3$, 75 MHz, 298 K)

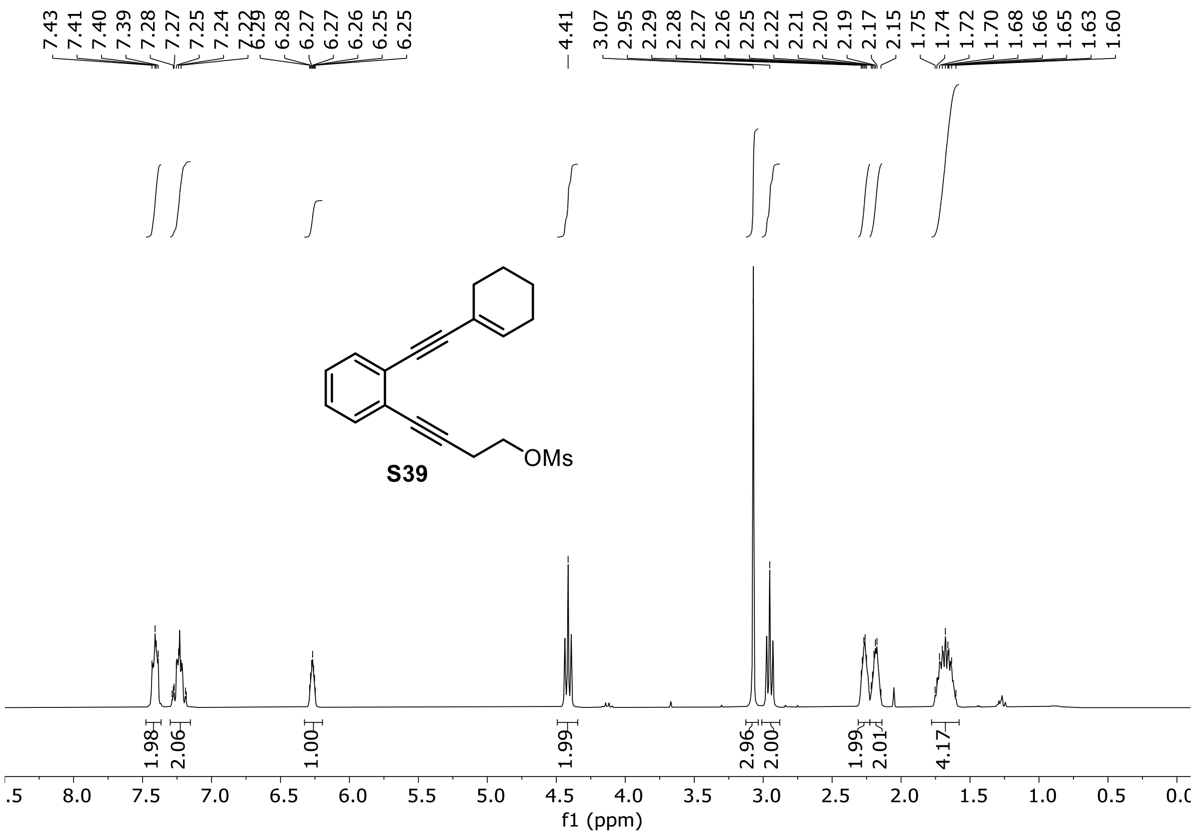

Figure S81. ^{1}H NMR spectrum of **S39** ($CDCl_3$, 300 MHz, 298 K)

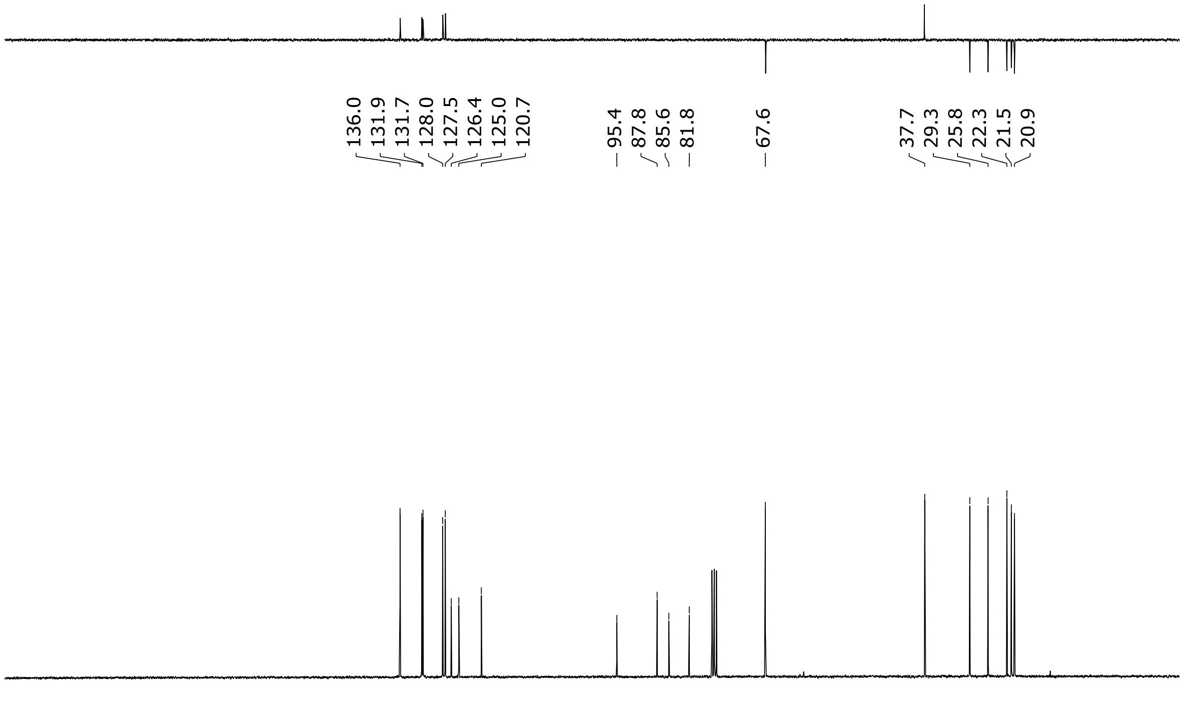

Figure S82. $^{13}C\{1H\}$ NMR spectrum of **S39** ($CDCl_3$, 75 MHz, 298 K)

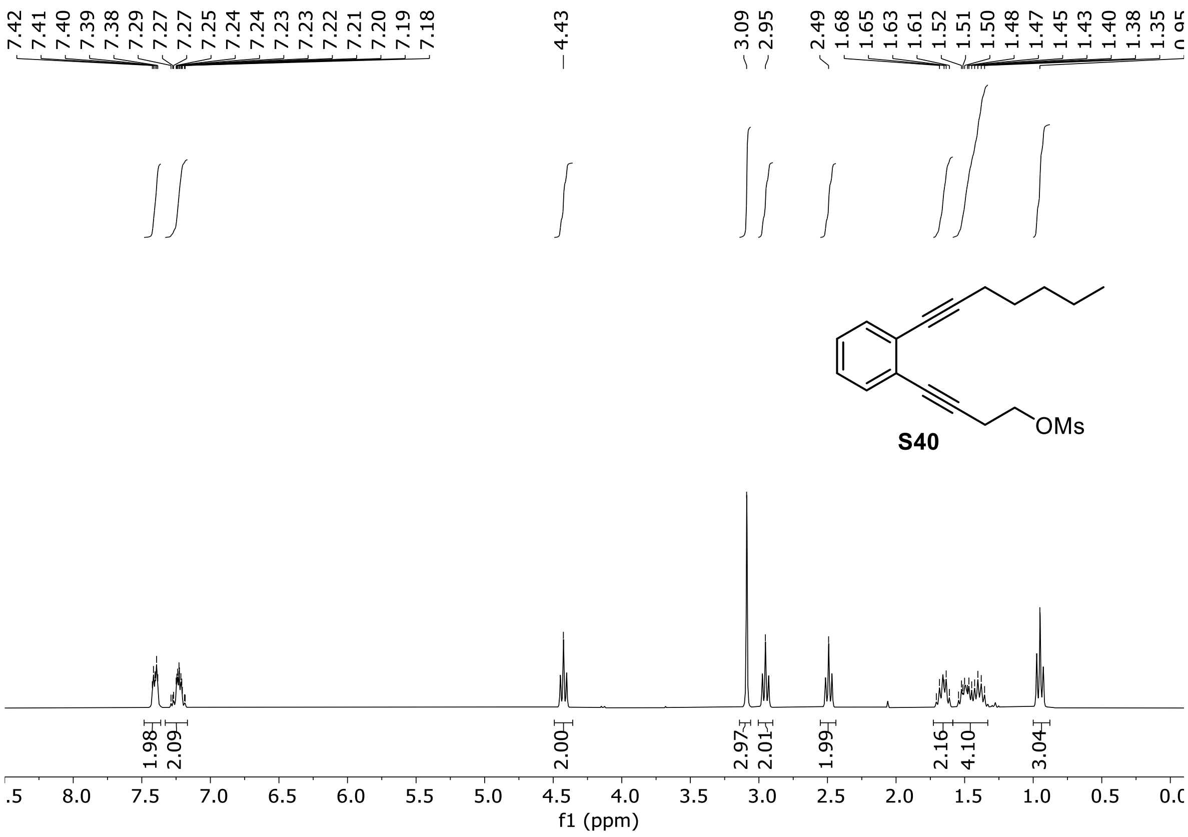

Figure S83. ^{1}H NMR spectrum of **S40** ($CDCl_3$, 300 MHz, 298 K)

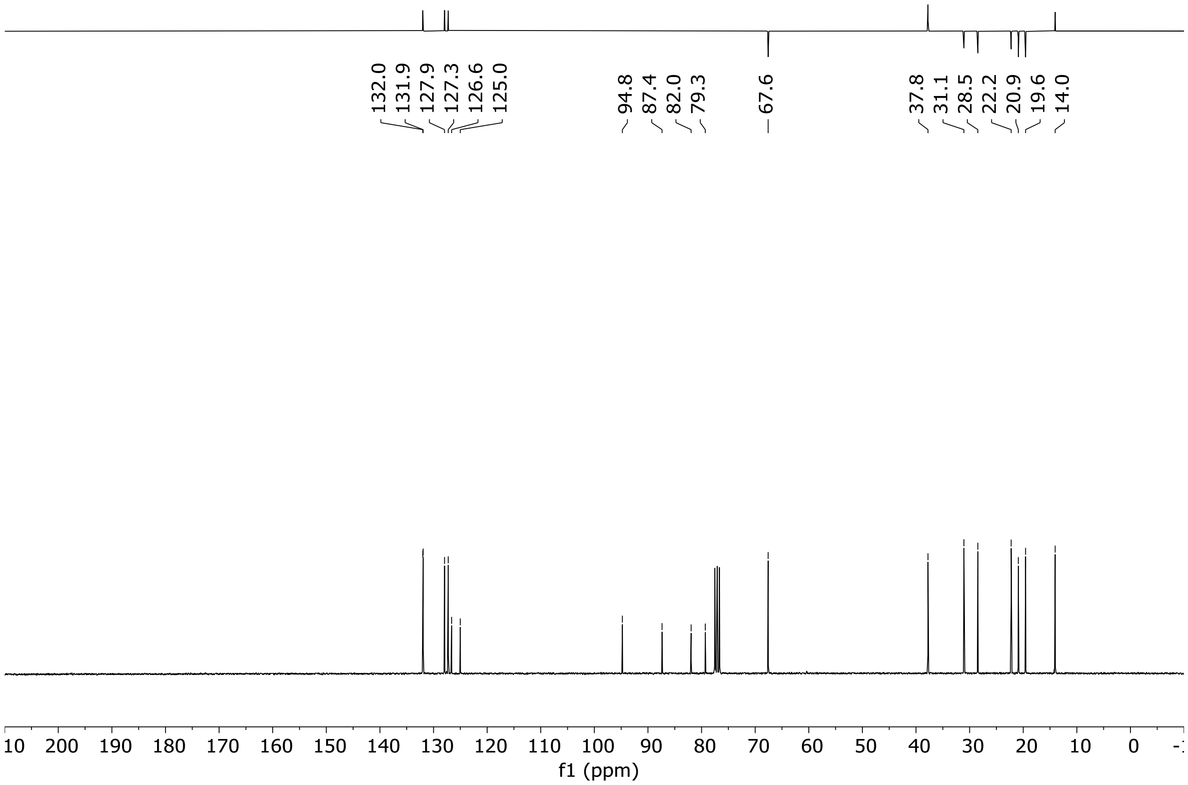

Figure S84. $^{13}C\{1H\}$ NMR spectrum of **S40** ($CDCl_3$, 75 MHz, 298 K)

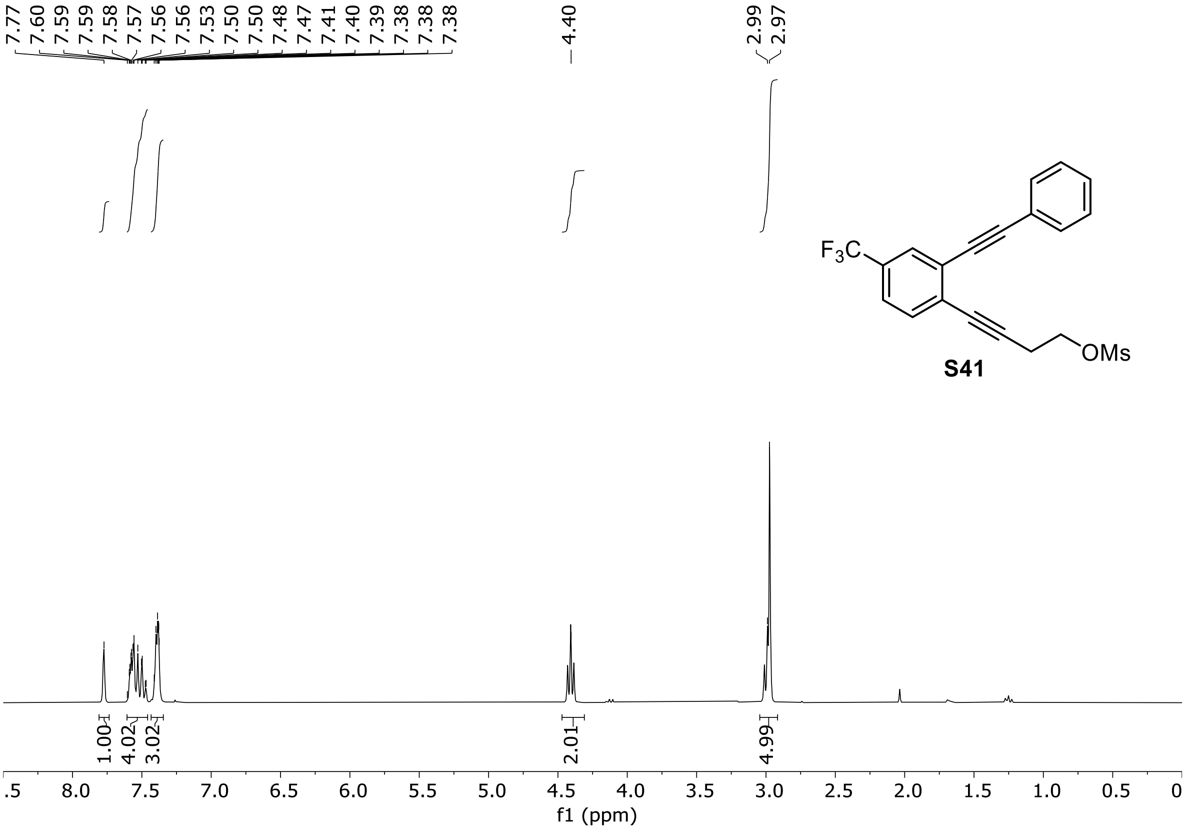

Figure S85. ^{1}H NMR spectrum of **S41** ($CDCl_3$, 300 MHz, 298 K)

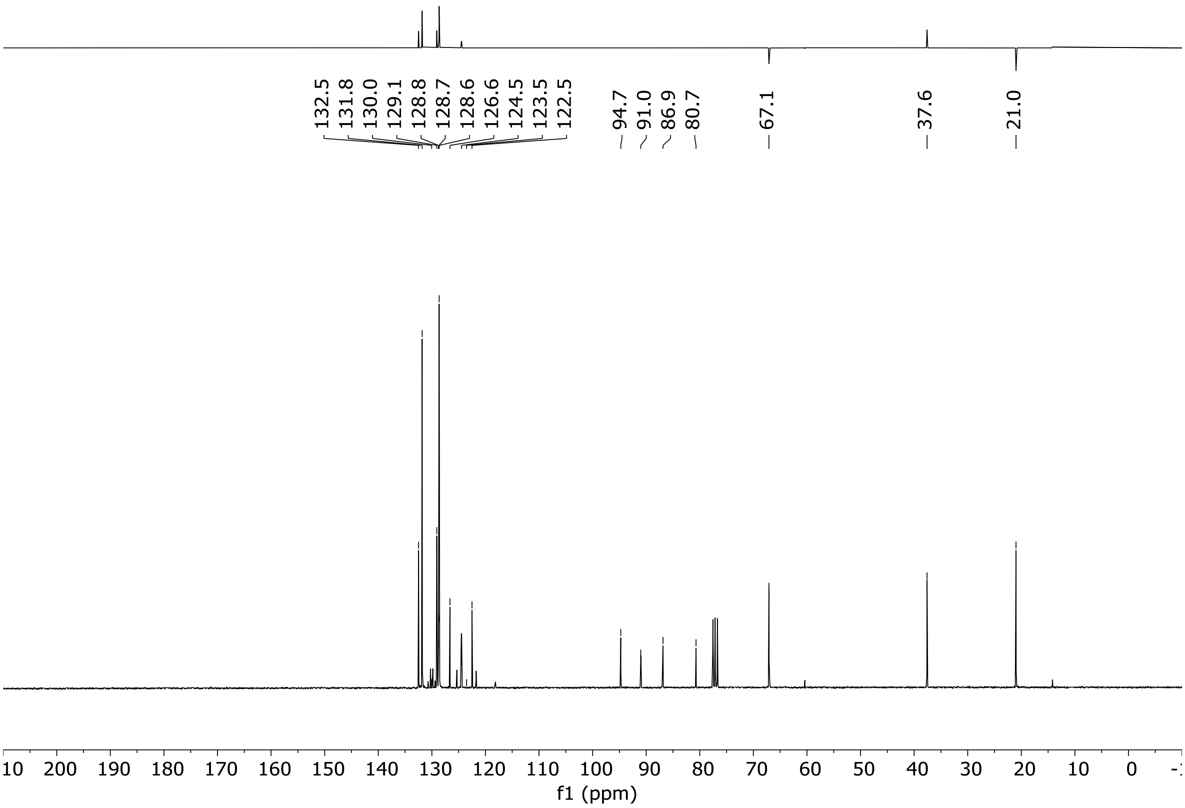

Figure S86. $^{13}C\{1H\}$ NMR spectrum of **S41** ($CDCl_3$, 75 MHz, 298 K)

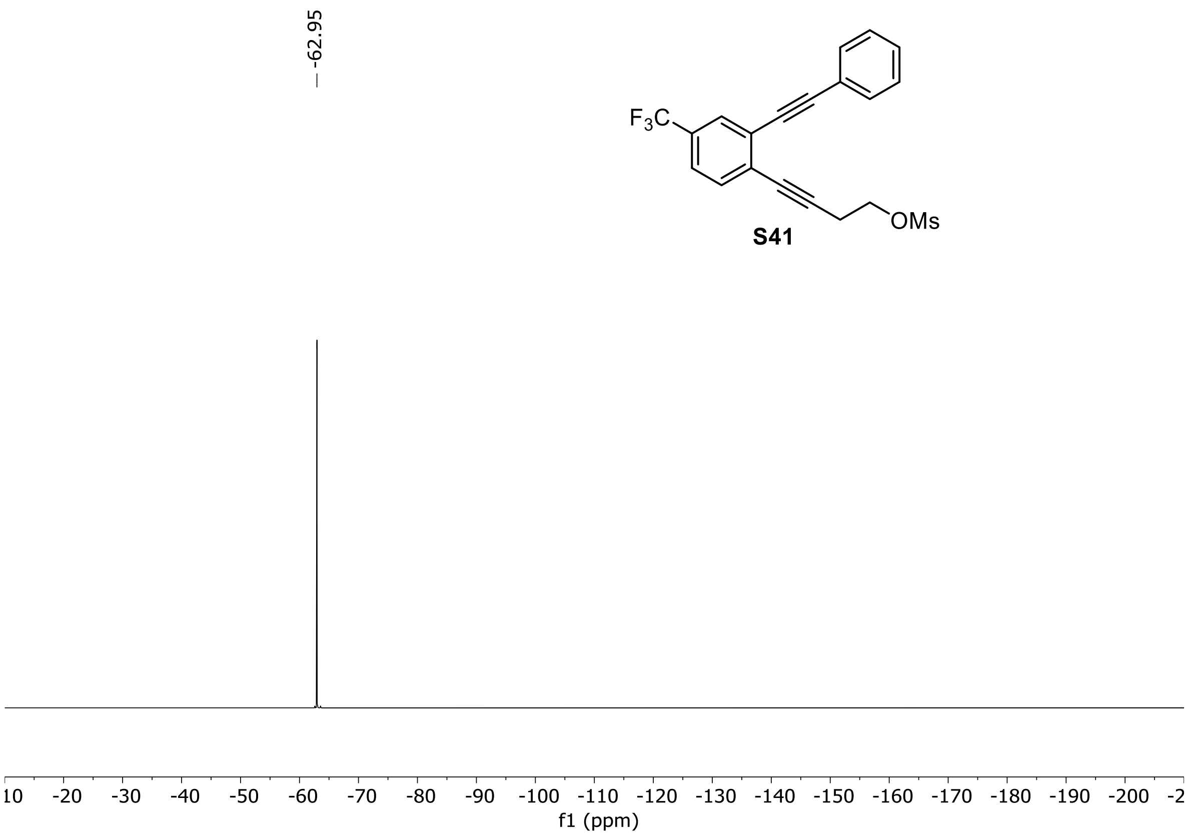

Figure S87. ^{19}F NMR spectrum of **S41** ($CDCl_3$, 282 MHz, 298 K)

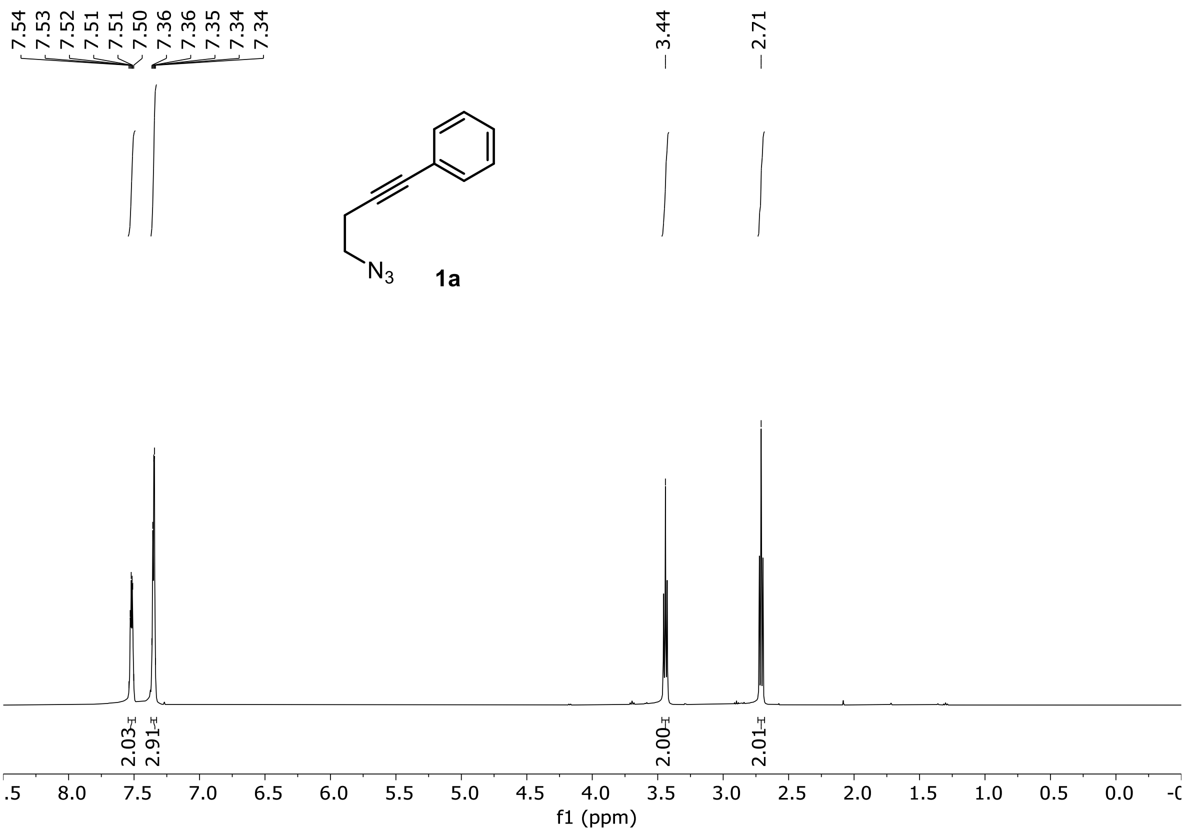

Figure S88. ^{1}H NMR spectrum of **1a** ($CDCl_3$, 300 MHz, 298 K)

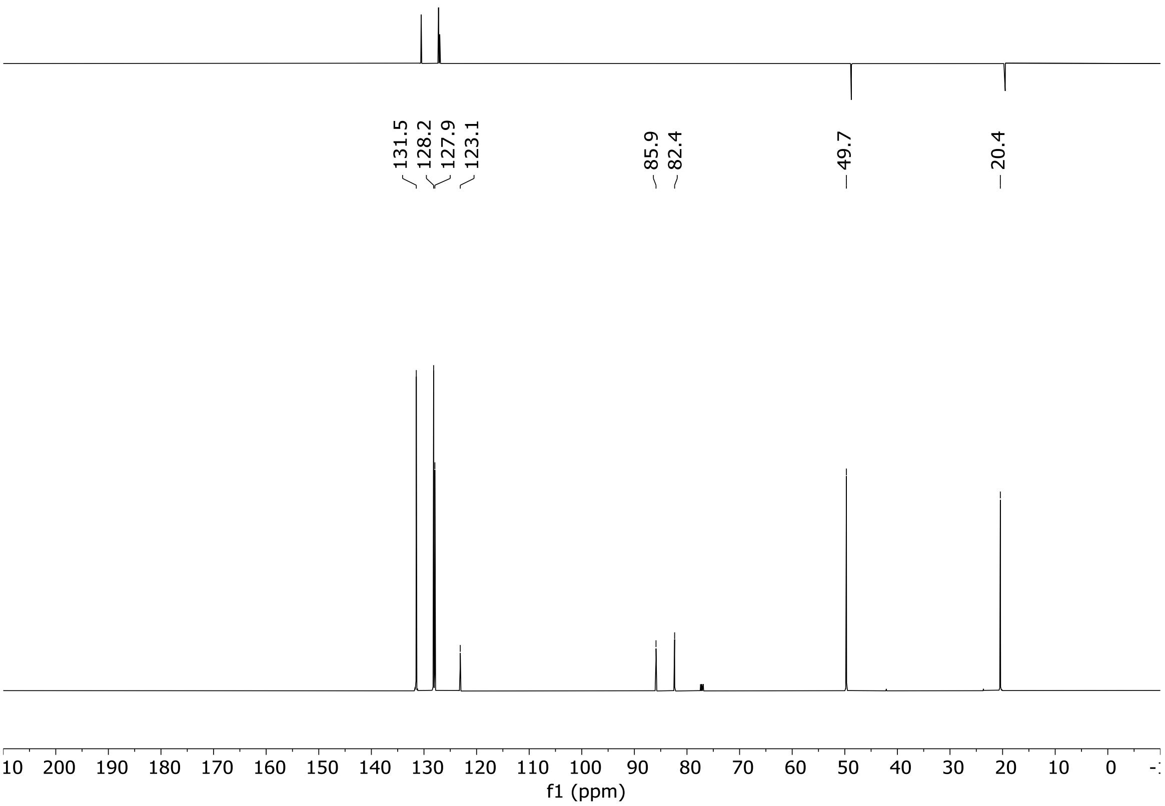

Figure S89. $^{13}C\{1H\}$ NMR spectrum of **1a** ($CDCl_3$, 75 MHz, 298 K)

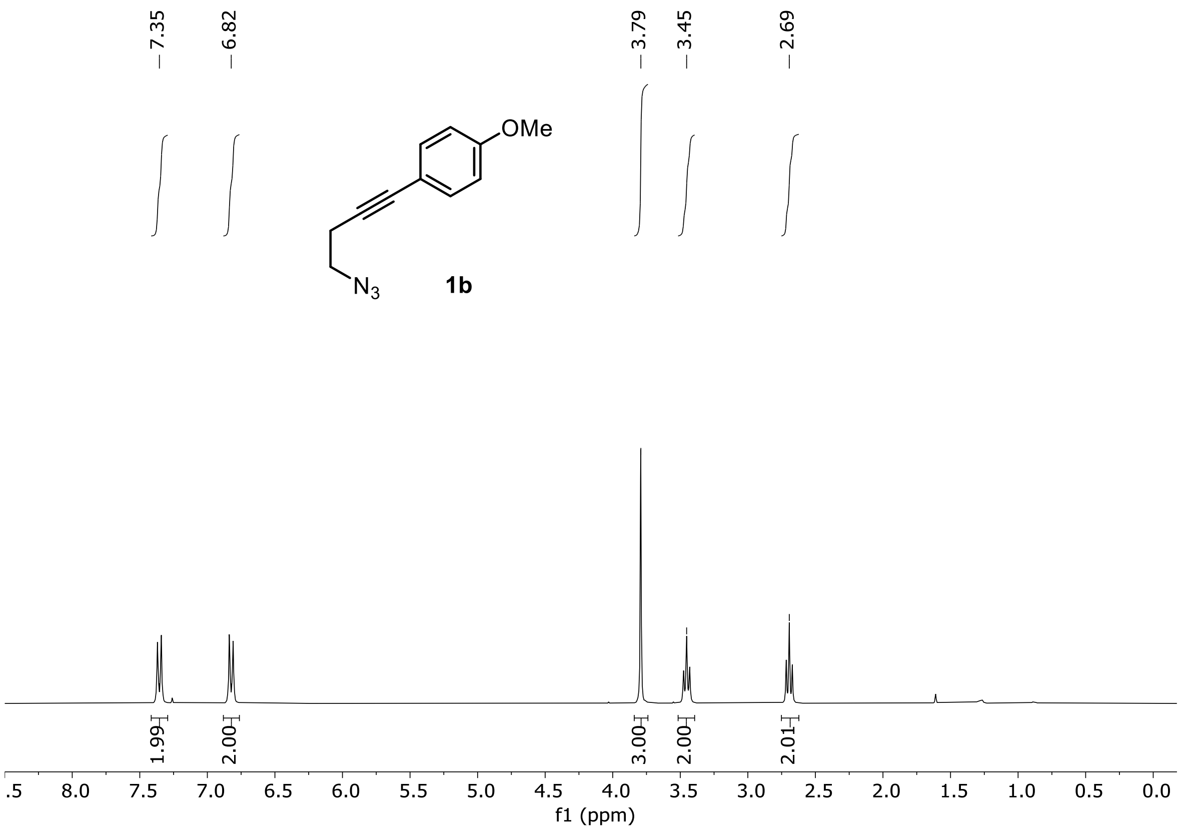

Figure S90. ^{1}H NMR spectrum of **1b** ($CDCl_3$, 300 MHz, 298 K)

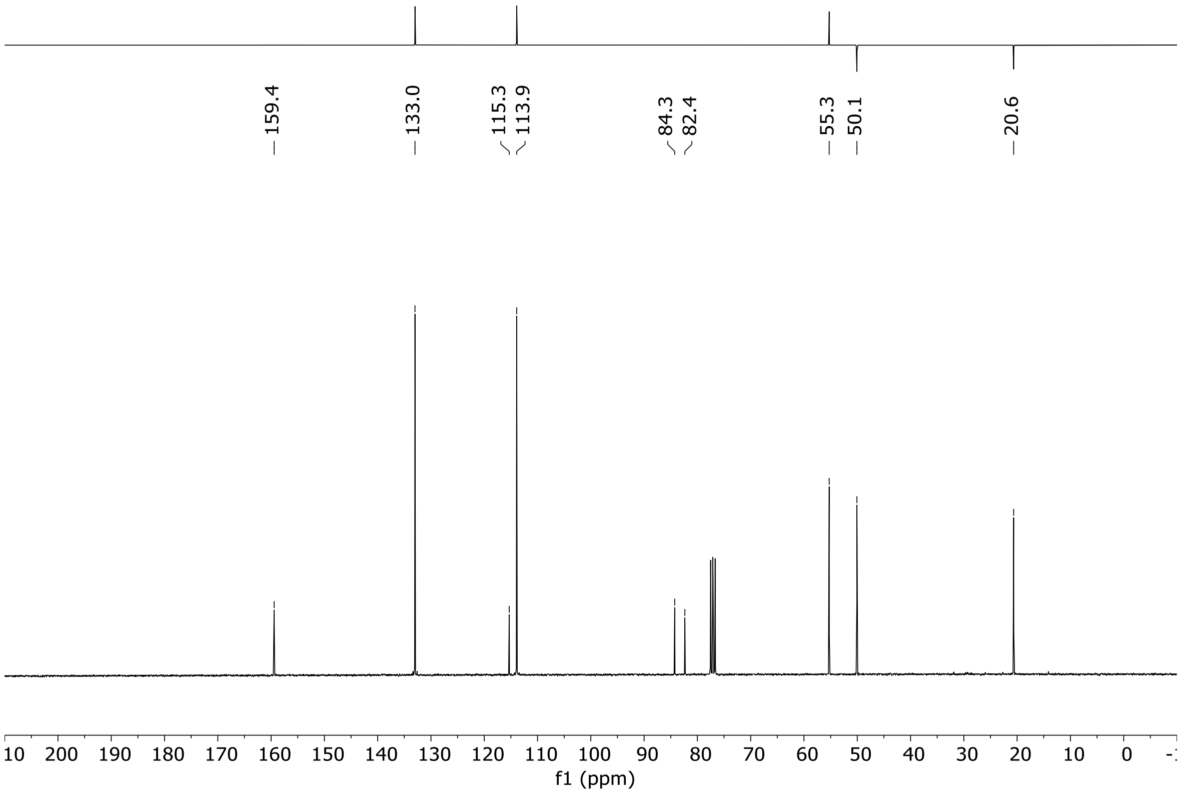

Figure S91. $^{13}C\{1H\}$ NMR spectrum of **1b** ($CDCl_3$, 75 MHz, 298 K)

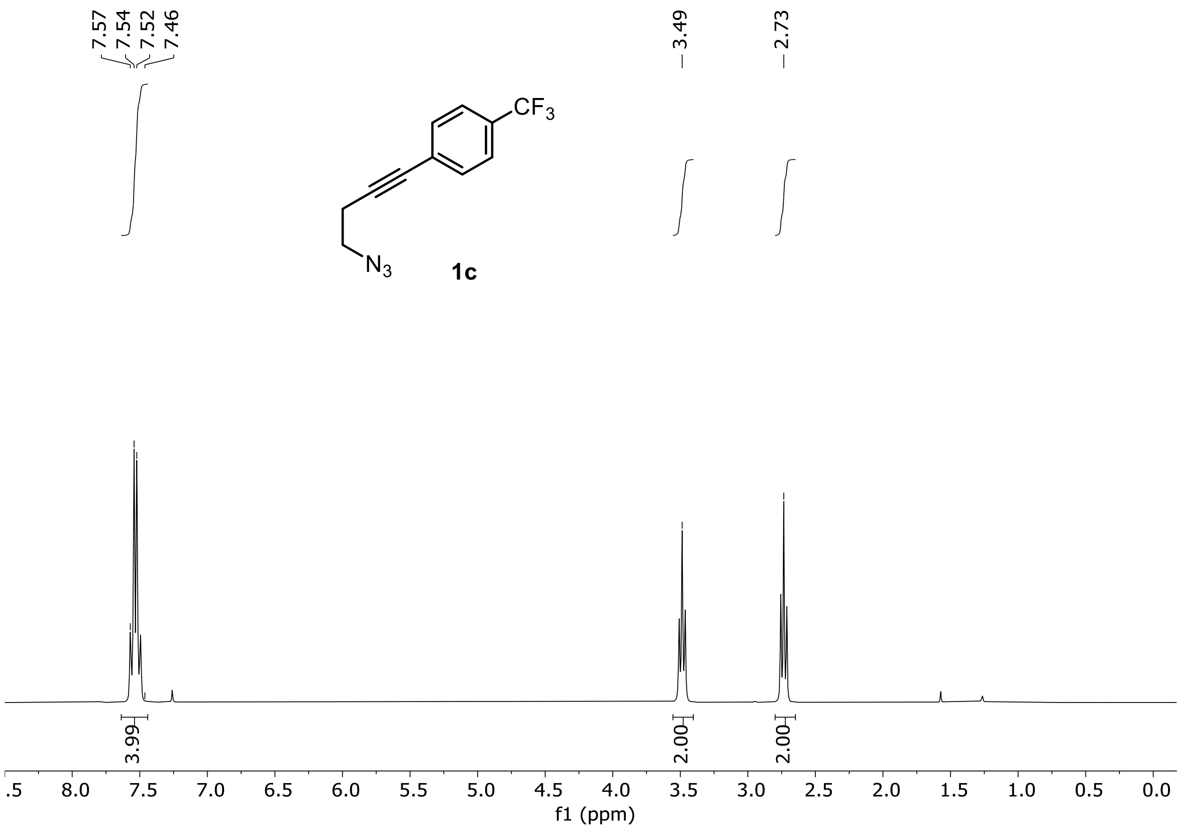

Figure S92. ^{1}H NMR spectrum of **1c** ($CDCl_3$, 300 MHz, 298 K)

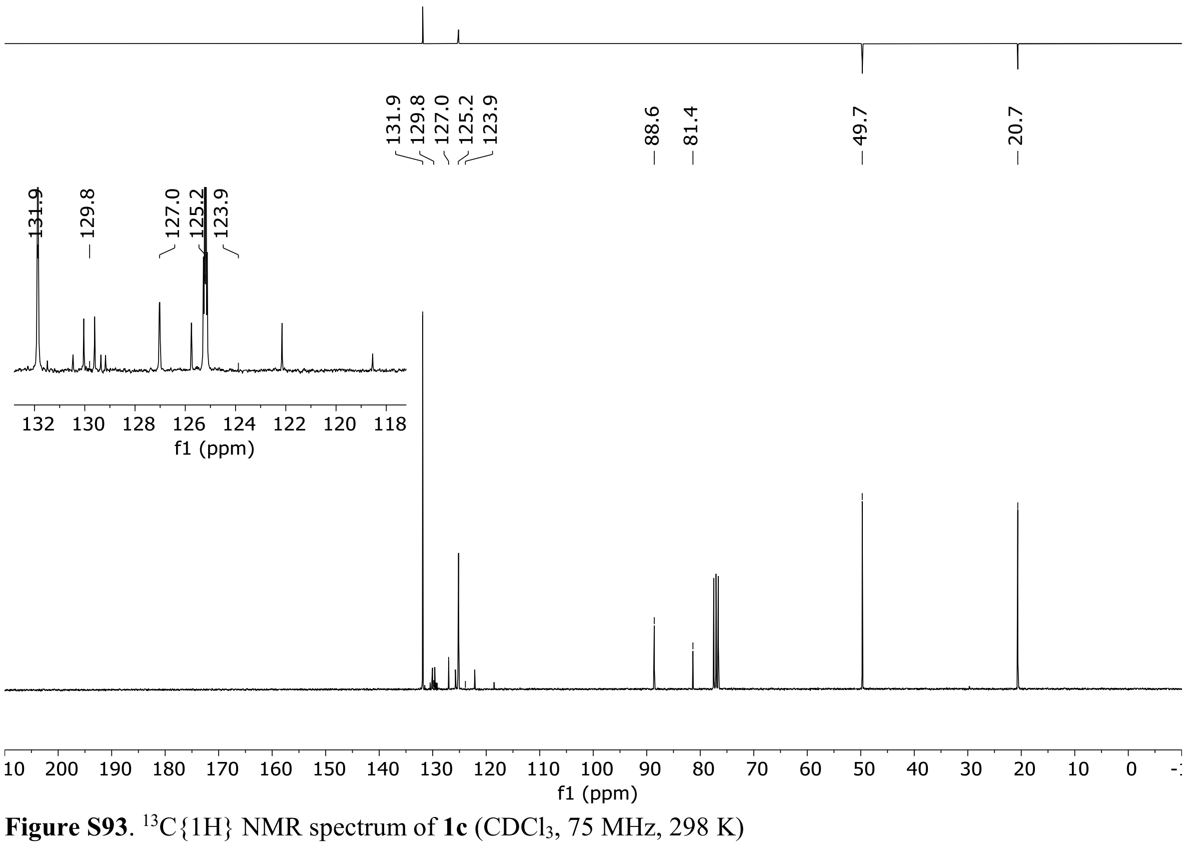

Figure S93. $^{13}C\{1H\}$ NMR spectrum of **1c** ($CDCl_3$, 75 MHz, 298 K)

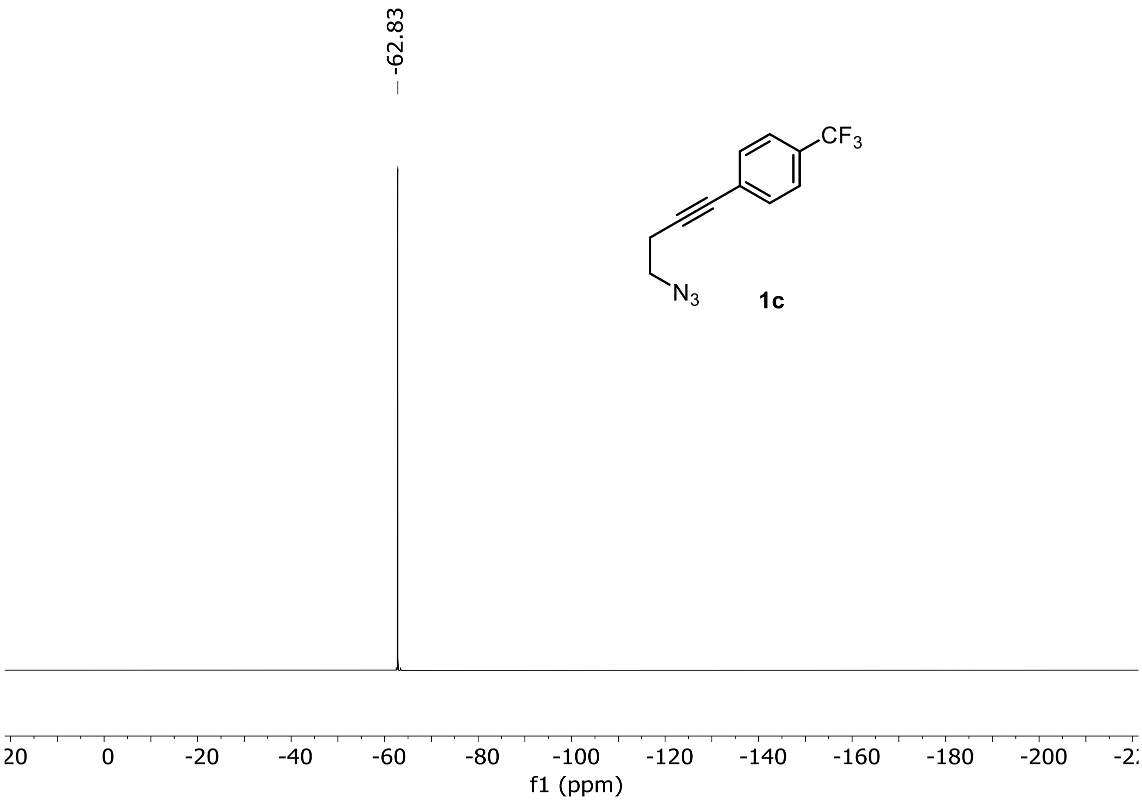

Figure S94. ^{19}F NMR spectrum of **1c** ($CDCl_3$, 282 MHz, 298 K)

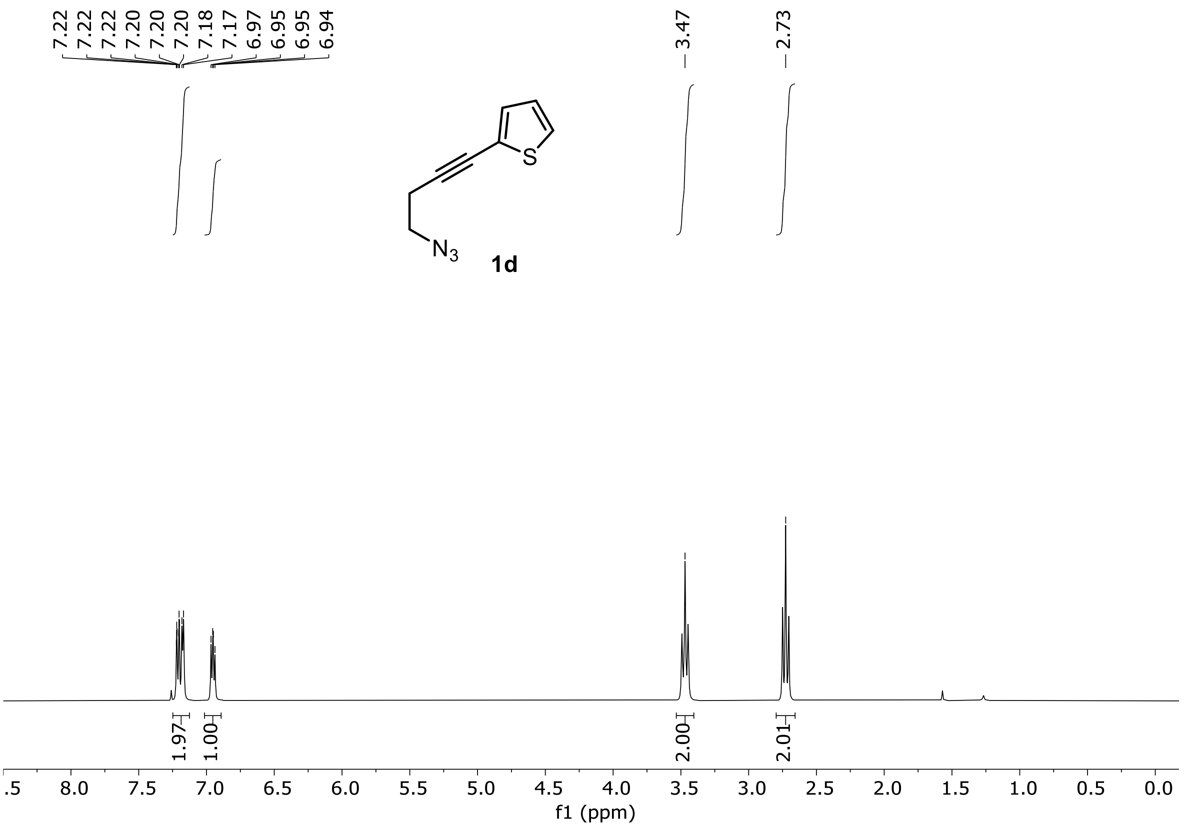

Figure S95. ^{1}H NMR spectrum of **1d** ($CDCl_3$, 300 MHz, 298 K)

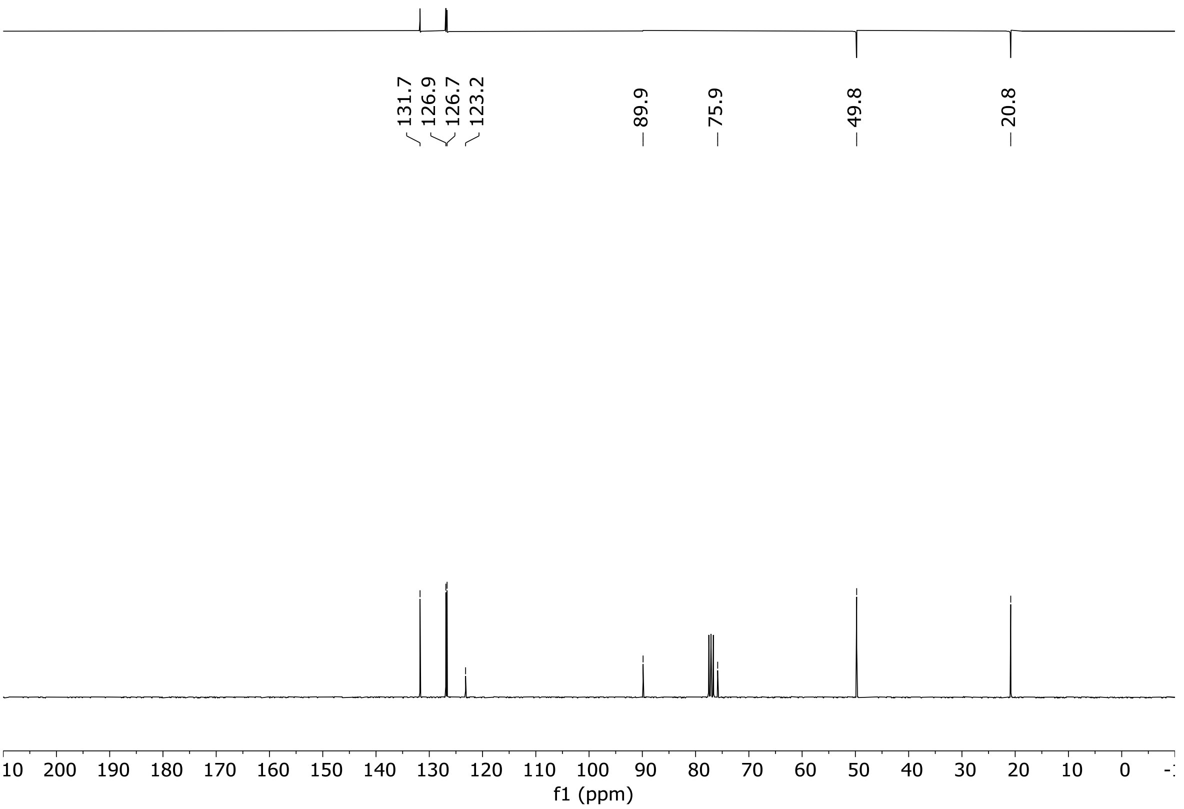

Figure S96. $^{13}C\{1H\}$ NMR spectrum of **1d** ($CDCl_3$, 75 MHz, 298 K)

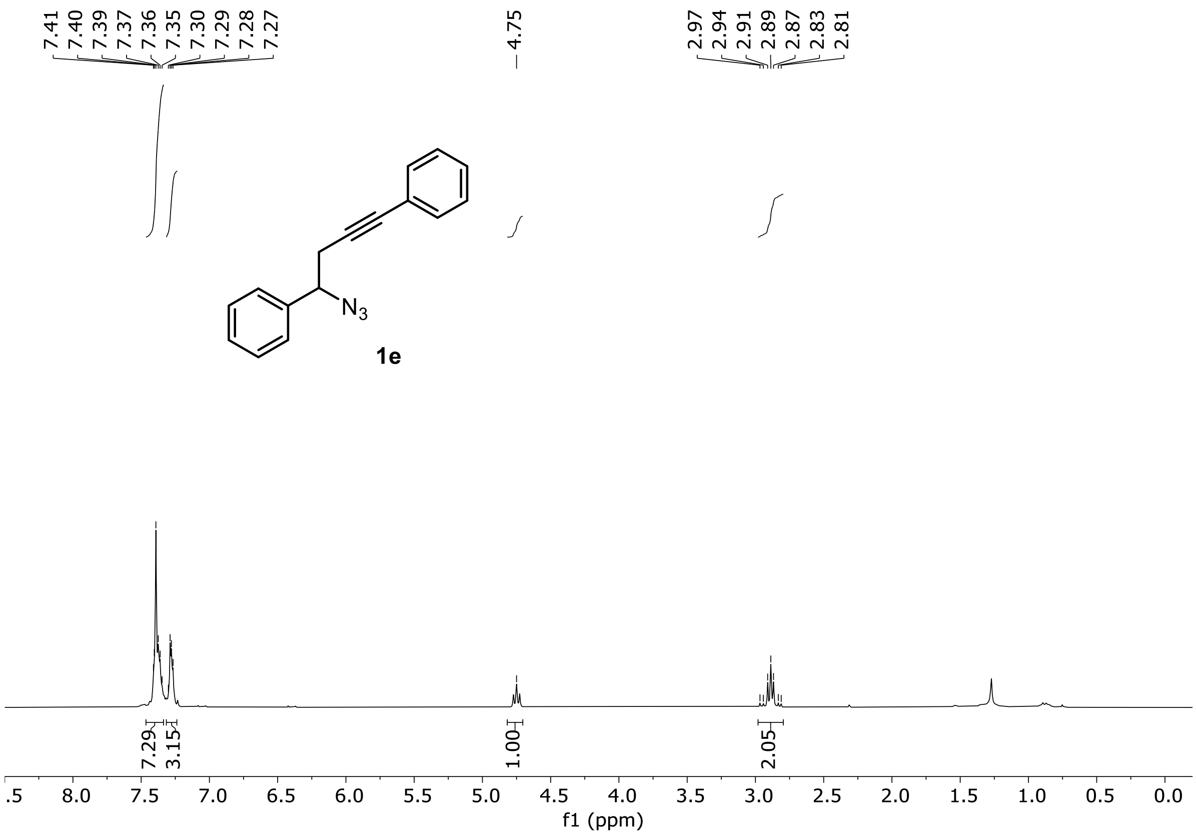

Figure S97. ^{1}H NMR spectrum of **1e** ($CDCl_3$, 300 MHz, 298 K)

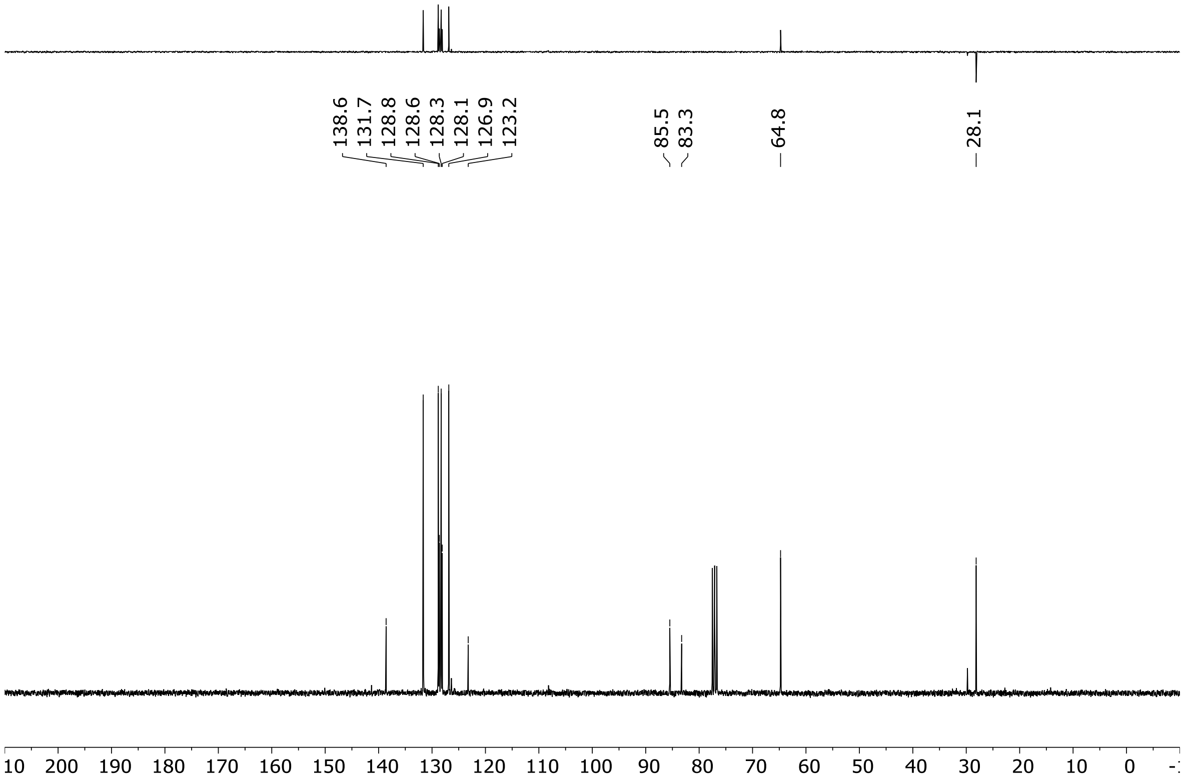

Figure S98. $^{13}C\{1H\}$ NMR spectrum of **1e** ($CDCl_3$, 75 MHz, 298 K)

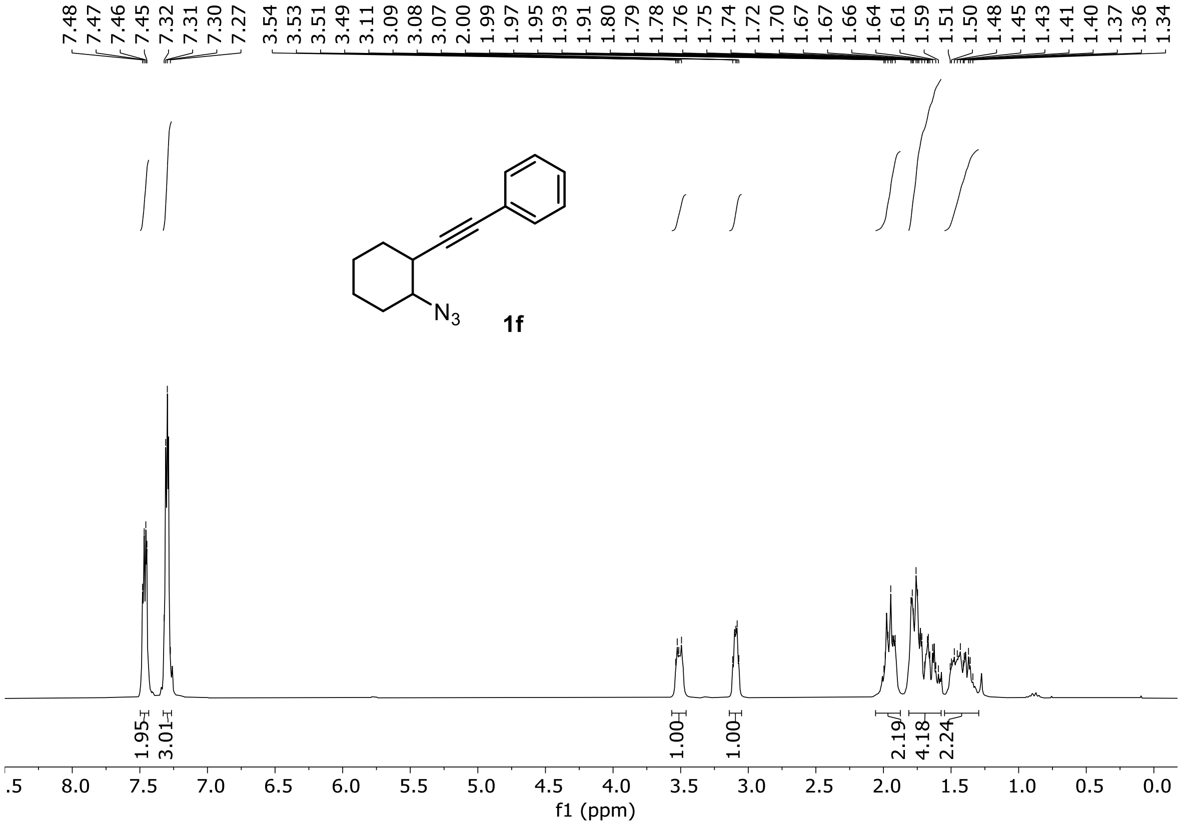

Figure S99. ^{1}H NMR spectrum of **1f** ($CDCl_3$, 300 MHz, 298 K)

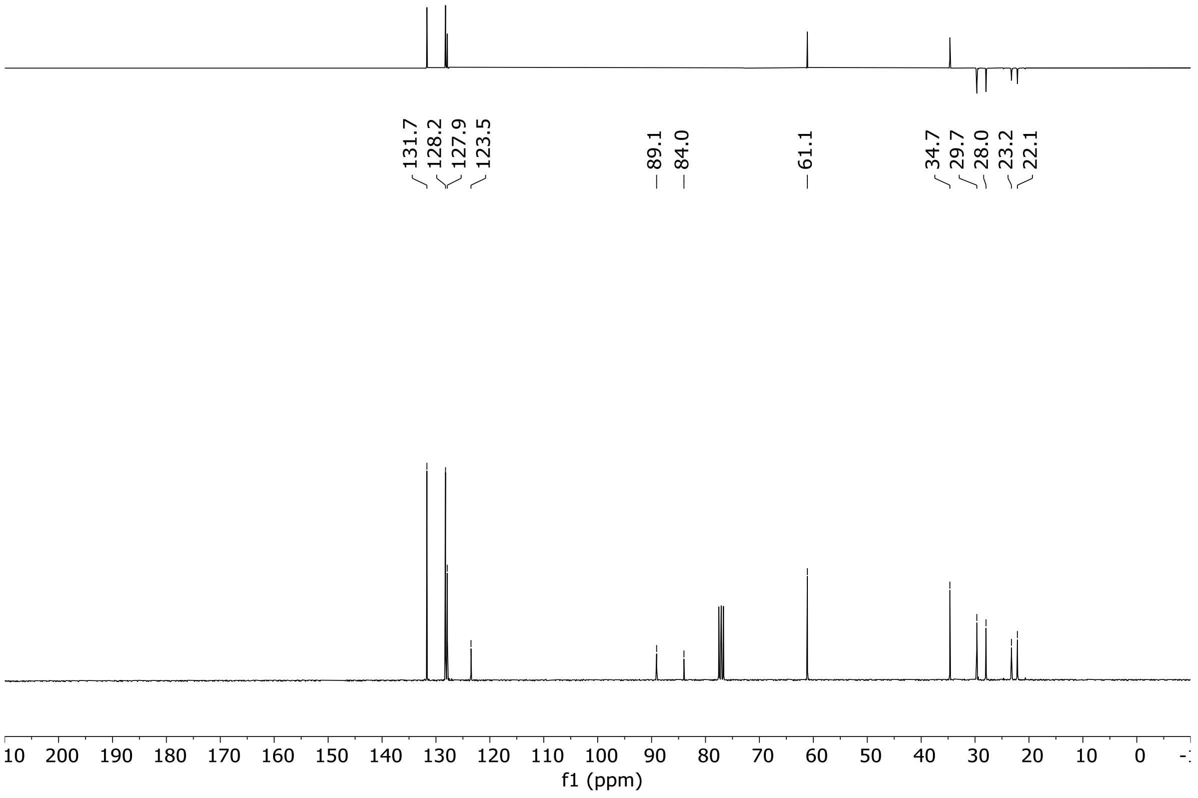

Figure S100. ^{13}C{1H} NMR spectrum of **1f** ($CDCl_3$, 75 MHz, 298 K)

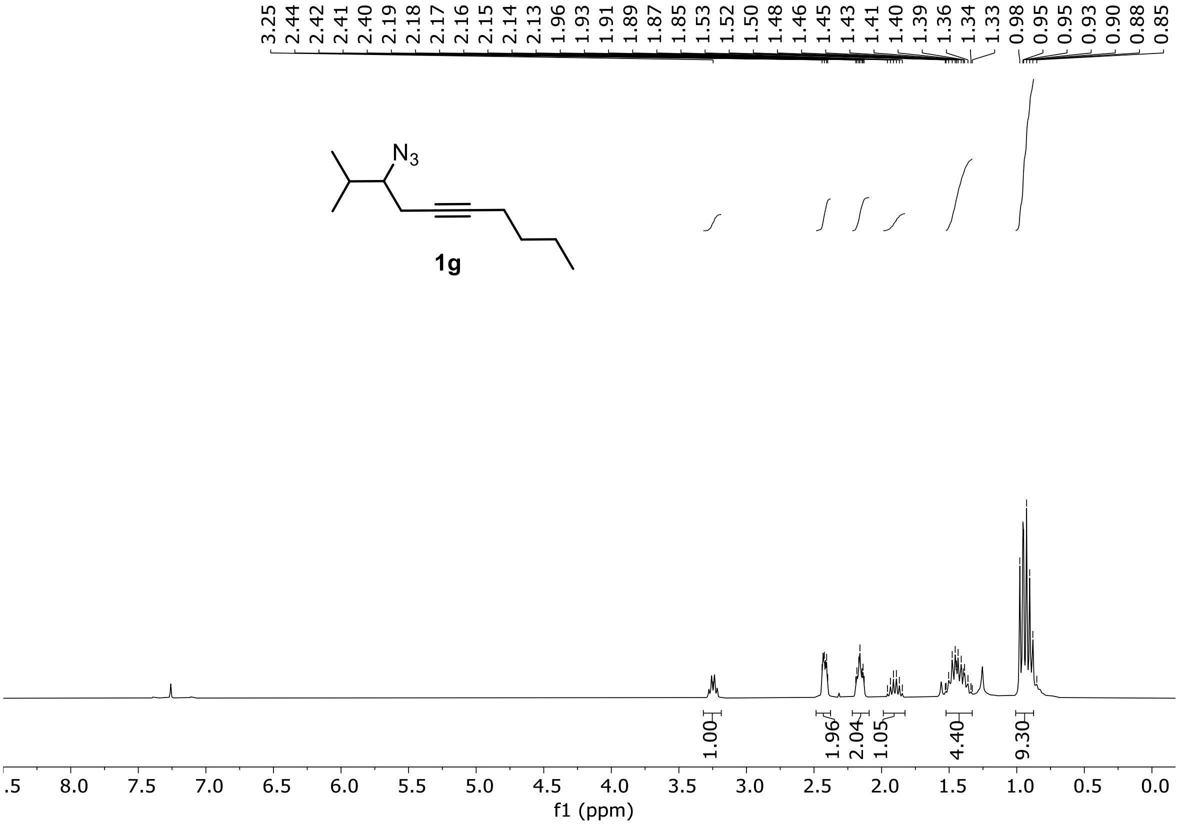

Figure S101. ^{1}H NMR spectrum of **1g** ($CDCl_3$, 300 MHz, 298 K)

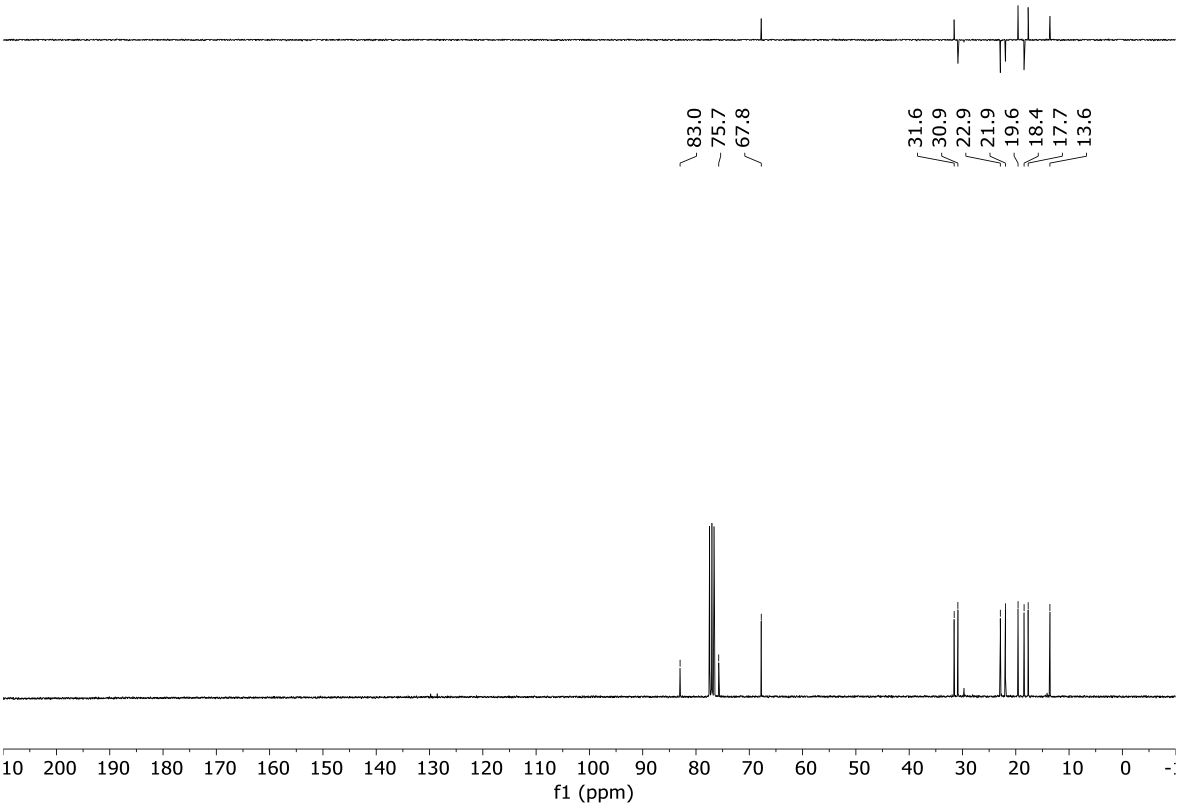

Figure S102. ^{13}C{1H} NMR spectrum of **1g** ($CDCl_3$, 75 MHz, 298 K)

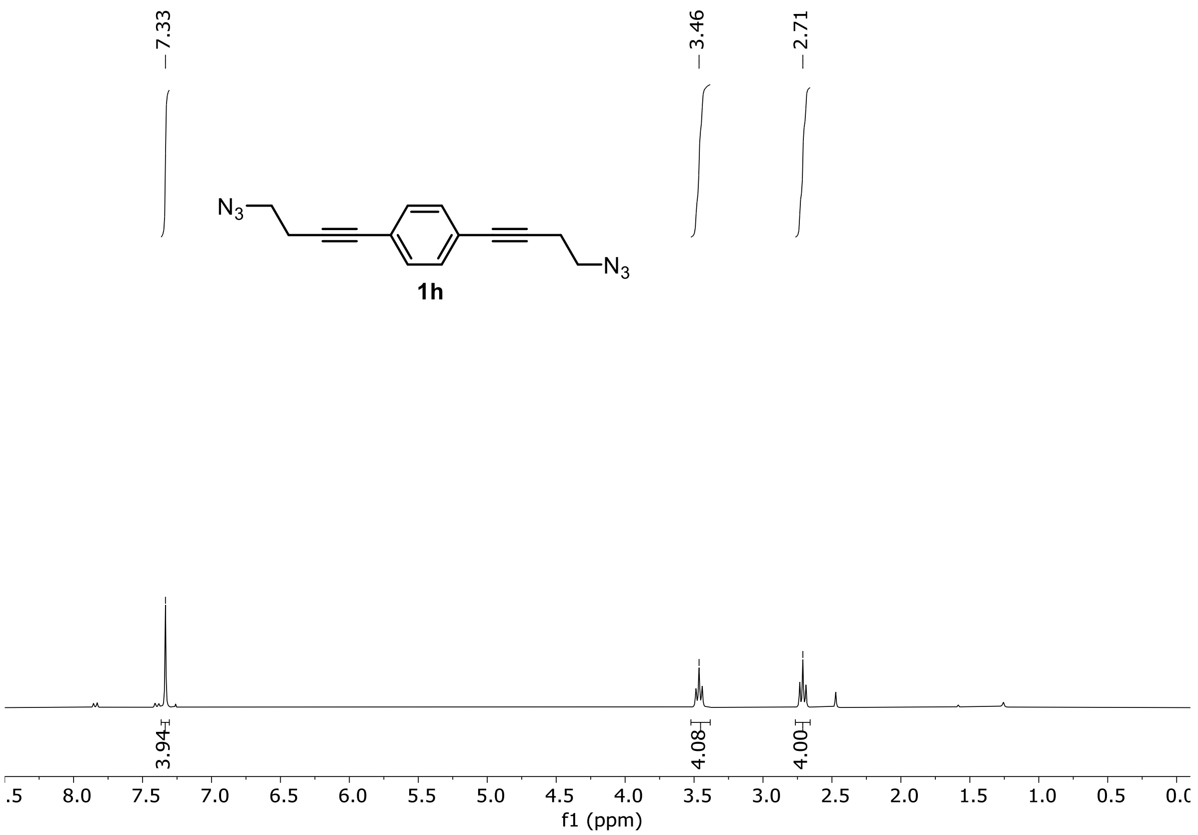

Figure S103. ^{1}H NMR spectrum of **1h** ($CDCl_3$, 300 MHz, 298 K)

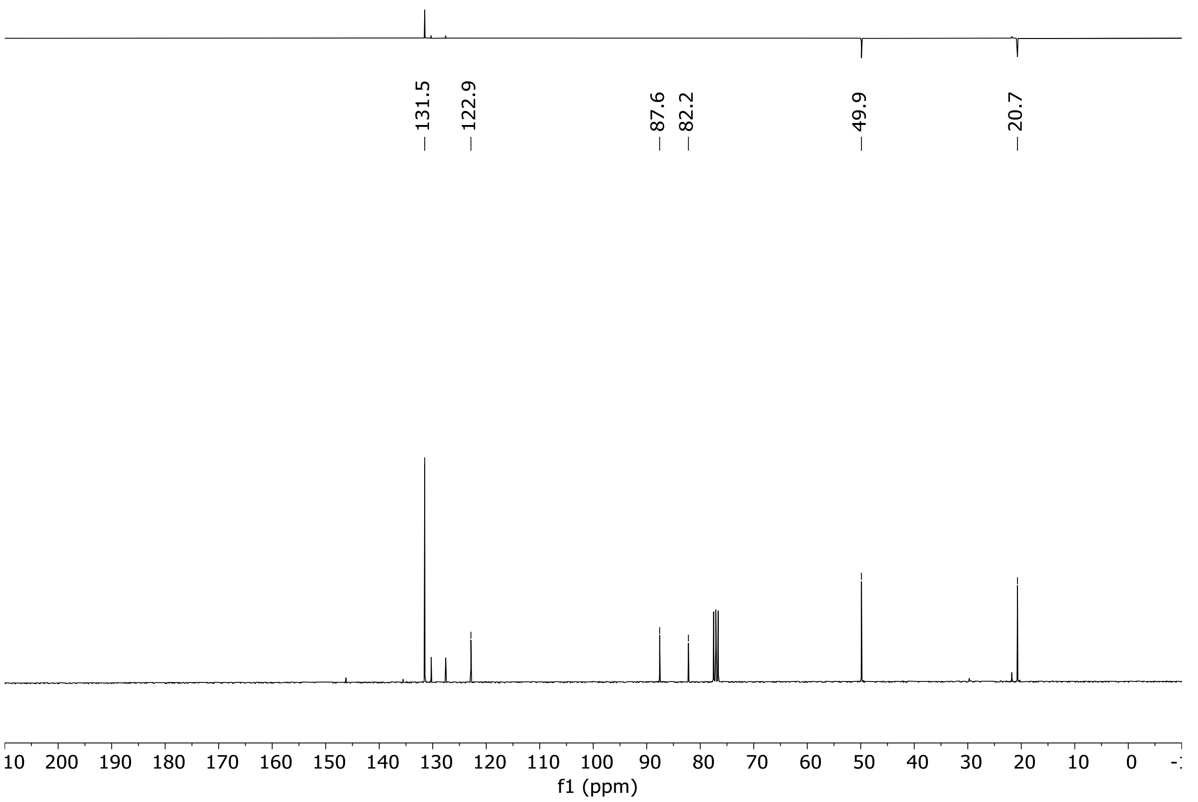

Figure S104. $^{13}C\{1H\}$ NMR spectrum of **1h** ($CDCl_3$, 75 MHz, 298 K)

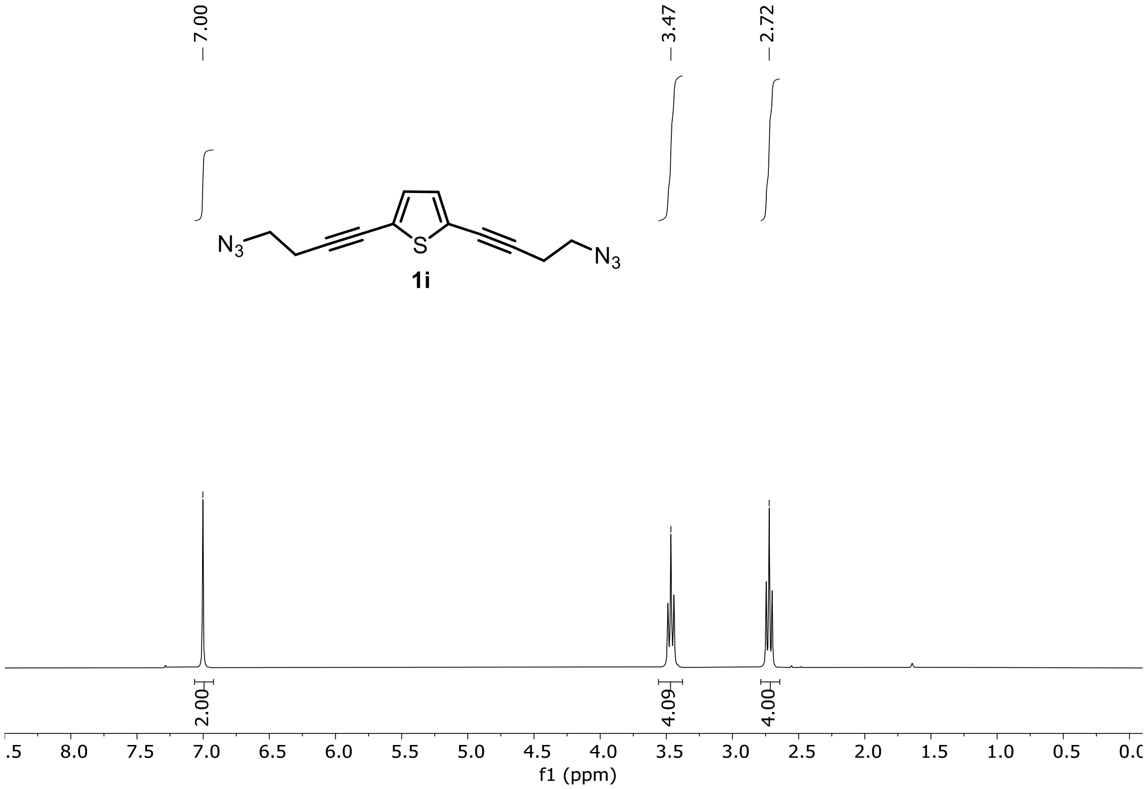

Figure S105. ^{1}H NMR spectrum of **1i** ($CDCl_3$, 300 MHz, 298 K)

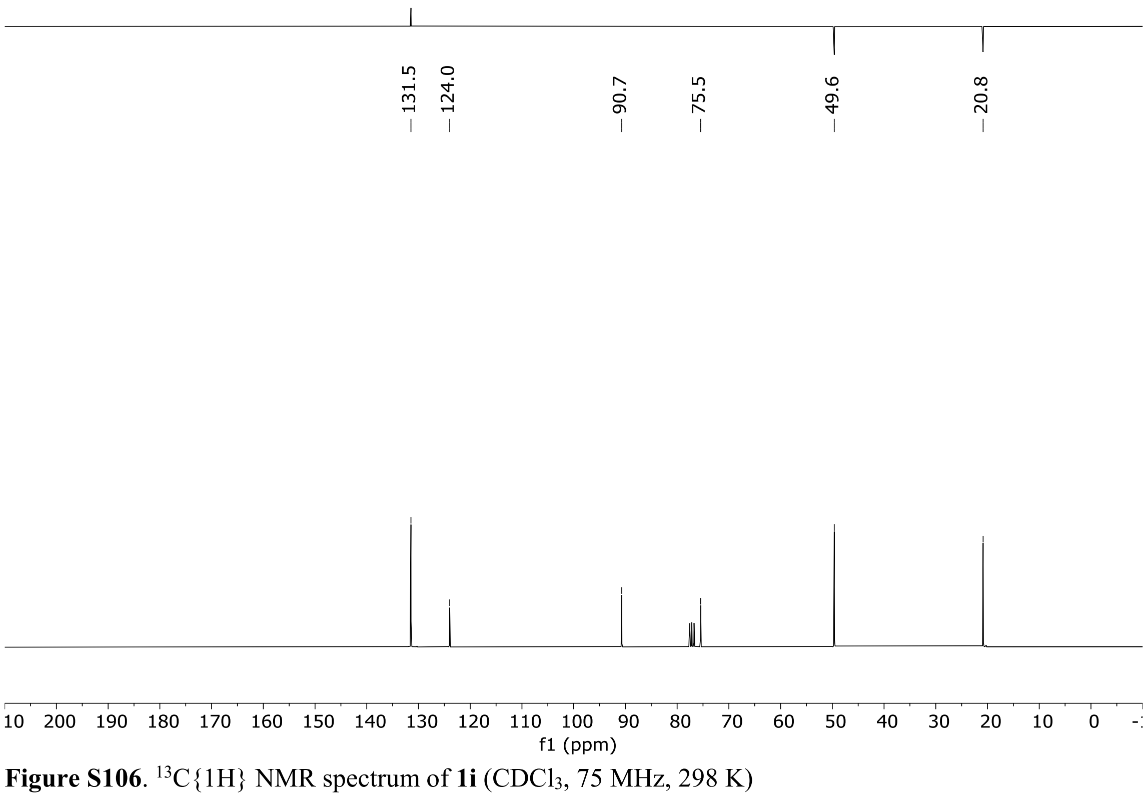

Figure S106. $^{13}C\{1H\}$ NMR spectrum of **1i** ($CDCl_3$, 75 MHz, 298 K)

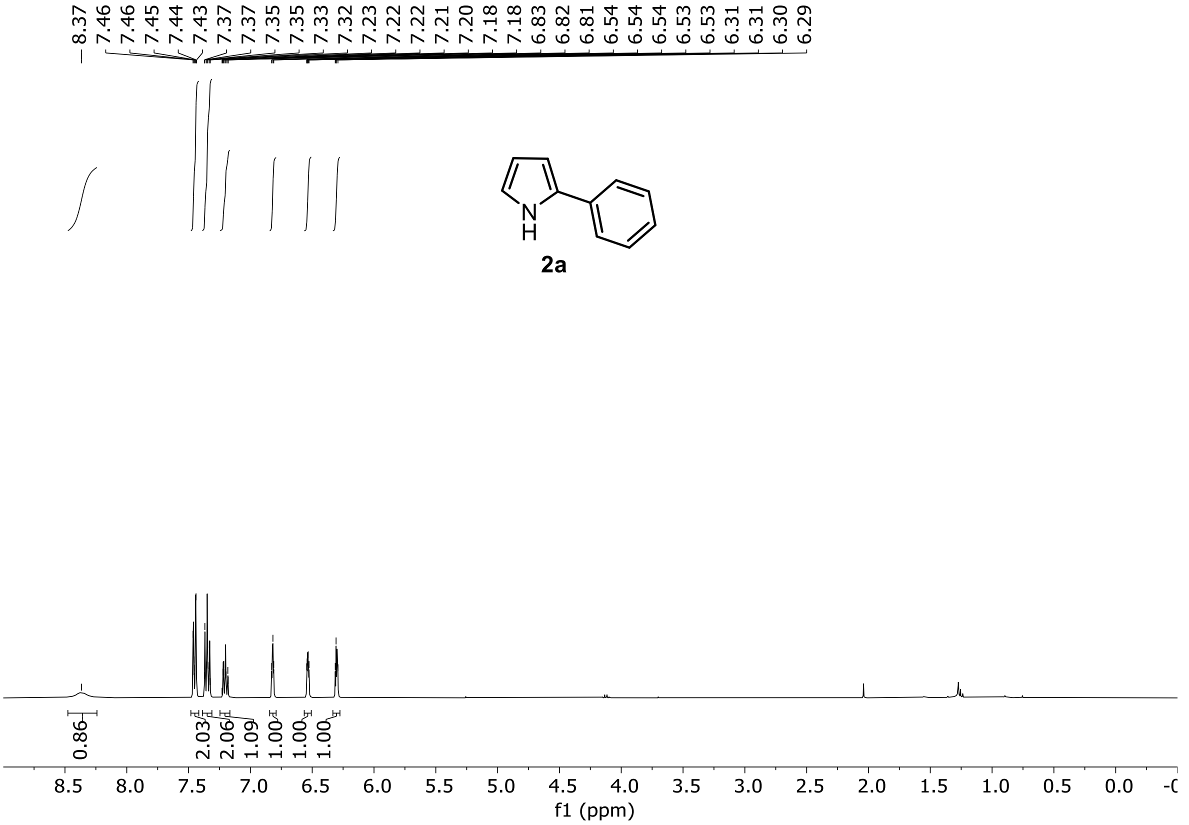

Figure S107. ^{1}H NMR spectrum of **2a** ($CDCl_3$, 300 MHz, 298 K)

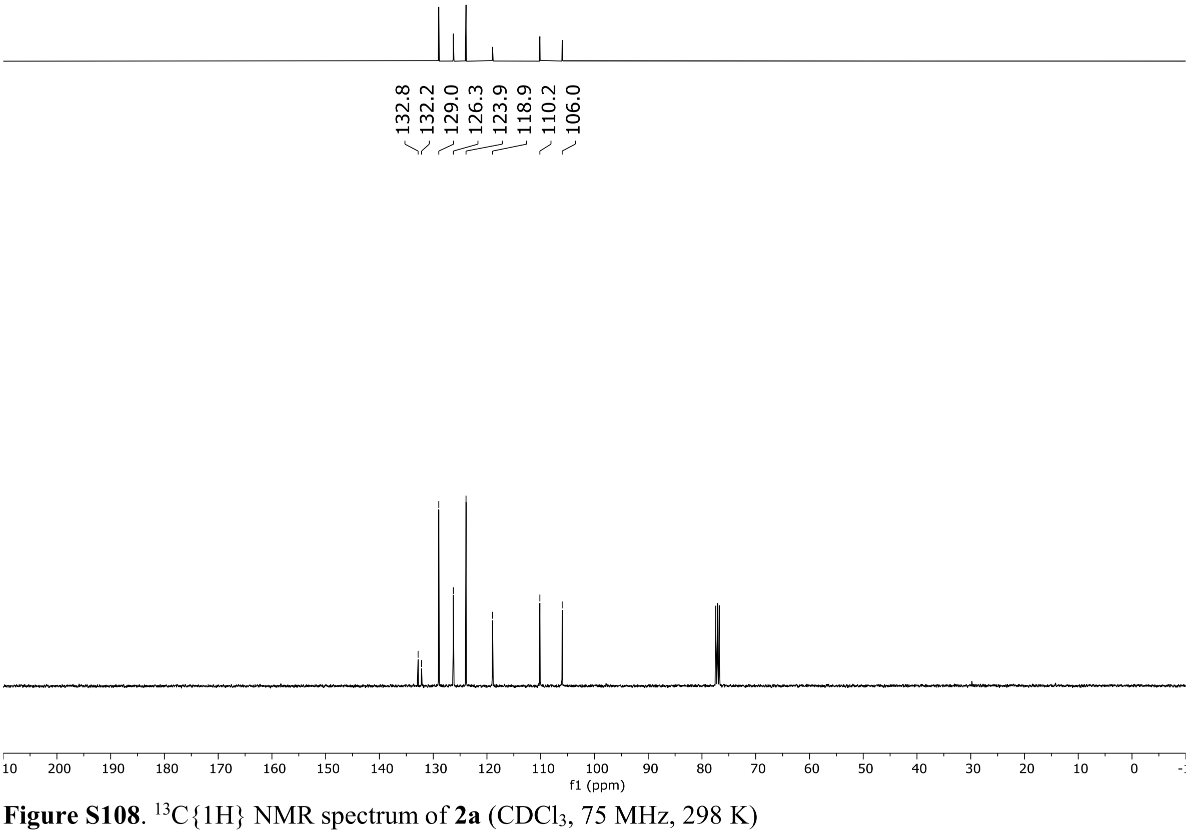

Figure S108. ^{13}C{1H} NMR spectrum of **2a** ($CDCl_3$, 75 MHz, 298 K)

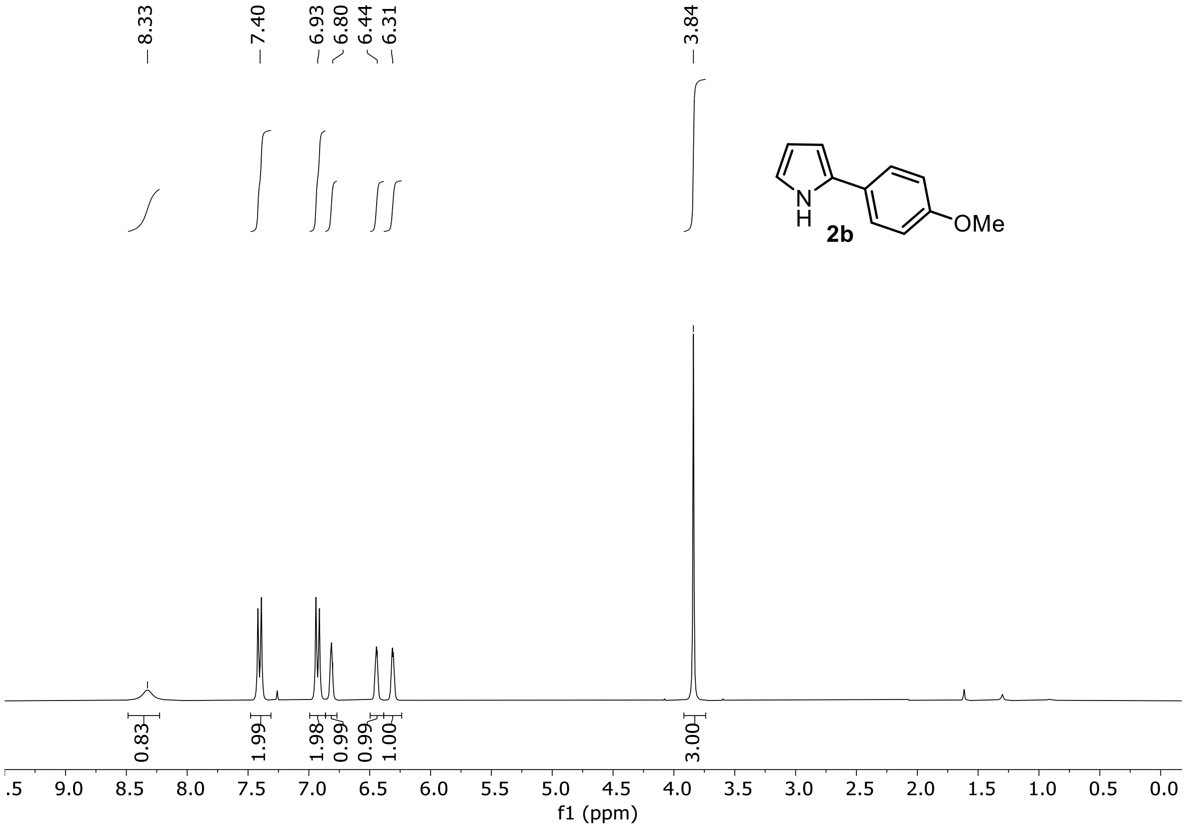

Figure S109. ^{1}H NMR spectrum of **2b** ($CDCl_3$, 300 MHz, 298 K)

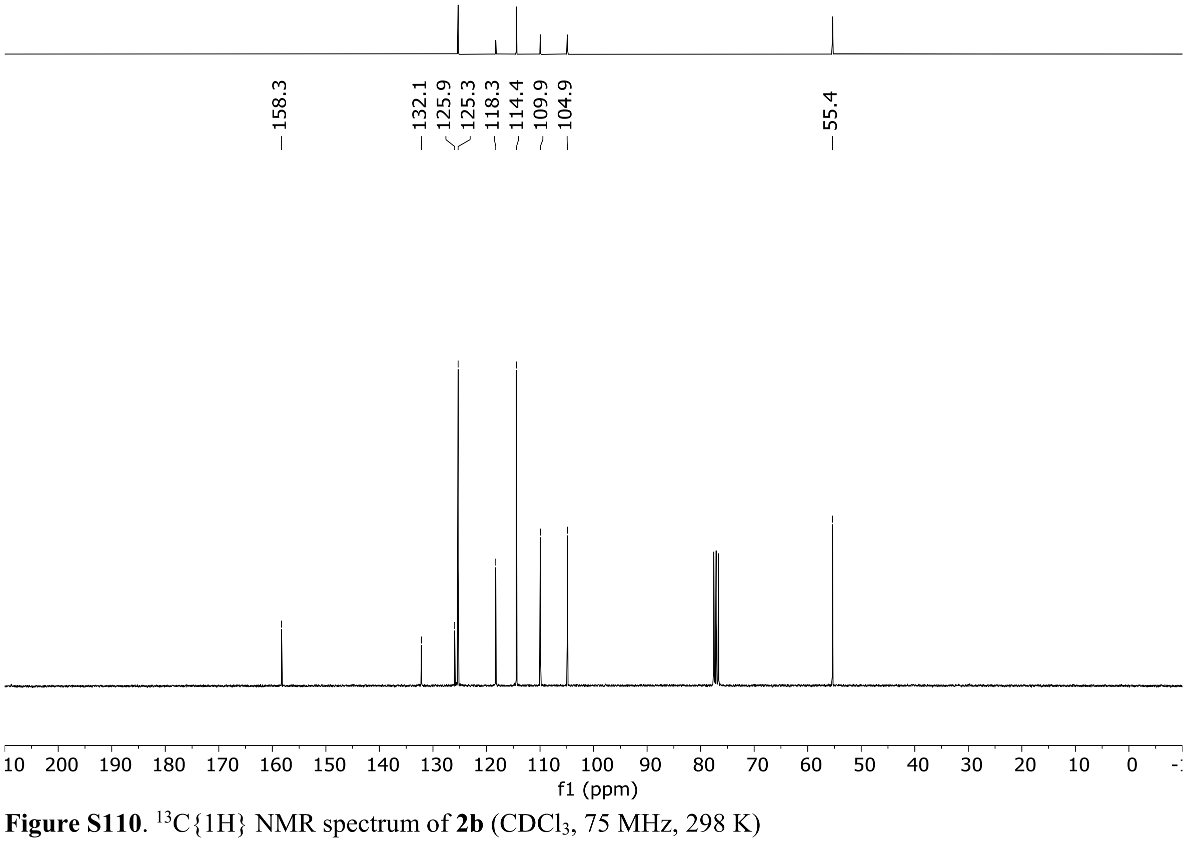

Figure S110. $^{13}C\{1H\}$ NMR spectrum of **2b** ($CDCl_3$, 75 MHz, 298 K)

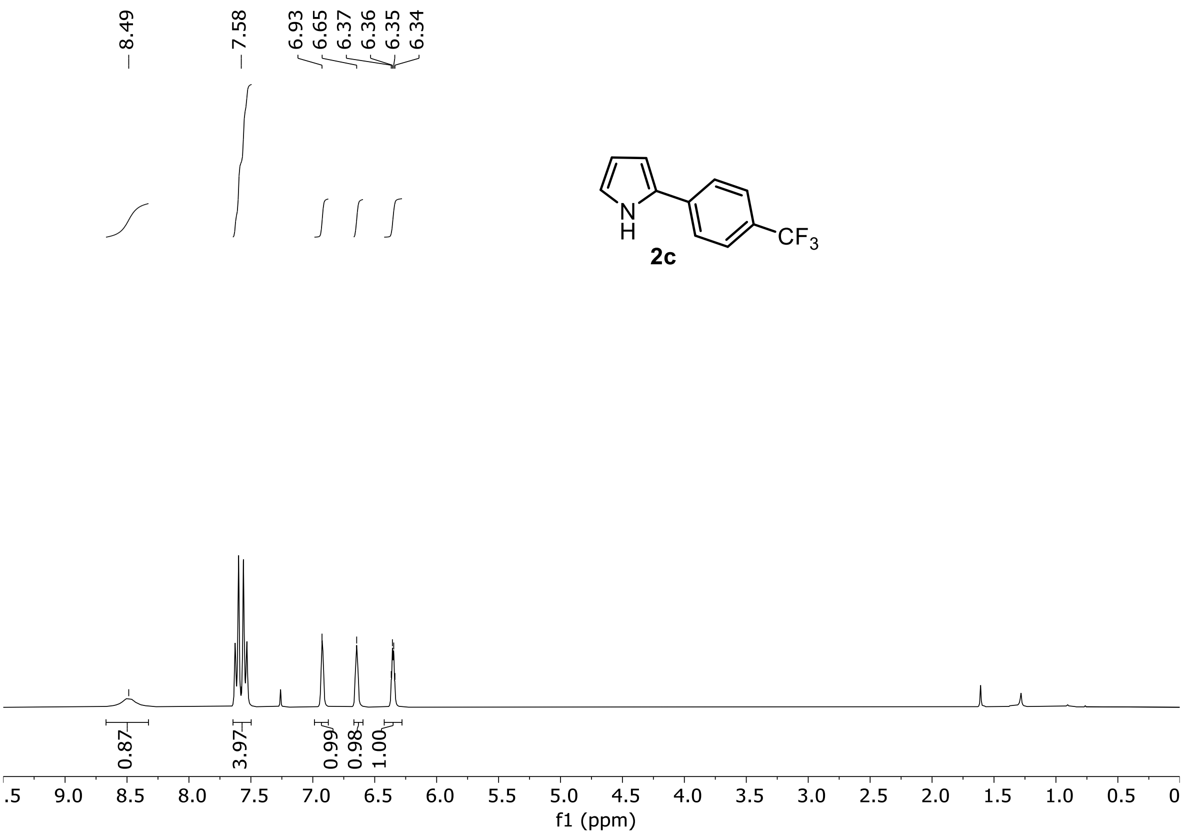

Figure S111. ^{1}H NMR spectrum of **2c** ($CDCl_3$, 300 MHz, 298 K)

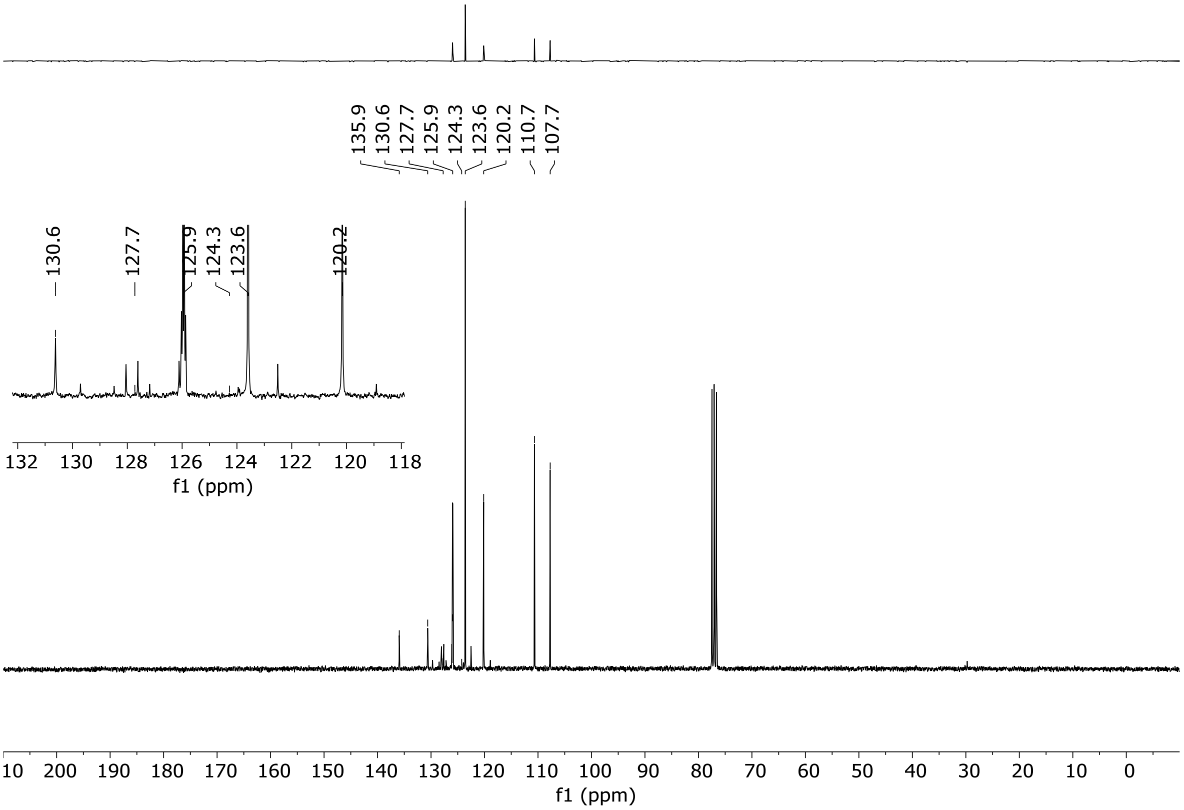

Figure S112. $^{13}C\{1H\}$ NMR spectrum of **2c** ($CDCl_3$, 75 MHz, 298 K)

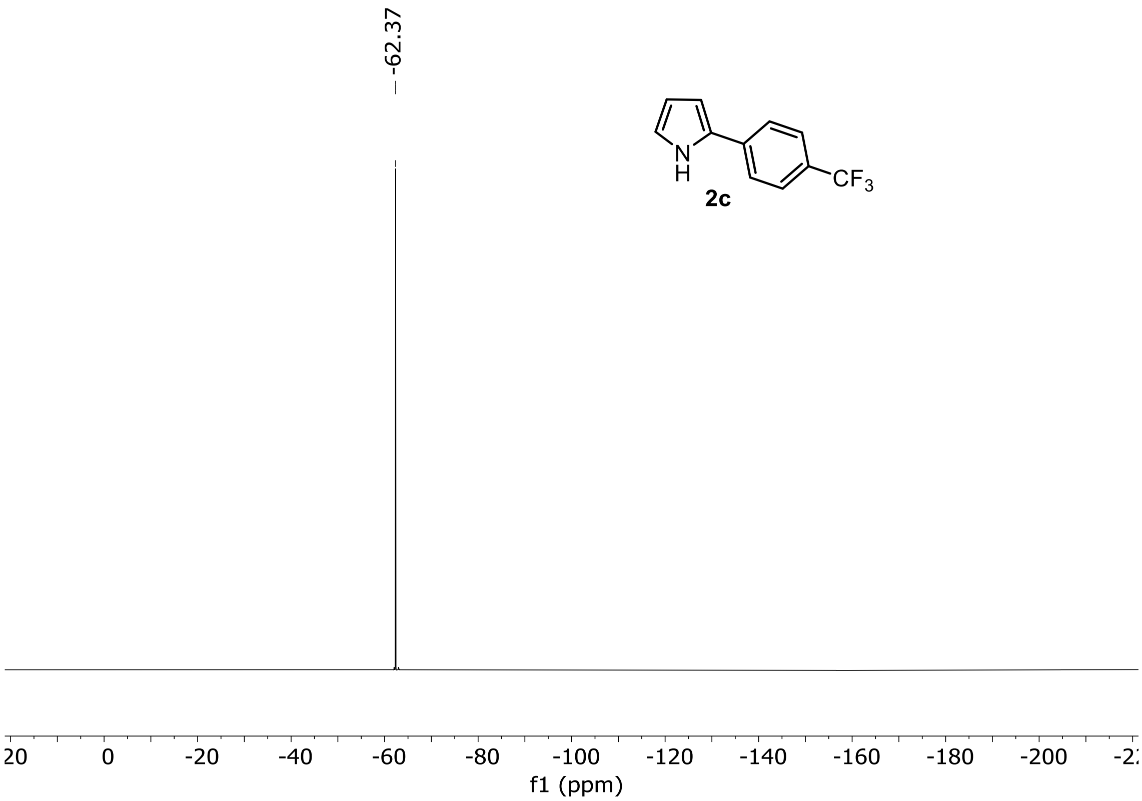

Figure S113. ^{19}F NMR spectrum of **2c** ($CDCl_3$, 282 MHz, 298 K)

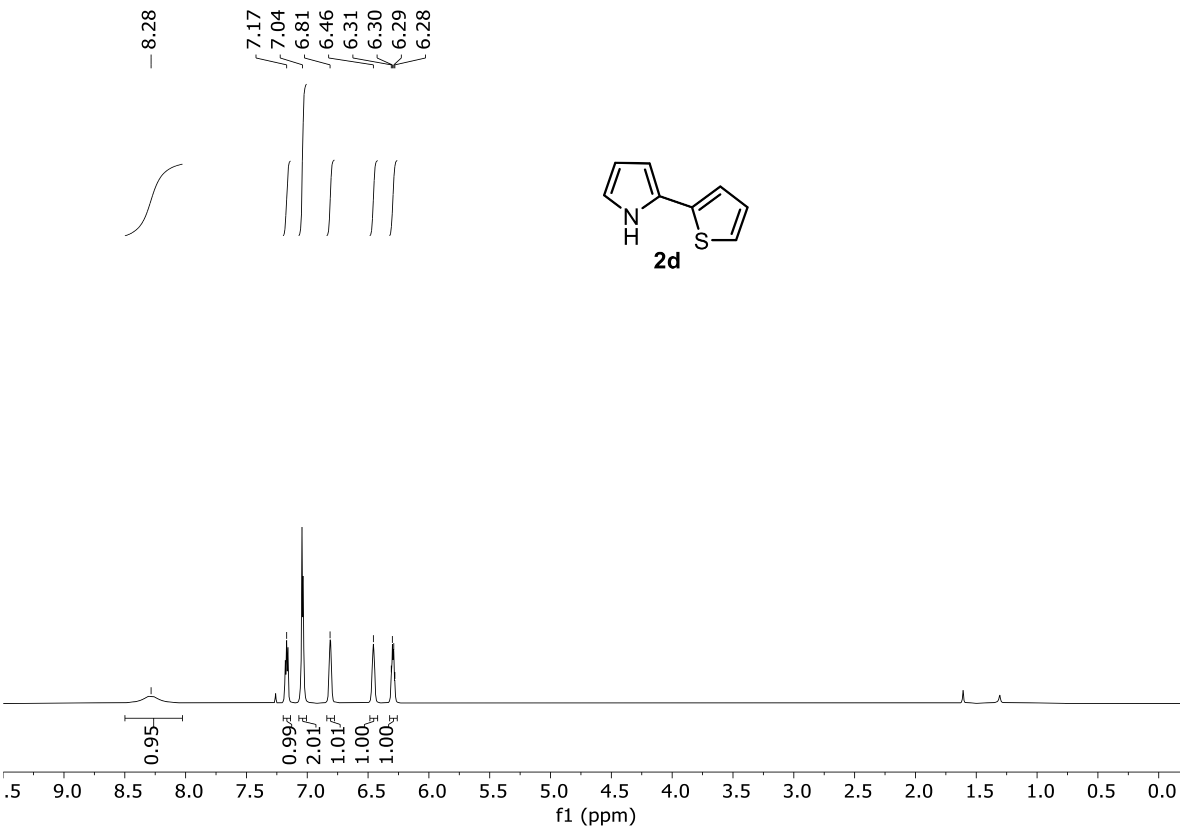

Figure S114. ^{1}H NMR spectrum of **2d** ($CDCl_3$, 300 MHz, 298 K)

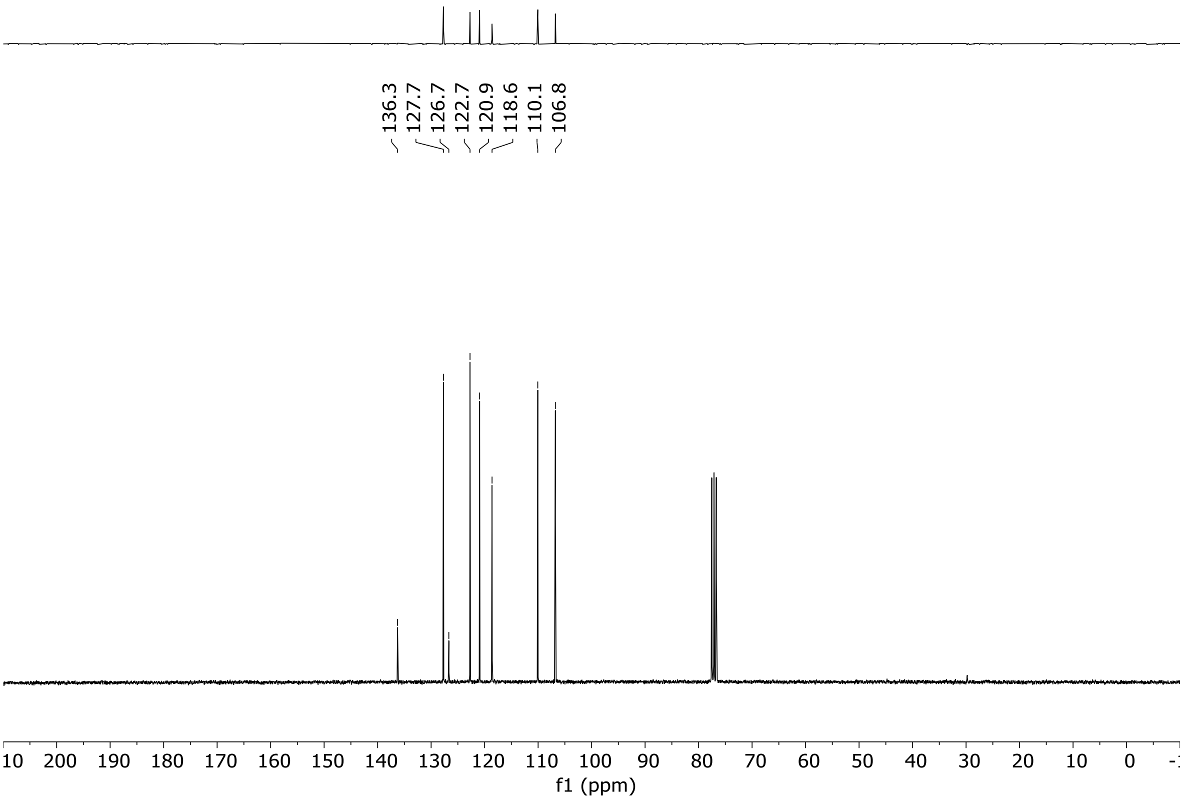

Figure S115. ^{13}C{1H} NMR spectrum of **2d** ($CDCl_3$, 75 MHz, 298 K)

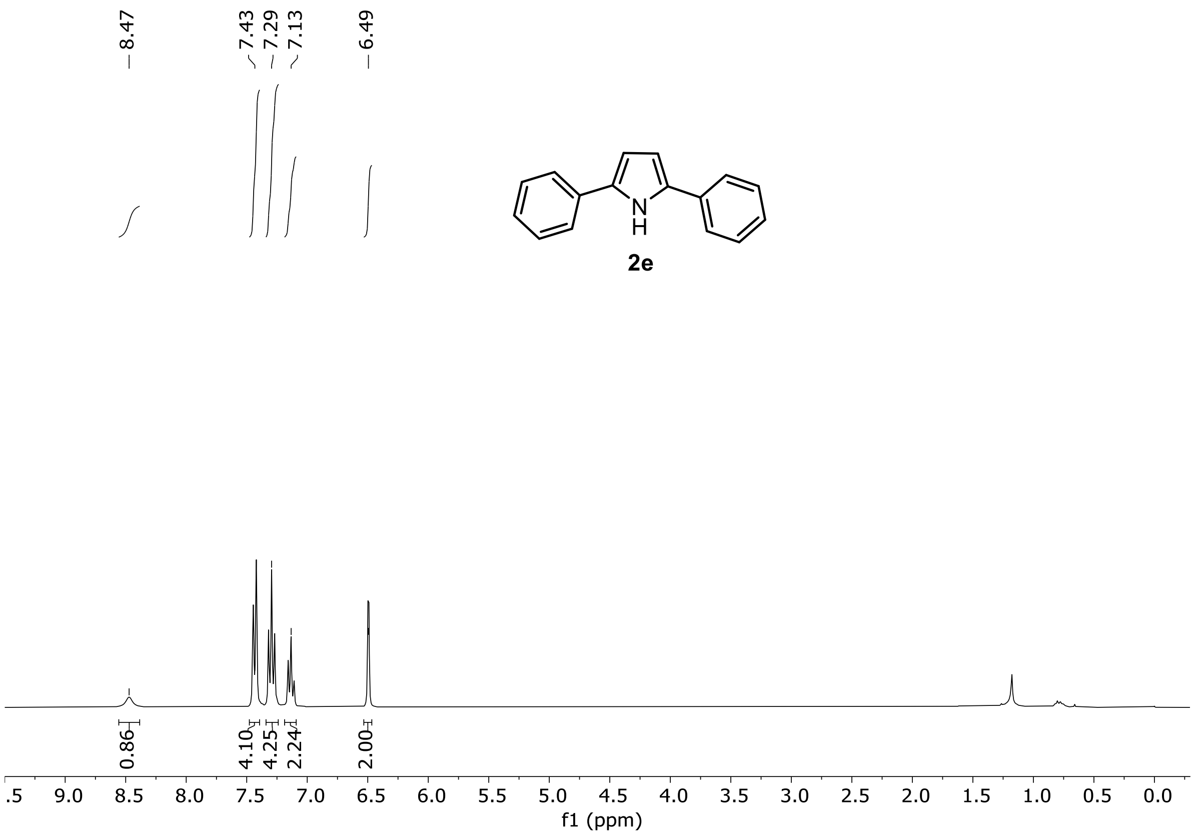

Figure S116. ^{1}H NMR spectrum of **2e** ($CDCl_3$, 300 MHz, 298 K)

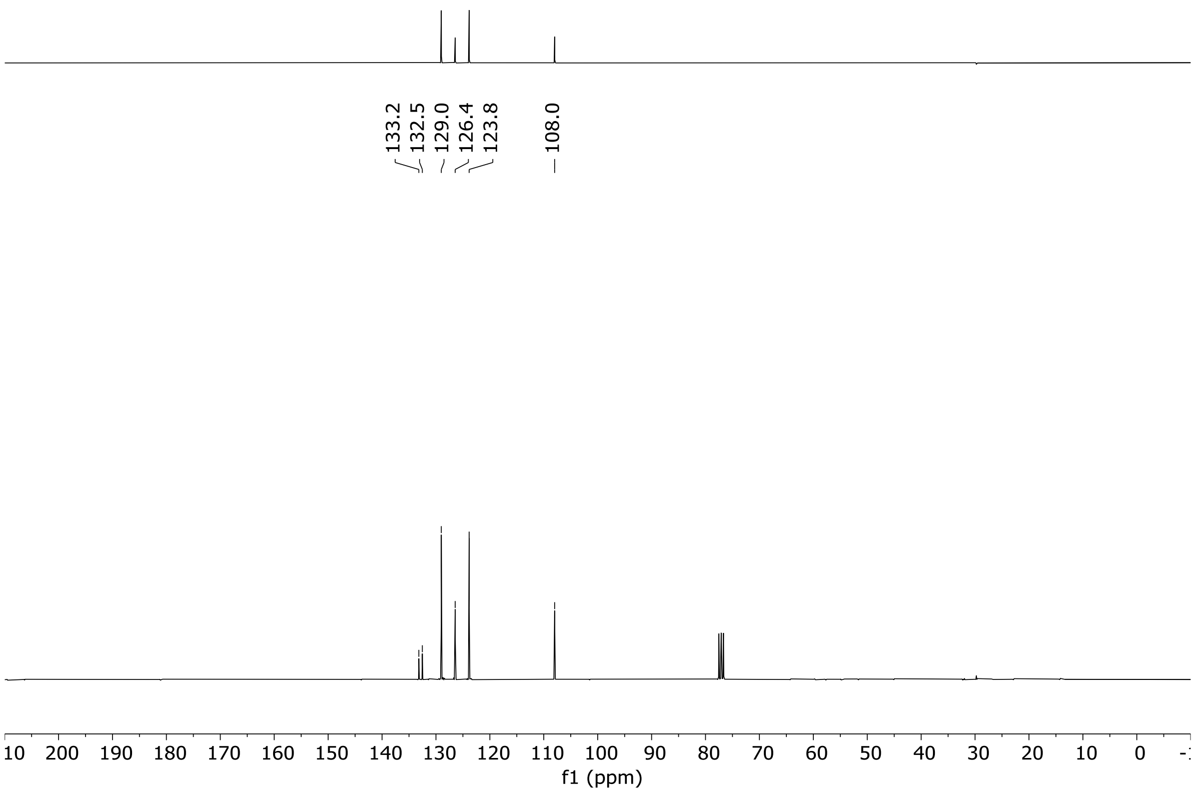

Figure S117. ^{13}C{1H} NMR spectrum of **2e** ($CDCl_3$, 75 MHz, 298 K)

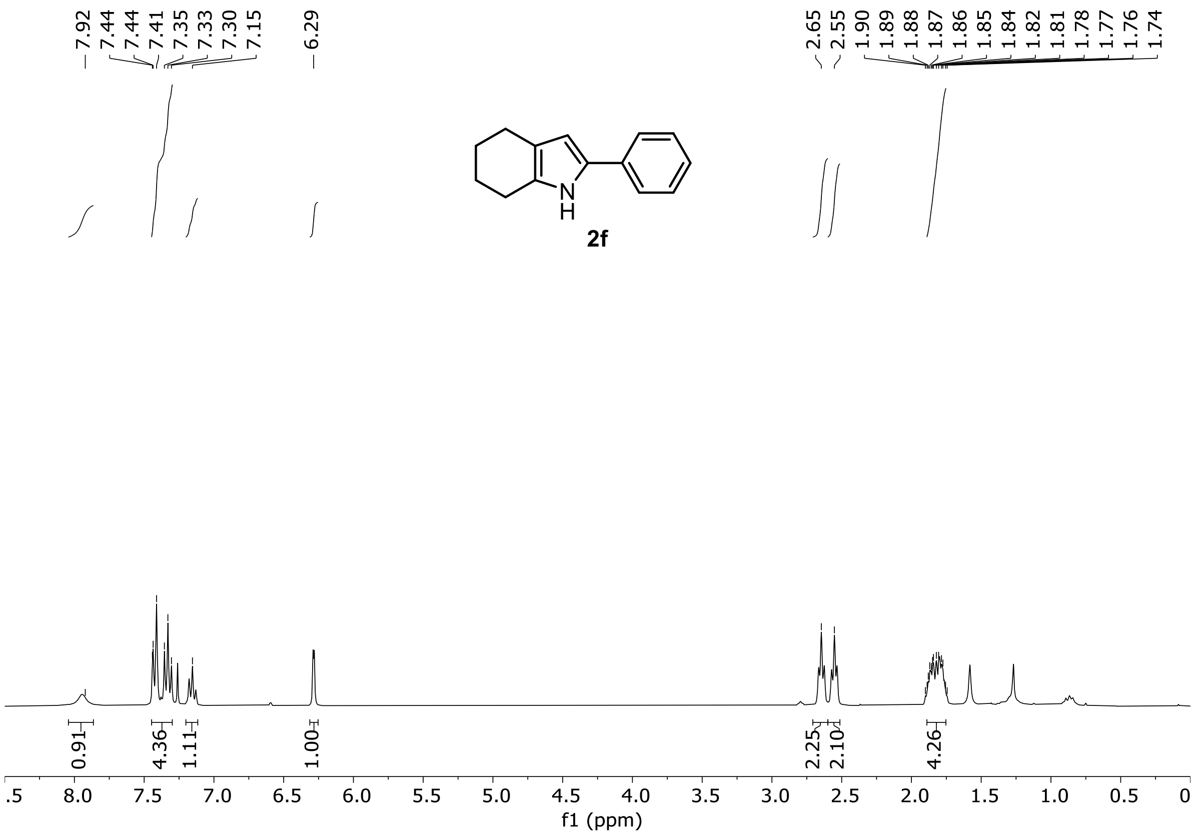

Figure S118. ^{1}H NMR spectrum of **2f** ($CDCl_3$, 300 MHz, 298 K)

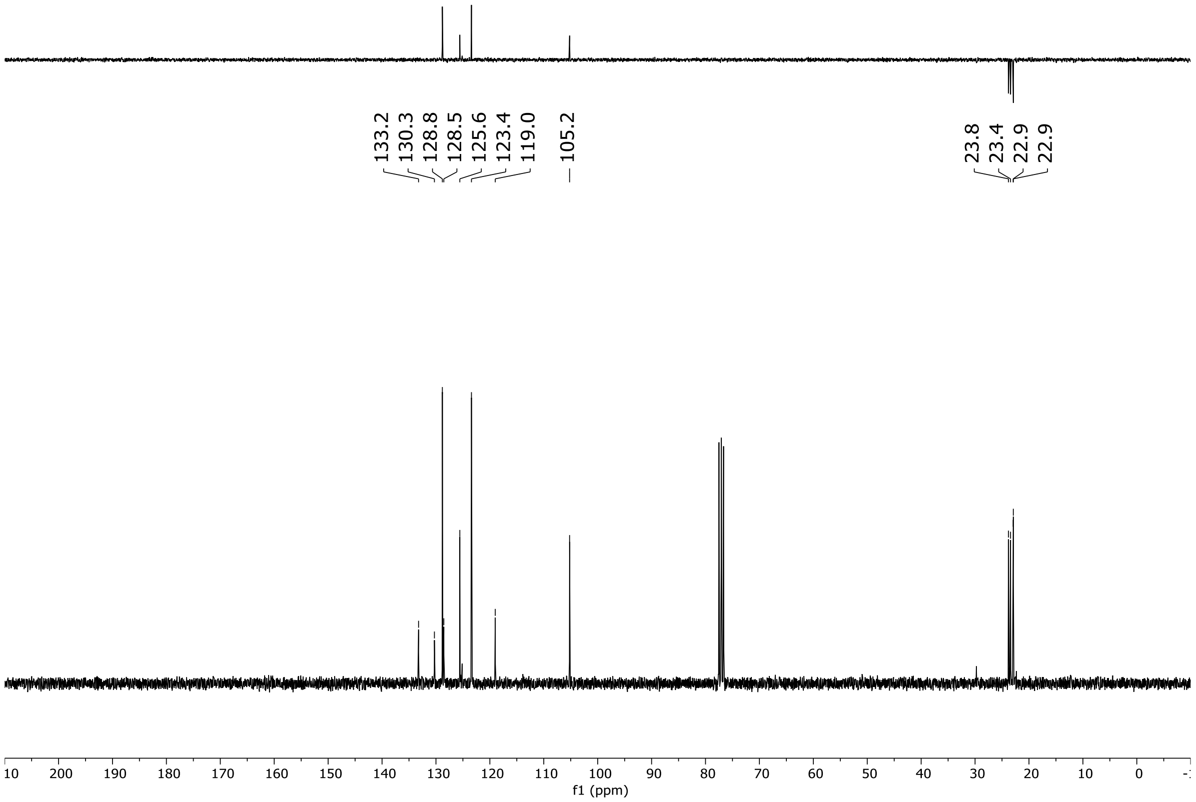

Figure S119. $^{13}C\{1H\}$ NMR spectrum of **2f** ($CDCl_3$, 75 MHz, 298 K)

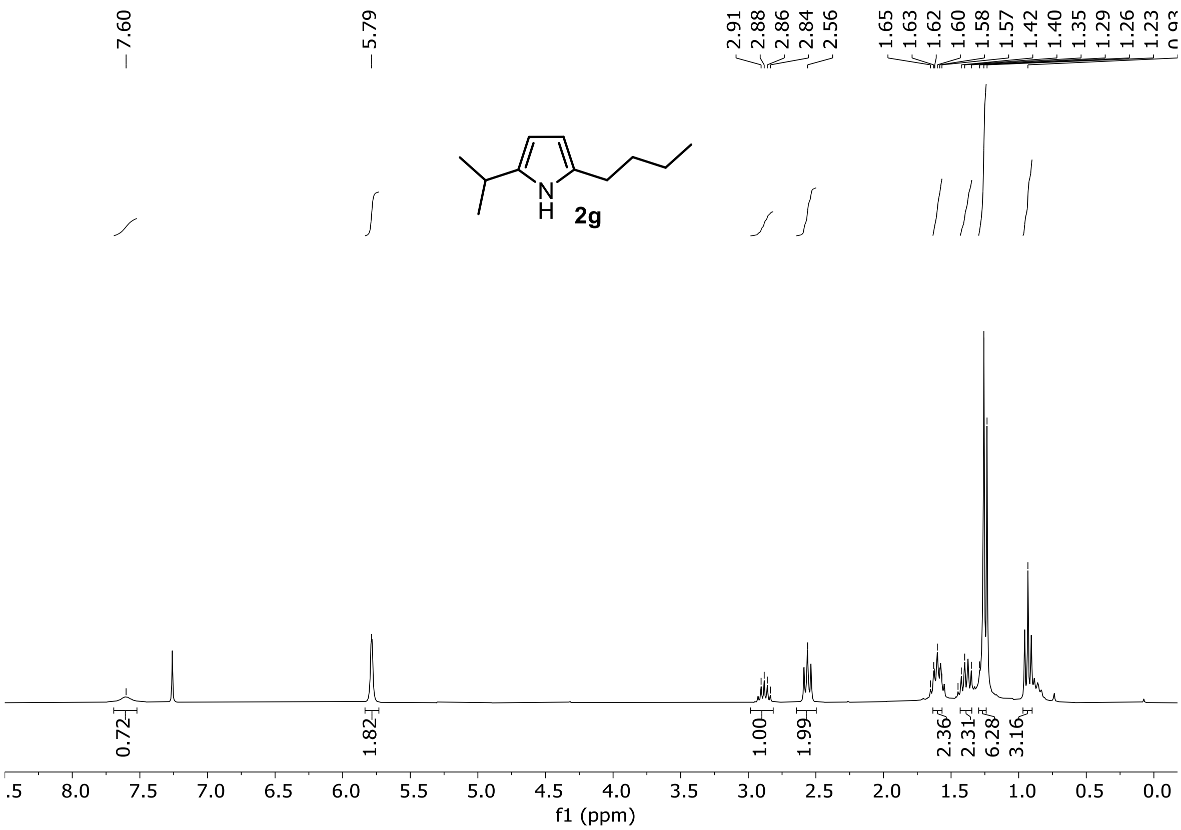

Figure S120. ^{1}H NMR spectrum of **2g** ($CDCl_3$, 300 MHz, 298 K)

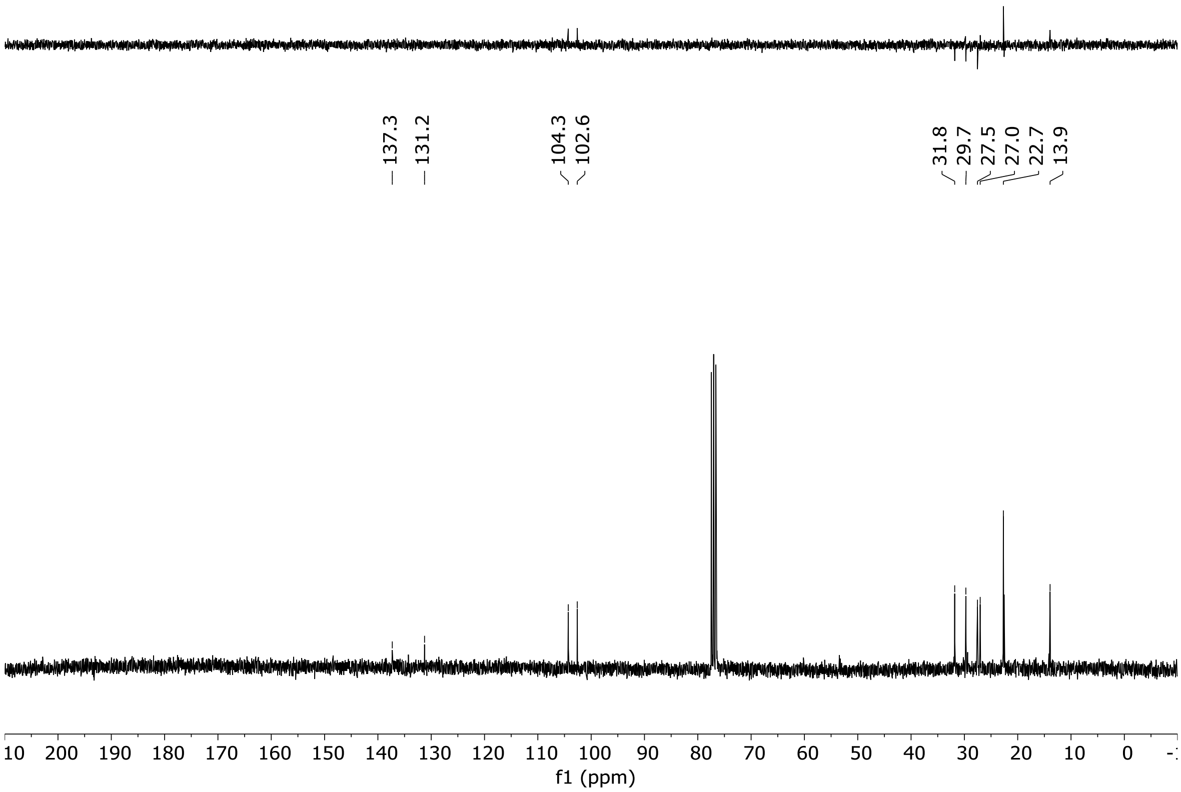

Figure S121. $^{13}C\{1H\}$ NMR spectrum of **2g** ($CDCl_3$, 75 MHz, 298 K)

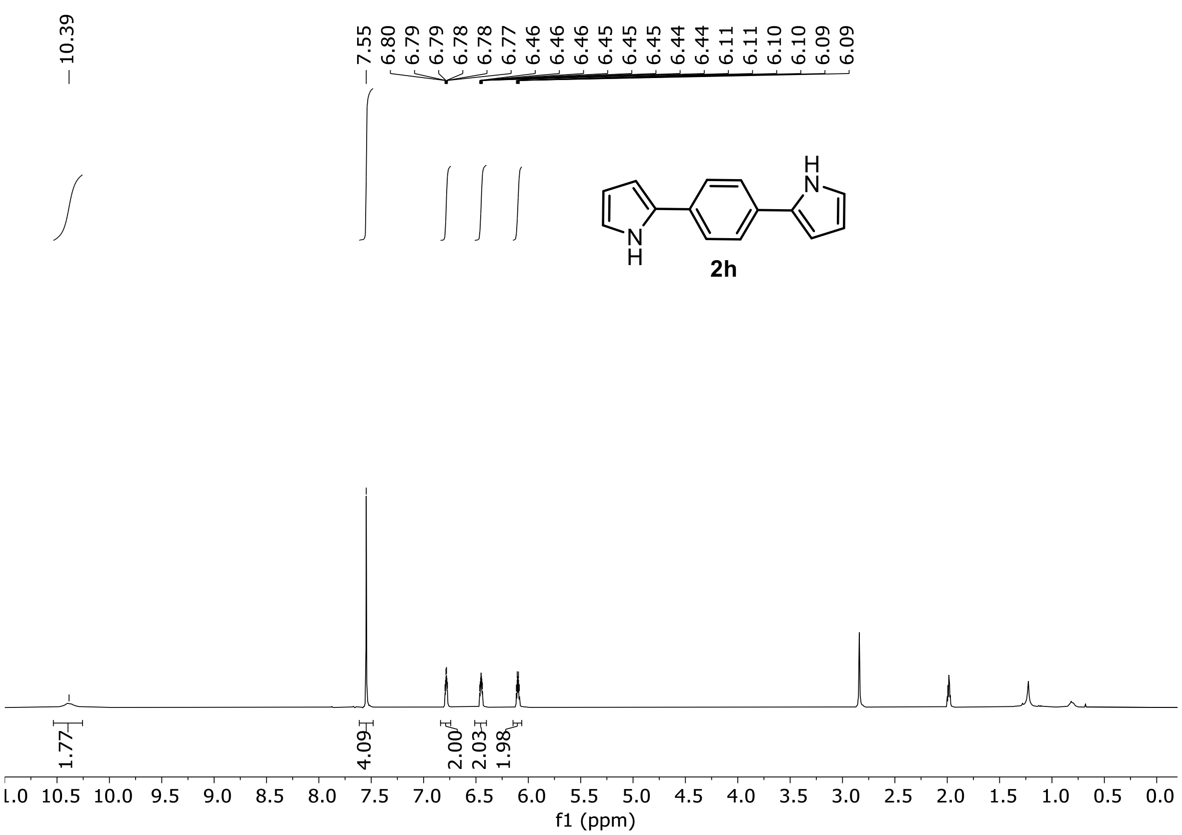

Figure S122. ^{1}H NMR spectrum of **2h** (Acetone-d_6, 300 MHz, 298 K)

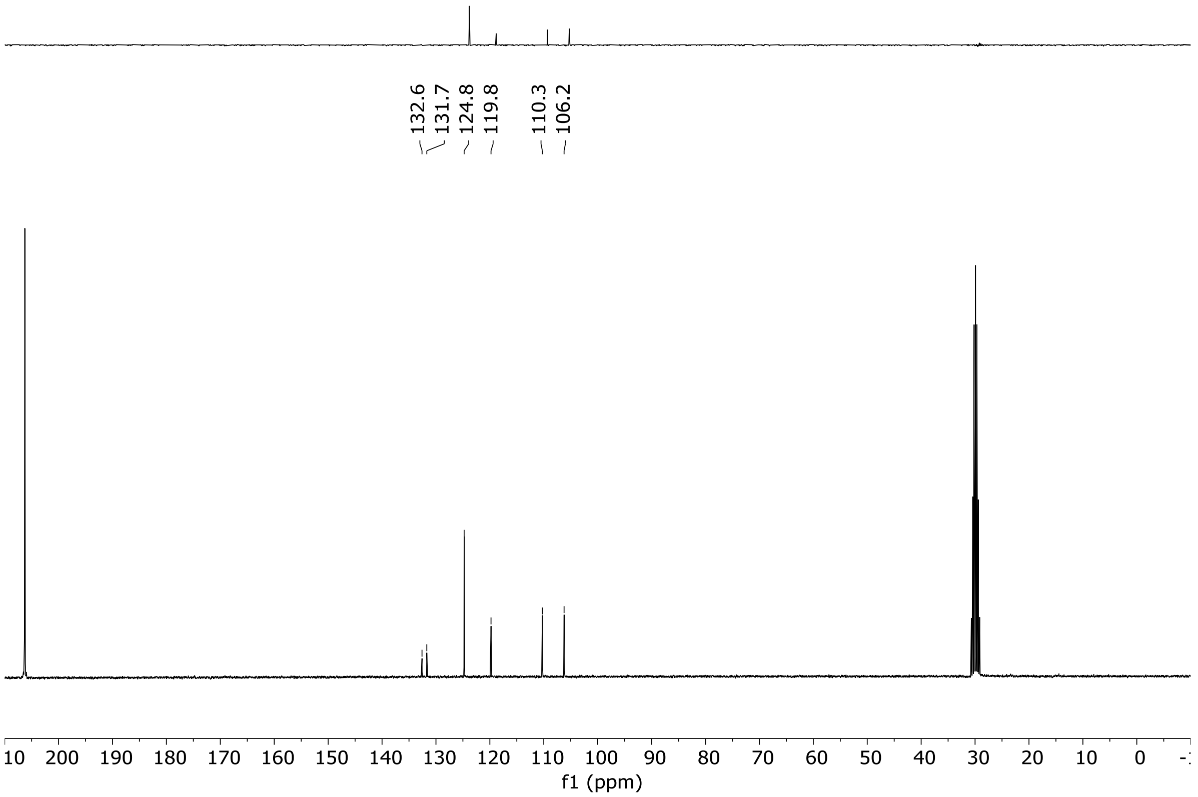

Figure S123. $^{13}C\{1H\}$ NMR spectrum of **2h** (Acetone-d_6, 75 MHz, 298 K)

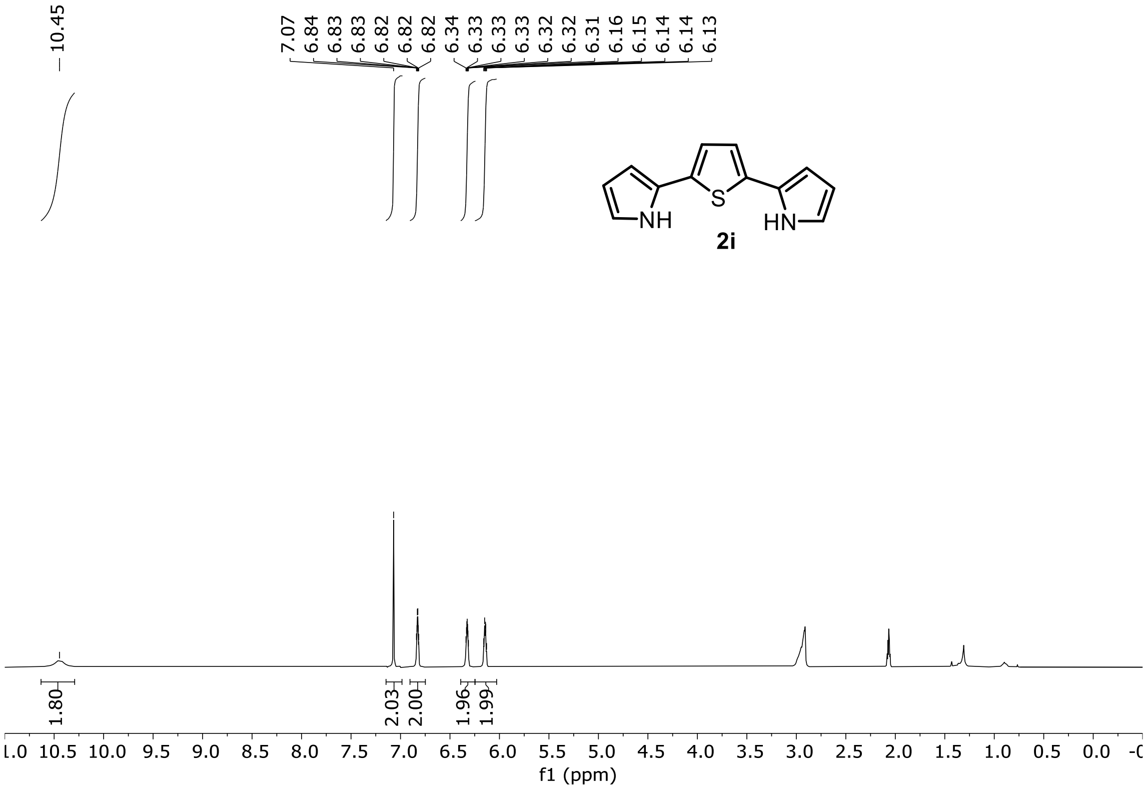

Figure S124. ^{1}H NMR spectrum of **2i** (Acetone-d_6, 300 MHz, 298 K)

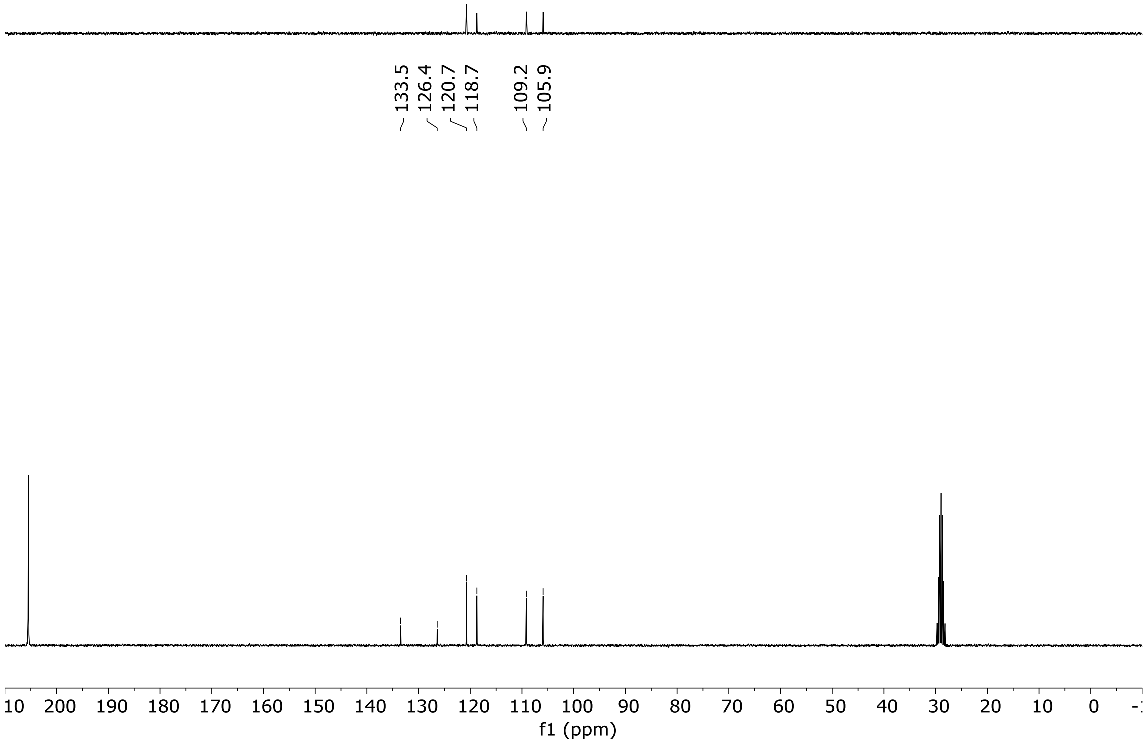

Figure S125. $^{13}C\{1H\}$ NMR spectrum of **2i** (Acetone-d_6, 75 MHz, 298 K)

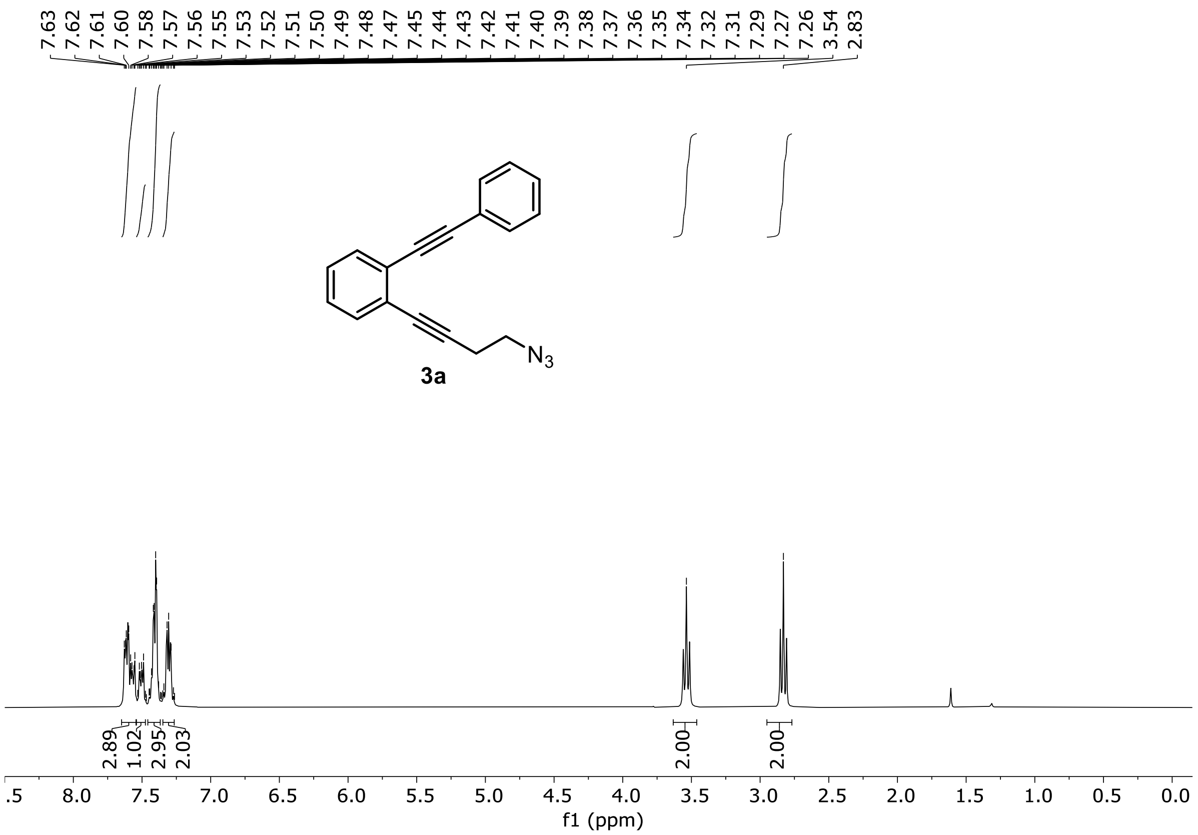

Figure S126. ^{1}H NMR spectrum of **3a** ($CDCl_3$, 300 MHz, 298 K)

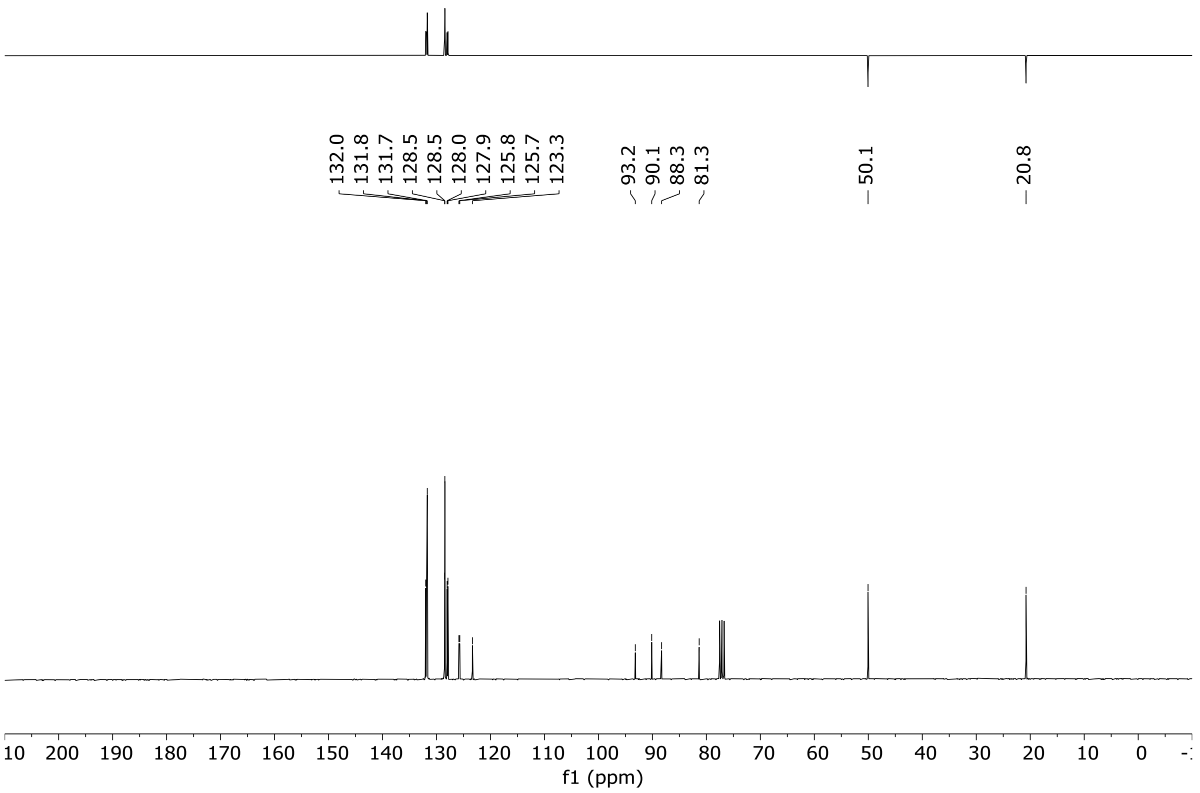

Figure S127. $^{13}C\{1H\}$ NMR spectrum of **3a** ($CDCl_3$, 75 MHz, 298 K)

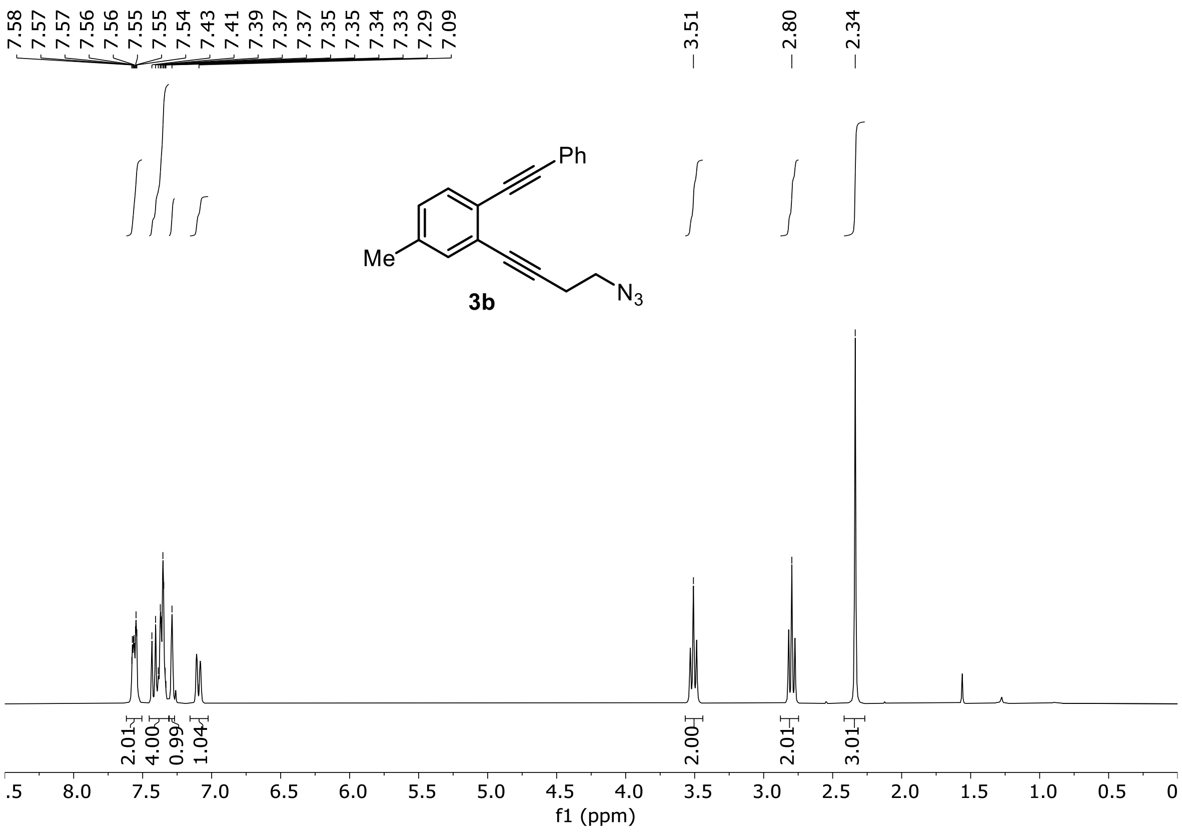

Figure S128. ^{1}H NMR spectrum of **3b** ($CDCl_3$, 300 MHz, 298 K)

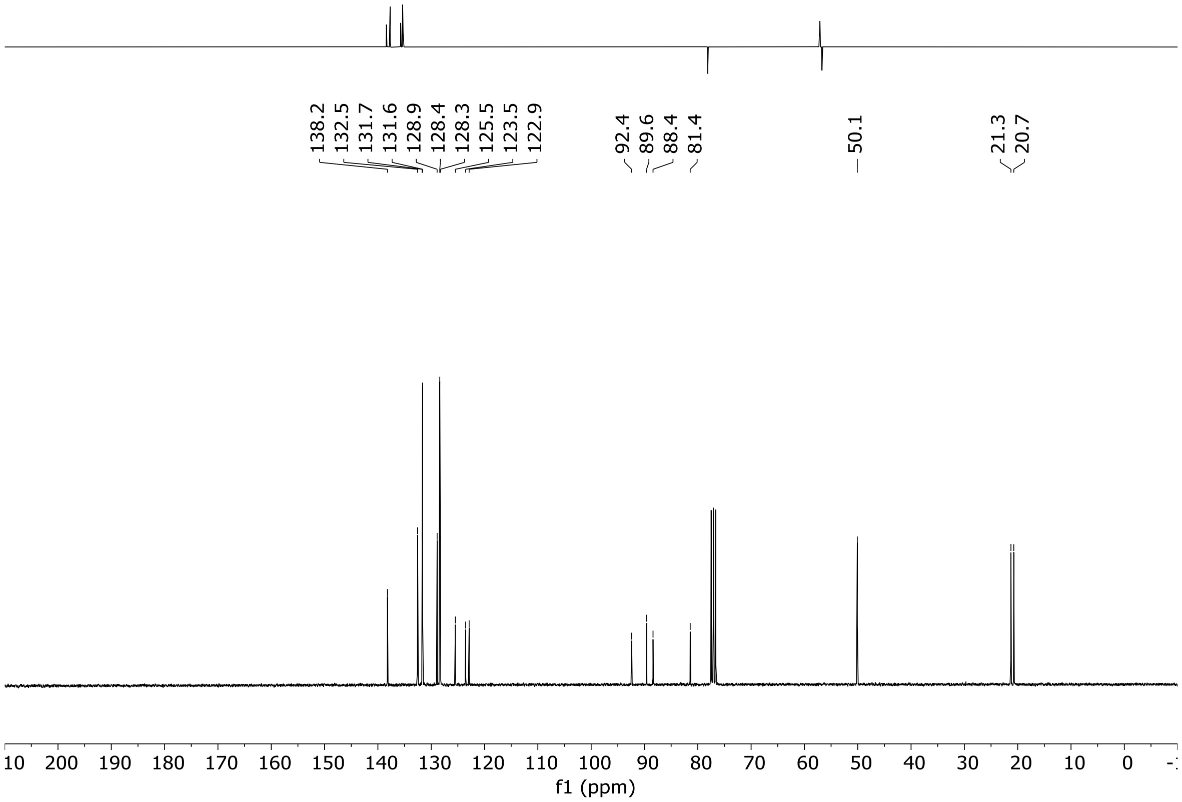

Figure S129. $^{13}C\{1H\}$ NMR spectrum of **3b** ($CDCl_3$, 75 MHz, 298 K)

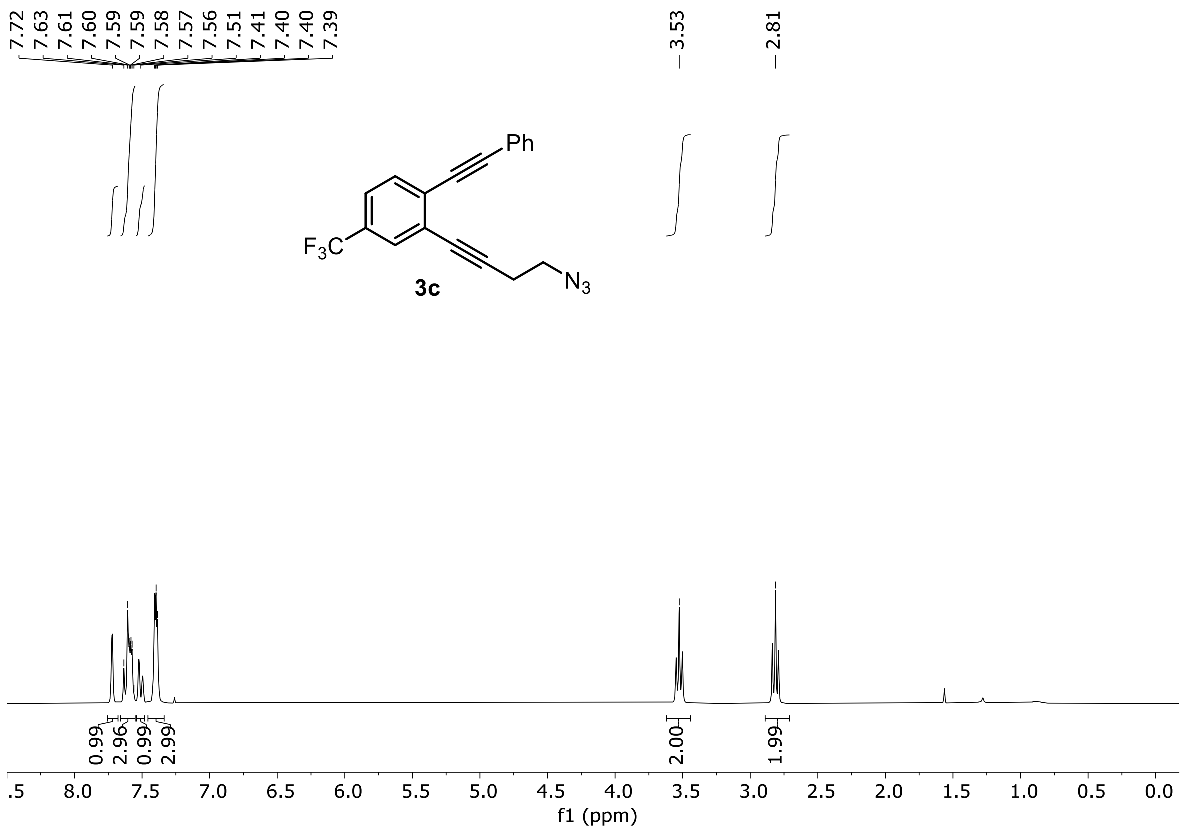

Figure S130. ^{1}H NMR spectrum of **3c** ($CDCl_3$, 300 MHz, 298 K)

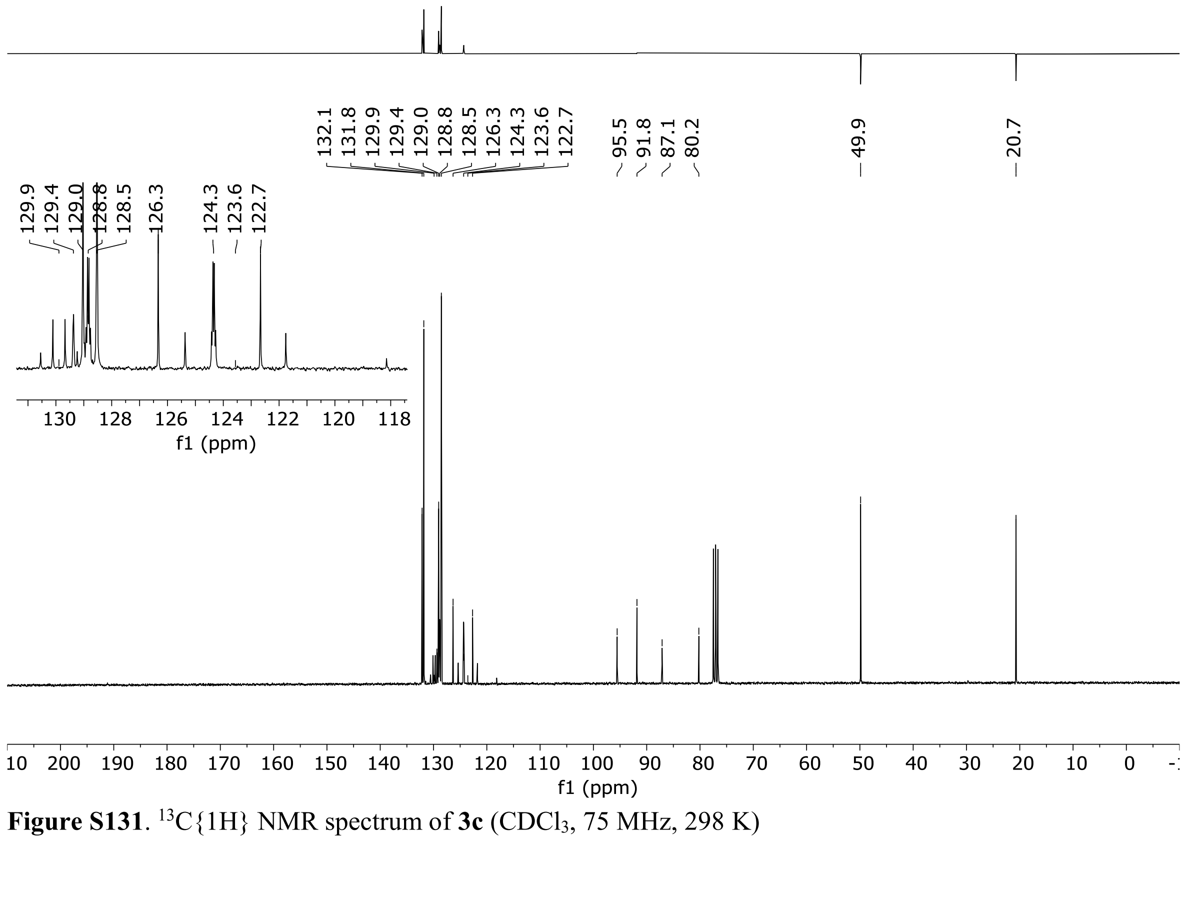

Figure S131. $^{13}C\{1H\}$ NMR spectrum of **3c** ($CDCl_3$, 75 MHz, 298 K)

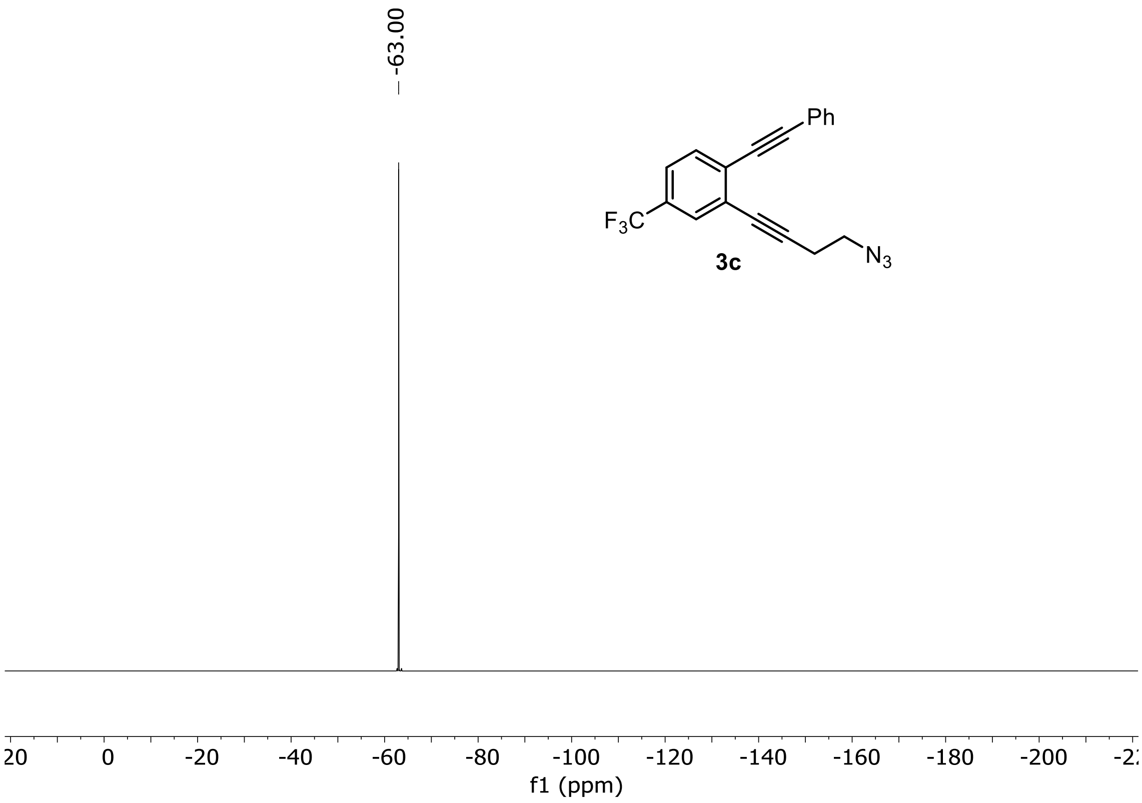

Figure S132. ^{19}F NMR spectrum of **3c** ($CDCl_3$, 282 MHz, 298 K)

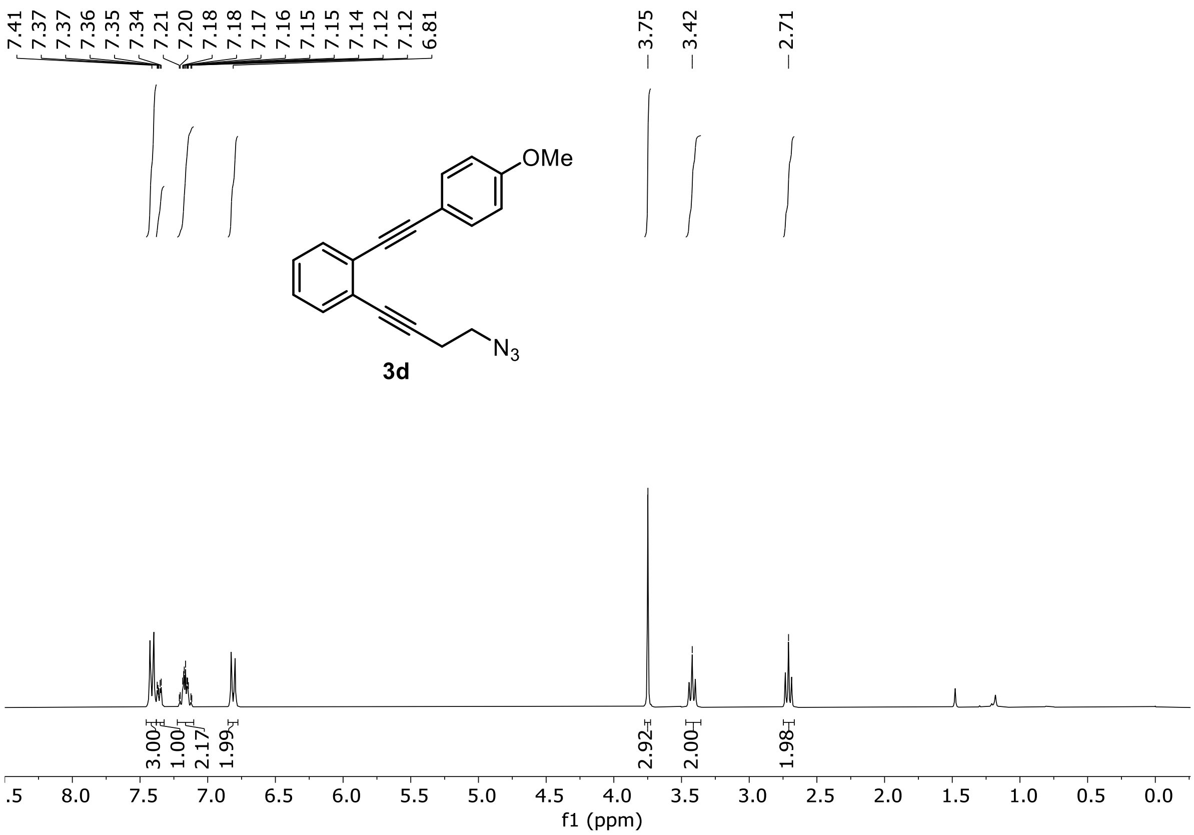

Figure S133. ^{1}H NMR spectrum of **3d** ($CDCl_3$, 300 MHz, 298 K)

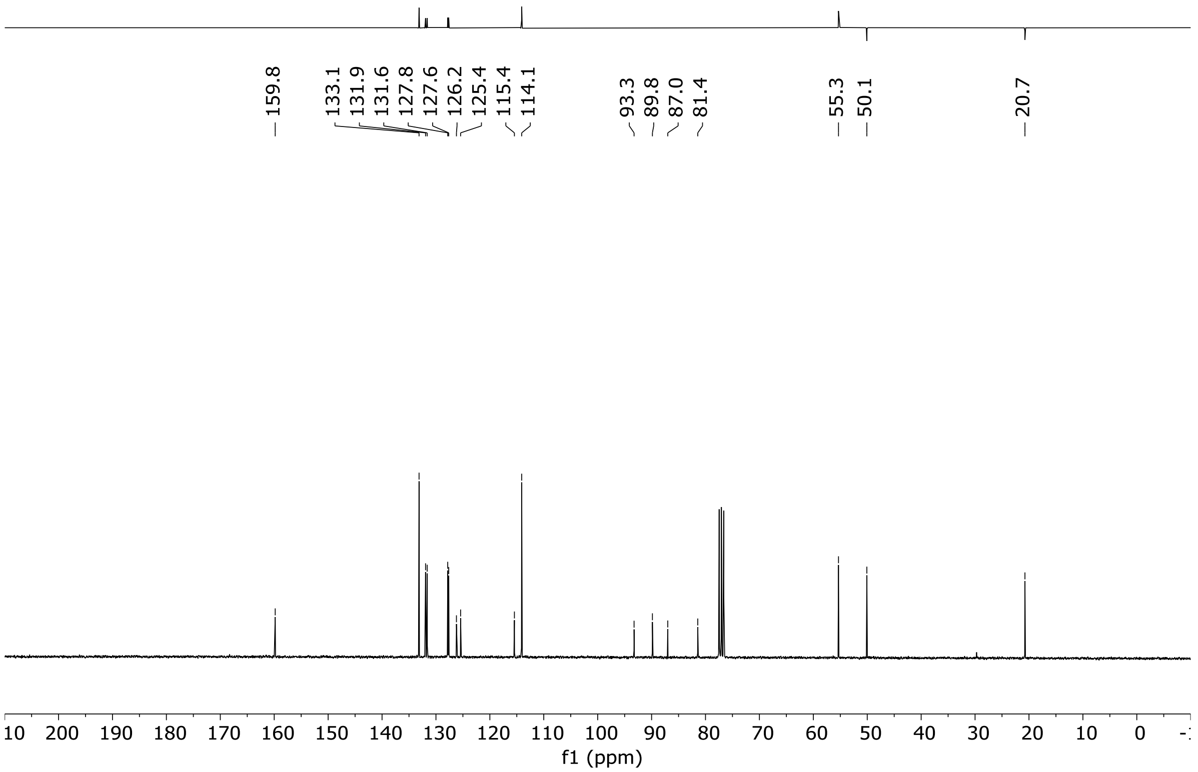

Figure S134. ^{13}C{1H} NMR spectrum of **3d** ($CDCl_3$, 75 MHz, 298 K)

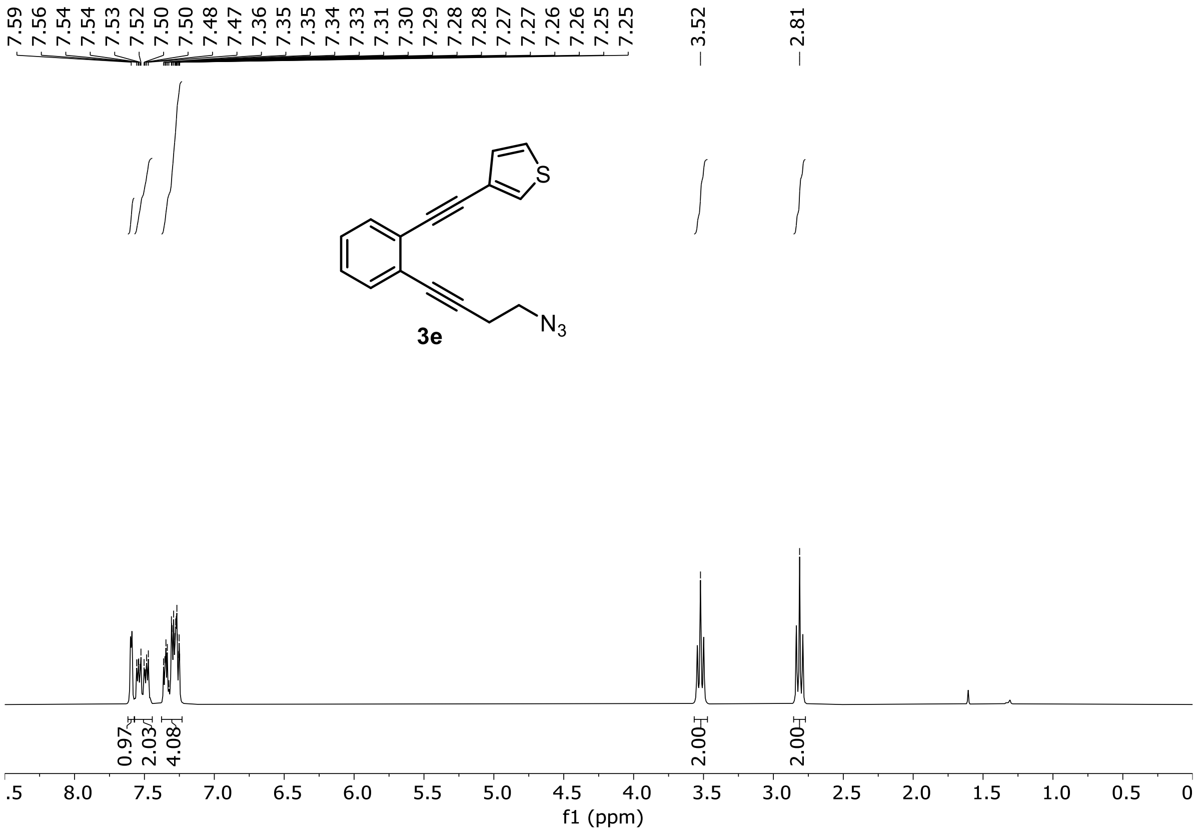

Figure S135. ^{1}H NMR spectrum of **3e** ($CDCl_3$, 300 MHz, 298 K)

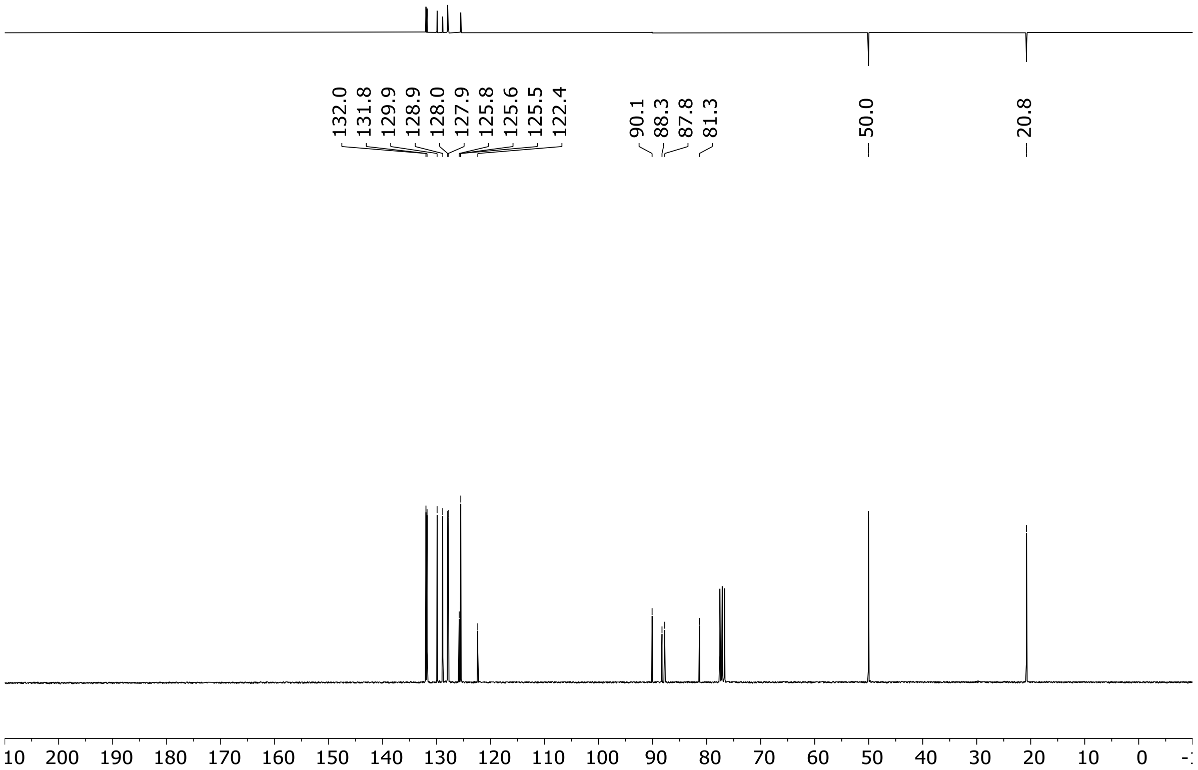

Figure S136. $^{13}C\{1H\}$ NMR spectrum of **3e** ($CDCl_3$, 75 MHz, 298 K)

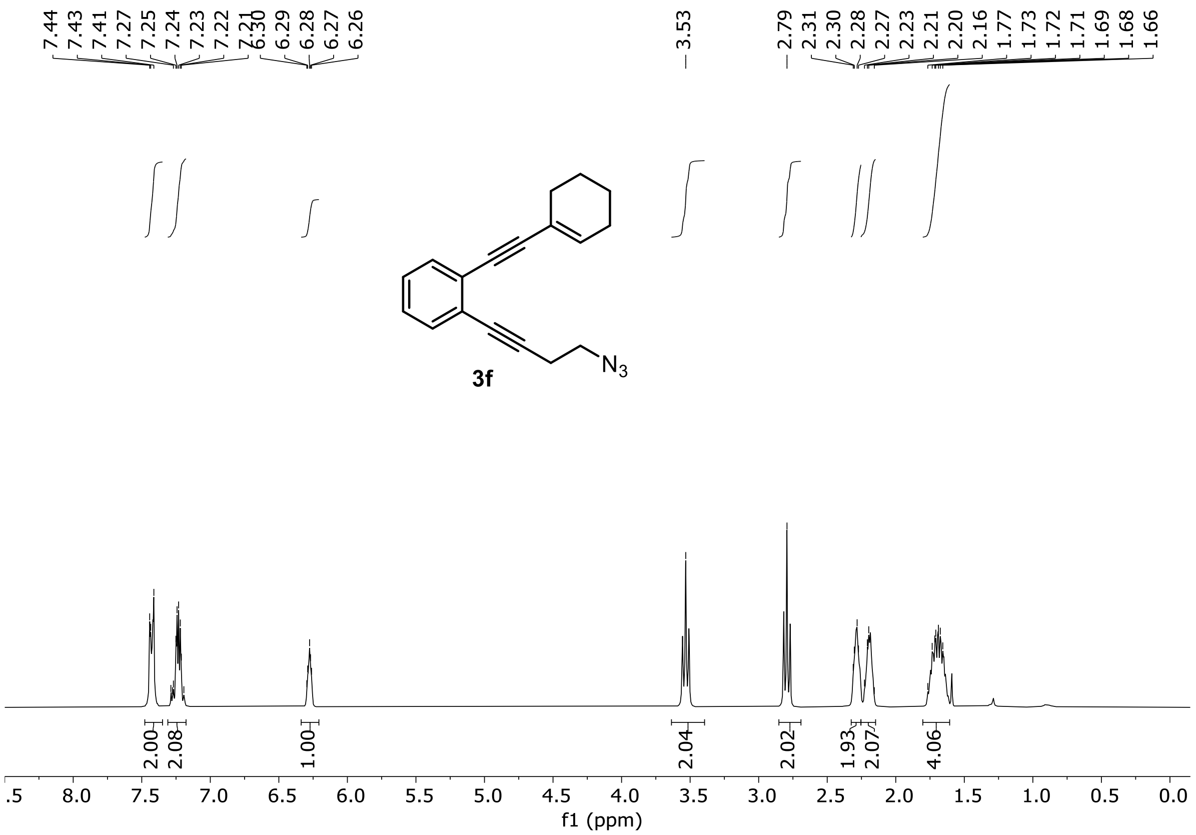

Figure S137. ^{1}H NMR spectrum of **3f** ($CDCl_3$, 300 MHz, 298 K)

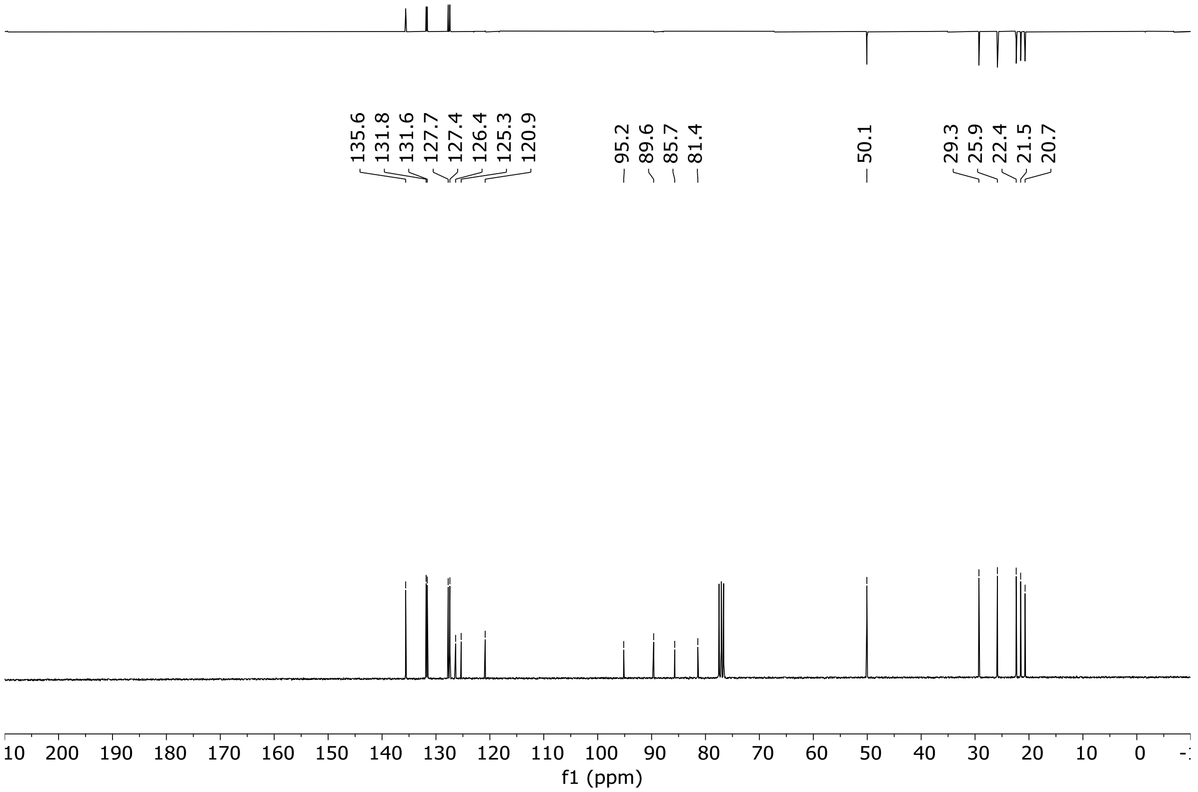

Figure S138. ^{13}C{1H} NMR spectrum of **3f** ($CDCl_3$, 75 MHz, 298 K)

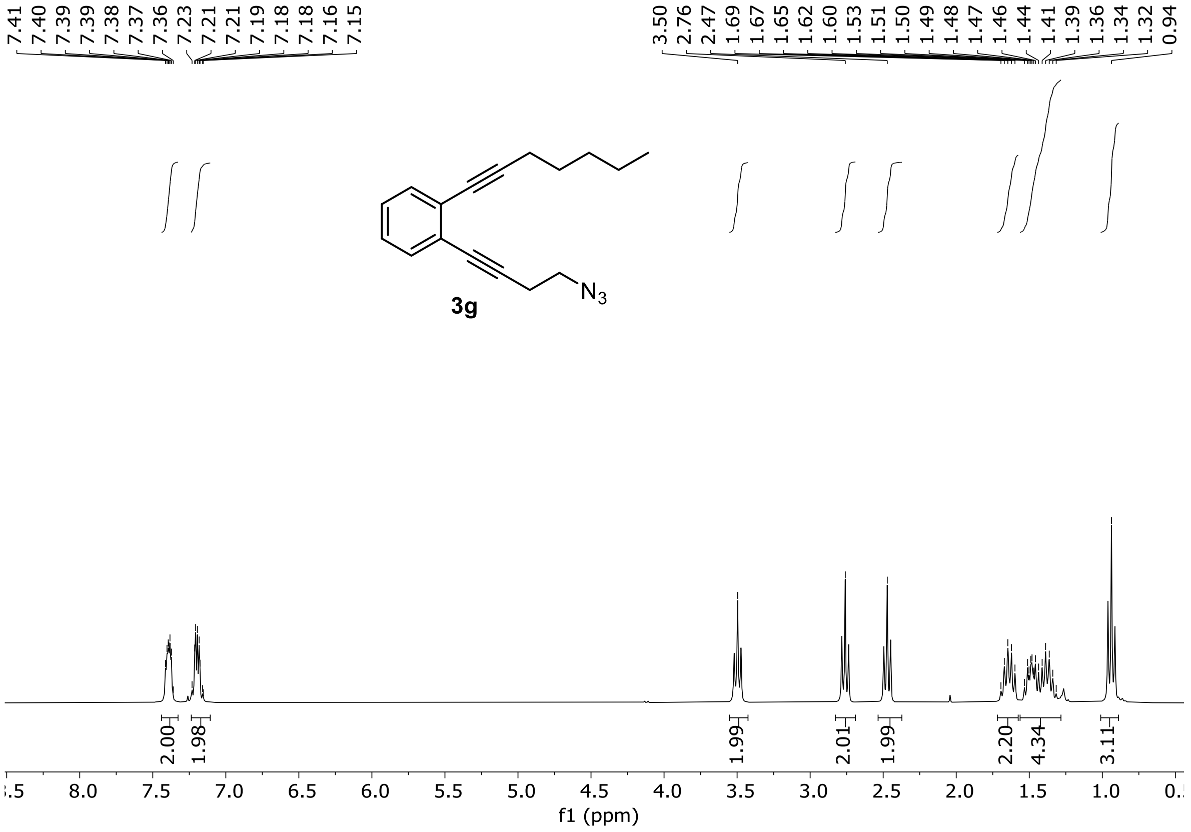

Figure S139. ^{1}H NMR spectrum of **3g** ($CDCl_3$, 300 MHz, 298 K)

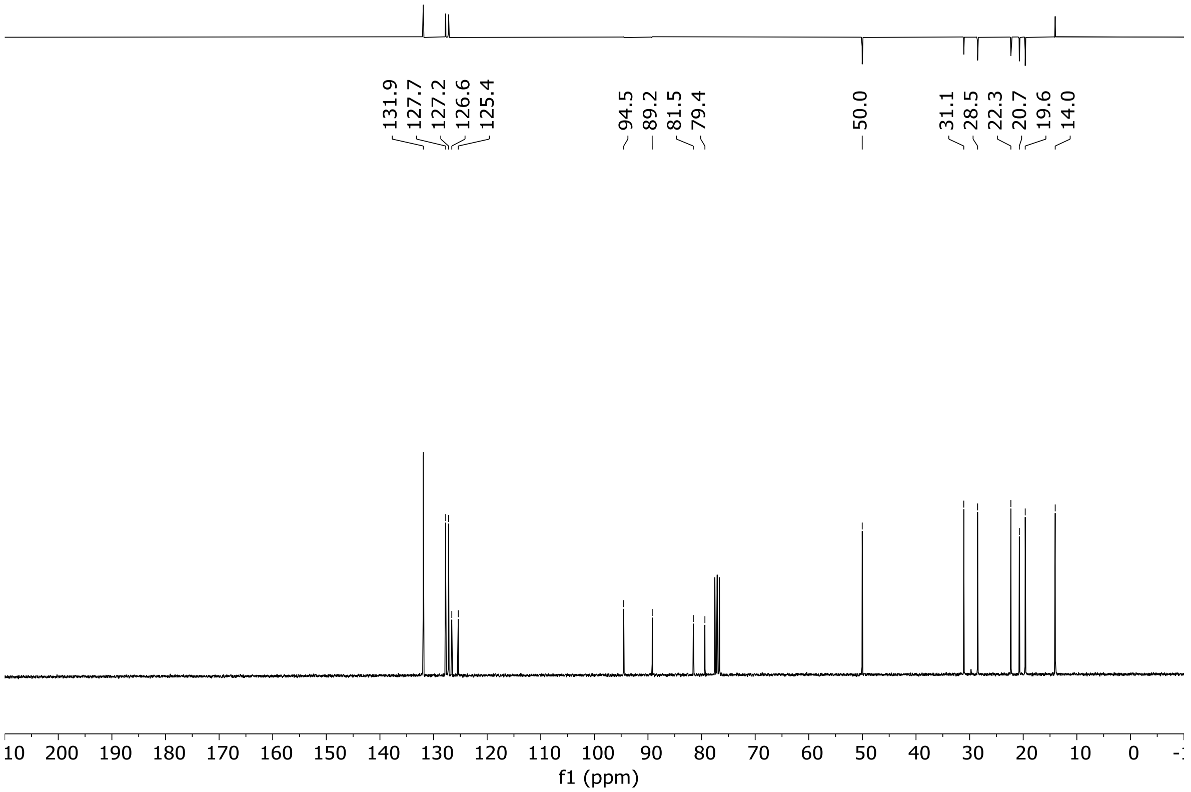

Figure S140. $^{13}C\{1H\}$ NMR spectrum of **3g** ($CDCl_3$, 75 MHz, 298 K)

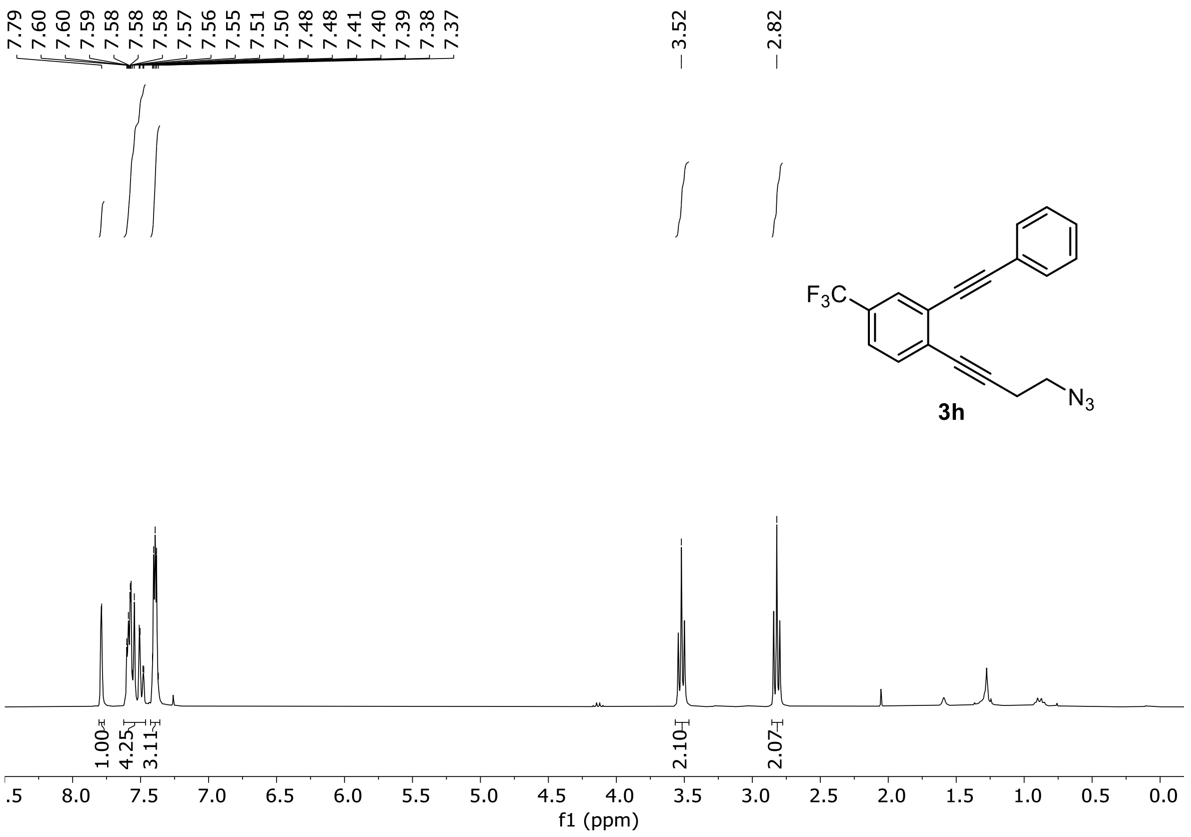

Figure S141. ^{1}H NMR spectrum of **3h** ($CDCl_3$, 300 MHz, 298 K)

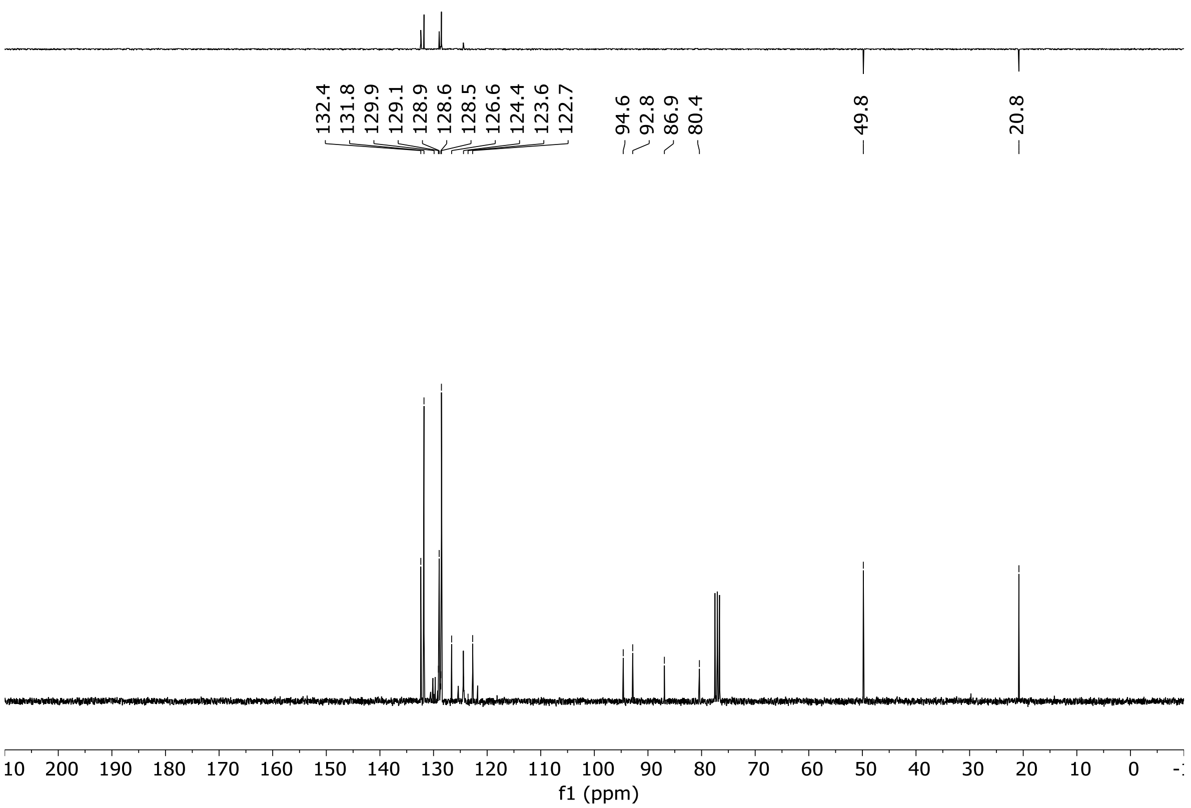

Figure S142. $^{13}C\{1H\}$ NMR spectrum of **3h** ($CDCl_3$, 75 MHz, 298 K)

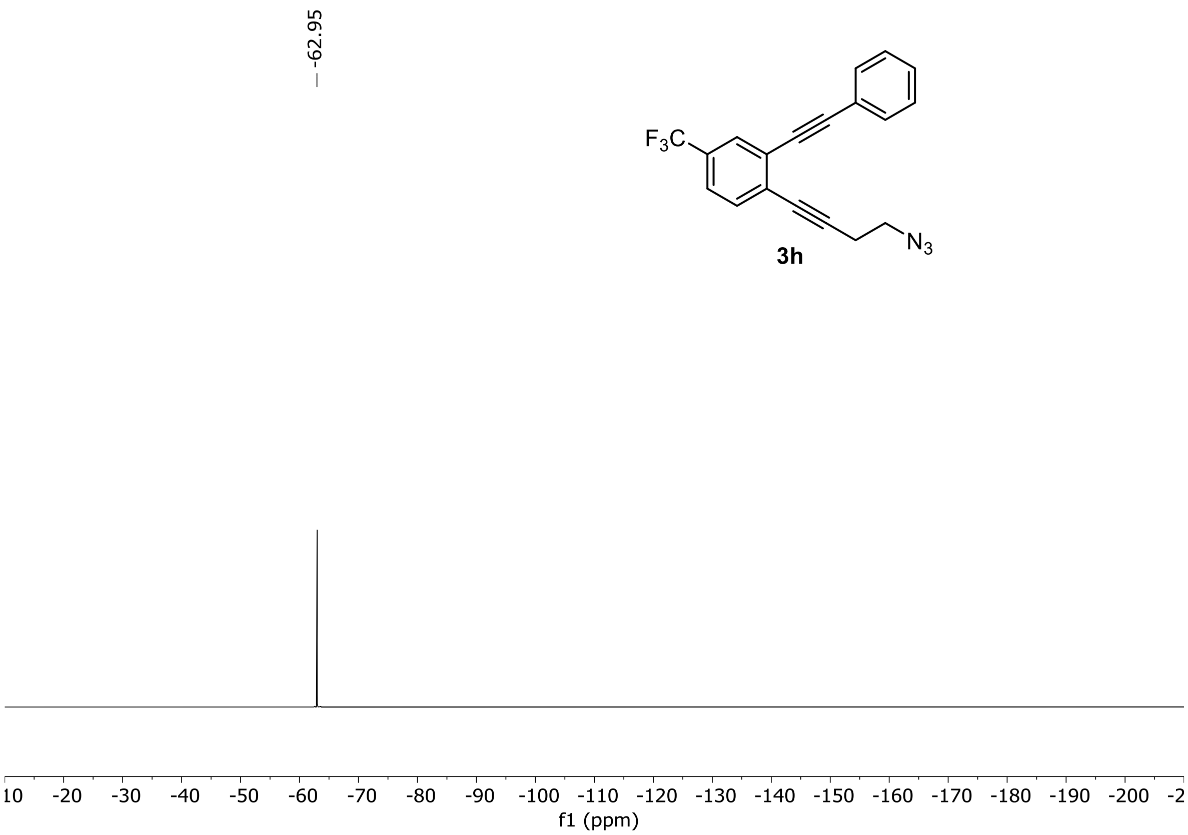

Figure S143. ^{19}F NMR spectrum of **3h** ($CDCl_3$, 282 MHz, 298 K)

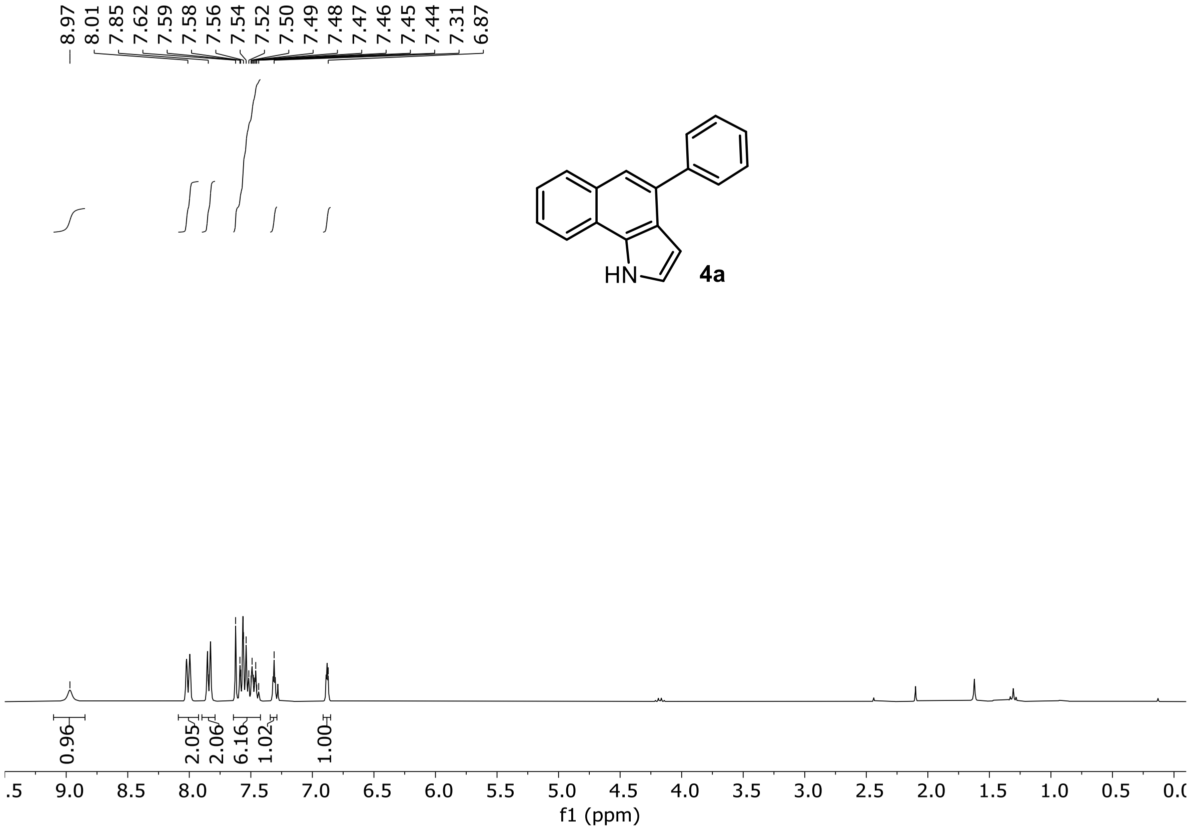

Figure S144. ^{1}H NMR spectrum of **4a** ($CDCl_3$, 300 MHz, 298 K)

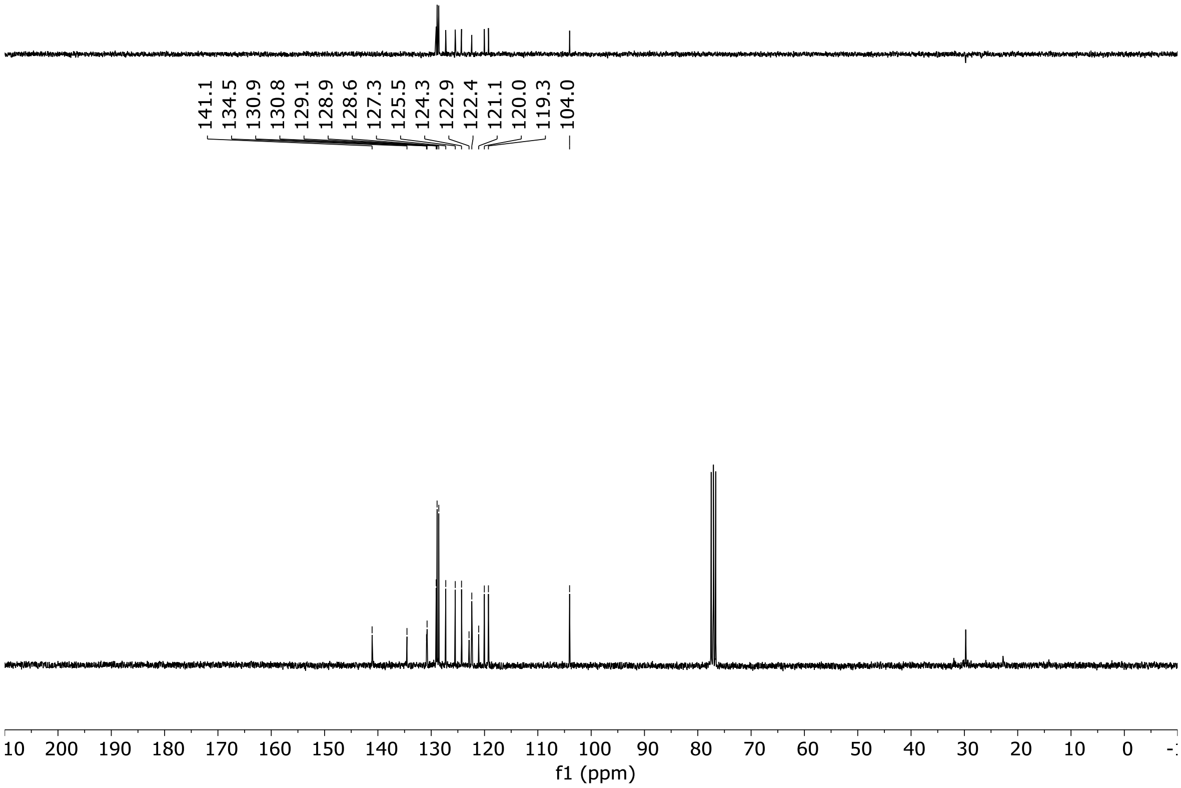

Figure S145. $^{13}C\{1H\}$ NMR spectrum of **4a** ($CDCl_3$, 75 MHz, 298 K)

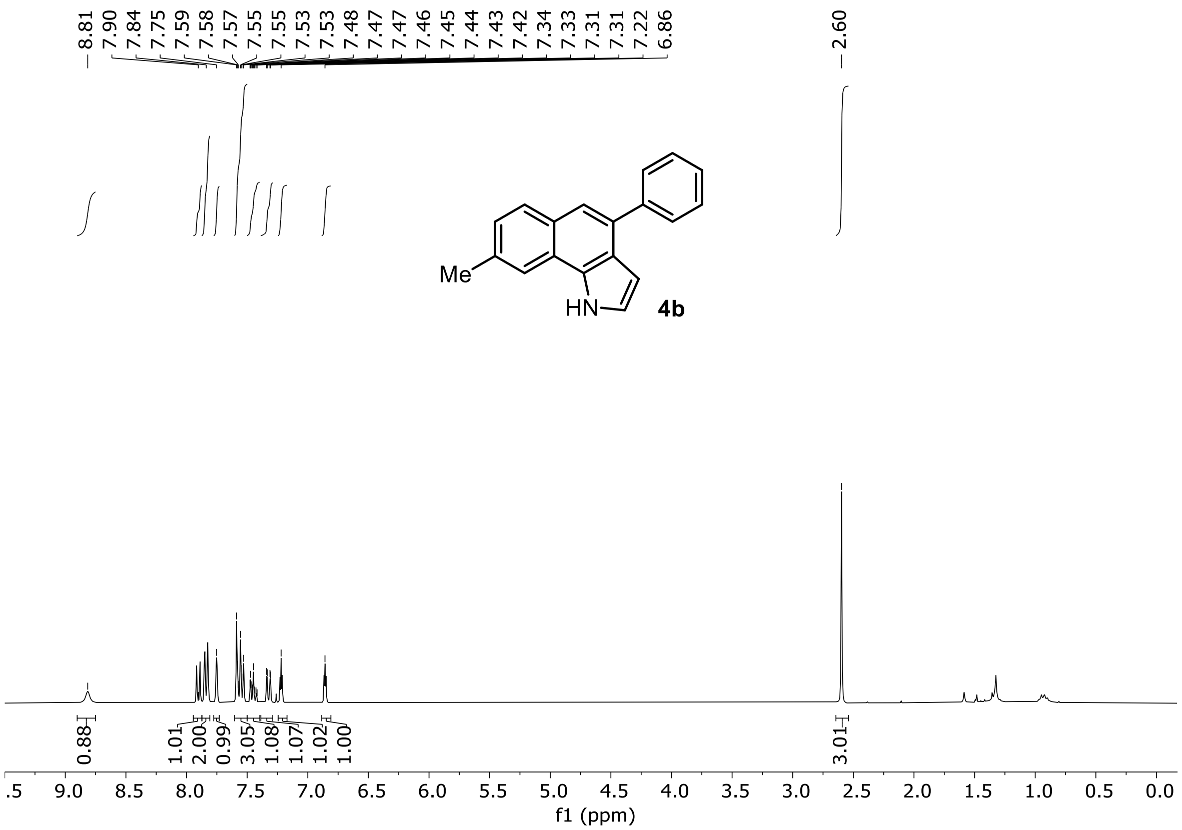

Figure S146. ^{1}H NMR spectrum of **4b** ($CDCl_3$, 300 MHz, 298 K)

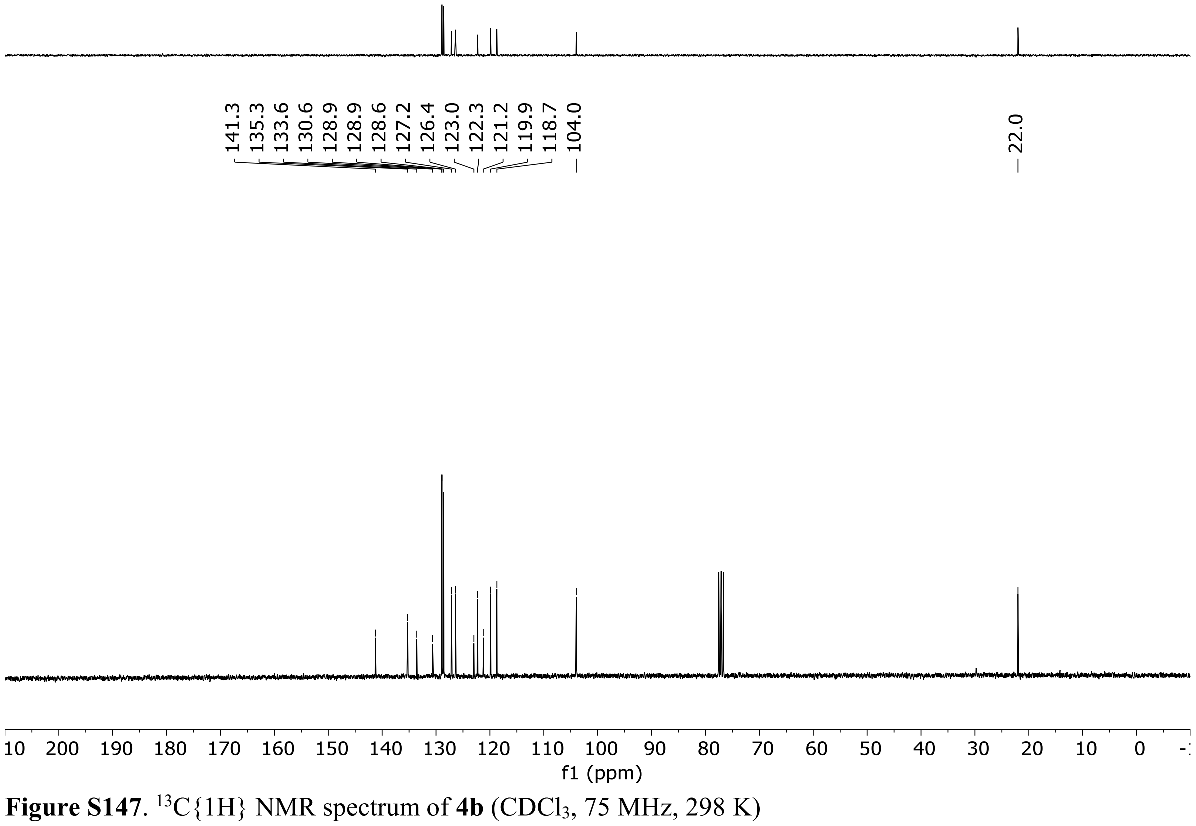

Figure S147. $^{13}C\{1H\}$ NMR spectrum of **4b** ($CDCl_3$, 75 MHz, 298 K)

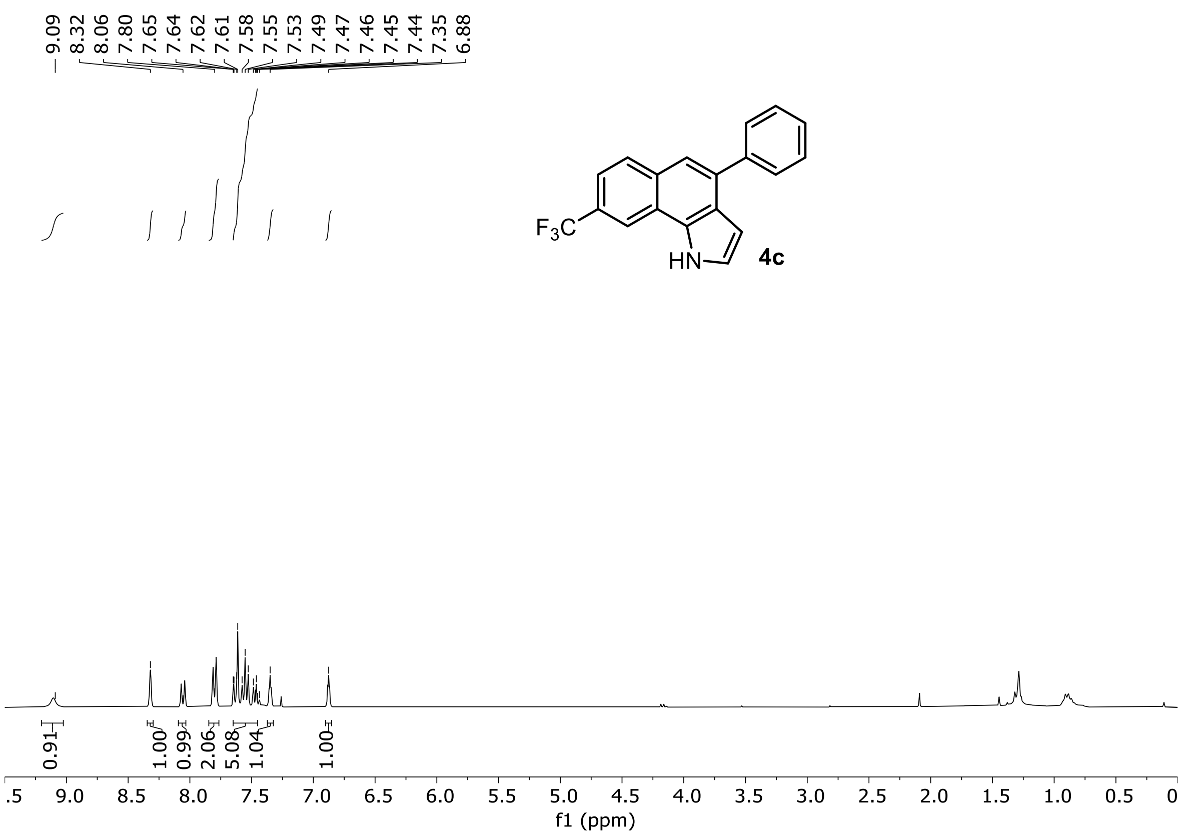

Figure S148. ^{1}H NMR spectrum of **4c** ($CDCl_3$, 300 MHz, 298 K)

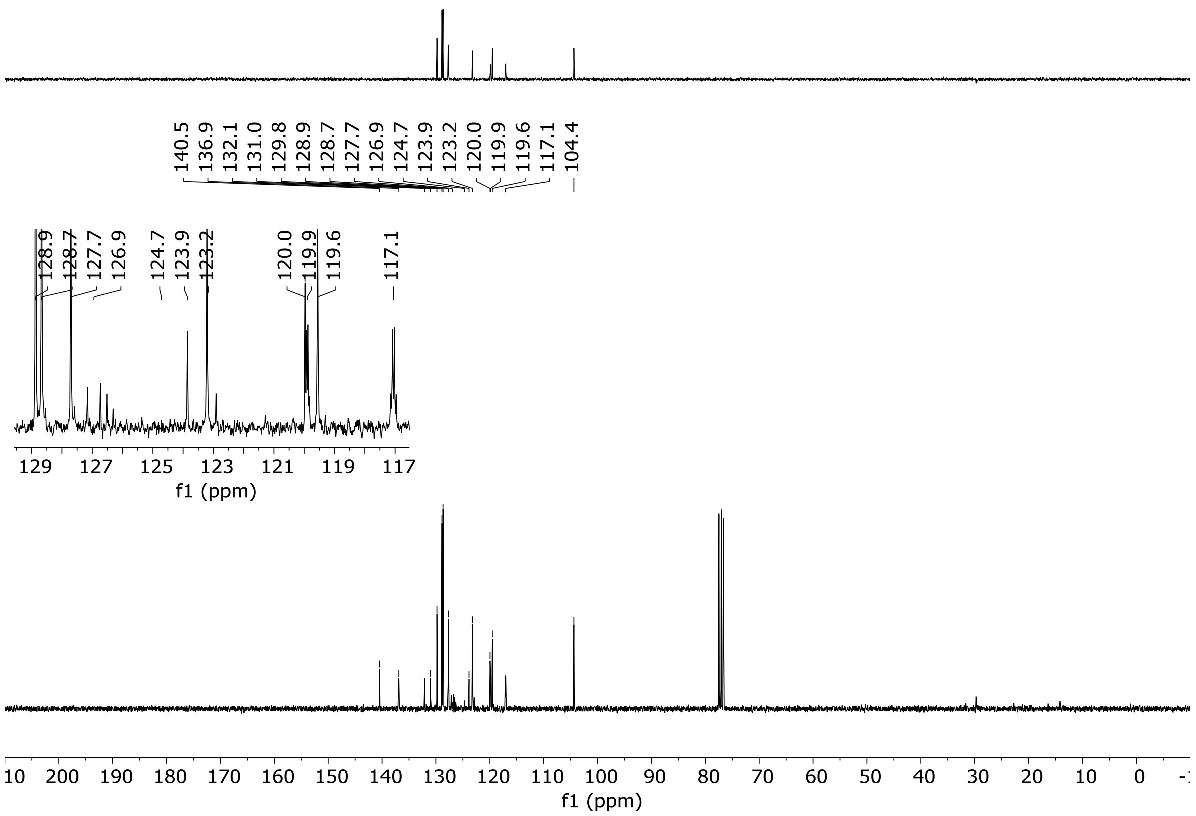

Figure S149 ^{13}C{1H} NMR spectrum of **4c** ($CDCl_3$, 75 MHz, 298 K)

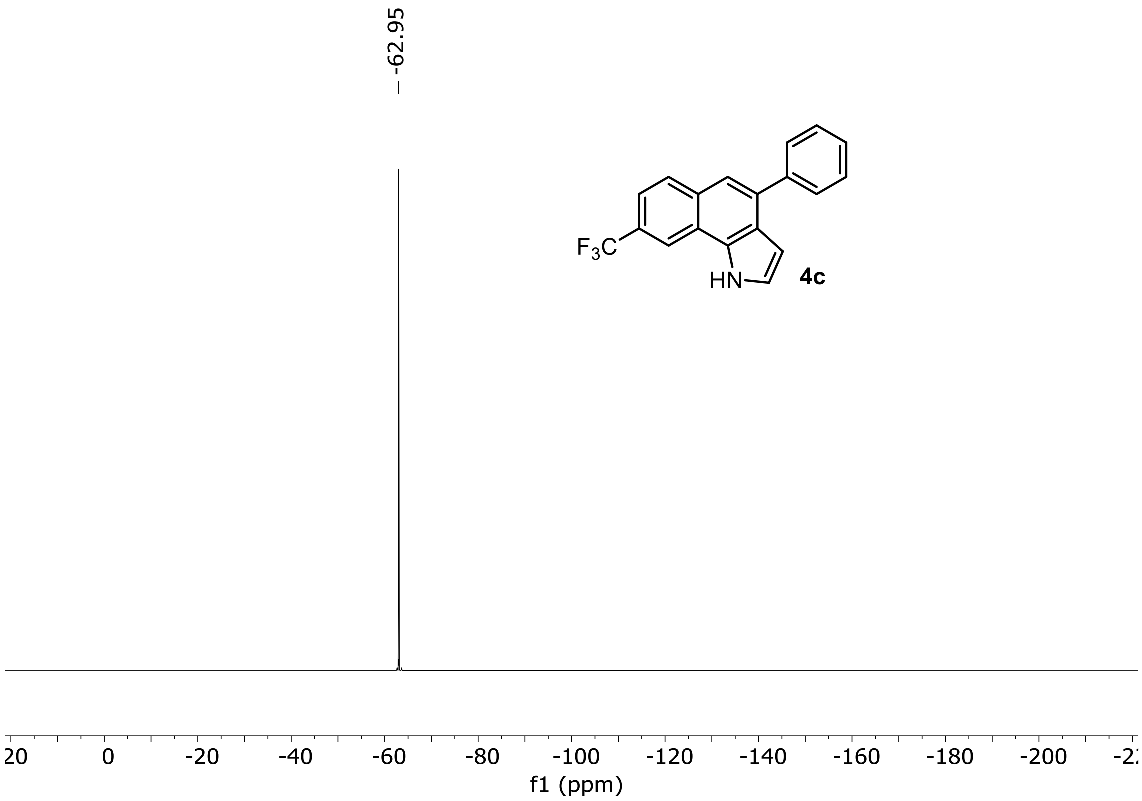

Figure S150. ^{19}F NMR spectrum of **4c** ($CDCl_3$, 282 MHz, 298 K)

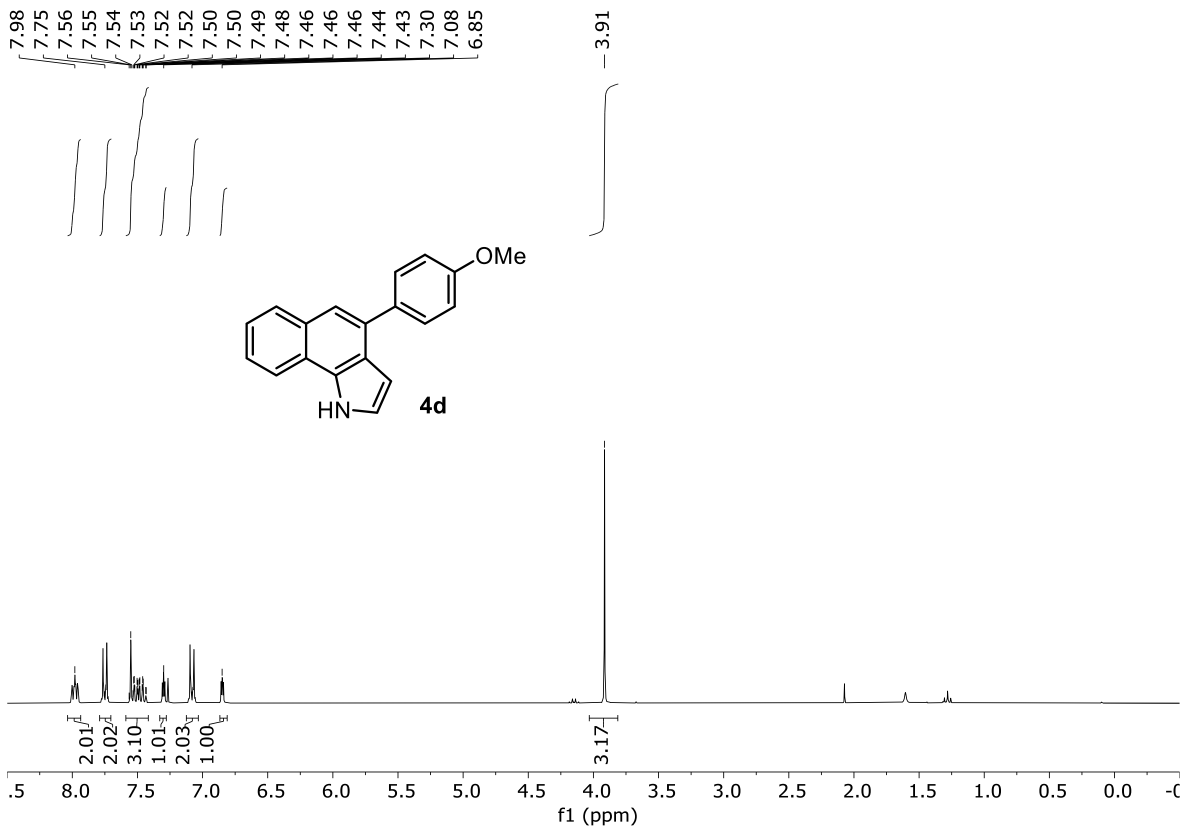

Figure S151. ^{1}H NMR spectrum of **4d** ($CDCl_3$, 300 MHz, 298 K)

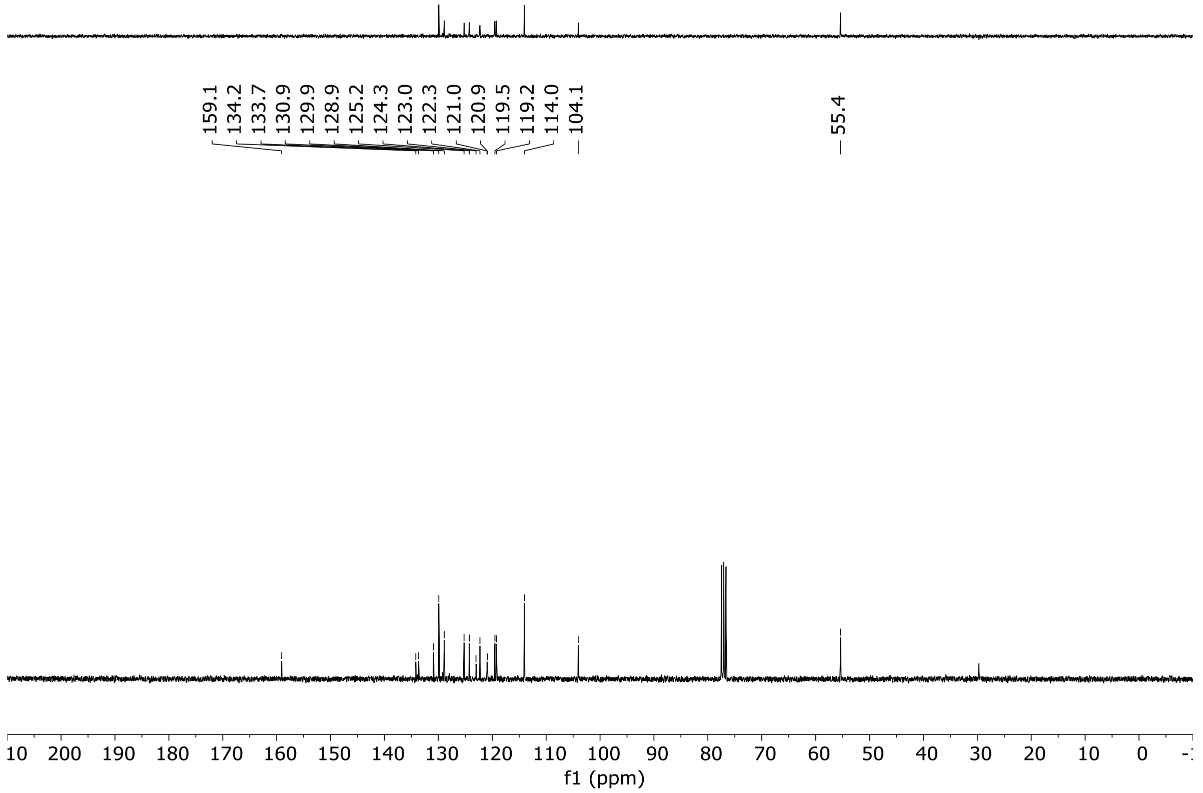

Figure S152. $^{13}C\{1H\}$ NMR spectrum of **4d** ($CDCl_3$, 75 MHz, 298 K)

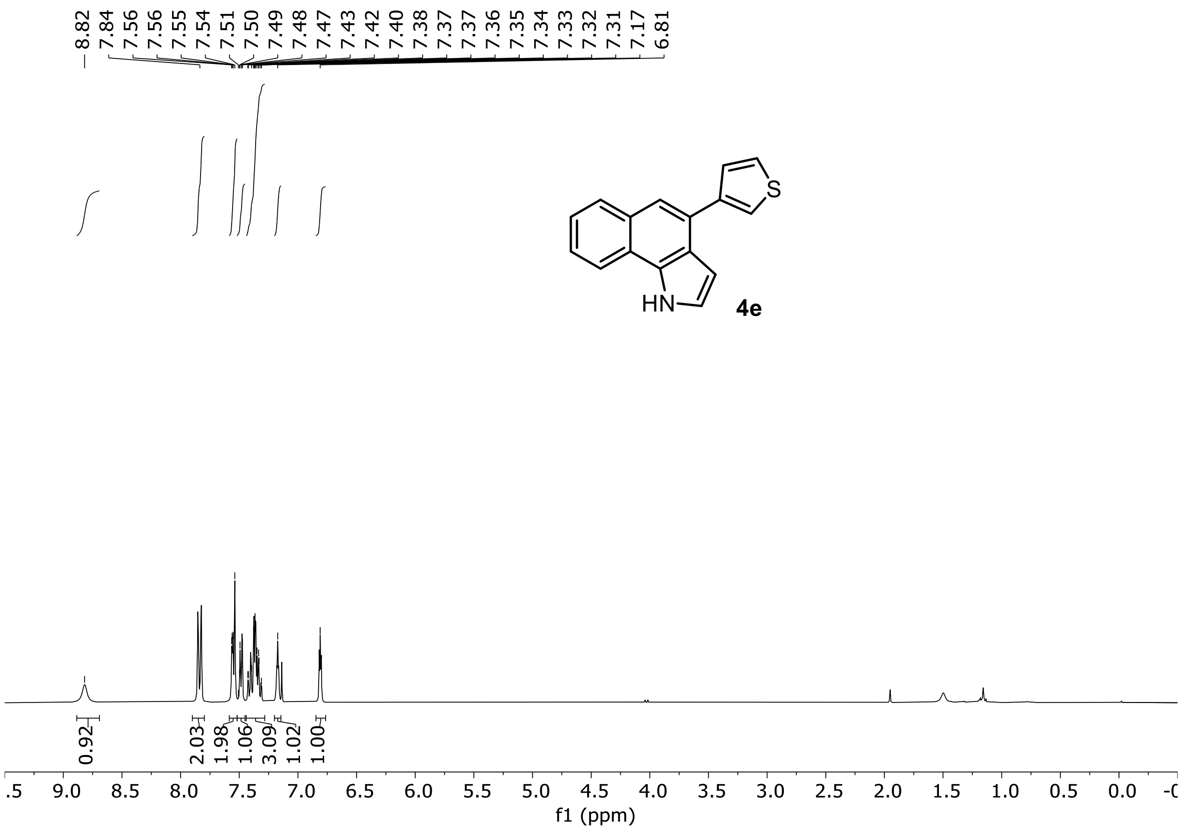

Figure S153. ^{1}H NMR spectrum of **4e** ($CDCl_3$, 300 MHz, 298 K)

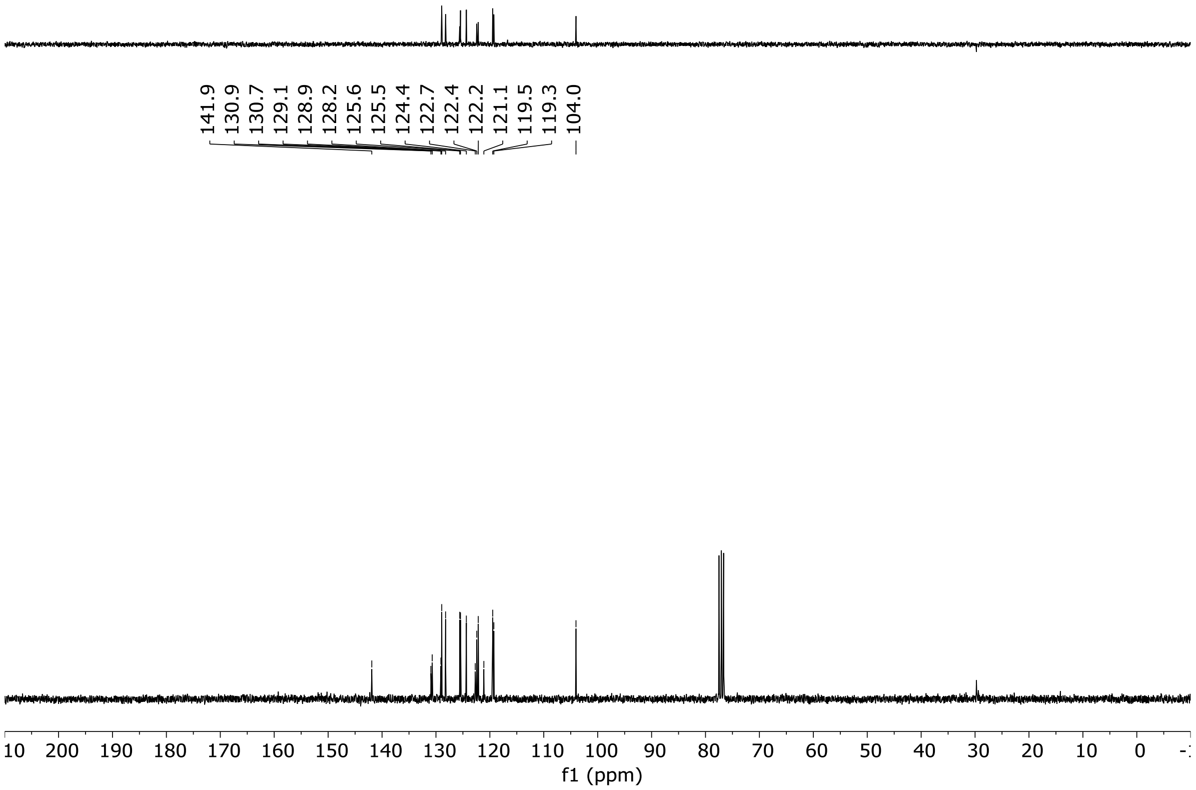

Figure S154. ^{13}C{1H} NMR spectrum of **4e** ($CDCl_3$, 75 MHz, 298 K)

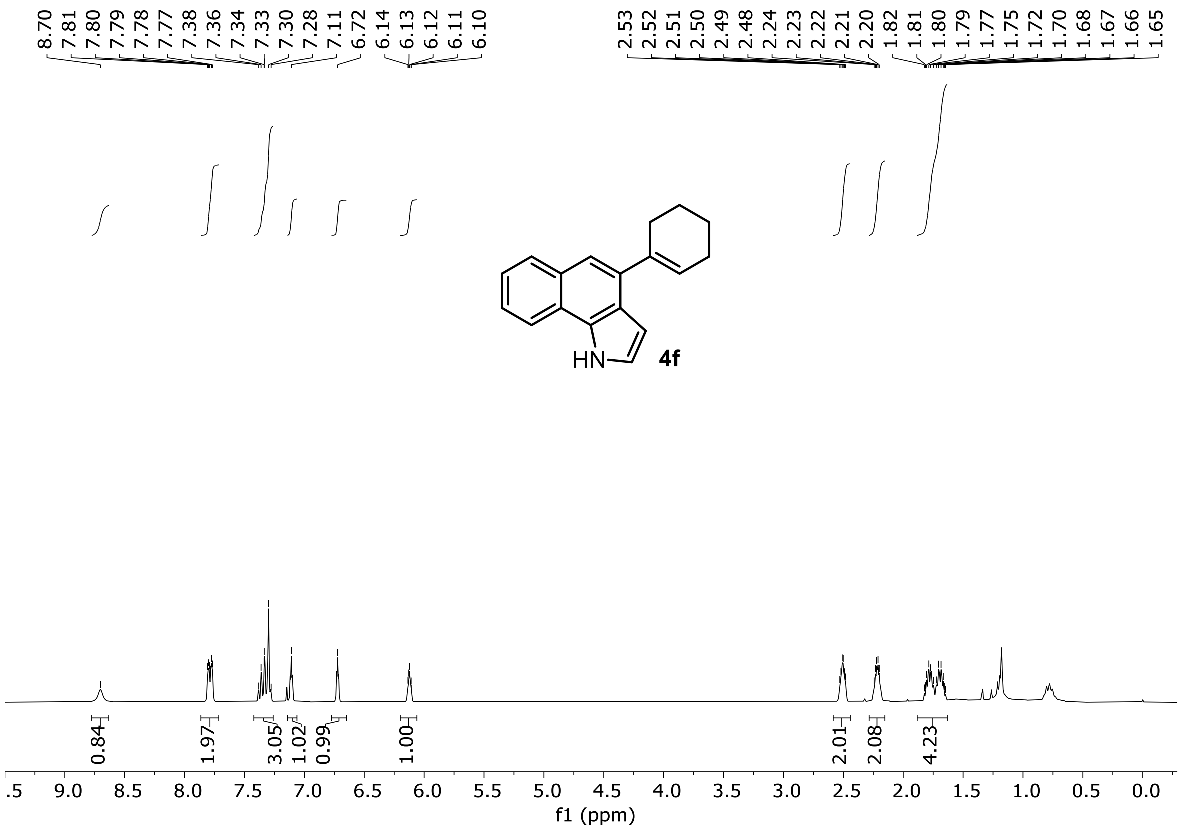

Figure S155. ^{1}H NMR spectrum of **4f** ($CDCl_3$, 300 MHz, 298 K)

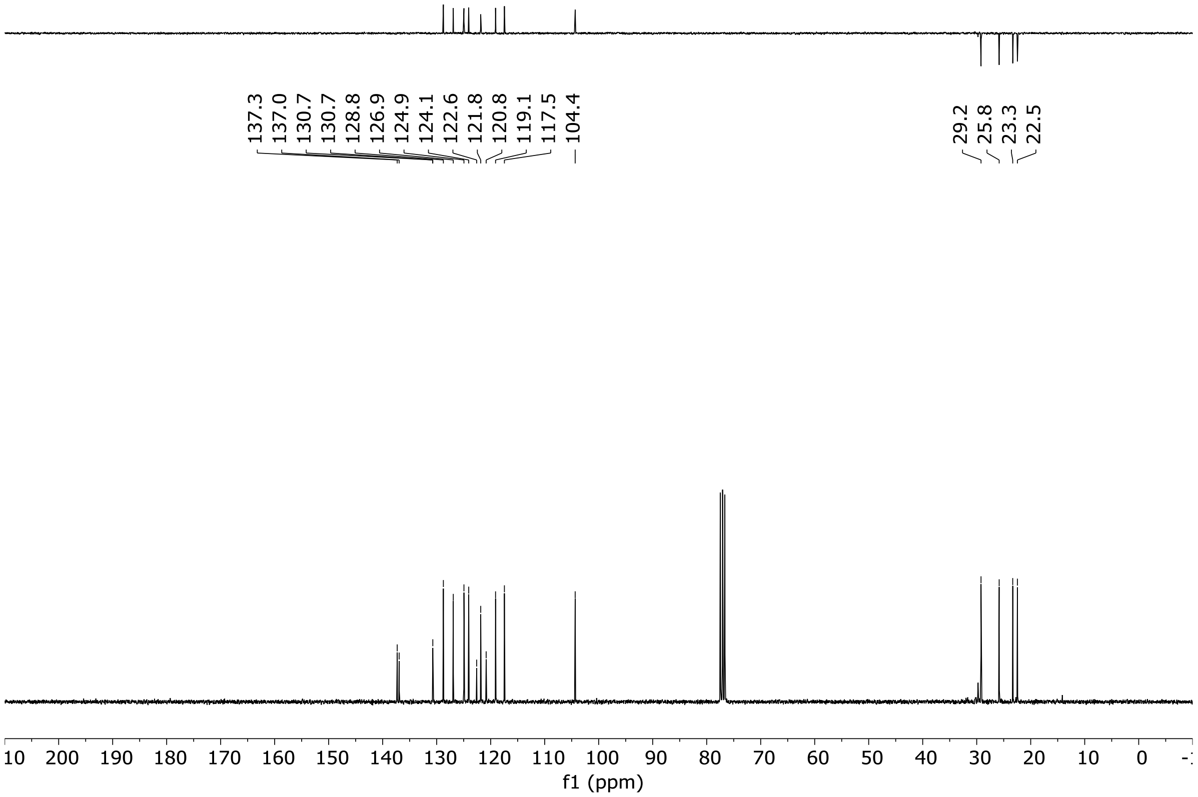

Figure S156. $^{13}C\{1H\}$ NMR spectrum of **4f** ($CDCl_3$, 75 MHz, 298 K)

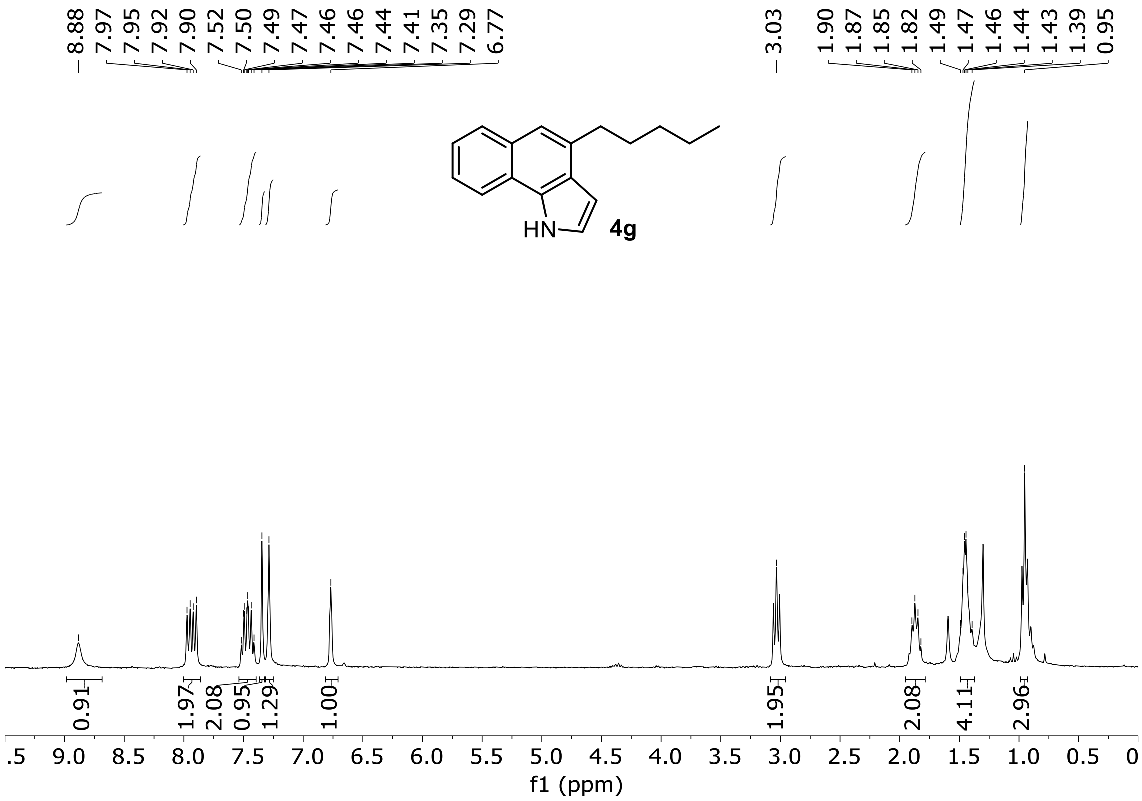

Figure S157. ^{1}H NMR spectrum of **4g** ($CDCl_3$, 300 MHz, 298 K)

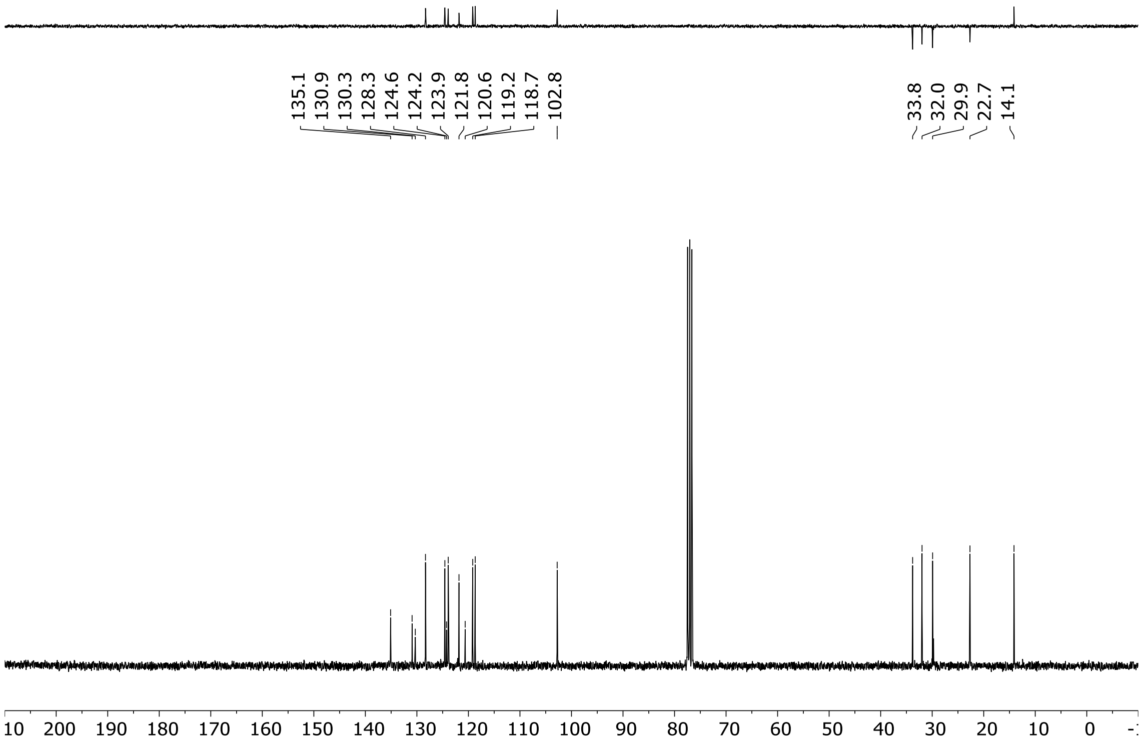

Figure S158. $^{13}C\{1H\}$ NMR spectrum of **4g** ($CDCl_3$, 75 MHz, 298 K)

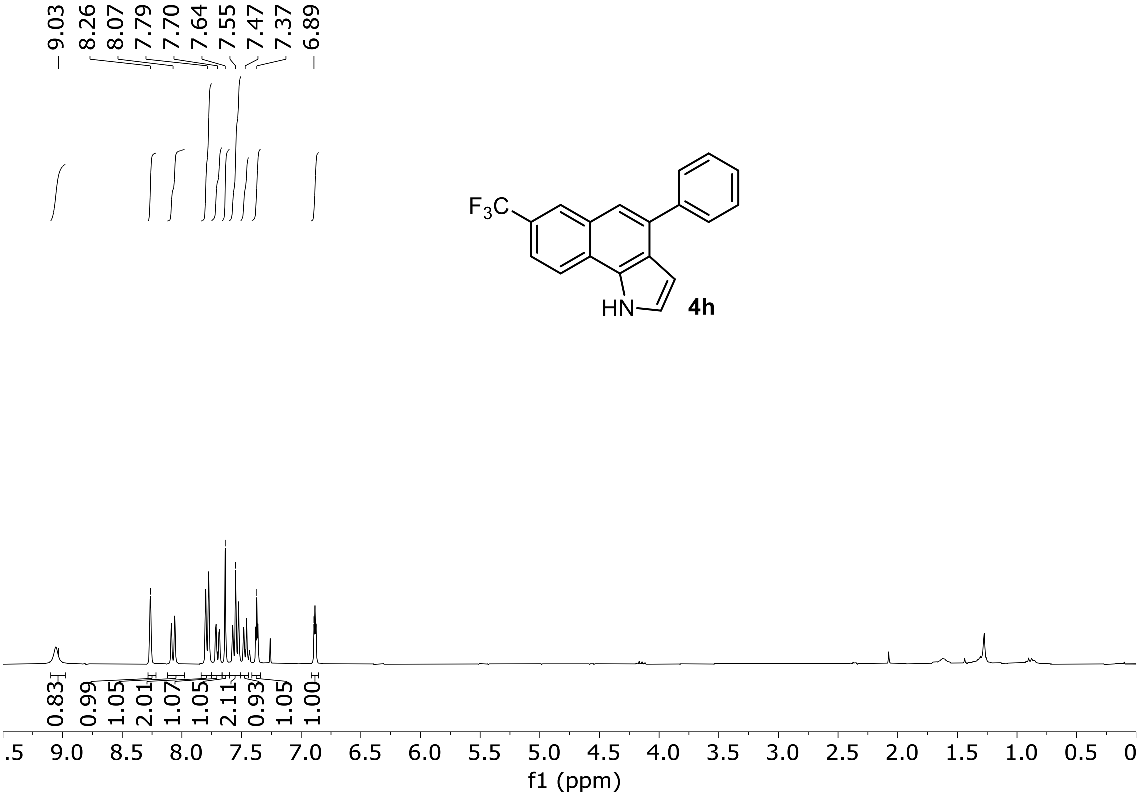

Figure S159. ¹H NMR spectrum of **4h** ($CDCl_3$, 300 MHz, 298 K)

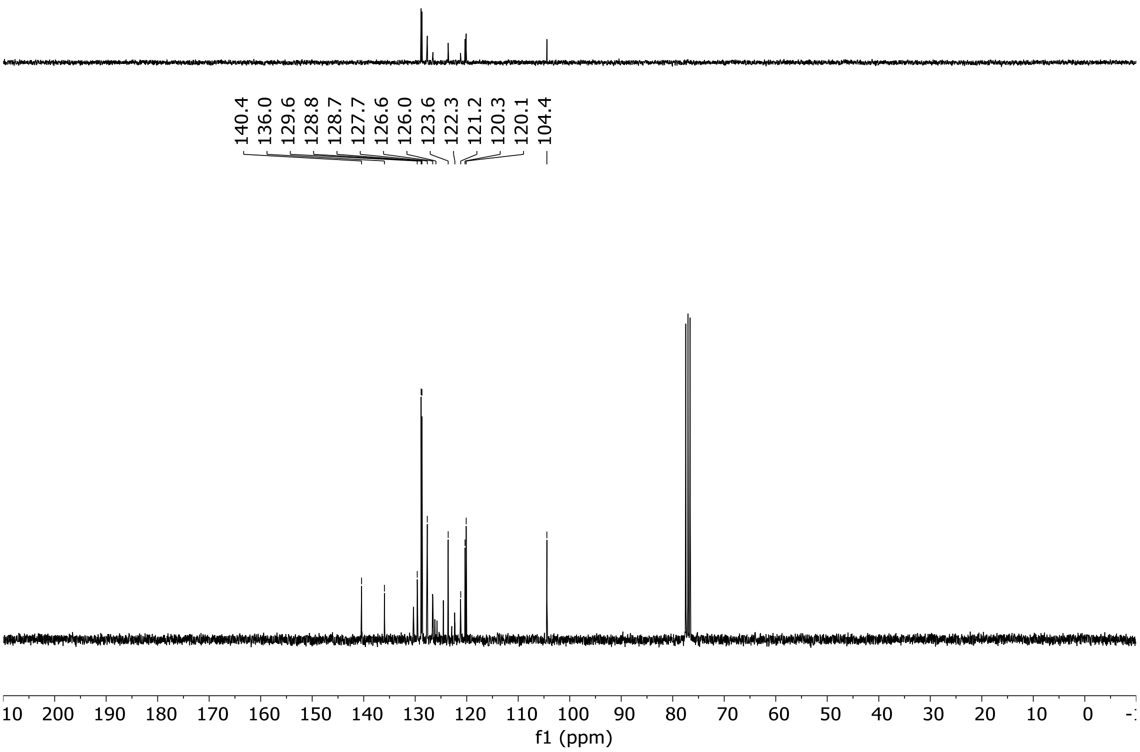

Figure S160. ¹³C{1H} NMR spectrum of **4h** ($CDCl_3$, 75 MHz, 298 K)

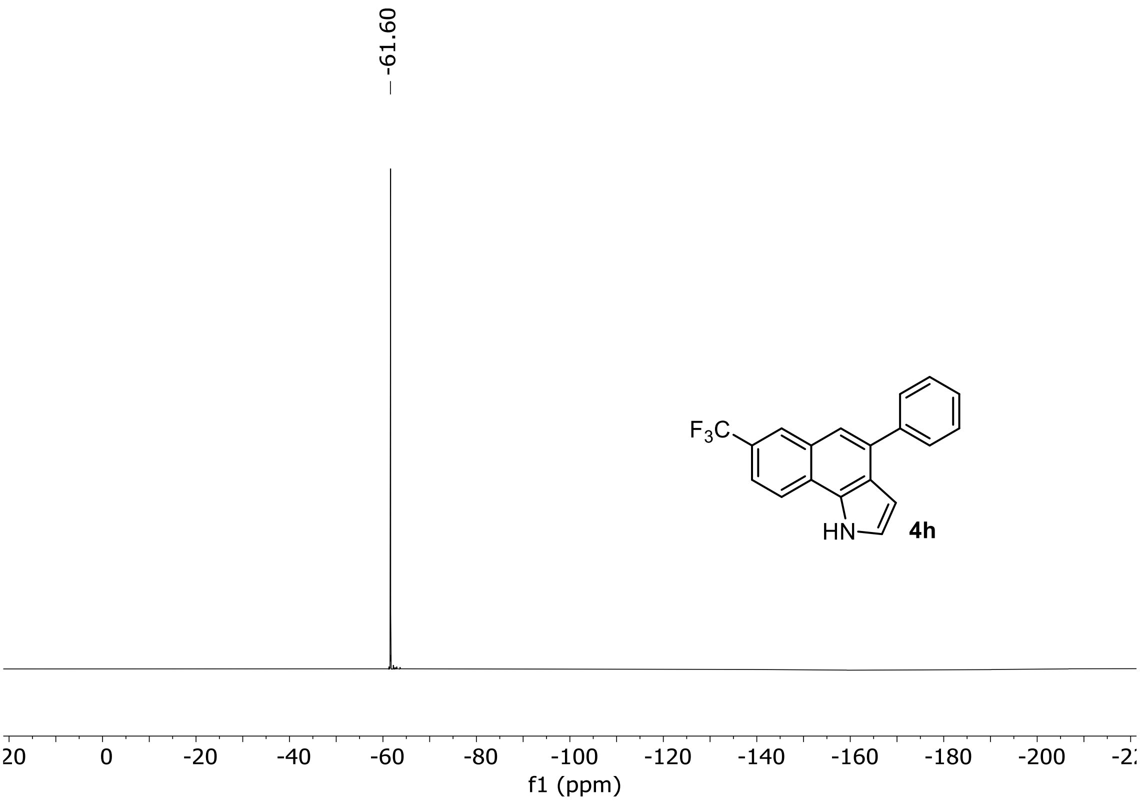

Figure S161. ^{19}F NMR spectrum of **4h** ($CDCl_3$, 282 MHz, 298 K)
